# Mapping and predictive variations of soil bacterial richness across France

**DOI:** 10.1371/journal.pone.0186766

**Published:** 2017-10-23

**Authors:** Sébastien Terrat, Walid Horrigue, Samuel Dequietd, Nicolas P. A. Saby, Mélanie Lelièvre, Virginie Nowak, Julie Tripied, Tiffanie Régnier, Claudy Jolivet, Dominique Arrouays, Patrick Wincker, Corinne Cruaud, Battle Karimi, Antonio Bispo, Pierre Alain Maron, Nicolas Chemidlin Prévost-Bouré, Lionel Ranjard

**Affiliations:** 1 Agroécologie, AgroSup Dijon, INRA, Univ. Bourgogne Franche-Comté, Dijon, France; 2 INRA Orléans - US 1106, Unité INFOSOL, Orleans, France; 3 Agroécologie-Plateforme GenoSol, Dijon, France; 4 CEA / Institut de Génomique / Génoscope, Evry, France; 5 ADEME, Service Agriculture et Forêt, Angers, France; Chengdu Institute of Biology, CHINA

## Abstract

Although numerous studies have demonstrated the key role of bacterial diversity in soil functions and ecosystem services, little is known about the variations and determinants of such diversity on a nationwide scale. The overall objectives of this study were i) to describe the bacterial taxonomic richness variations across France, ii) to identify the ecological processes (*i*.*e*. selection by the environment and dispersal limitation) influencing this distribution, and iii) to develop a statistical predictive model of soil bacterial richness. We used the French Soil Quality Monitoring Network (RMQS), which covers all of France with 2,173 sites. The soil bacterial richness (*i*.*e*. OTU number) was determined by pyrosequencing 16S rRNA genes and related to the soil characteristics, climatic conditions, geomorphology, land use and space. Mapping of bacterial richness revealed a heterogeneous spatial distribution, structured into patches of about 111km, where the main drivers were the soil physico-chemical properties (18% of explained variance), the spatial descriptors (5.25%, 1.89% and 1.02% for the fine, medium and coarse scales, respectively), and the land use (1.4%). Based on these drivers, a predictive model was developed, which allows a good prediction of the bacterial richness (R^2^_adj_ of 0.56) and provides a reference value for a given pedoclimatic condition.

## Introduction

Numerous studies performed over the last two decades in the field of microbial ecology have focused on variations of the soil microbial diversity under different environmental conditions to better understand its regulation and predict the impact of perturbations [1–4]. These works were justified by the lack of knowledge about the determinants of microbial diversity in space and time, but also by the growing awareness of the key role of soil microbial diversity in soil functions (C and N recycling, pathogen management, bioremediation…) [1,5–8] and the supply of ecosystem services. In this context, we have therefore accumulated a huge number of studies dealing with precise perturbations on a plot scale (*e*.*g*. [1]). Soil microbial community as a whole and how it varies has already been examined over regional (*e*.*g*. [9]), territorial (*e*.*g*. [10]) or continental scales (*e*.*g*. [4,11]) by several studies. One of the main pioneer works was performed by Fierer & Jackson (2006) who described soil bacterial diversity on a continental scale by applying DNA fingerprinting to 98 soils sampled along an environmental transect from the north to the south of America [12]. They demonstrated that bacterial diversity was closely related to soil characteristics and especially the pH, as recently confirmed in other studies [10,13]. But, more recently, other studies [9,11,14] have demonstrated the prevalence of other parameters (like climate, geomorphology or land use) on bacterial distributions across regional or global scales. For example, Maestre et al., (2015) highlighted that aridity indirectly impacted the diversity and abundance of soil *bacteria* and *fungi* by strongly affecting soil pH, soil organic C content, and total plant cover using data from 80 dryland sites across the globe [11]. Another study, based on a European soil transect (72 sites showed that soil pH was the main driver of soil bacterial community structure), and established a predictive model of soil bacterial community structure allowing to draw a map at the European scale based solely on this soil parameter [15]. In the same way, two recent studies have developed statistical models to build global spatially explicit predictions of soil microbial biomass [16,17].

However, most of the studies that compared soil microbial diversity and composition were conducted in very different types of ecosystems and soils (generally chosen with *a priori*), which could have facilitated community discrimination and exacerbated the relationship with contrasting environmental filters (soil characteristics, climatic conditions, land cover etc.). In addition, reducing the number of environmental parameters examined and/or their range of variation can lead to contradictory results concerning, for example, the influence of climatic conditions on soil bacterial diversity [12,18]. Drawing a robust conclusion, as to the ecological processes involved (deterministic *vs* neutral processes) or the hierarchy of environmental filters driving soil microbial diversity on a nationwide scale, currently seems impossible from these studies. However, more recently some studies using large soil sampling on a regional scale started to decipher the ecological processes (deterministic *vs* neutral processes) driving soil microbial diversity. Recently, a study showed that habitat turnover was the primary driver of bacterial community turnover, but its importance decreased with increasing isolation [19]. These studies paved the way for the importance of conducting new extensive studies on a nationwide scale with high resolution sampling and without *a priori* to improve the robustness and general applicability of the conclusions and then the understanding of soil microbial community regulation.

**Fig 1:**
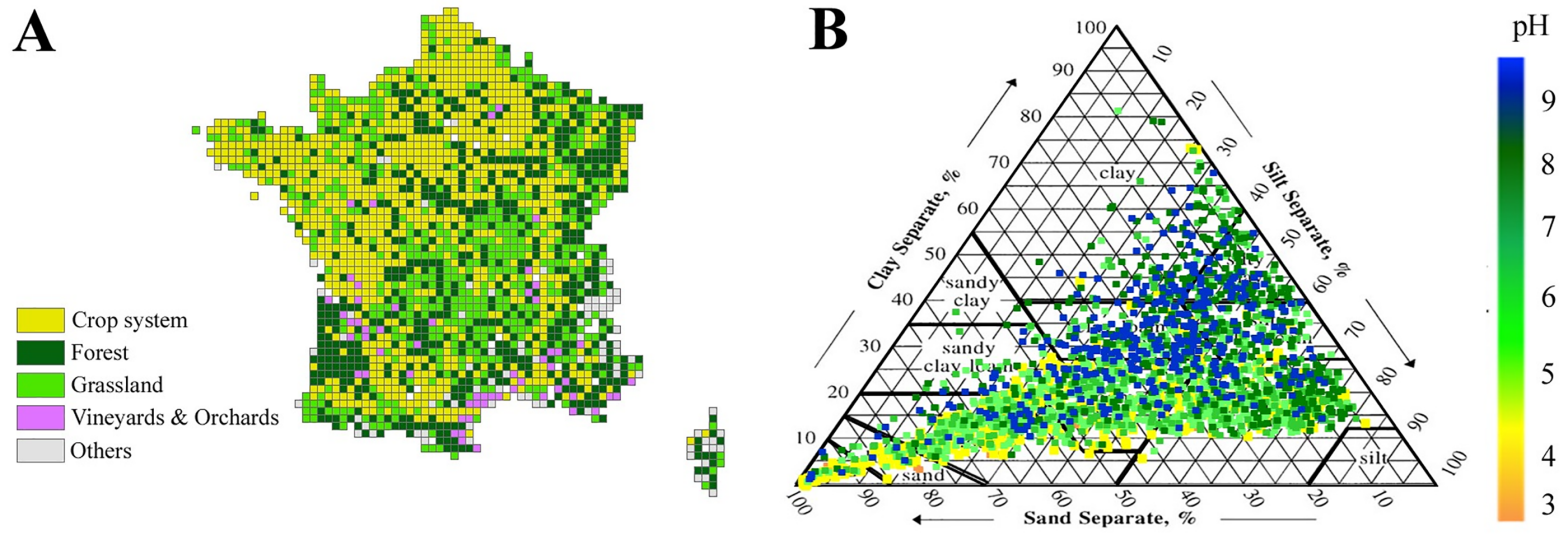
Locations, land uses and texture of sampling sites from the French Soil Quality Monitoring Network (RMQS). (**A**) Location of sampling sites in the systematic sampling grid of the French Soil Quality Monitoring Network (RMQS) criss-crossing the whole French territory. Colour legend indicates the various types of land use encountered in France on this scale. “Others” land use corresponds to sites impossible to sample which corresponded to inaccessible sites (mountain, sea, etc.) or sites without natural soils (urban zone, rocky zone…). (**B**) Distribution of the RMQS soils in the USDA soil texture triangle. Colour legend from yellow to blue represents the soil pH of each RMQS soil.

In France, the French Soil Quality Monitoring Network (Réseau de Mesures de la Qualité des Sols = RMQS) represents the most extensive and without *a priori* soil sampling survey available to date and fulfils most of the above-cited requirements [20]. It consists of a systematic sampling grid (16 x 16 km) extending over the whole of France with 2,173 sites covering an area of ≈5.3 x 10^5^ km^2^ with a huge diversity of soil physico-chemical characteristics, plant cover, land use, geomorphology and climatic conditions and coupled with an extensive collection of corresponding environmental data (Fig 1) [21]. In previous studies, by applying molecular tools to characterize the microbial communities in all RMQS samples, we demonstrated that soil molecular microbial biomass was heterogeneously distributed on the scale of France with biogeographical patterns of about 160 km radius, mostly driven by the soil texture, the pH, the organic carbon content of the soil and by the land use with a negative impact of agricultural land use conversely to natural or semi natural land use [22,23]. Based on these drivers we developed an original predictive polynomial model that provides a reference value for microbial biomass for a given pedoclimatic condition, which can then be compared with the corresponding measured value to provide a robust diagnosis of soil microbiological status [17]. By applying a DNA-fingerprinting approach, we also proved a heterogeneous distribution of soil bacterial community structure, which was independent of soil microbial biomass distribution but driven by soil physico-chemical properties and land use [22]. By comparing estimates of the taxa-area relationship with habitat heterogeneity, we demonstrated that the turnover rate of bacterial diversity in soils on a nationwide scale was (i) highly significant and strongly correlated with the turnover rate of soil habitat [24], and (ii) driven by dispersal limitation as well as environmental selection, this latter including soil and land use properties [13]. Since all these studies were based on quantitative and community structure characterization of bacterial communities, they did not provide information about bacterial diversity in terms of richness, evenness and taxonomic composition.

The aim of the present study was to use the RMQS monitoring network to evaluate the variations and to decipher the spatial patterns of bacterial richness in soils across French national territory. More precisely, our study focused on bacterial taxonomic richness (in terms of number of Operational Taxonomic Units or OTUs at 95% of sequence similarity, corresponding roughly to the *genus* level) [1,6]. Bacterial richness was determined in all 2,173 soils samples of the RMQS by using a pyrosequencing of bacterial 16S rRNA genes directly amplified from soil DNA. Geostatistics was applied to these data to provide the first comprehensive map of soil bacterial richness variation along the environmental gradients encountered in France. The ecological processes structuring the variation of bacterial richness were identified and ranked by variance partitioning analysis. Finally, a statistical predictive model was developed according to the environmental filters identified. This model represents an operational tool highly complementary with the predictive model of soil molecular microbial biomass developed previously [16,17] to establish a comprehensive diagnosis of the soil microbiological status (in terms of abundance and diversity of soil microorganisms).

## Materials and methods

### Soil sampling strategy

Soil samples were obtained from the French Soil Quality Monitoring Network (“Réseau de Mesures de la Qualité des Sols” = RMQS) which is a soil monitoring network based on a 16 km regular grid across the 550,000 km^2^ French territory [25]. The RMQS includes 2,173 monitoring sites, collected between 2000 and 2009, each located at the centre of a 16 x 16 km cell (Fig 1). All sites have been geo-positioned with a precision <0.5m and the soil profile, site environment, climatic factors, vegetation and land use described (see Table 1). In the middle of each 16 x 16 km square, 25 individual core samples were taken from the topsoil (0–30 cm) using an unaligned sampling design within an area of 20 x 20 m. The core samples were bulked to obtain a composite sample for each RMQS site. The soil samples were gently air-dried, sieved to 2mm and then stored at -40°C before analysis [23]. Physico-chemical parameters were measured for each composite soil, *e*.*g*. particle-size distribution, pH water, organic C, N, C/N ratio, soluble P contents, calcareous, cation exchange capacity (CEC) or exchangeable cations (Ca, Mg). Physical and chemical analyses are available for 2,131 soils and were performed by the Soil Analysis Laboratory of INRA (Arras, France, http://www.lille.inra.fr/las). Available climatic data for the RMQS were annual rain, evapotranspiration and temperature. These data were obtained for each node of a 12 x 12 km grid defined by Meteo-France, obtained by interpolating observational data using the SAFRAN model [26]. Obtained measures for the period 1992–2004 were then averaged, to integrate all transitory effects into one value corresponding to a global effect of climate on soil microbial communities. Finally, the RMQS site-specific data were linked to the climatic data by finding for each RMQS site on the grid of 16 x 16 km the closest node within the 12 x 12 km climatic grid. Land use was recorded according to the coarse level of the CORINE Land Cover classification (http://land.copernicus.eu/pan-european/corine-land-cover), which consists of a rough descriptive classification into five classes: forests, croplands, grasslands, others and perennial crops (corresponding to vineyards and orchards). All these data were available in the DONESOL database [21].

**Table 1:** Statistical description of environmental parameters for RMQS soil samples. These values are based on the 1,798 sites analyzed. CEC: cation-exchange capacity; ETP: evapotranspiration.

Soil properties (unit)	Minimum	First Quartile	Median	Mean	Third Quartile	Maximum
pH water	3.70	5.40	6.23	6.42	7.80	8.90
Organic Carbon (g.kg^-1^)	2.57	13.60	19.80	26.08	30.70	243.00
Total Nitrogen (g.kg^-1^)	0.11	1.180	1.75	2.20	2.71	16.00
C:N	6.26	9.67	10.56	12.10	13.27	52.72
Total Calcium Carbonate (g.kg^-1^)	0.50	0.50	0.50	56.19	12.38	866.00
Available phosphorous (g.kg^-1^)	0.001	0.014	0.036	0.053	0.077	1.110
CEC (Cmol + kg^-1^)	0.25	5.84	10.30	14.32	20.30	70.10
Clay (g.kg^-1^)	5.0	154.0	213.0	248.9	325.8	819.0
Silt (g.kg^-1^)	2.0	280.0	406.5	410.6	540.8	819.0
Sand (g.kg^-1^)	7.0	152.0	287.0	340.4	502.0	985.0
Total Cd (g.100g^-1^)	0.01	0.12	0.20	0.30	0.35	4.10
Total Cu (g.100g^-1^)	0.50	8.77	13.90	19.93	22.10	491.00
Total Ni (g.100g^-1^)	0.50	11.72	19.70	25.20	31.50	1,530.00
Total Pb (g.100g^-1^)	3.06	21.40	28.20	32.97	38.00	624.00
Total Zn (g.100g^-1^)	2.50	43.83	64.32	74.55	90.20	1,080.00
Total K (g.100g^-1^)	0.02	1.06	1.44	1.60	2.01	5.40
Elevation (m)	-3.0	106.0	194.5	331.1	388.8	2,540.0
Mean Annual ETP (mm)	43.38	50.45	54.31	55.67	58.61	96.11
Mean Annual Rain (mm)	45.78	62.66	71.89	76.77	84.11	183.71
Mean Annual Temperature (°C)	-2.32	9.93	10.72	10.66	11.73	15.49

### Molecular characterization of bacterial community diversity

#### Soil DNA extraction and purification

Microbial DNA was extracted and purified from 1g of the 2,173 composite soils (composed of a bulk of 25 individual core soils) sampled in each RMQS site, using the GnS-GII procedure as described previously [27]. Crude DNA extracts were quantified by agarose gel electrophoresis stained with ethidium bromide and using calf thymus DNA as standard curve [23]. Crude DNA was then purified using a MinElute gel extraction kit (Qiagen, France) and quantified using a QuantiFluor staining kit (Promega, USA), prior to further investigations.

#### PCR amplification and pyrosequencing of 16S rRNA gene sequences

A 16S rRNA gene fragment targeting the V3-V4 regions to characterize bacterial diversity was amplified using the primers F479 (5’-CAGCMGCYGCNGTAANAC-3’) and R888 (5’-CCGYCAATTCMTTTRAGT-3’) [27]. 2,132 soil samples were successfully amplified from the 2,173 DNA soil samples. The 16S PCR products were then purified using a MinElute PCR purification kit (Qiagen, Courtaboeuf, France) and quantified using the QuantiFluor staining kit (Promega, USA). A second PCR of 7 cycles was then duplicated for each sample under similar PCR conditions, with purified PCR products as matrix (7.5ng of DNA were used for a 25μl mix of PCR) and dedicated fusion primers (‘F479/AdaptorB’, ‘R888/MID/AdaptorA’) integrating needed adaptors, keys and multiplex identifiers at 5’ extremities. All duplicated PCR products were then pooled, purified using a MinElute PCR purification kit (Qiagen, Courtaboeuf, France), and quantified using the QuantiFluor staining kit (Promega, USA). For all libraries, equal amounts from 30 samples were pooled, and then cleaned to remove excess nucleotides, salts and enzymes using the Agencourt AMPure XP system (Beckman Coulter Genomics). 100μl of TE buffer (Roche) was used for the elution. Pyrosequencing was then carried out on a GS FLX Titanium (Roche 454 Sequencing System) by Genoscope (Evry, France).

#### Bioinformatics sequence analysis

Bioinformatic analyses were done using the GnS-PIPE developed by the GenoSol platform (INRA, Dijon, France) [28]. Chosen parameters for each step can be found in S1 Table and the details of all steps have been already described previously [27]. Regarding the filtering step, it was then carried out to check all single-singletons (reads detected only once and not clustered) were checked in order to eliminate PCR chimeras and large sequencing errors produced by the PCR and the pyrosequencing, based on the quality of their taxonomic assignments. More precisely, each single-singleton was compared with a dedicated reference database from the Silva curated database using similarity approaches (USEARCH), with sequences longer than 500 nucleotides, and kept only if their identity was higher than the defined threshold (S1 Table). Finally, the number of high-quality reads for each sample was normalized (*i*.*e*. 10,000 high-quality reads for each sample) by random selection to allow efficient comparison of the data sets and avoid biased community comparisons (see S1 File). A total of 1,798 soil samples were finally kept for subsequent analyses.

A post-processing filtering was then applied to this global dataset to account for potentially artefactual data. First all the homogenized high-quality reads from all samples (encompassing a total of 17,980,000 reads) were merged and aligned. Then, as the analysis of microbial community richness relies on the construction of similarity clusters (called OTUs), we chose here to use OTUs to examine the distribution of 16S rRNA gene sequences in our datasets. However, there is no single best definition of ‘species’, ‘*genus’* when this approach is used, because of controversy about thresholds of similarity allowing clear differentiation of taxonomic units [29]. Moreover, a recent study regarding the diversity of bacterial genomes demonstrated that when the standard threshold of 97% is used, some species can fall to different OTUs due to intragenomic or intraspecific differences [30]. So, we decided to apply the 95% threshold of sequence similarity, usually considered as the ‘*genus’* level. This clustering was realized with a PERL program that groups rare reads to abundant ones, and does not count differences in homopolymer lengths. A post-processing step was then applied to remove all singleton OTUs that occurred only once in the overall dataset, and comprised only a singleton (reads detected only once after the dereplication step and not clustered) [27]. This post-processing step reduced the number of total OTUs from 205,590 to 92,571 (loss of 50%), but the number of reads only from 17,980,000 to 17,866,981 (loss of less than 1%). For each sample, the number of deleted reads with this step was 62 ± 60 on average (minimum: 10, maximum: 1,093). Finally, contingency tables of OTUs were obtained with the samples in lines and OTUs in columns, indicating the number of reads in each OTU for all samples. The retained high-quality reads were then used to determine OTU richness and rarefaction curves (see S2 File) [31]. All raw data sets are publicly available in the EBI database system (in the Short Read Archive) under project accession PRJEB21351.

### Metadata analysis

#### Mapping using geostatistic

The geostatistical method of kriging was used to map microbial richness and to characterize their spatial variations [32]. More precisely, as the studied variable followed a normal distribution (Kolmogorov-Smirnov test, *p*-value = 0.2703 for Richness), no transformation was considered prior to modelling the spatial correlations (S1 Fig). In conventional geostatistical analysis, an estimate of a variogram model is computed based on the observations, which describe the spatial variation of the property of interest. This model is then used to predict the property at unsampled locations using kriging [32]. A common method for variogram estimation is first to calculate the empirical (so called experimental) variogram by the method of moments [33], and then to fit a model to the empirical variogram by (weighted) nonlinear least squares. We tried to fit several models and retained the one that minimized the objective function [34]. The validity of the best fitted geostatistical model was then assessed in terms of the standardized squared prediction errors (SSPE) using the results of a leave one out cross validation. If the fitted model was a valid representation of the spatial variation of the microbial property, then these errors would have a χ^2^ distribution with a mean of 1 and median of 0.455 [35]. The mean and median values of the SSPE were also calculated for 1,000 simulations of the fitted model to determine the 95% confidence limits. The ‘gstat’ package of R software (version 3.2.2) was used for geostatistical analysis and kriging [36].

#### Variance partitioning

The relative contributions of soil physicochemical parameters, land use (forests: 492 sites, croplands: 740 sites, grasslands: 464 sites, perennial crops, corresponding to vineyards and orchards: 36 sites, and others: 36 sites), climatic conditions, geomorphology and space in shaping the patterns of soil bacterial richness and evenness were estimated by variance partitioning. The Principal Coordinates of a Neighbour Matrix approach (PCNM) was used to describe and identify the scales of spatial relationship between samples [37]. This PCNM method was applied to the geographic coordinates and only PCNMs with a significant Moran’s index were selected for the variance partitioning analysis (*P*<0.001). The spatial neighbourhood described by each PCNM was determined by the range of a Gaussian variogram models [38]. All quantitative (response and explanatory) data were standardized (centered and scaled) in order to have an approximated Gaussian and homoskedastic residual distribution. A two steps procedure was used to determine the environmental parameters significantly shaping bacterial richness and to limit over fitting and to exclude co-linear variables [39]. The first step consisted of a coarse selection of explanatory variables included in models minimizing the Bayesian Information Criterion (BIC) and maximizing the adjusted R^2^ using the *regsubset* function (“leaps” package) [40]. In the second step, a forward selection procedure was applied to the subset of explanatory variables to identify the model maximizing the adjusted R^2^ [39]. Spatial descriptors were then selected from the model residuals [41] using the forward selection step only since all PCNM are linearly independent. The respective amounts of variance (*i*.*e*., marginal and shared) for bacterial richness, were determined by canonical variation partitioning and the adjusted R^2^ with Redundancy Analysis [39]. The statistical significance of the marginal effects was assessed from 1,000 permutations of the reduced model. All these analyses were performed with R (http://www.r-project.org/) using the vegan package.

#### Predictive modelling strategy

Three steps were assessed in order to find the best explanatory and most parsimonious model that explained the bacterial richness (response variable) as a function of soil physico-chemical characteristics and geographical coordinates (climatic data were not retained since they are rarely available, expensive to obtain, and limit the use of the model in a diagnostic approach [17]) (explanatory variables), i) selection of the significant explanatory variables, ii) selection of the best model form based on its predictive capacity and cross validation, and iii) sensitivity analyses of the model. For the first step, two tools were used to assess colinearity between the explanatory variables, namely correlation coefficients and variance inflation factors (VIF). Only those with a correlation coefficient ranging from -0.7 to 0.7 and with a VIF ≤ 4 were considered in the modeling steps. The VIF values were calculated using the *vif* function in the car R package [42]. This selection step allowed the exclusion of highly collinear variables and defined a reduced explanatory dataset more comprehensive and of easier use for the following steps. Since the number of explanatory variables was large (less than 50), the best explanatory variables were selected by applying the exhaustive search method described by Miller, 2002 [43]. This approach involved using the *regsubsets* function in the leaps package in R [40]. The selection criteria were the Bayesian Information Criterion (BIC) and the adjusted coefficient of determination (R^2^_adj_) by minimizing the first and maximizing the second.

For the second step, the bacterial richness dataset was randomly divided into a modeling dataset (90% of the data, 1,618 soil samples) and a cross-validation dataset (about 10% of the data, 180 soil samples), selected by applying the KennardStone algorithm. The *kenStone* function of the “prospectr” package was used to determine the distribution of the modeling and cross-validation datasets. Different polynomial linear models were then compared, with different numbers and types of explanatory variables as well as different degrees. Model selection was therefore based on maximizing R^2^_adj_, while minimizing BIC and by cross-validating the model on the cross-validation dataset.

Since the basis of the model was linear regression, standardized regression coefficients (SRC) were used as sensitivity index, as classically reported in the literature [44]. The regression coefficients denoted by $\hat{\beta }$ were determined by ordinary least-squares regressions and provided information about the sensitivity of the model response to the various input-factors, and their combinations. SRC is equal to $(\sigma_{Xi}/\sigma_{Y})*\hat{\beta }$, where *σ*_*Xi*_ and *σ*_*Y*_ are the standard deviation of inputs and output variables, respectively. The SRC values were determined using the “sensitivity” package in R [45]. With this approach, the sensitivity of the model to a given variable is high when the absolute value of SRC is high.

## Results and discussion

This study provides an extensive compilation of bacterial richness from the soil environment i) to draw the first map of bacterial richness across France with over 1,700 geo-located samples, ii) to decipher the ecological processes (selection vs dispersal limitations) involved in such distribution and also iii) to elaborate an operational predictive model of bacterial richness according to soil parameters. By applying pyrosequencing technology to soil DNA from all the composite soil samples in the French monitoring network, we were able to generate more than 17,980,000 16S rRNA sequences and to describe 92,571 different OTUs.

### Soil bacterial richness variation and distribution across French national territory

Bacterial richness recovered from the 1,798 RMQS soils, ranged from 555 to 2,007 detected OTUs with an average value of 1,288 (± 207) OTUs and half of the RMQS soils harboured between 1,170 and 1,424 OTUs (S1 Fig). These results are in the same order of magnitude as those classically obtained in different soil environments, using comparable sequencing technology and sequencing depth [46,47]. Such a great variation might result from our extensive sampling strategy, which enabled various types of soil and land uses to be compared. Another consequence of this huge variability of soils and environmental parameters was that the cumulative number of different OTUs detected did not reach the saturation even when all the 1,798 RMQS soils studied were considered (S2 Fig).

**Fig 2:**
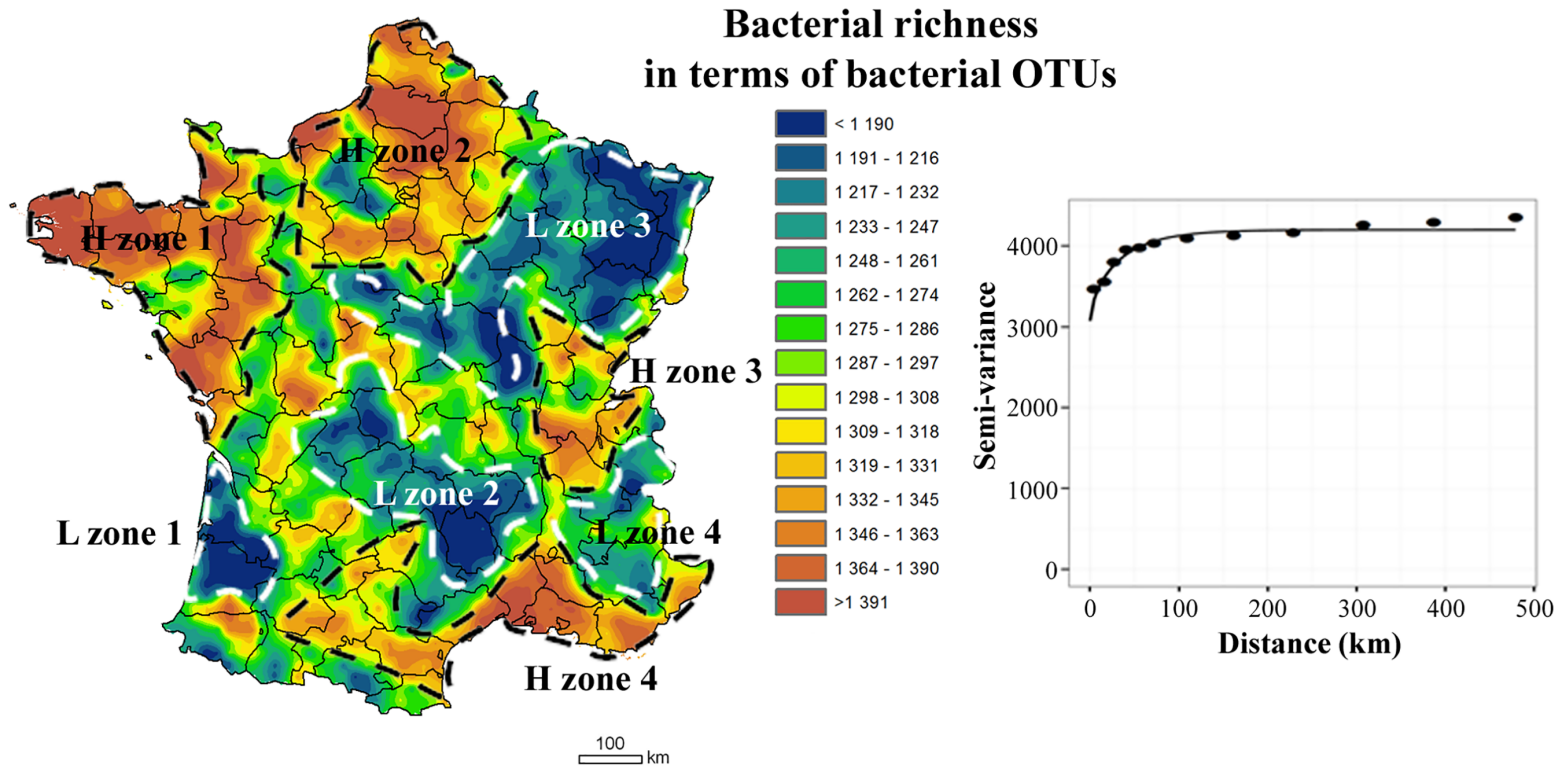
Mapping and robust variograms of soil bacterial richness on the scale of France. The colors indicate the extrapolated values expressed as OTU per soil sample. The **L** and **H** zones visually observed on the map correspond to Low and High bacterial richness zones on a regional scale, respectively. In the graph, points represent the experimental variogram, and continuous lines the Matérn models fitted by maximum likelihood method.

In this study we provide the first national map of soil bacterial richness with its experimental and fitted variogram (Fig 2). The results of the 10-fold cross-validation gave a mean value of the SPPEs that is 1.021 and very close to the expected value, and a median value of 0.3922, both values falling within the 95% confidence interval. As indicated by the parameters of the Matérn function of the variogram, the observed (nugget / (nugget + sill)) ratio was high (= 0.73), suggesting that a large proportion of the variance was unexplained. Despite the rigorous standardisation of our molecular tools from soil DNA extraction to sequencing technology [27], the unexplained variance might be partly due to methodological variability. It might also be due to the large scale of the sampling scheme, which is unsuitable for detecting rough spatial process at small distance as previously suggested [48].

The map obtained revealed a heterogeneous distribution of bacterial richness, which was to a large extent spatially structured in geographical patterns defining more or less wider regions with hot- or cold-spots (Fig 2). The fitted model gave an effective range of 111.6 km revealing a large autocorrelation distance but smaller than those observed for molecular microbial biomass (160km) [23]. This difference confirmed that the abundance and diversity of soil microorganisms are not driven by the same filters as demonstrated at another spatial scale [49].

The scale of spatial variations of bacterial richness did not correspond to the French climatic distribution (Soil Atlas of Europe, climate p. 122) or to the presence of large natural barriers (mountain, sea…; Soil Atlas of Europe, elevation p. 121) [50]. On the other hand, the observed geographical patterns of bacterial diversity could be matched with large pedological patterns. The distribution of French soil types in terms of physico-chemical characteristics (http://gissol.orleans.inra.fr/programme/bdgsf/carte.php) corresponded to certain richness hot- or cold-spots, suggesting that these soil physico-chemical characteristics had a strong influence. For example, the cold-spots of OTUs located in North-East and in South-West (L-zone 1, and a part of L-zone 3, respectively, Fig 2) correspond closely to the most acidic soils in France (http://www.gissol.fr/donnees/cartes). In addition, distribution of bacterial richness patterns could correspond to the coarse level of land cover distribution described for France (Fig 1; http://www.statistiques.developpement-durable.gouv.fr/clc/fichiers/; Soil Atlas of Europe, land cover p. 123) [50]. The low number of bacterial OTUs recorded in Landes, Centre and North-East (L-zones 1, 2 and 3, respectively, Fig 2) could be related to the distribution of particular land covers, notably forest and grasslands, in these regions (Fig 1). In contrast, hot spots of bacterial richness seemed mainly to correspond to regions under crop systems, such as the Brittany, the North and the South around the Mediterranean (H-zones 1, 2 and 4, respectively). These observations imply that the autocorrelation distance might be partly driven by the influence of large patterns of soil types and coarse level of land cover distribution on bacterial diversity.

### Ecological processes driving soil bacterial richness

**Fig 3:**
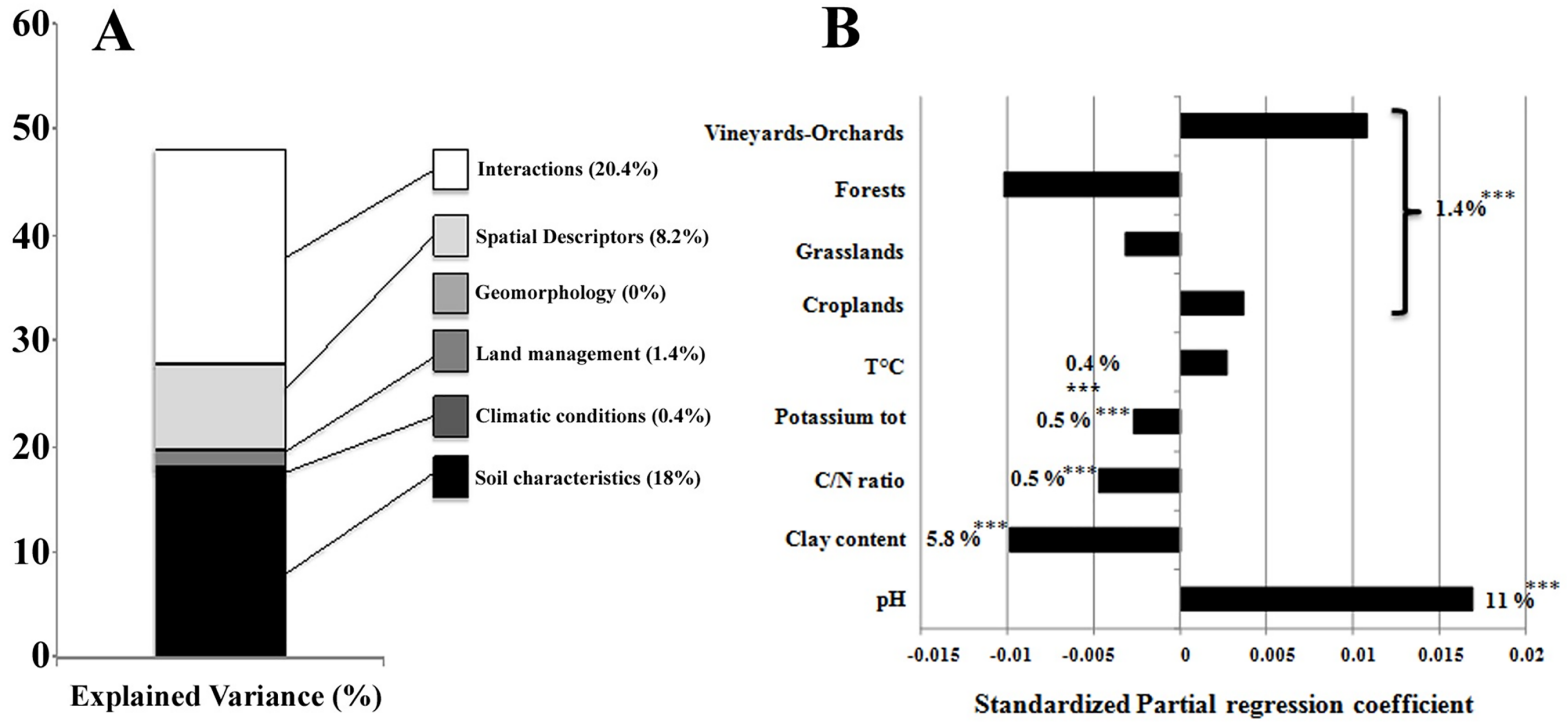
Variance partitioning, contribution and effect of model parameters for the distribution of bacterial richness on the scale of France. (A) Variance partitioning of bacterial richness. The amount of explained variance corresponds to the adjusted R^2^ values of the contextual groups using partial redundancy analysis. The significance level of the contribution of the sets of variables is at least *P* < 0.01. (B) Model parameters for the distribution of bacterial richness on the scale of France. Each parameter is presented with its estimated model coefficients and its marginal effect assessed by a permutation test. *P*<0.01: **, *P*<0.001: ***. Missing values indicate that the variable was not retained in the model. Sand was removed prior to model evaluation since it was represented by the opposite of the sum of silt and clay contents.

Total variance was partitioned between five types of explanatory sets of environmental parameters: soil properties, land management, climate, spatial descriptors and interactions. Soil parameters, land management, climate and their interactions are linked with ecological processes derived from the deterministic theory and based on selection by the environment [51] whereas spatial descriptors can be partly related to variations in unmeasured environmental parameters [52] and/or linked with neutral processes such as dispersal limitation [53]. The variance partitioning approach revealed that the total amount of explained variance of bacterial richness was 48.2%, which is significantly lower than those observed at the landscape scale [49]. This difference might be due to the smaller variation in soil characteristics, climate and geomorphology on a landscape scale than on the scale of France. Variance partitioning indicated a significant influence (*P*<0.01) of soil characteristics (18% of explained variance), spatial descriptors (8.2%), land use (1.4%), climatic conditions (0.4%), but not of geomorphology (Fig 3A). Interactions between soil characteristics, land use and climate represented also a large proportion of the explained variance (20.4%). These observations are congruent with recent studies evidencing the major effect of soil characteristics on bacterial richness, and consequently the high impact of selection processes (due to the influence of specific environmental parameters) shaping bacterial richness [4,46]. On the other hand, the influence of space might be partly related to variations in unmeasured environmental characteristics [52] but also suggests that dispersal limitation may be non negligible in shaping bacterial richness [13,54].

For each filter, within the sets of environmental and spatial descriptors, the marginal effect accounted for relatively small, but significant, proportions of the total variance (from 0.4% to 11%) due to the large number of parameters involved (Fig 3B). Regarding soil characteristics, the pH (11%) and the clay content (5.8%) were the main drivers of bacterial richness, with pH having a positive effect (indicated by a positive sign for the standardized coefficient) conversely to the other parameters (Fig 3B). These results confirmed the overriding effect of pH as a stimulating factor of bacterial community diversity at various spatial scales [15,49,55]. The significant but negative effect of clay content might be partly explained by the decrease in heterogeneity at a microscale with increasing clay content, leading to a lower diversity of microbial habitats and thus to a smaller hosting capacity for various indigenous bacterial species [56]. In addition, the C:N ratio as well as total Potassium content had a weak (0.5%) but significant negative effect on bacterial richness distribution, (Fig 3B). This was consistent with several reports highlighting that soils with a high C:N ratio, corresponding to a high recalcitrance of soil organic matter to degradation by microbes harboured a lower richness of microorganisms [57,58]. The weak (0.4%) but positive influence of climate (temperature in°C) and geomorphology on bacterial richness was in agreement with other reports observing that these distal filters were little involved in microbial abundance and diversity distribution [12,23]. On the other hand, at larger scales, like continental or global scales with wider ranges of parameters (*e*.*g*. range: 2.5 to 25.7°C for mean annual temperature for Zhou et al., (2016)), the temperature can influence microbial diversity distribution (directly, or indirectly by impacting plant cover), or the distribution of specific groups like the *Cyanobacteria* [14]. Altogether, these results modulate the hypothesis that the main filters driving the biodiversity of macro- and micro-organisms are different [24,51,59].

Independently of the other environmental variables, on this scale land use accounted for a small proportion (1.4%) of the explained variance, in agreement with previous reports that bacterial richness is generally poorly impacted by land use [2,60]. Even if both soil properties and land use have discriminating effects on soil bacterial diversity, cross-effects may have occurred, since soil pH and organic carbon content, for example, could also be dependent on land use and especially on agricultural practices (liming and tillage, respectively; [49]). On the other hand, less fertile soils (acidic, sandy) have been historically dedicated to forests [23]. In addition, by comparing the signs and values of the standardized estimated coefficients of land use categories, we demonstrated that bacterial richness was negatively impacted by natural or semi natural land uses *i*.*e*. forests and grasslands, and positively by perennial (vineyards-orchards) or annual crop systems (Fig 3B). This observation supported a positive relationship between bacterial richness and soil disturbance due to cropping intensity, with Vineyards-Orchards > Crops > Grasslands > Forests, combining different types of agricultural practices (tillage, crop protection, fertilization, crop rotation; [61]). According to the “humped-back” model describing the response of the diversity of a community to environmental stress [62], a decrease in apparent diversity may occur (i) in a highly stressed environment due to dominance of particularly competitive species through selection, and (ii) in a notably unstressed environment, due to the dominance of particularly adapted species through competitive exclusion [63]. Contrastingly, moderate stress may increase apparent diversity, due to a diminution in competitive niche exclusion and in selection mechanisms. Our results showed that soils under annual (croplands) or perennial crop systems (vineyards/orchards) would correspond to these conditions, as they harboured highest richness levels compared to forests and grasslands (considered as unstressed environments) [3,61].

The spatial descriptors of the studied area, illustrating neighbourhood relationships between samples, corresponded to 26 significant (Principal Coordinates of a Neighbour Matrix), each representing different spatial scales (coarse: 110 to 250km, medium: 60 to 110km and fine: 30 to 60km, Table 2). The whole variance of bacterial richness explained by spatial descriptors was 8.2% and ranged from 0.19% to 0.74% according to PCNM. This scale dependency may reflect the effect of unmeasured spatial gradients [52], but may also be related to dispersal limitation of bacterial communities in regards of the large number of explanatory variables introduced in the analysis [54,64]. The influence of the scale was ranked by comparing the signs of the standardized coefficients and by cumulating the explained variance for each scale. A larger number of PCNMs (17) describing the fine scale were involved in explaining bacterial richness, with 5.25% of the cumulated explained variance, whereas those representing the coarse and medium scales were fewer (2 and 7 PCNMs with 1.02% and 1.89% of the cumulated explained variance, respectively). At fine and medium scales, the influence of spatial descriptors might be partly related to variations in unmeasured soil characteristics and land use, whereas at coarse scale it might results from geomorphology or the distribution of overall land cover (forest, grassland, mountains, sea). Our observation suggests that landscape configuration would be a significant driver of soil bacterial richness as also demonstrated on biodiversity turnover [24]. In addition, our analysis revealed numerous negative effects of spatial descriptors on bacterial richness at medium and fine scales, thus confirming that landscape configuration would be a significant driver and might partly affect bacterial richness by limiting bacterial dispersal as also demonstrated previously [24]. Altogether, our results showed that biogeographical patterns of bacterial richness can be explained by both selection (*i*.*e*. environmental filters like pH or C:N) and neutral processes (*i*.*e*. dispersal limitation), each being non-exclusive.

**Table 2:** Model parameters of spatial descriptors for the distribution of bacterial richness on the scale of France. Considering that the major part of the environmental selection was measured by the previous explanatory variables (soil properties, land-use, etc.), we investigated the effect of dispersal on the residuals of the variance partitioning models. To do that, the neighbourhood between sites at various classes of distance was evaluated, using a Principal Coordinates of Neighbour Matrix approach (PCNM). Each spatial descriptor is presented with its estimated model coefficients and its marginal effect assessed by a permutation test (*P*<0.05). Missing values indicate that the variable was not retained in the model. Spatial components were summarized according to the spatial scale considered: coarse, medium or fine.

Scale	Spatial descriptors (PCNM)	Explained variance (%)	Model coefficient
Coarse [110km; 250km]	PCNM_13_	0.28	0.19
	PCNM_29_	0.74	-0.29
Medium [60km; 110km]	PCNM_58_	0.30	0.19
	PCNM_75_	0.28	-0.19
	PCNM_113_	0.25	-0.18
	PCNM_120_	0.23	-0.17
	PCNM_126_	0.38	0.21
	PCNM_128_	0.19	0.16
	PCNM_142_	0.26	0.18
Fine [30km; 60km]	PCNM_188_	0.47	-0.23
	PCNM_211_	0.25	0.18
	PCNM_216_	0.29	-0.19
	PCNM_264_	0.56	-0.25
	PCNM_275_	0.33	-0.20
	PCNM_281_	0.19	0.16
	PCNM_296_	0.22	-0.17
	PCNM_305_	0.26	-0.18
	PCNM_316_	0.27	-0.18
	PCNM_319_	0.43	-0.23
	PCNM_327_	0.46	-0.23
	PCNM_359_	0.23	-0.17
	PCNM_387_	0.20	0.16
	PCNM_426_	0.19	-0.16
	PCNM_427_	0.26	0.18
	PCNM_436_	0.35	-0.21
	PCNM_466_	0.29	0.19

### Predictive model of soil bacterial richness

**Fig 4:**
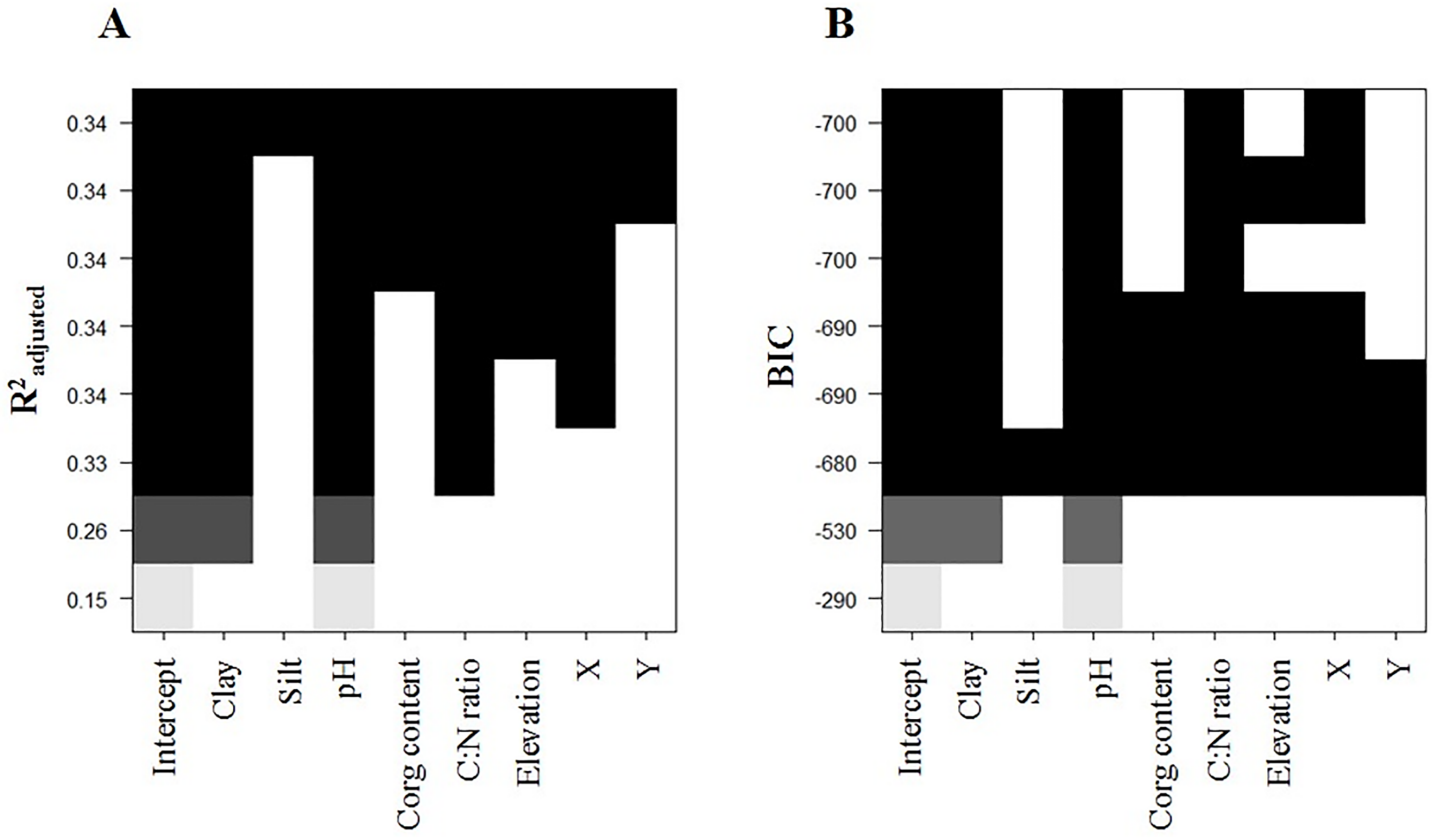
Hierarchy of the linear models of soil bacterial richness involving soil physicochemical, geographical coordinates and climatic variables. The hierarchy of the linear models implying environmental variables is given according to the R^2^_adj_ criterion (A) and the BIC criterion (B) with the exhaustive method. Each row in this graph represents a specific model. The variables included in a given model are represented by means of shaded rectangles. The intensity of the shading represents the ordering of the BIC and R^2^_adj_ values according to the absolute value.

Based on the RMQS dataset of bacterial richness and environmental parameters we have developed a predictive statistical model to provide a reference value of bacterial richness for a given pedoclimatic condition. The linear models with the smallest BIC (-700) and the largest R^2^_adj_ (0.34) highlighted eight environmental parameters as significant explanatory variables of the bacterial richness, which were ranked as follows: pH > Clay content > C:N ratio > X (longitude) > elevation > C_org_ content > Y (latitude) > Silt content (Fig 4). This observation confirmed and refined the hierarchy of the environmental filters obtained with variance partitioning (Fig 3). At this step, climate data are not retained despite their significant role, since they are rarely available and expensive to obtain, limiting the use of this model to compare predicted and measured values in a context of soil biological diagnosis for and by soil users [17]. Alternatively, climatic conditions were replaced in the model by longitude, which in France is integrative of climate and soil moisture and did not reduce the R^2^ of the model and therefore its robustness [25].

**Fig 5:**
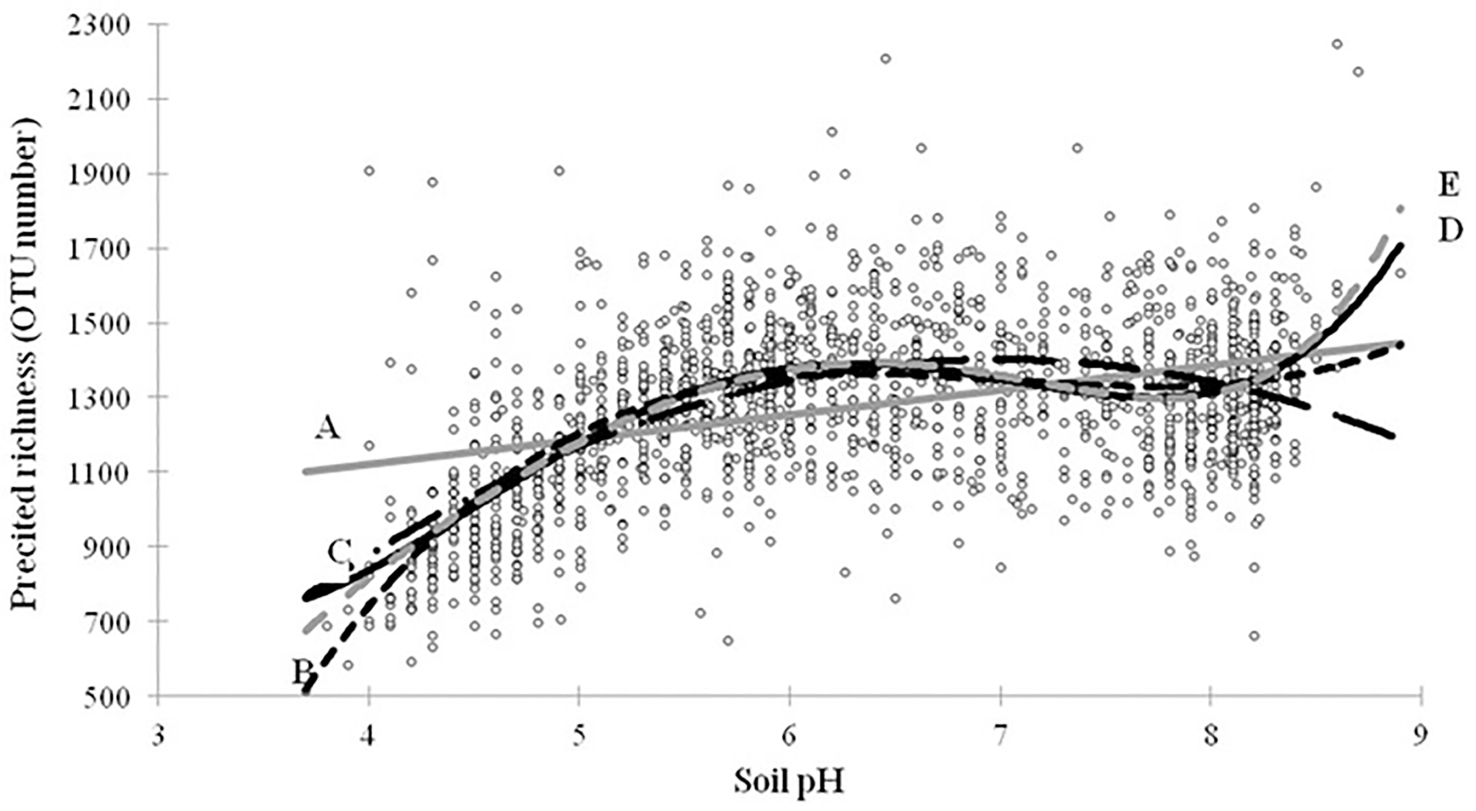
Polynomial regression between the bacterial richness and soil pH for different level of increasing complexity (from simple linear and up to the fifth degree) of the model. (A) Grey line, simple linear model, bacterial richness = 66.48*pH + 855.43; R^2^ = 0.15. (**B**) Dotted black curve, quadratic model, bacterial richness = -58.55*pH^2^ + 818.8*pH—1460.6; R^2^ = 0.29. (**C**) Dotted black curve, cubic model, bacterial richness = 25.458*pH^3^–537.9*pH^2^ + 3751.5*pH—7287.4; R^2^ = 0.32. (**D**) Black line, model degree four, bacterial richness = 13.06*pH4–302.64*pH^3^ + 2499.2*pH2–8510.8*pH + 10919; R^2^ = 0.34. (**E**) Dotted grey curve, model degree five, bacterial richness = 2.63*pH5–69.59*pH^4^ + 720.07*pH3–3731.7*pH^2^ + 10171pH—11127; R^2^ = 0.34.

First, we developed a linear polynomial regression model based only on pH as explanatory variable, as previously described [12]. By testing the increasing complexity (from simple linear and up to the fifth degree) of the polynomial model, we found that the model degree four gave the best R^2^ (0.34) since at higher degree the R^2^ remained virtually unchanged (Fig 5). Nevertheless, in their study based on 100 soils, Fierer & Jackson, 2006 obtained a higher R^2^ (0.58) with a degree 2. This difference might be partly explained by our deeper sampling effort, but mostly by the genotyping technics used by these authors (T-RFLP), which could limit the variability of the estimated richness (several tens of populations) by comparison with pyrosequencing technology (several tens of thousands of OTU in our case), as previously shown [4]. To improve the model in terms of higher R^2^_adj_ and to get closer to the normality hypotheses and to improve the variance homogeneity of the residuals [65], we tested for its ability to include interactions between explanatory variables identified [17,65,66]. In addition to pH, we selected only three main explanatory variables to be included in the model: Clay content, C:N ratio and longitude (X), since the R^2^ of the model based only on pH was not significantly improved by integrating additional variables (data not shown). Finally, the model developed has a R^2^ = 0.56 and a R^2^_adj_ = 0.58 and the following mathematical form (all parameters of the models are given in the S2 Table):

$$Richness=1044+3.305*pH^{4}-0.0457*Clay^{2}+0.0597+0.00298*Clay^{2}*C:N-1.54*10^{-6}*Clay^{3}*C:N+2.336*10^{-5}*{\left(C:N\right)}^{2}*X.$$

To validate the model, we evidenced the normality distribution of model residuals, which was confirmed by the Shapiro-Wilk test of normality (*P* = 0.149), as well as a good homogeneity of the residuals tested by the Breush-Pagan test of homogeneity (*P* = 0.5469). In addition, plotting the measured richness against the predicted richness using the cross-validation dataset revealed an important scatter of the points around the y = x line (S3 Fig), which validated the high predictive ability of the developed polynomial model. Finally, the sensitivity of the model to measurement errors on each explanatory variable was evaluated by a sensitivity index [17]. This analysis demonstrated that the model was highly sensitive to variations in pH, clay content and C:N ratio, together with their interactions and cubic effects, which was in agreement with the above discussion concerning the variance partitioning analysis (S3 Table). However, one limitation of our model is the absence of variations in bacterial richness between different years (or between seasons during one year) that can result from modifications of climatic conditions, plant cover or agricultural practices. In complement to our study, it will be relevant to validate the robustness of this model on a sampling strategy that integrates such temporal variation.

Altogether, our study provides the first French national atlas of soil bacterial richness using an extensive sampling survey of about 1,800 samples, and confirms the relevance of investigating microbial community on a nationwide scale to better understand the ecological processes involved in regulating microbial richness. We showed that the distribution of bacterial richness at this scale was heterogeneous and spatially structured, mainly driven by proximal filters such as soil characteristics and land use (both supporting a selection process) but also significantly influenced by spatial descriptors (potentially supporting dispersal limitation in microbial populations, derived from neutral theory, or the influence of unmeasured soil properties). This nationwide spatial scale was also shown to be relevant for evaluating overall land use in the context of a sustainable use of soil resources. Based on the referential dataset, the predictive model developed in this study complements the one developed for molecular microbial biomass [17], as both present innovative and operational mathematical tools for assessing a comprehensive soil microbiological status in the French pedoclimatic context. Comparison of predicted and measured values provides a robust diagnosis of soil microbial abundance and diversity and their evolution under environmental pressures such as agricultural practices, industrial pollutions or more global changes. Altogether, mapping and a predictive model of bacterial richness involving over 1,700 geo-located samples covering the French territory could help policy makers to produce conservation policies based on soil biodiversity. Based on this primary analysis of bacterial richness other aspects of soil bacterial beta-diversity such as evenness, community structure, taxa-area relationships and variations in the core bacterial taxa across France need to be investigated to have a comprehensive overview of the biogeography of microorganisms.

## Supporting information

S1 Fig Distribution of detected bacterial OTU numbers in French soils. The curves correspond to simulation of normal distributions (dotted line with estimated parameters: average: 1288.53 ± 207.39 for OTU number) and log normal distributions (black line with estimated parameters: average: 7.1471 ± 0.1719 for OTU number). Normal and log normal distributions were obtained using Maximum Likelihood estimations.
(PDF)

S2 Fig Cumulative curve of different detected OTUs according to the number of studied soils. The thickness of the curve represents the standard deviation obtained from 1,000 cumulative curves with a random selection of soils.
(PDF)

S3 Fig Relationship between the measured and the predicted values of bacterial richness by applying the polynomial model of degree four on the cross validation dataset (180 soil samples). The black line represents the 1:1 line (y = x).
(PDF)

S1 Table Bioinformatic parameters and databases used in the analysis of 16S rRNA gene sequences. (DOCX)

S2 TableSummary of model coefficients and significance.This table describes the Coefficients $\hat{\beta }$ of the fourth degree polynomial model for each of its components. The standard error of each coefficient and its significance is also provided (*P* < 0.05).(DOCX)

S3 Table Overview of the model sensitivity analysis. The Standardized Regression Coefficients (SRC) of the variables to which the model is most sensitive are presented here. The variables are organized according to the absolute value of their associated SRC from the highest to the lowest.
(DOCX)

S1 File Influence of the normalization step on RMQS sample representativeness. 100 replicates of the normalization step were done on each of the 200 randomly selected samples (10% of the samples). For each replicate, high-quality-reads were clustered, and obtained OTUs analyzed to determine the impact of the normalization step on OTUs. Four groups of OTUs were considered: Major (composed of more than 1% of reads), Medium (1–0.1% of reads), Low (0.01–0.1% of reads) and Rare (less than 0.01% of reads), showing no impact of the normalization step.
(ZIP)

S2 File Rarefaction curves of RMQS samples computed after normalization step. (ZIP)
