# Correction: Mapping and predictive variations of soil bacterial richness across France

**DOI:** 10.1371/journal.pone.0190128

**Published:** 2017-12-18

**Authors:** Sébastien Terrat, Walid Horrigue, Samuel Dequiedt, Nicolas P. A. Saby, Mélanie Lelièvre, Virginie Nowak, Julie Tripied, Tiffanie Régnier, Claudy Jolivet, Dominique Arrouays, Patrick Wincker, Corinne Cruaud, Battle Karimi, Antonio Bispo, Pierre Alain Maron, Nicolas Chemidlin Prévost-Bouré, Lionel Ranjard

The third author’s name is spelled incorrectly. The correct name is Samuel Dequiedt. The correct citation is: Terrat S, Horrigue W, Dequiedt S, Saby NPA, Lelièvre M, Nowak V, et al. (2017) Mapping and predictive variations of soil bacterial richness across France. PLoS ONE 12(10): e0186766. https://doi.org/10.1371/journal.pone.0186766
