# Correction: Mapping and predictive variations of soil bacterial richness across France

**DOI:** 10.1371/journal.pone.0268101

**Published:** 2022-05-02

**Authors:** Sébastien Terrat, Walid Horrigue, Samuel Dequiedt, Nicolas P. A. Saby, Mélanie Lelièvre, Virginie Nowak, Julie Tripied, Tiffanie Régnier, Claudy Jolivet, Dominique Arrouays, Patrick Wincker, Corinne Cruaud, Battle Karimi, Antonio Bispo, Pierre Alain Maron, Nicolas Chemidlin Prévost-Bouré, Lionel Ranjard

The Data Availability statement for this paper is incorrect. The correct statement is: All raw data sets are publicly available in the EBI database system (in the Short Read Archive) under project accession no PRJEB21351. The complete soil characteristics are publicly available in the Data INRAe deposit under project DOI: https://doi.org/10.15454/J9H4BS. The dataset published contains all the raw data used in the statistical analysis (Table 1) in order to make them available for any further study. The table contains soil properties, observations on land use, and theoretical coordinates. Real coordinates cannot be made publicly available because they are considered personal data by GDPR and French law–only anonymized data can be shared publicly. The anonymization technique used here is to give theoretical coordinates corresponding to the centre of the 16 km by 16 km grid cell used for the sampling strategy of the RMQS network.
