# Supplementary figures and images for: Mapping and predictive variations of soil bacterial richness across France (part 1 of 2)
Source: PLoS One. 2017 Oct 23;12(10):e0186766. doi: 10.1371/journal.pone.0186766 (PMC5653302; doi:10.1371/journal.pone.0186766)

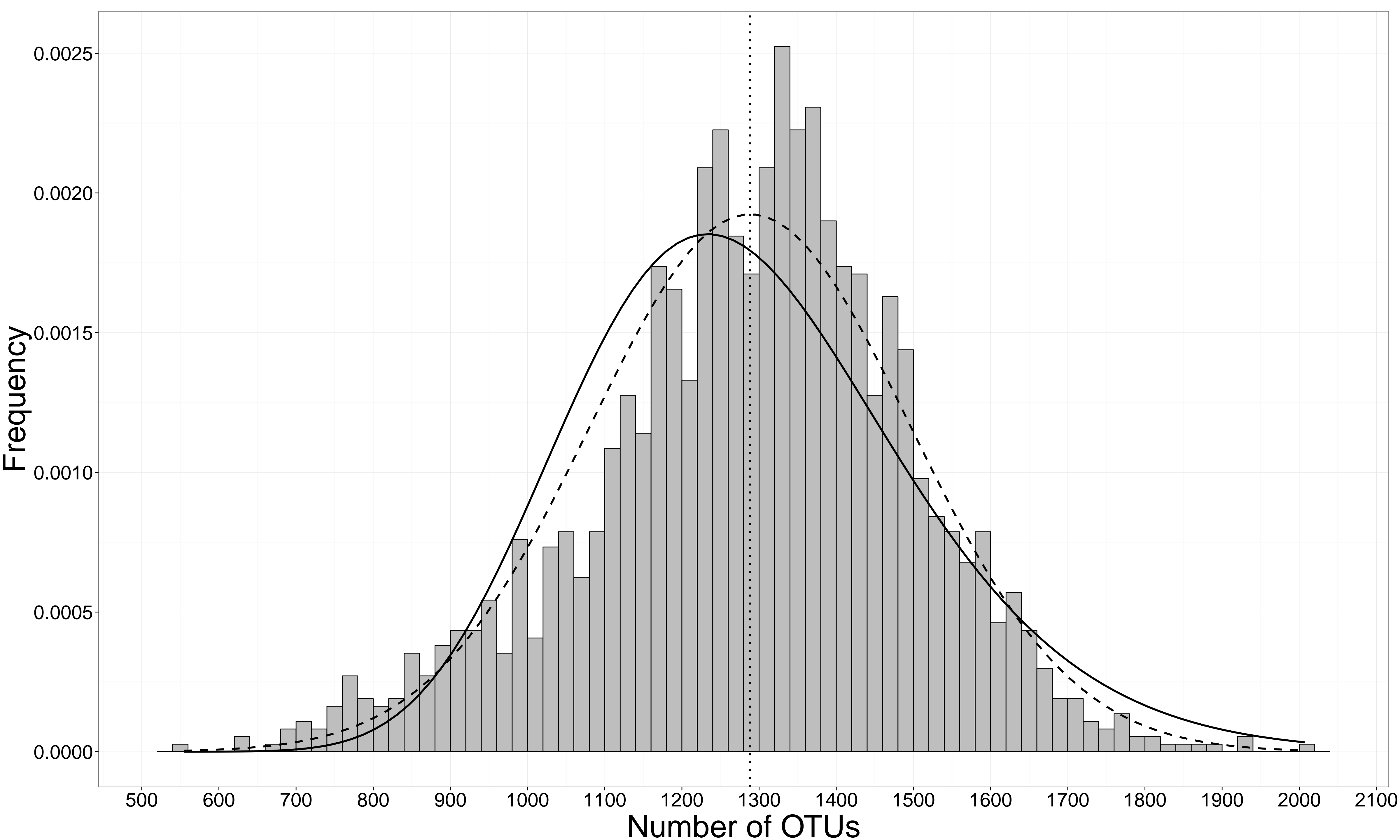

Supplement: S1 Fig — The curves correspond to simulation of normal distributions (dotted line with estimated parameters: average: 1288.53 ± 207.39 for OTU number) and log normal distributions (black line with estimated parameters: average: 7.1471 ± 0.1719 for OTU number). Normal and log normal distributions were obtained using Maximum Likelihood estimations. (PDF) [file pone.0186766.s001.pdf]

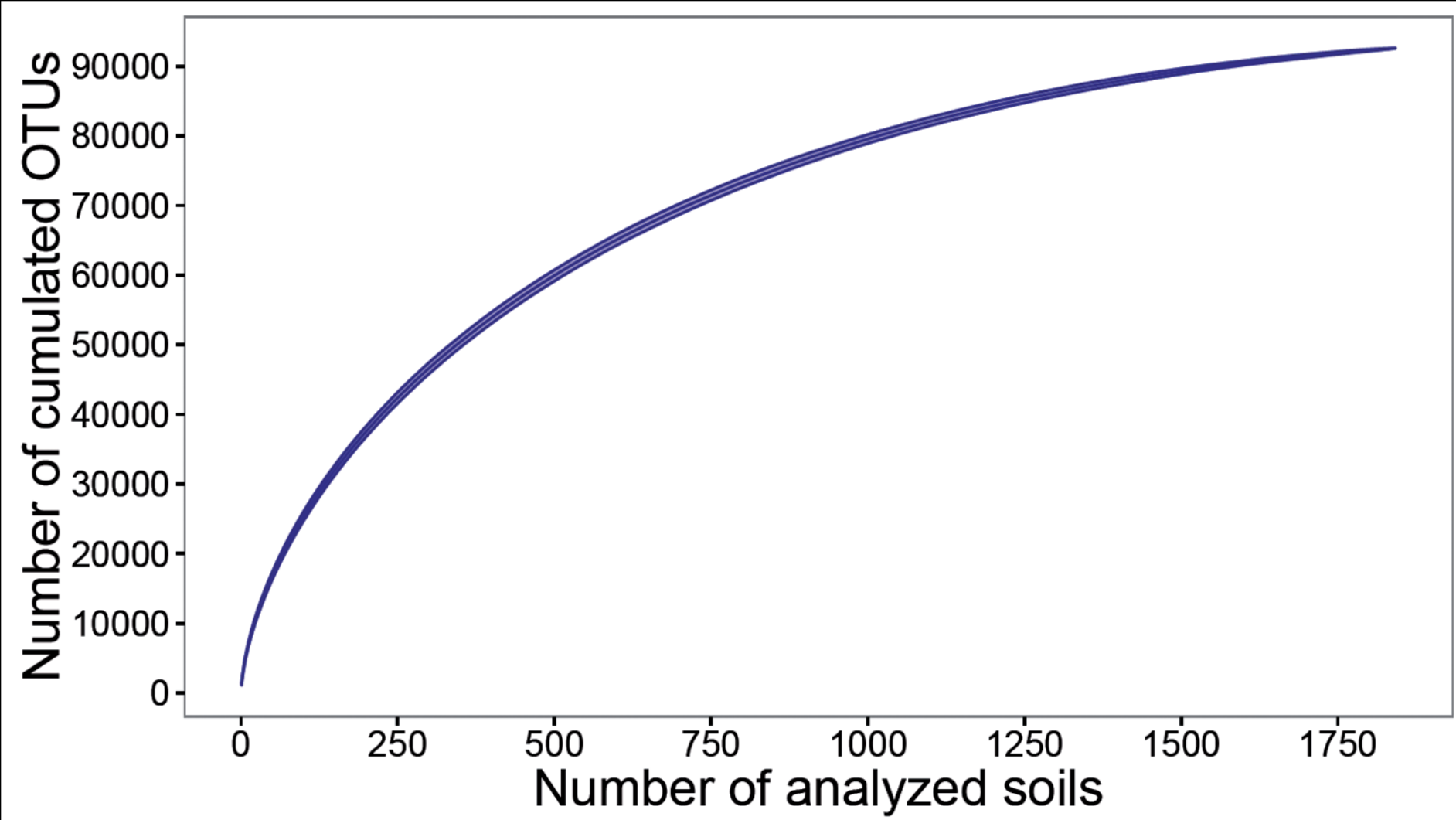

Supplement: S2 Fig — The thickness of the curve represents the standard deviation obtained from 1,000 cumulative curves with a random selection of soils. (PDF) [file pone.0186766.s002.pdf]

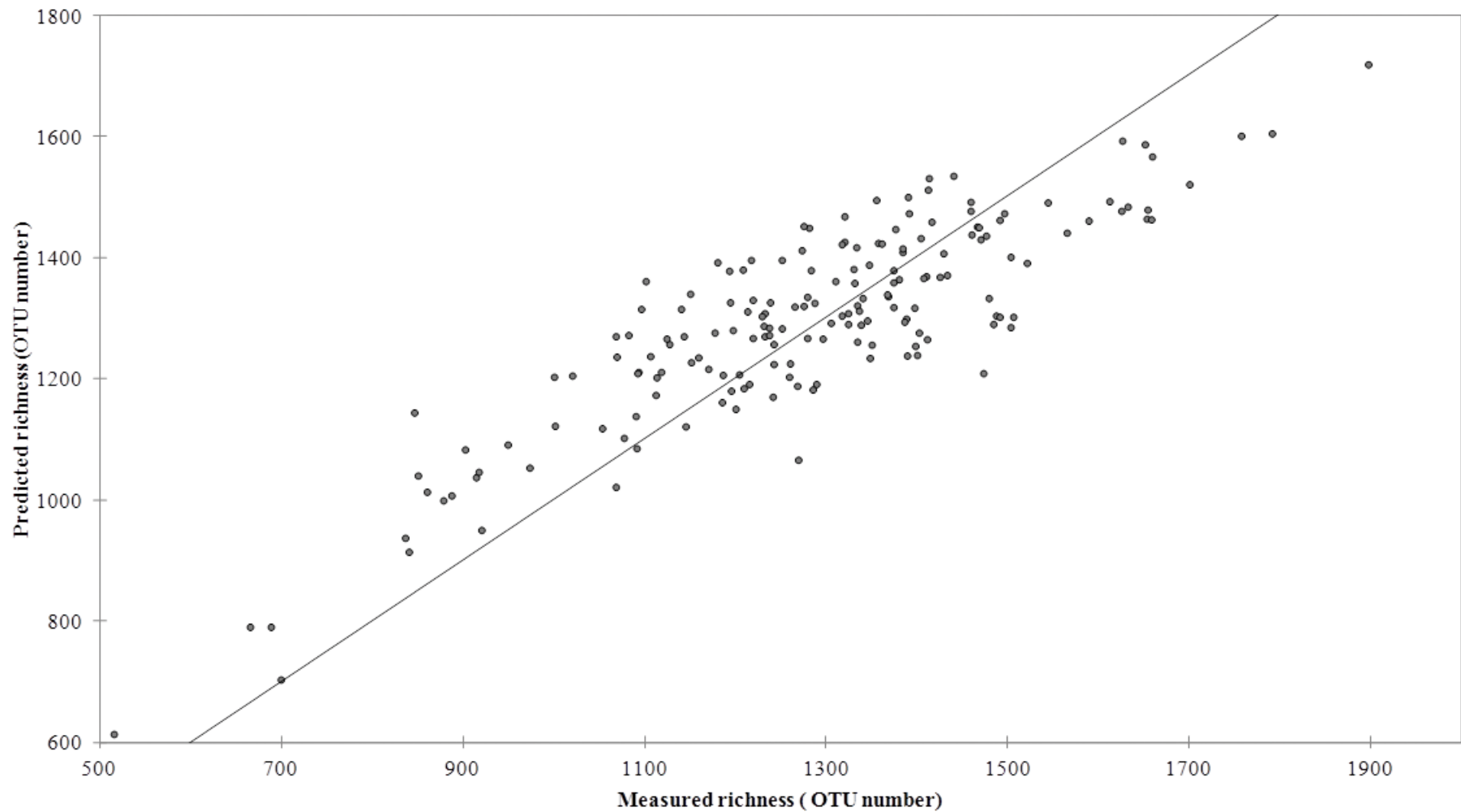

Supplement: S3 Fig — The black line represents the 1:1 line (y = x). (PDF) [file pone.0186766.s003.pdf]

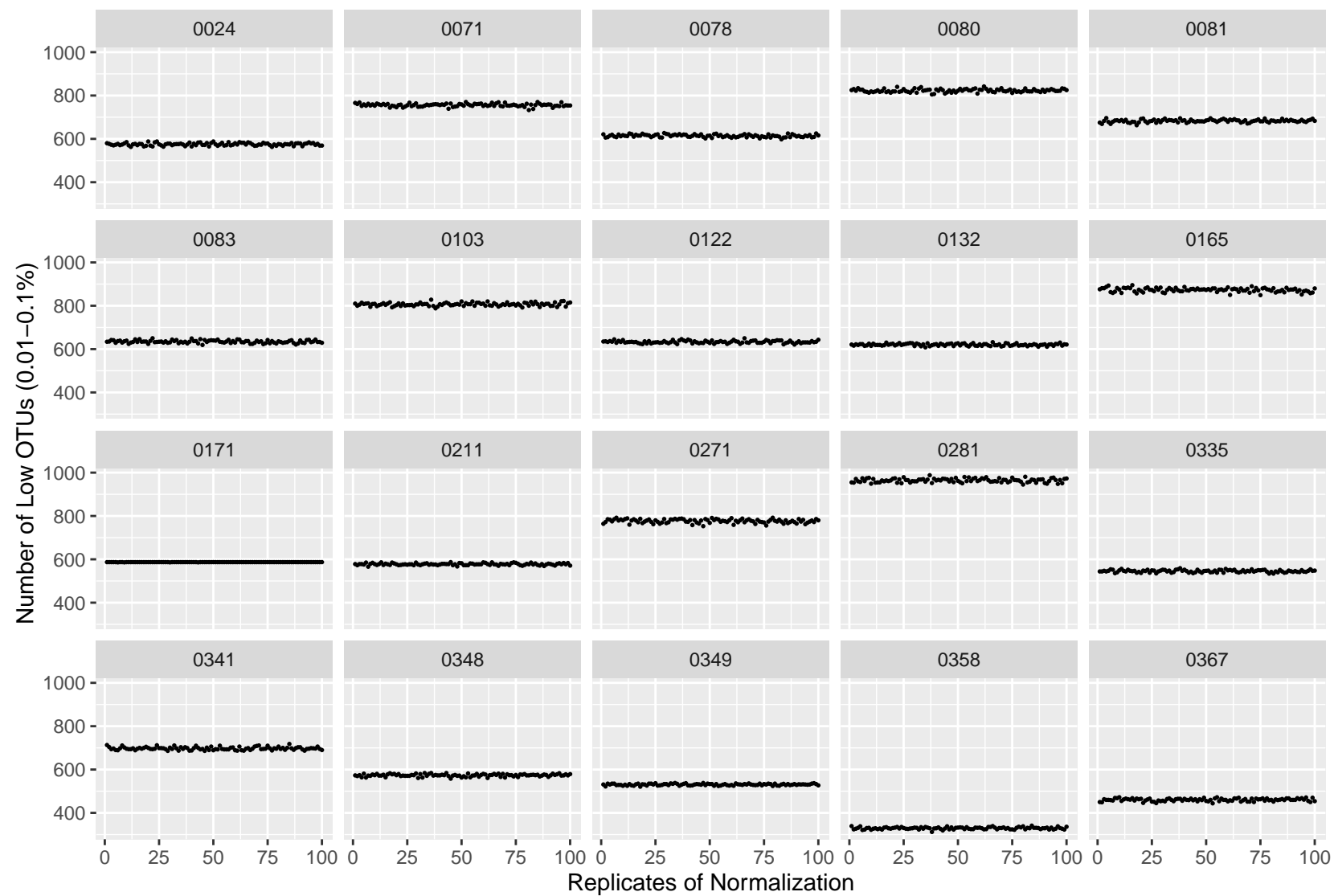

Supplement: S1 File — 100 replicates of the normalization step were done on each of the 200 randomly selected samples (10% of the samples). For each replicate, high-quality-reads were clustered, and obtained OTUs analyzed to determine the impact of the normalization step on OTUs. Four groups of OTUs were considered: Major (composed of more than 1% of reads), Medium (1–0.1% of reads), Low (0.01–0.1% of reads) and Rare (less than 0.01% of reads), showing no impact of the normalization step. (ZIP) [file pone.0186766.s007.zip › Low_Img_1.pdf]

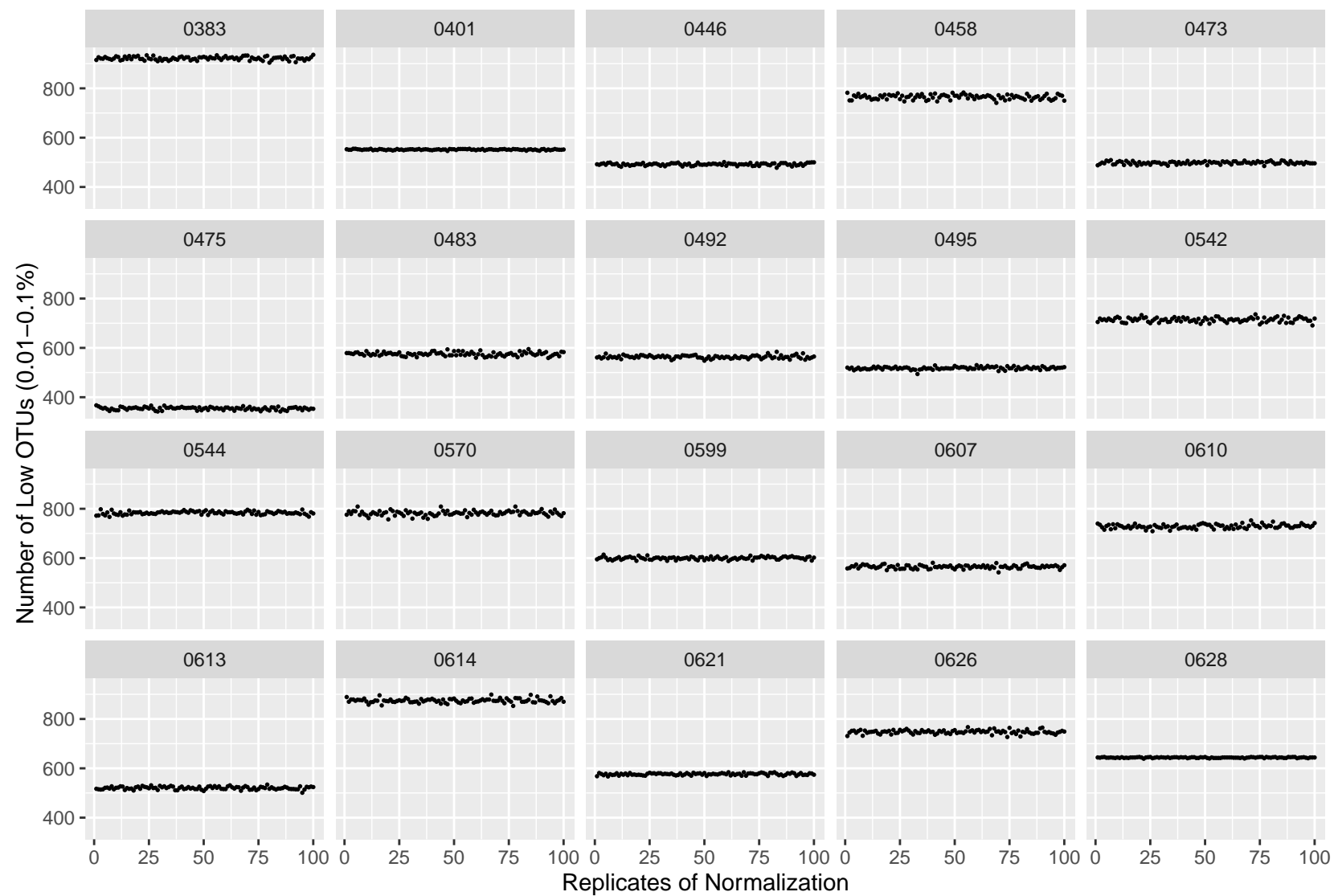

Supplement: S1 File — 100 replicates of the normalization step were done on each of the 200 randomly selected samples (10% of the samples). For each replicate, high-quality-reads were clustered, and obtained OTUs analyzed to determine the impact of the normalization step on OTUs. Four groups of OTUs were considered: Major (composed of more than 1% of reads), Medium (1–0.1% of reads), Low (0.01–0.1% of reads) and Rare (less than 0.01% of reads), showing no impact of the normalization step. (ZIP) [file pone.0186766.s007.zip › Low_Img_2.pdf]

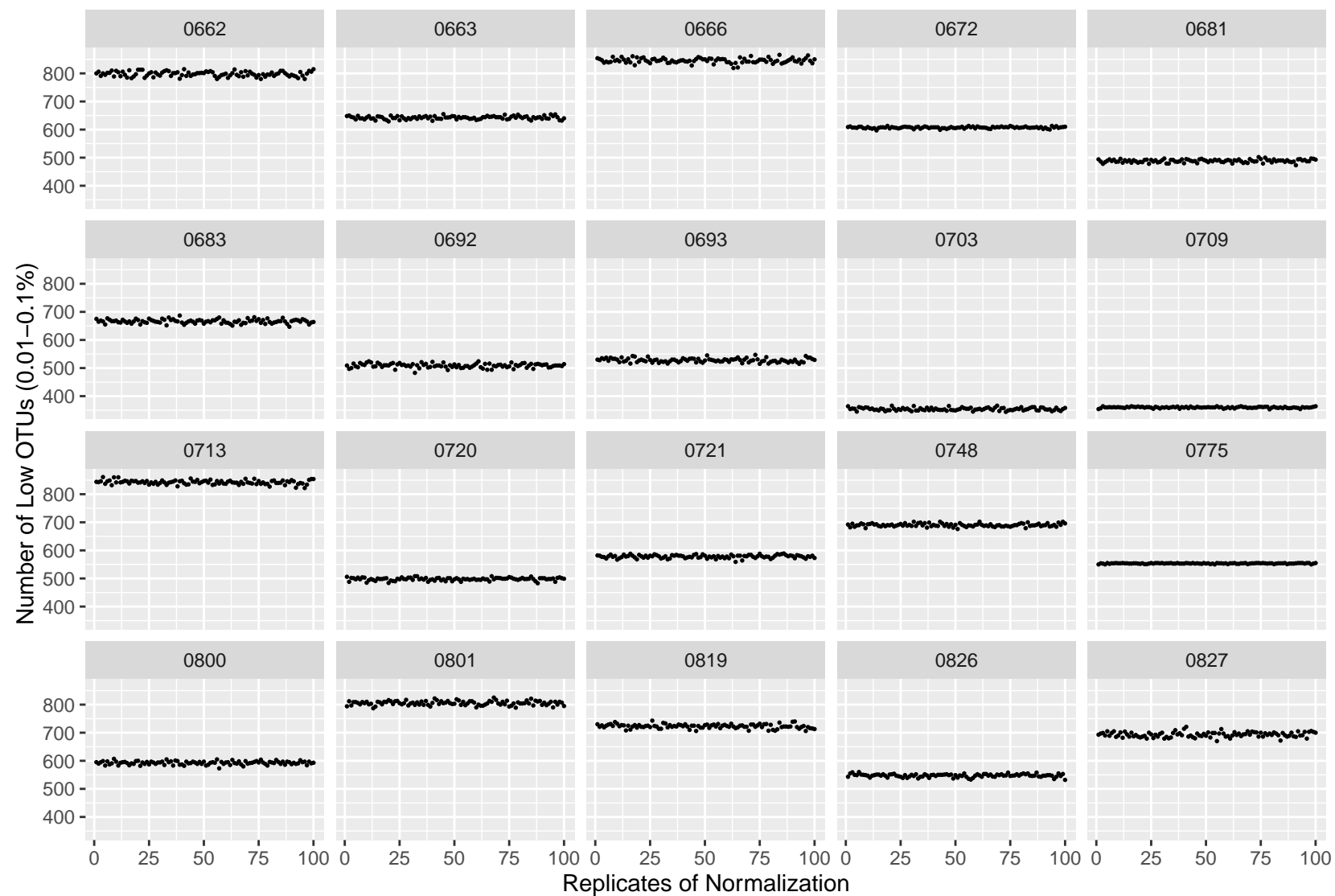

Supplement: S1 File — 100 replicates of the normalization step were done on each of the 200 randomly selected samples (10% of the samples). For each replicate, high-quality-reads were clustered, and obtained OTUs analyzed to determine the impact of the normalization step on OTUs. Four groups of OTUs were considered: Major (composed of more than 1% of reads), Medium (1–0.1% of reads), Low (0.01–0.1% of reads) and Rare (less than 0.01% of reads), showing no impact of the normalization step. (ZIP) [file pone.0186766.s007.zip › Low_Img_3.pdf]

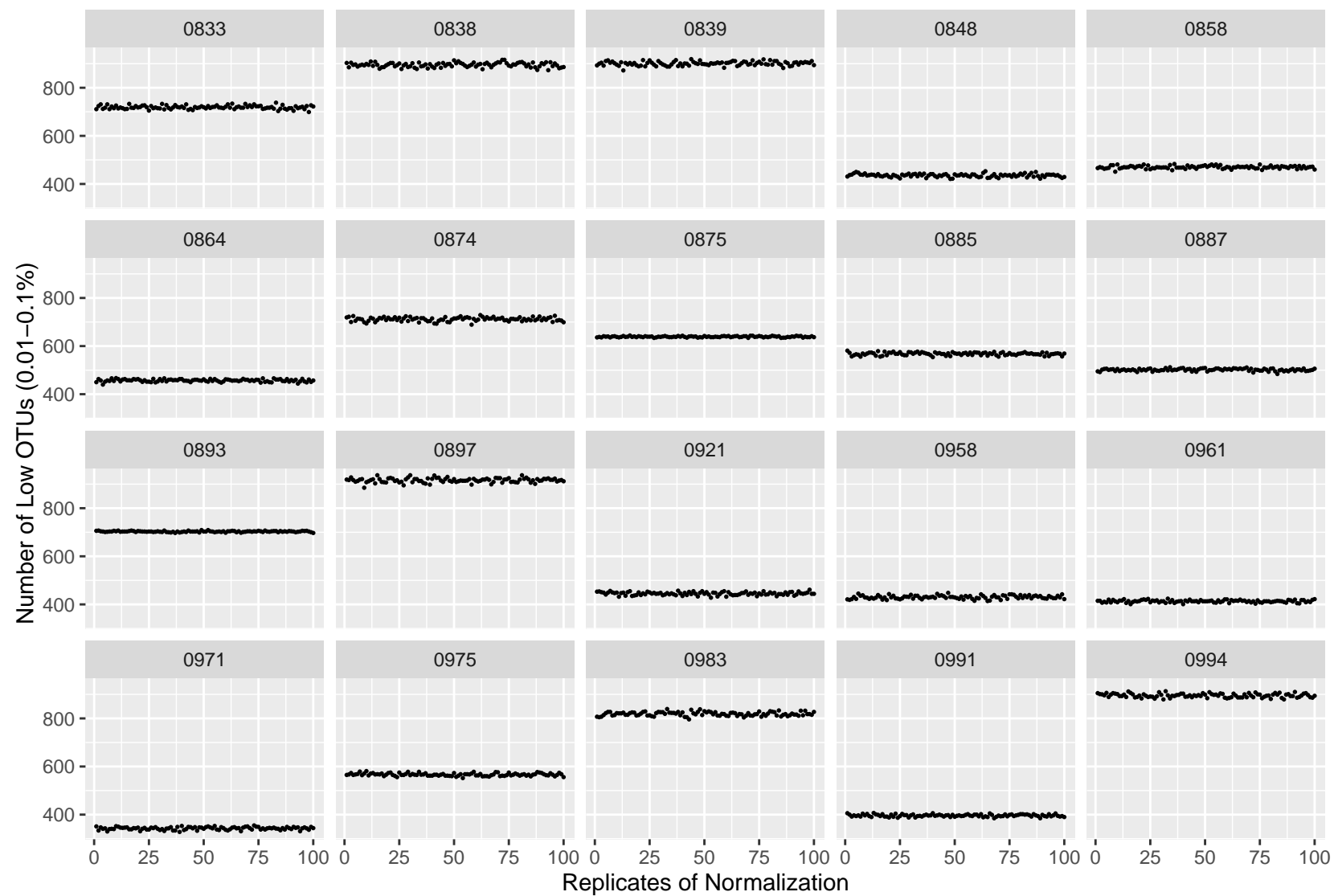

Supplement: S1 File — 100 replicates of the normalization step were done on each of the 200 randomly selected samples (10% of the samples). For each replicate, high-quality-reads were clustered, and obtained OTUs analyzed to determine the impact of the normalization step on OTUs. Four groups of OTUs were considered: Major (composed of more than 1% of reads), Medium (1–0.1% of reads), Low (0.01–0.1% of reads) and Rare (less than 0.01% of reads), showing no impact of the normalization step. (ZIP) [file pone.0186766.s007.zip › Low_Img_4.pdf]

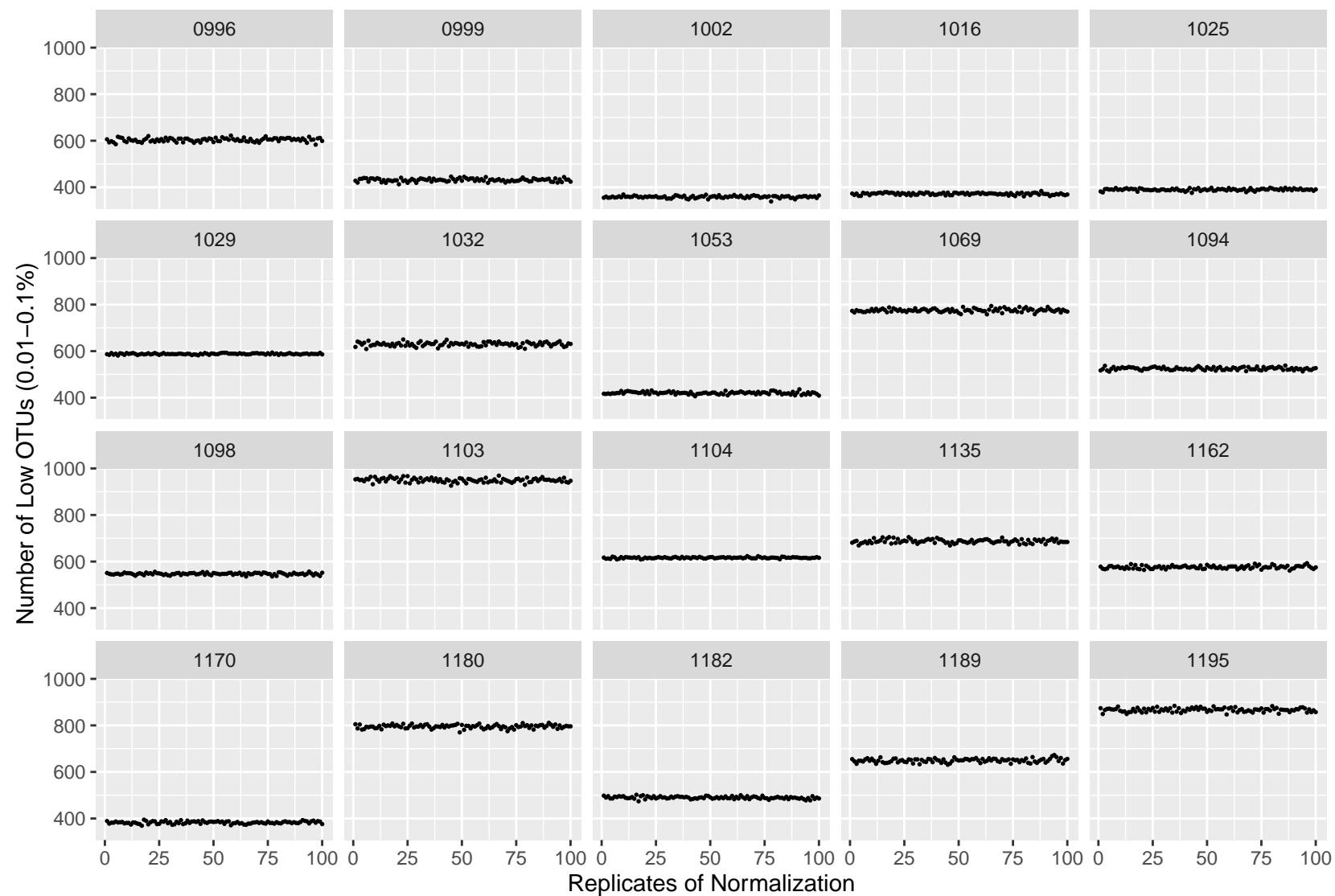

Supplement: S1 File — 100 replicates of the normalization step were done on each of the 200 randomly selected samples (10% of the samples). For each replicate, high-quality-reads were clustered, and obtained OTUs analyzed to determine the impact of the normalization step on OTUs. Four groups of OTUs were considered: Major (composed of more than 1% of reads), Medium (1–0.1% of reads), Low (0.01–0.1% of reads) and Rare (less than 0.01% of reads), showing no impact of the normalization step. (ZIP) [file pone.0186766.s007.zip › Low_Img_5.pdf]

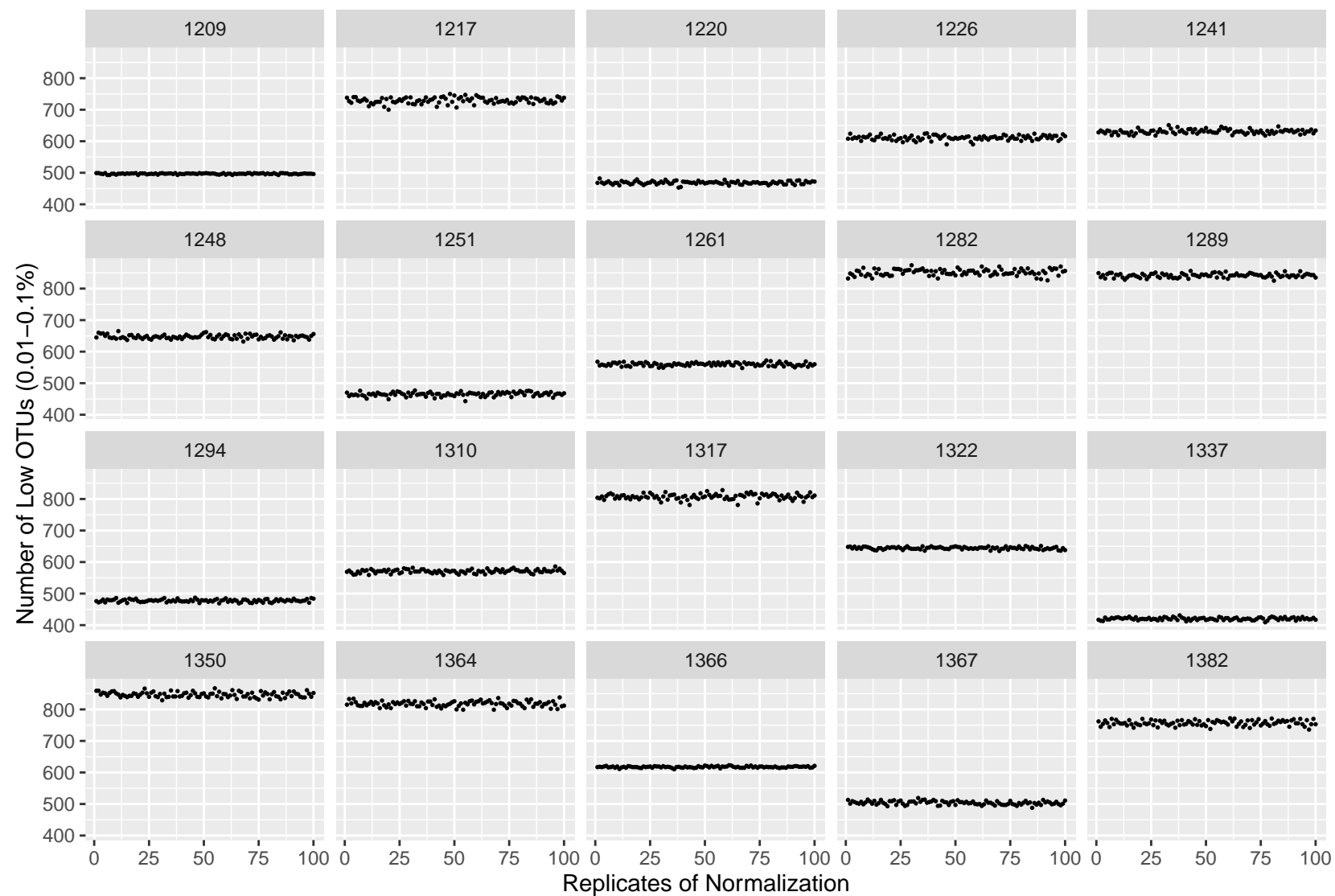

Supplement: S1 File — 100 replicates of the normalization step were done on each of the 200 randomly selected samples (10% of the samples). For each replicate, high-quality-reads were clustered, and obtained OTUs analyzed to determine the impact of the normalization step on OTUs. Four groups of OTUs were considered: Major (composed of more than 1% of reads), Medium (1–0.1% of reads), Low (0.01–0.1% of reads) and Rare (less than 0.01% of reads), showing no impact of the normalization step. (ZIP) [file pone.0186766.s007.zip › Low_Img_6.pdf]

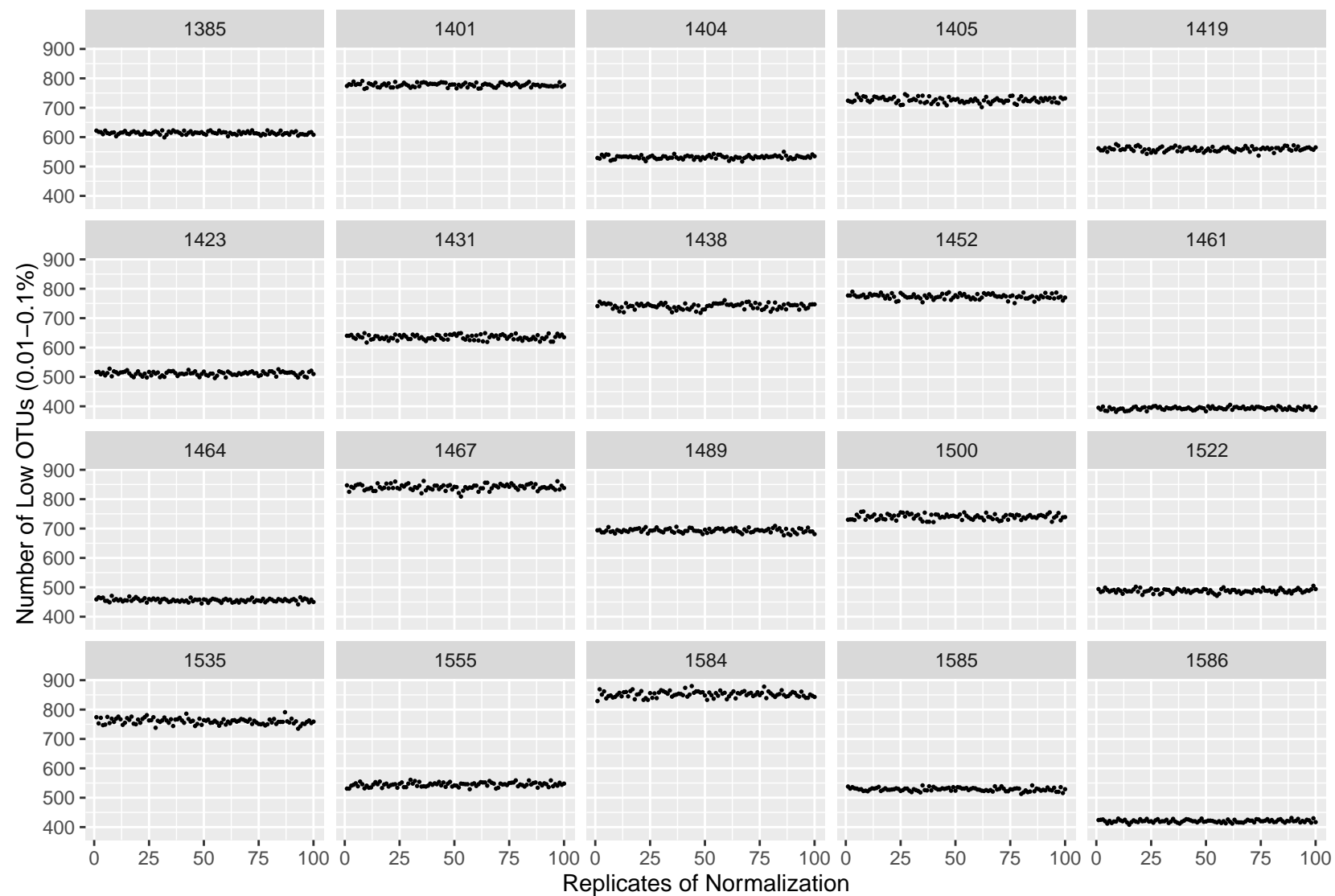

Supplement: S1 File — 100 replicates of the normalization step were done on each of the 200 randomly selected samples (10% of the samples). For each replicate, high-quality-reads were clustered, and obtained OTUs analyzed to determine the impact of the normalization step on OTUs. Four groups of OTUs were considered: Major (composed of more than 1% of reads), Medium (1–0.1% of reads), Low (0.01–0.1% of reads) and Rare (less than 0.01% of reads), showing no impact of the normalization step. (ZIP) [file pone.0186766.s007.zip › Low_Img_7.pdf]

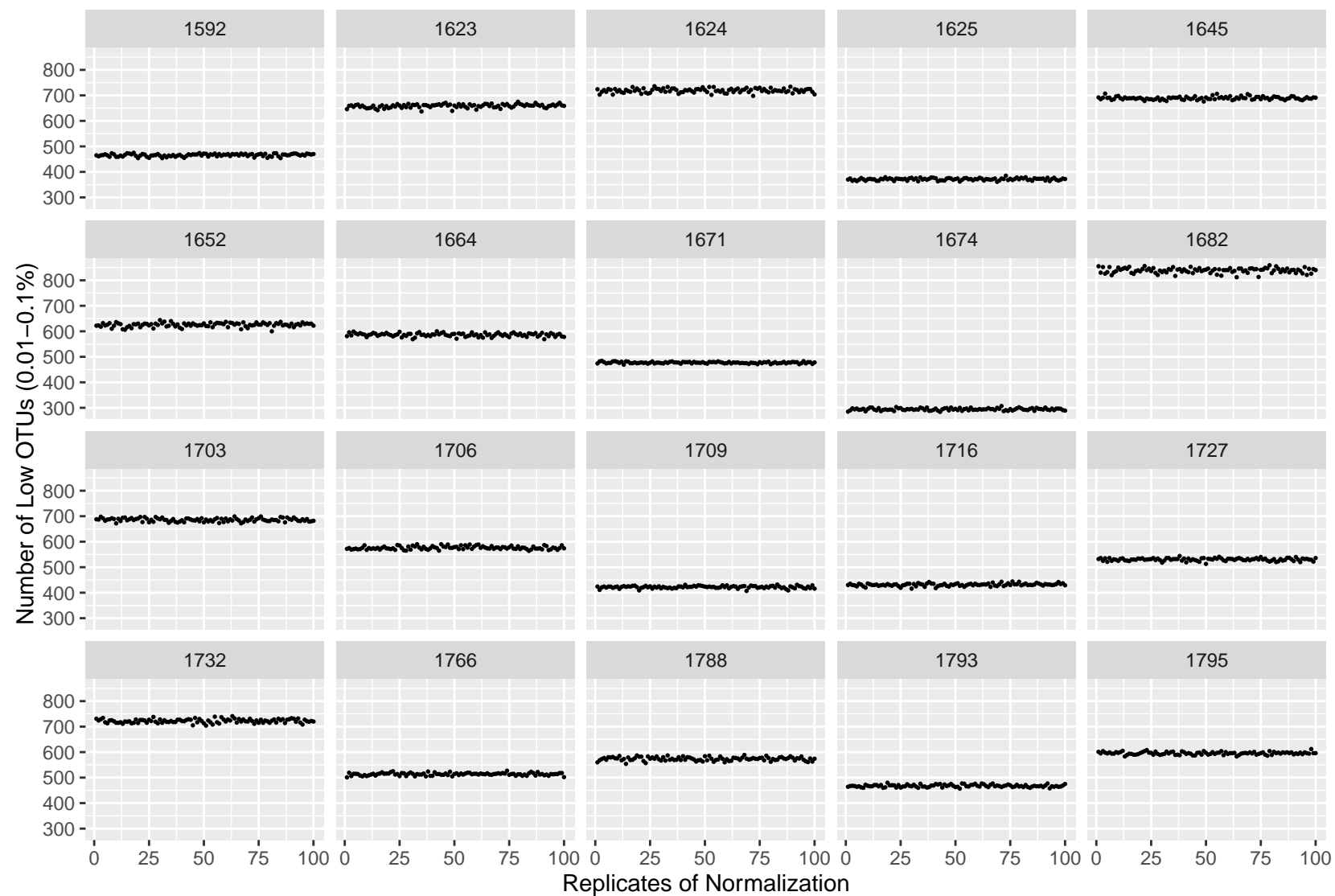

Supplement: S1 File — 100 replicates of the normalization step were done on each of the 200 randomly selected samples (10% of the samples). For each replicate, high-quality-reads were clustered, and obtained OTUs analyzed to determine the impact of the normalization step on OTUs. Four groups of OTUs were considered: Major (composed of more than 1% of reads), Medium (1–0.1% of reads), Low (0.01–0.1% of reads) and Rare (less than 0.01% of reads), showing no impact of the normalization step. (ZIP) [file pone.0186766.s007.zip › Low_Img_8.pdf]

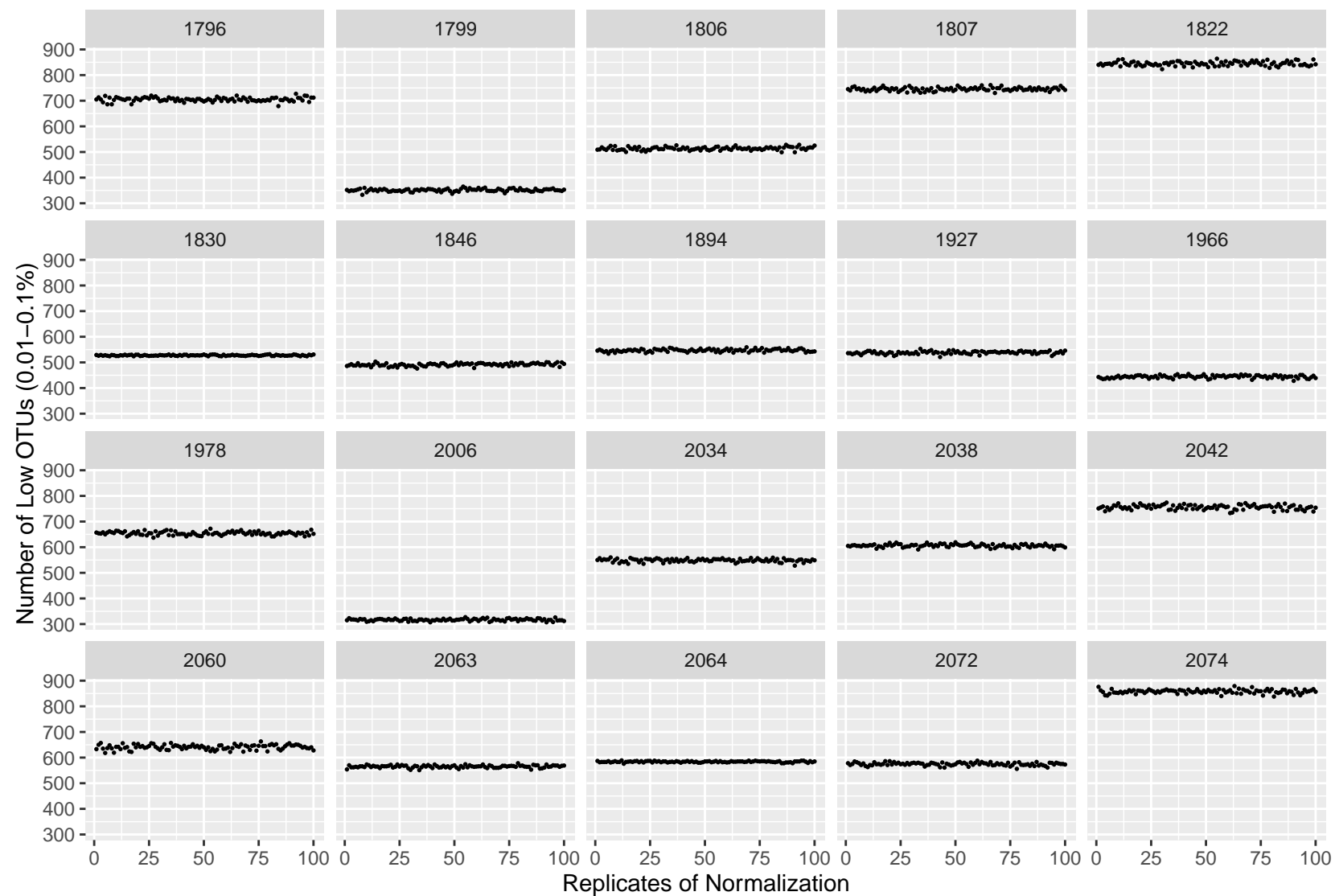

Supplement: S1 File — 100 replicates of the normalization step were done on each of the 200 randomly selected samples (10% of the samples). For each replicate, high-quality-reads were clustered, and obtained OTUs analyzed to determine the impact of the normalization step on OTUs. Four groups of OTUs were considered: Major (composed of more than 1% of reads), Medium (1–0.1% of reads), Low (0.01–0.1% of reads) and Rare (less than 0.01% of reads), showing no impact of the normalization step. (ZIP) [file pone.0186766.s007.zip › Low_Img_9.pdf]

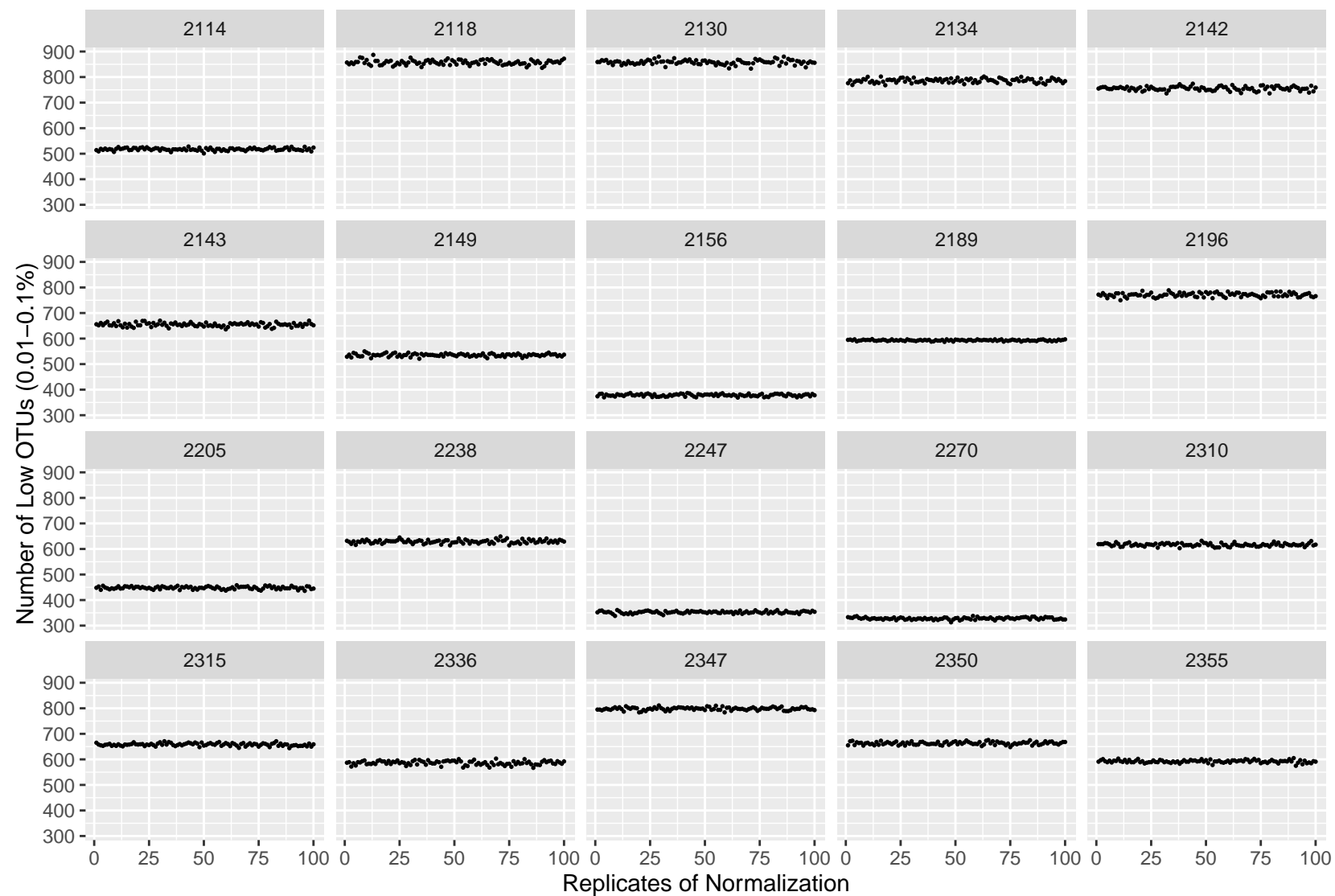

Supplement: S1 File — 100 replicates of the normalization step were done on each of the 200 randomly selected samples (10% of the samples). For each replicate, high-quality-reads were clustered, and obtained OTUs analyzed to determine the impact of the normalization step on OTUs. Four groups of OTUs were considered: Major (composed of more than 1% of reads), Medium (1–0.1% of reads), Low (0.01–0.1% of reads) and Rare (less than 0.01% of reads), showing no impact of the normalization step. (ZIP) [file pone.0186766.s007.zip › Low_Img_10.pdf]

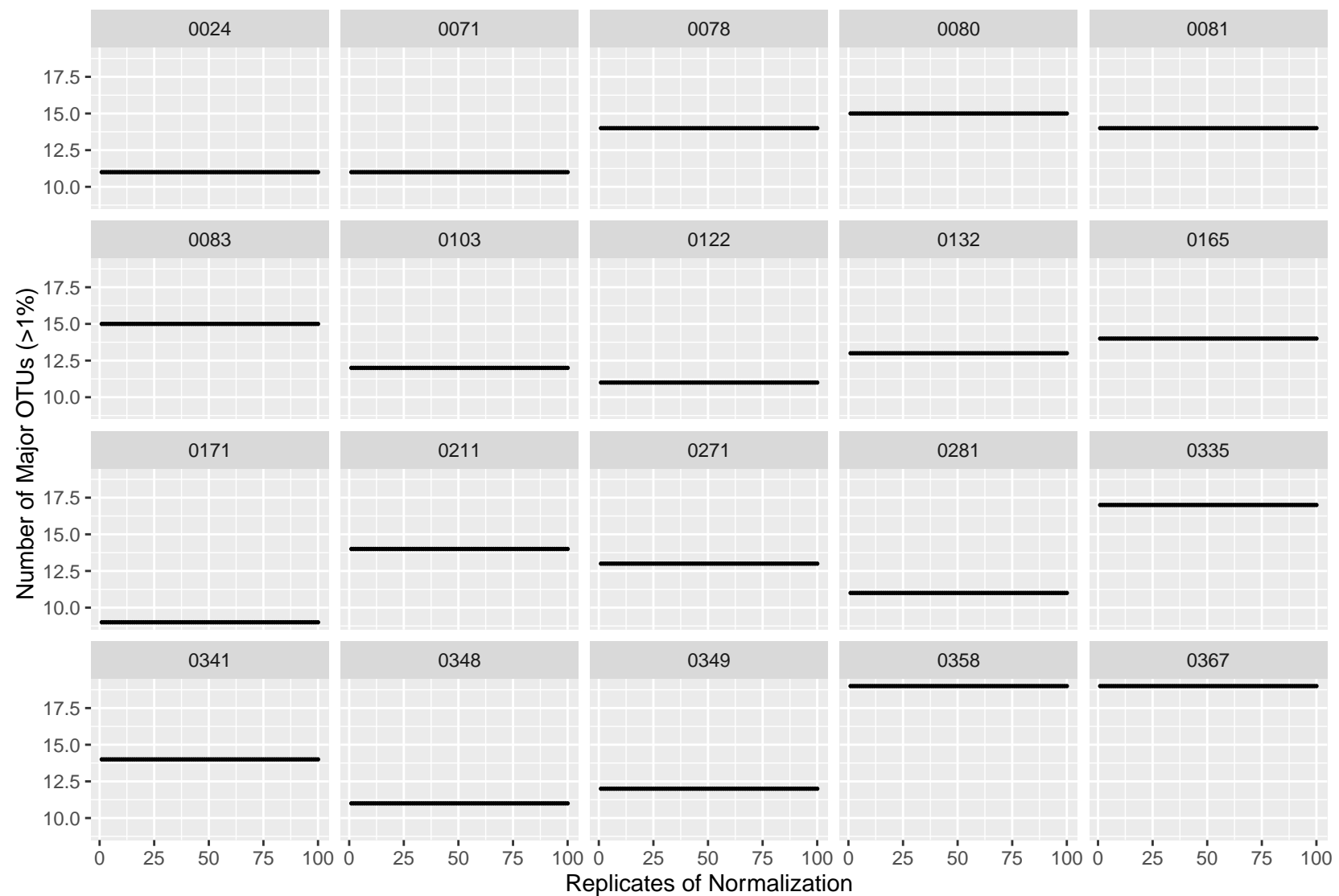

Supplement: S1 File — 100 replicates of the normalization step were done on each of the 200 randomly selected samples (10% of the samples). For each replicate, high-quality-reads were clustered, and obtained OTUs analyzed to determine the impact of the normalization step on OTUs. Four groups of OTUs were considered: Major (composed of more than 1% of reads), Medium (1–0.1% of reads), Low (0.01–0.1% of reads) and Rare (less than 0.01% of reads), showing no impact of the normalization step. (ZIP) [file pone.0186766.s007.zip › Major_Img_1.pdf]

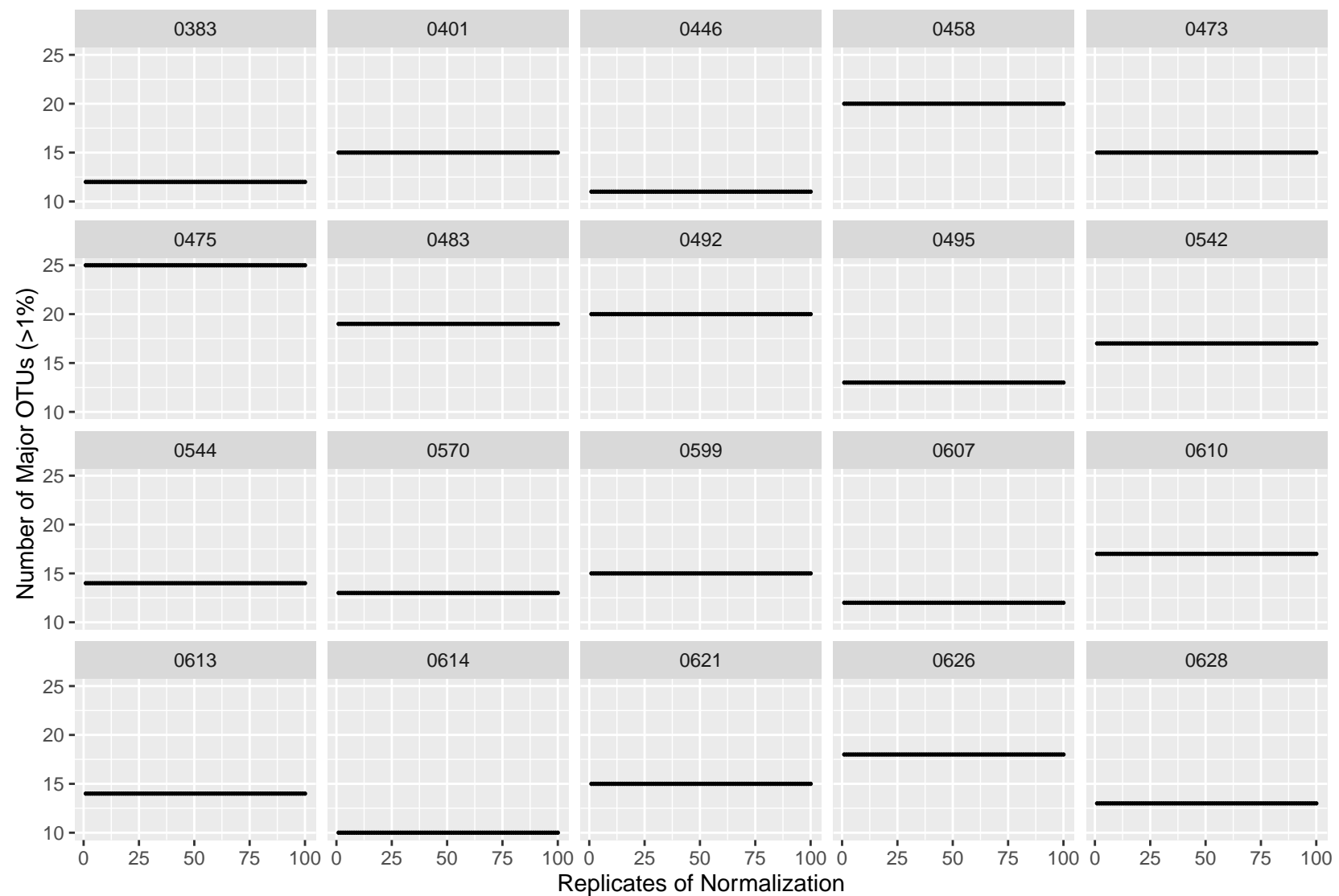

Supplement: S1 File — 100 replicates of the normalization step were done on each of the 200 randomly selected samples (10% of the samples). For each replicate, high-quality-reads were clustered, and obtained OTUs analyzed to determine the impact of the normalization step on OTUs. Four groups of OTUs were considered: Major (composed of more than 1% of reads), Medium (1–0.1% of reads), Low (0.01–0.1% of reads) and Rare (less than 0.01% of reads), showing no impact of the normalization step. (ZIP) [file pone.0186766.s007.zip › Major_Img_2.pdf]

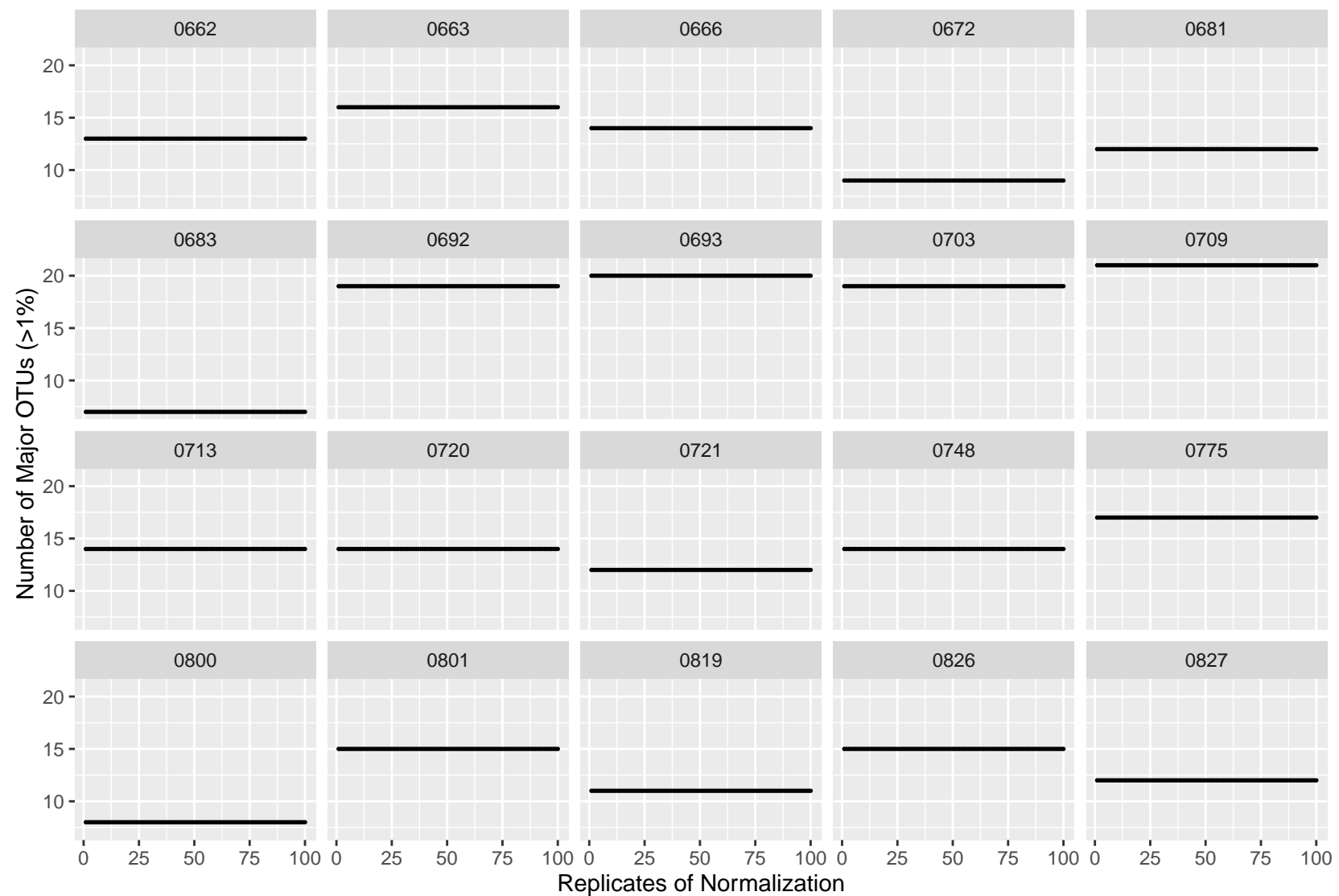

Supplement: S1 File — 100 replicates of the normalization step were done on each of the 200 randomly selected samples (10% of the samples). For each replicate, high-quality-reads were clustered, and obtained OTUs analyzed to determine the impact of the normalization step on OTUs. Four groups of OTUs were considered: Major (composed of more than 1% of reads), Medium (1–0.1% of reads), Low (0.01–0.1% of reads) and Rare (less than 0.01% of reads), showing no impact of the normalization step. (ZIP) [file pone.0186766.s007.zip › Major_Img_3.pdf]

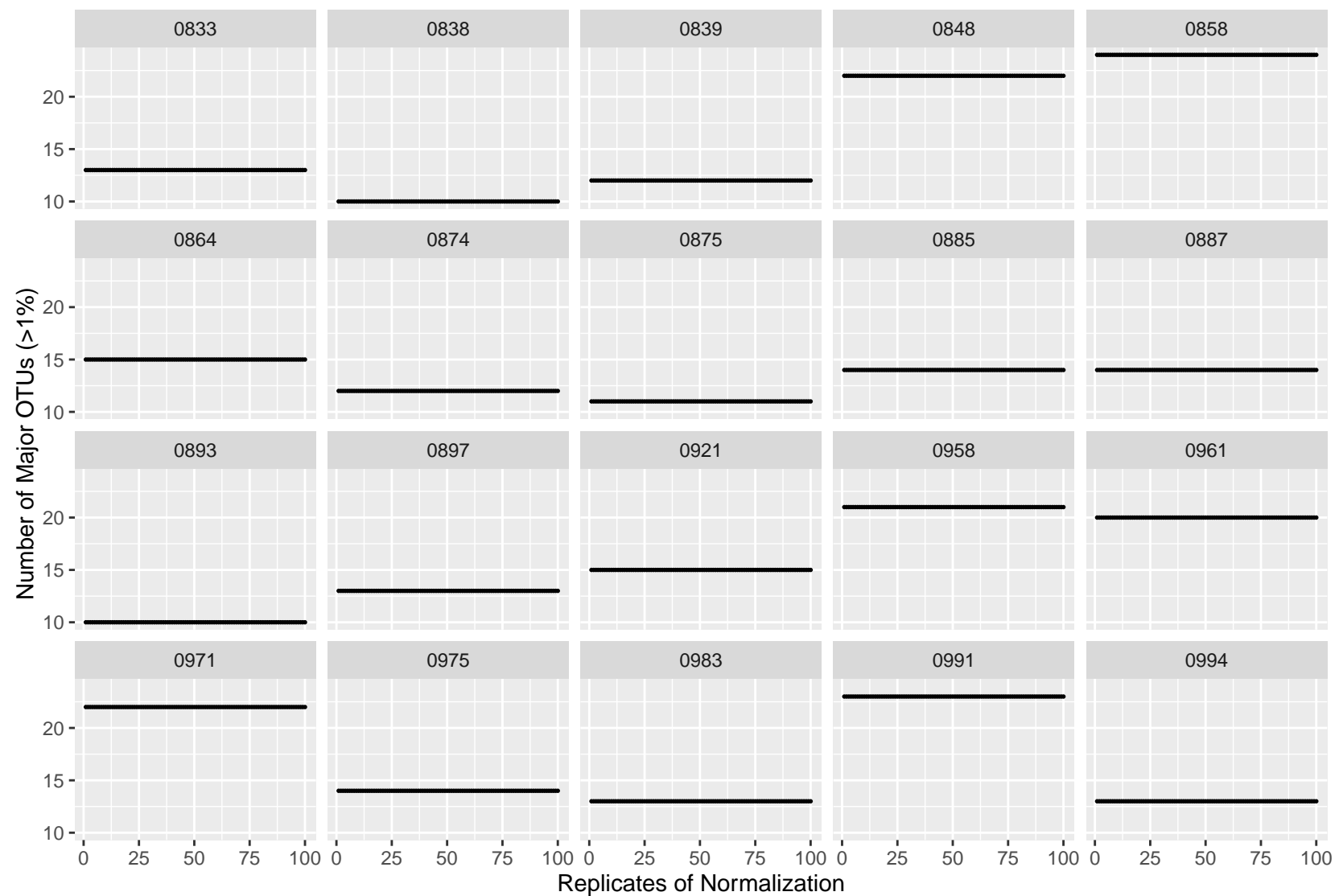

Supplement: S1 File — 100 replicates of the normalization step were done on each of the 200 randomly selected samples (10% of the samples). For each replicate, high-quality-reads were clustered, and obtained OTUs analyzed to determine the impact of the normalization step on OTUs. Four groups of OTUs were considered: Major (composed of more than 1% of reads), Medium (1–0.1% of reads), Low (0.01–0.1% of reads) and Rare (less than 0.01% of reads), showing no impact of the normalization step. (ZIP) [file pone.0186766.s007.zip › Major_Img_4.pdf]

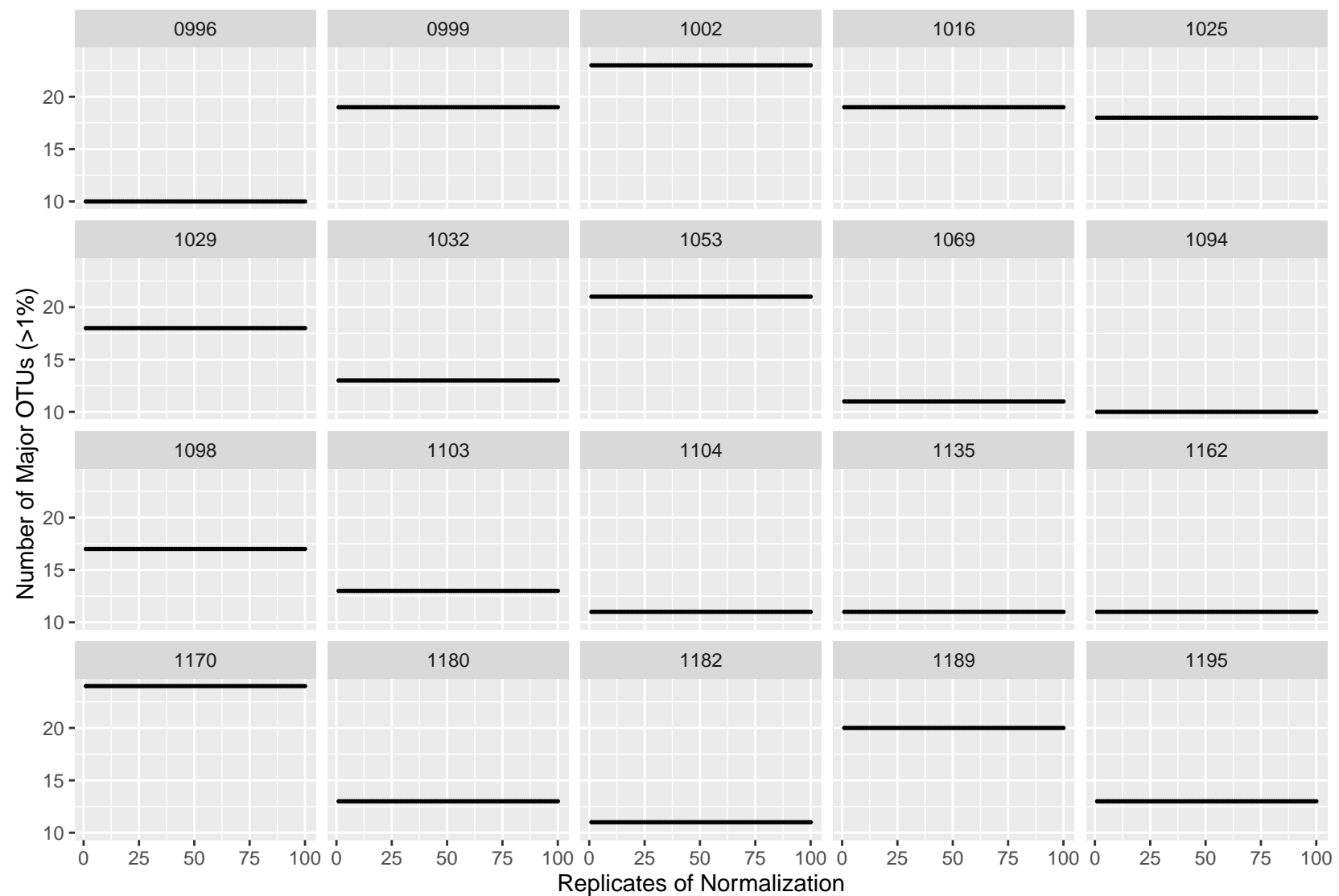

Supplement: S1 File — 100 replicates of the normalization step were done on each of the 200 randomly selected samples (10% of the samples). For each replicate, high-quality-reads were clustered, and obtained OTUs analyzed to determine the impact of the normalization step on OTUs. Four groups of OTUs were considered: Major (composed of more than 1% of reads), Medium (1–0.1% of reads), Low (0.01–0.1% of reads) and Rare (less than 0.01% of reads), showing no impact of the normalization step. (ZIP) [file pone.0186766.s007.zip › Major_Img_5.pdf]

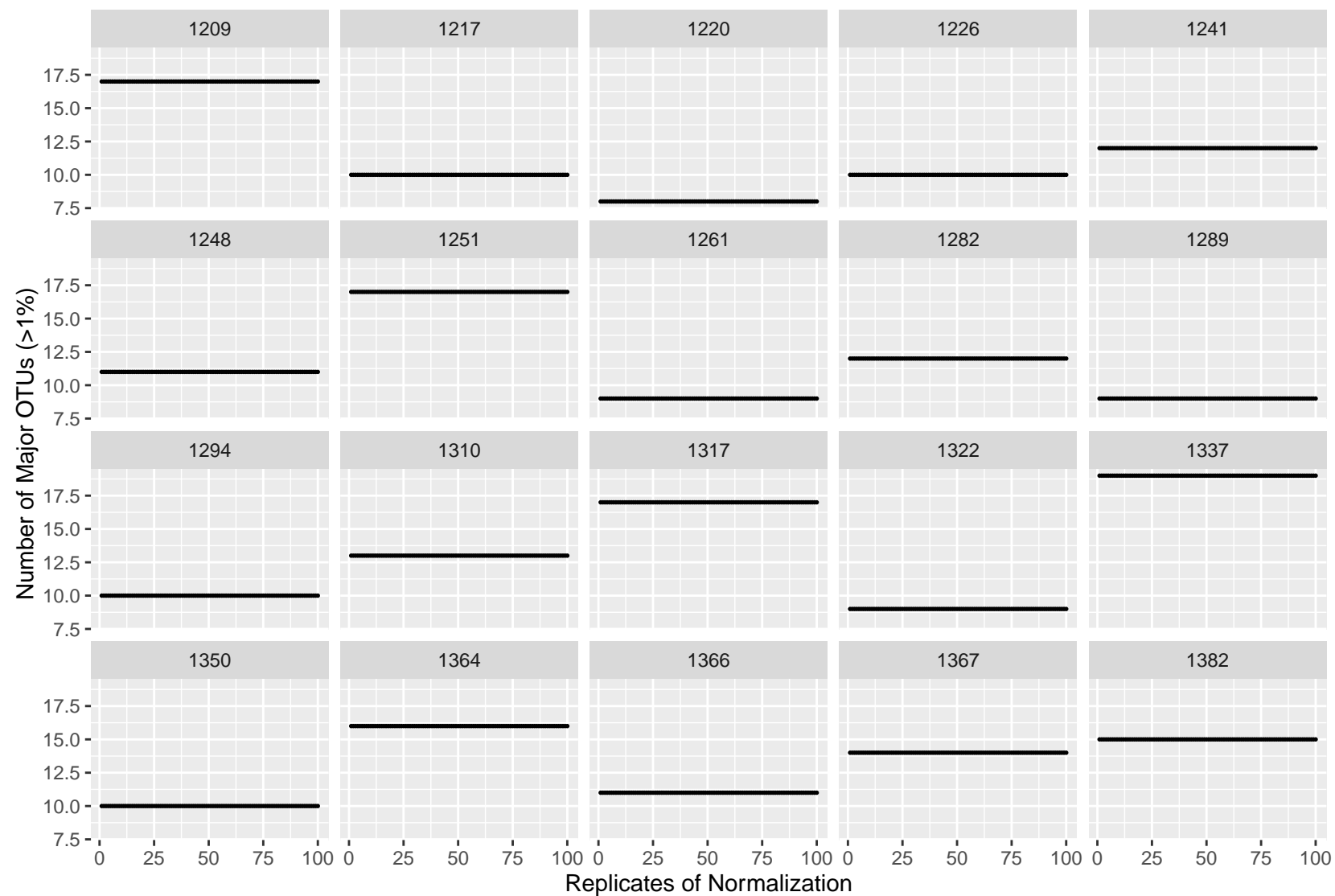

Supplement: S1 File — 100 replicates of the normalization step were done on each of the 200 randomly selected samples (10% of the samples). For each replicate, high-quality-reads were clustered, and obtained OTUs analyzed to determine the impact of the normalization step on OTUs. Four groups of OTUs were considered: Major (composed of more than 1% of reads), Medium (1–0.1% of reads), Low (0.01–0.1% of reads) and Rare (less than 0.01% of reads), showing no impact of the normalization step. (ZIP) [file pone.0186766.s007.zip › Major_Img_6.pdf]

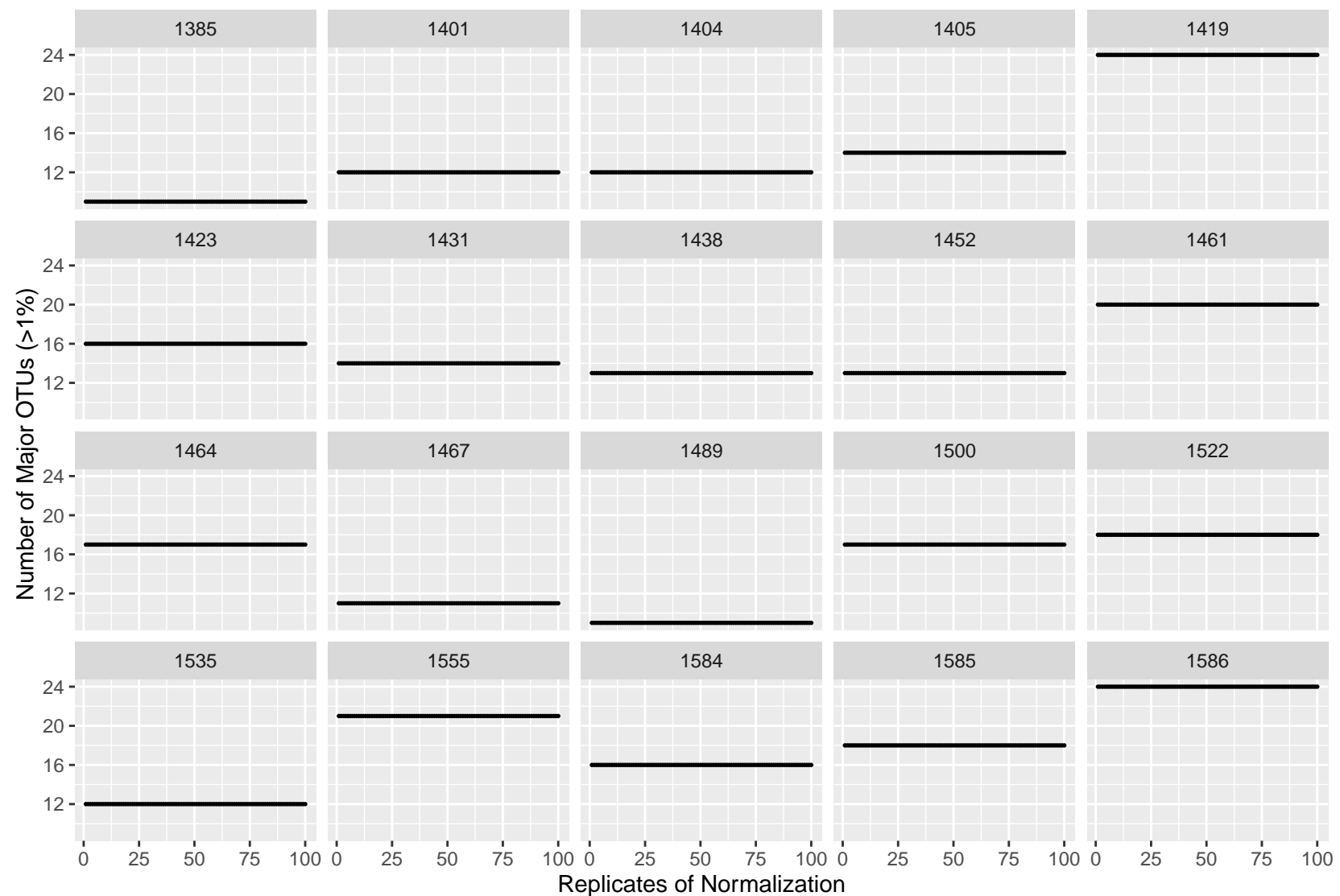

Supplement: S1 File — 100 replicates of the normalization step were done on each of the 200 randomly selected samples (10% of the samples). For each replicate, high-quality-reads were clustered, and obtained OTUs analyzed to determine the impact of the normalization step on OTUs. Four groups of OTUs were considered: Major (composed of more than 1% of reads), Medium (1–0.1% of reads), Low (0.01–0.1% of reads) and Rare (less than 0.01% of reads), showing no impact of the normalization step. (ZIP) [file pone.0186766.s007.zip › Major_Img_7.pdf]

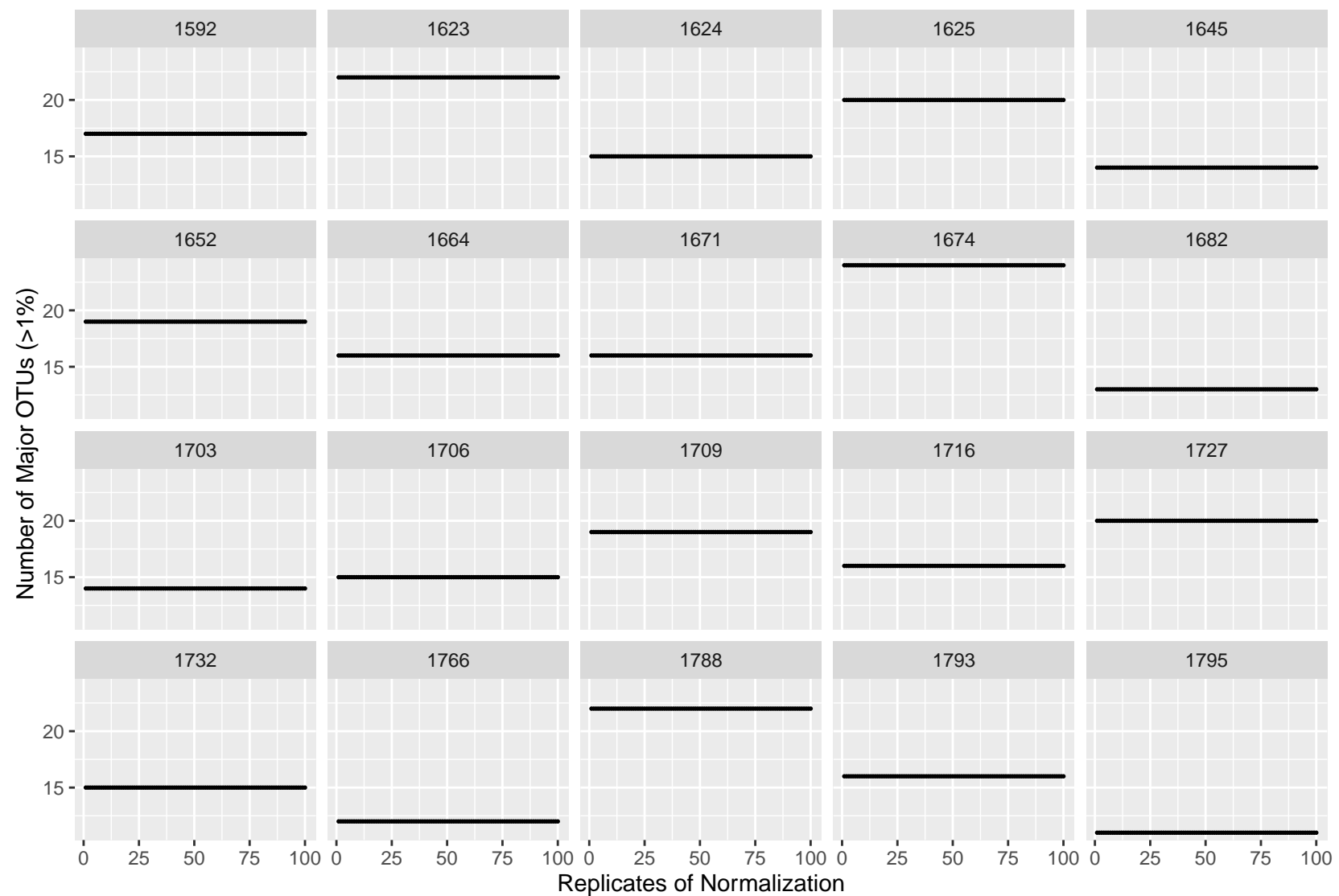

Supplement: S1 File — 100 replicates of the normalization step were done on each of the 200 randomly selected samples (10% of the samples). For each replicate, high-quality-reads were clustered, and obtained OTUs analyzed to determine the impact of the normalization step on OTUs. Four groups of OTUs were considered: Major (composed of more than 1% of reads), Medium (1–0.1% of reads), Low (0.01–0.1% of reads) and Rare (less than 0.01% of reads), showing no impact of the normalization step. (ZIP) [file pone.0186766.s007.zip › Major_Img_8.pdf]

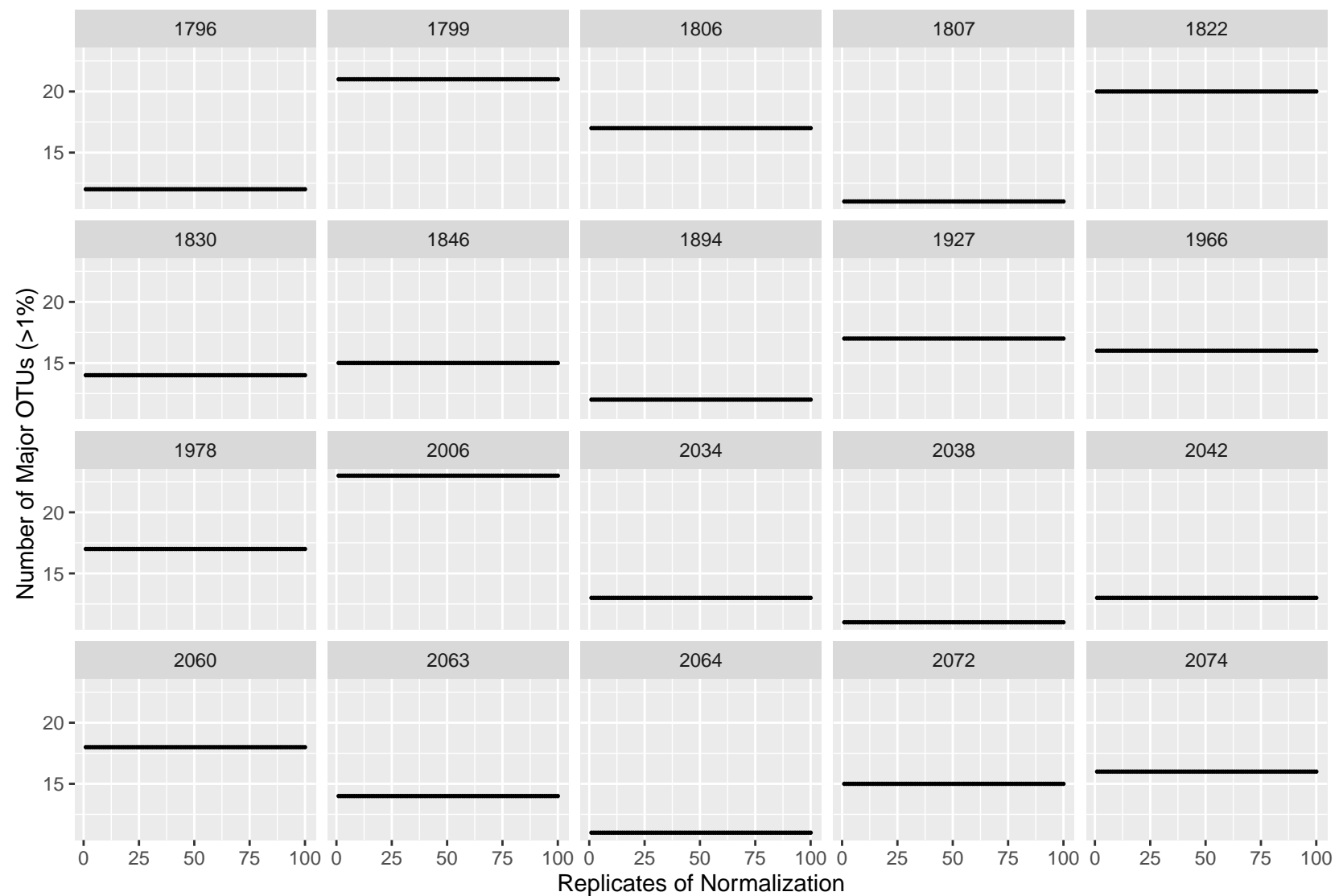

Supplement: S1 File — 100 replicates of the normalization step were done on each of the 200 randomly selected samples (10% of the samples). For each replicate, high-quality-reads were clustered, and obtained OTUs analyzed to determine the impact of the normalization step on OTUs. Four groups of OTUs were considered: Major (composed of more than 1% of reads), Medium (1–0.1% of reads), Low (0.01–0.1% of reads) and Rare (less than 0.01% of reads), showing no impact of the normalization step. (ZIP) [file pone.0186766.s007.zip › Major_Img_9.pdf]

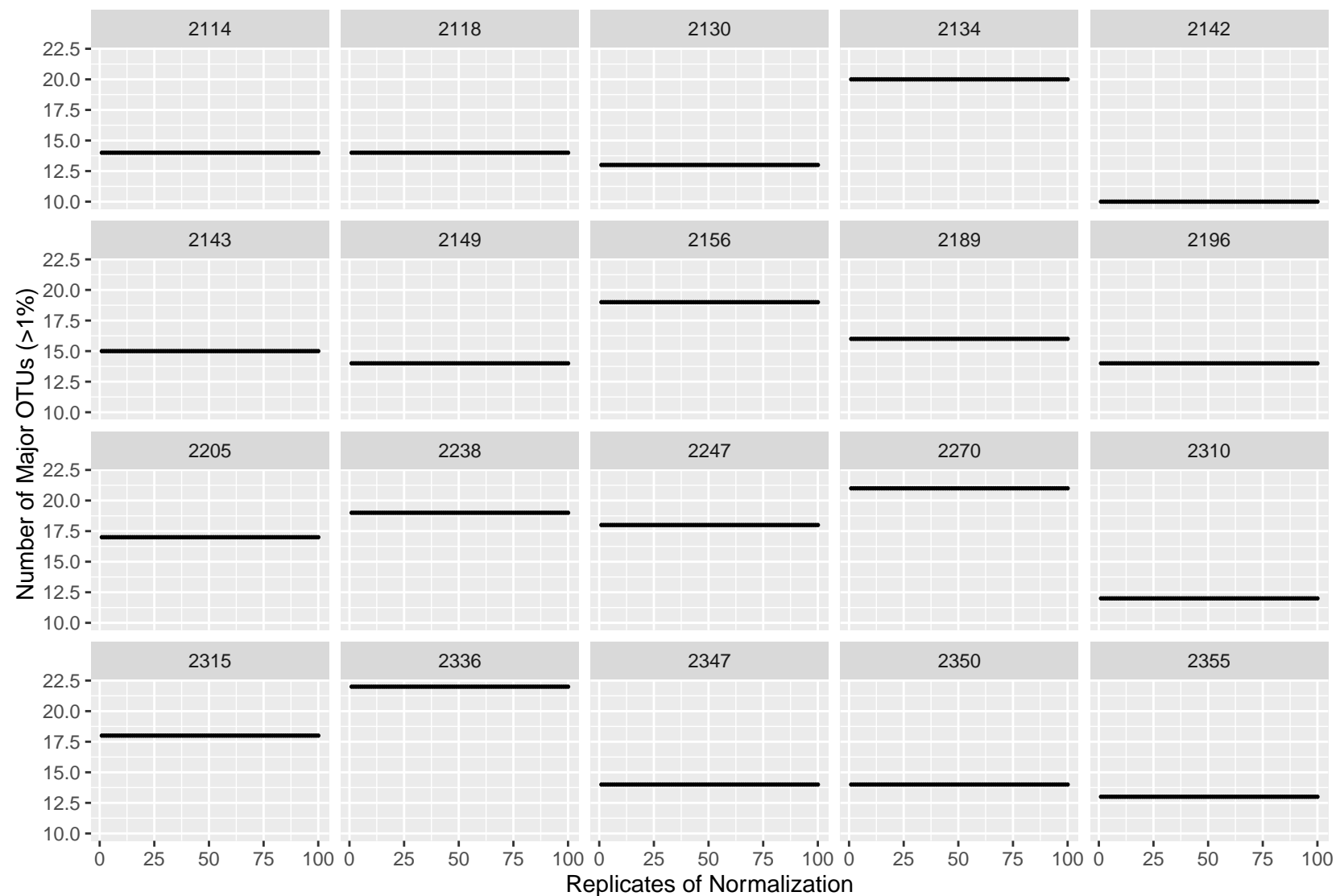

Supplement: S1 File — 100 replicates of the normalization step were done on each of the 200 randomly selected samples (10% of the samples). For each replicate, high-quality-reads were clustered, and obtained OTUs analyzed to determine the impact of the normalization step on OTUs. Four groups of OTUs were considered: Major (composed of more than 1% of reads), Medium (1–0.1% of reads), Low (0.01–0.1% of reads) and Rare (less than 0.01% of reads), showing no impact of the normalization step. (ZIP) [file pone.0186766.s007.zip › Major_Img_10.pdf]

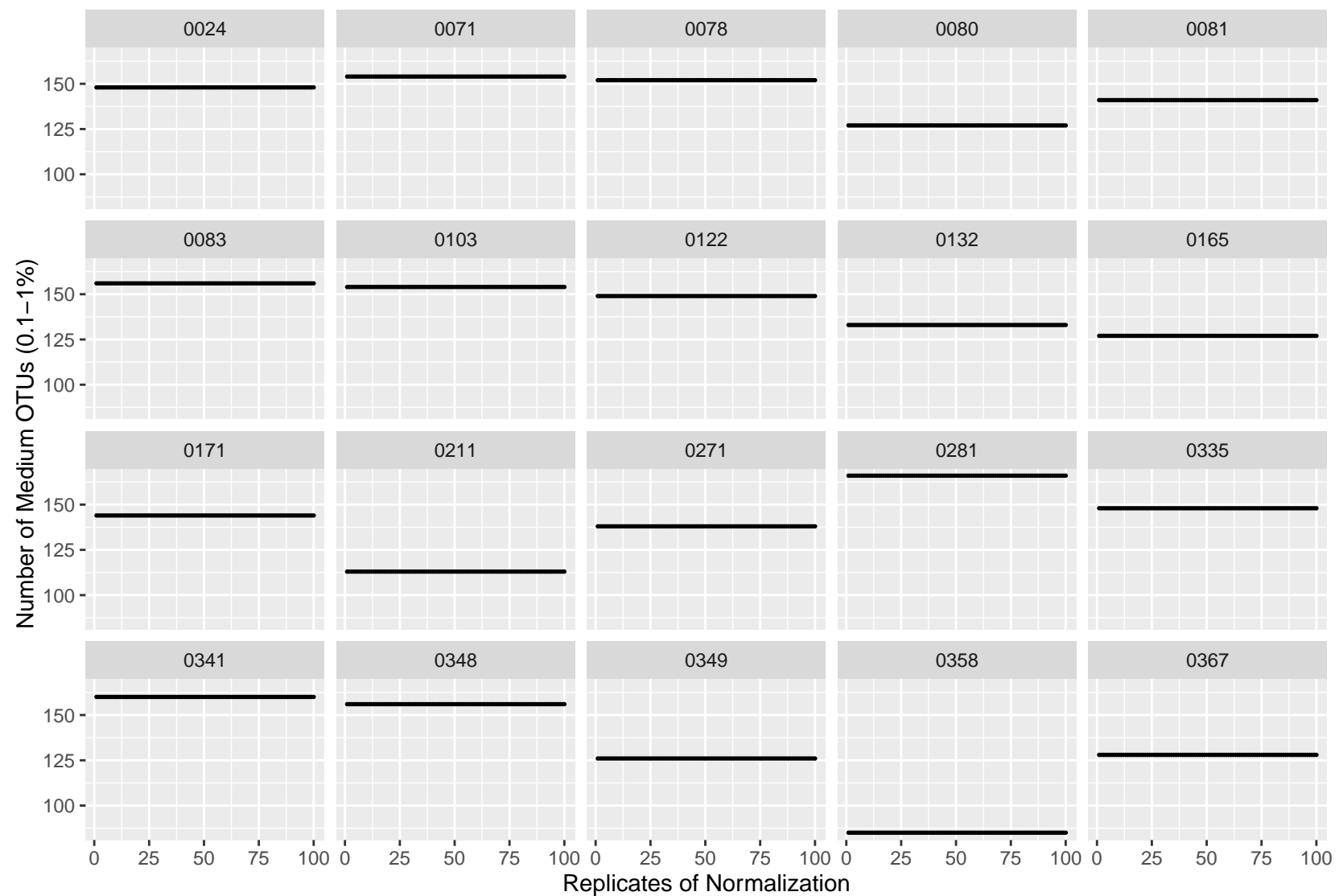

Supplement: S1 File — 100 replicates of the normalization step were done on each of the 200 randomly selected samples (10% of the samples). For each replicate, high-quality-reads were clustered, and obtained OTUs analyzed to determine the impact of the normalization step on OTUs. Four groups of OTUs were considered: Major (composed of more than 1% of reads), Medium (1–0.1% of reads), Low (0.01–0.1% of reads) and Rare (less than 0.01% of reads), showing no impact of the normalization step. (ZIP) [file pone.0186766.s007.zip › Medium_Img_1.pdf]

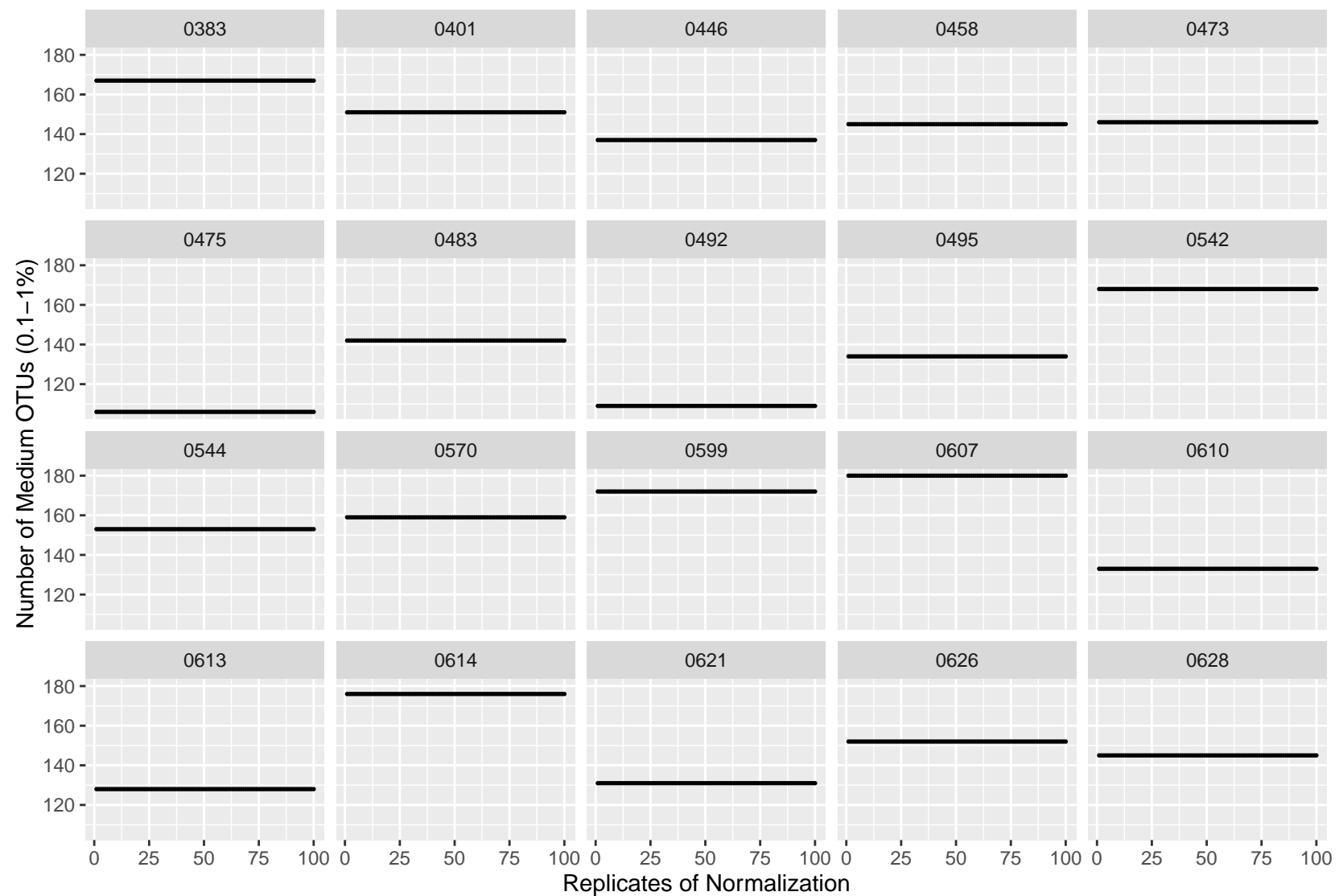

Supplement: S1 File — 100 replicates of the normalization step were done on each of the 200 randomly selected samples (10% of the samples). For each replicate, high-quality-reads were clustered, and obtained OTUs analyzed to determine the impact of the normalization step on OTUs. Four groups of OTUs were considered: Major (composed of more than 1% of reads), Medium (1–0.1% of reads), Low (0.01–0.1% of reads) and Rare (less than 0.01% of reads), showing no impact of the normalization step. (ZIP) [file pone.0186766.s007.zip › Medium_Img_2.pdf]

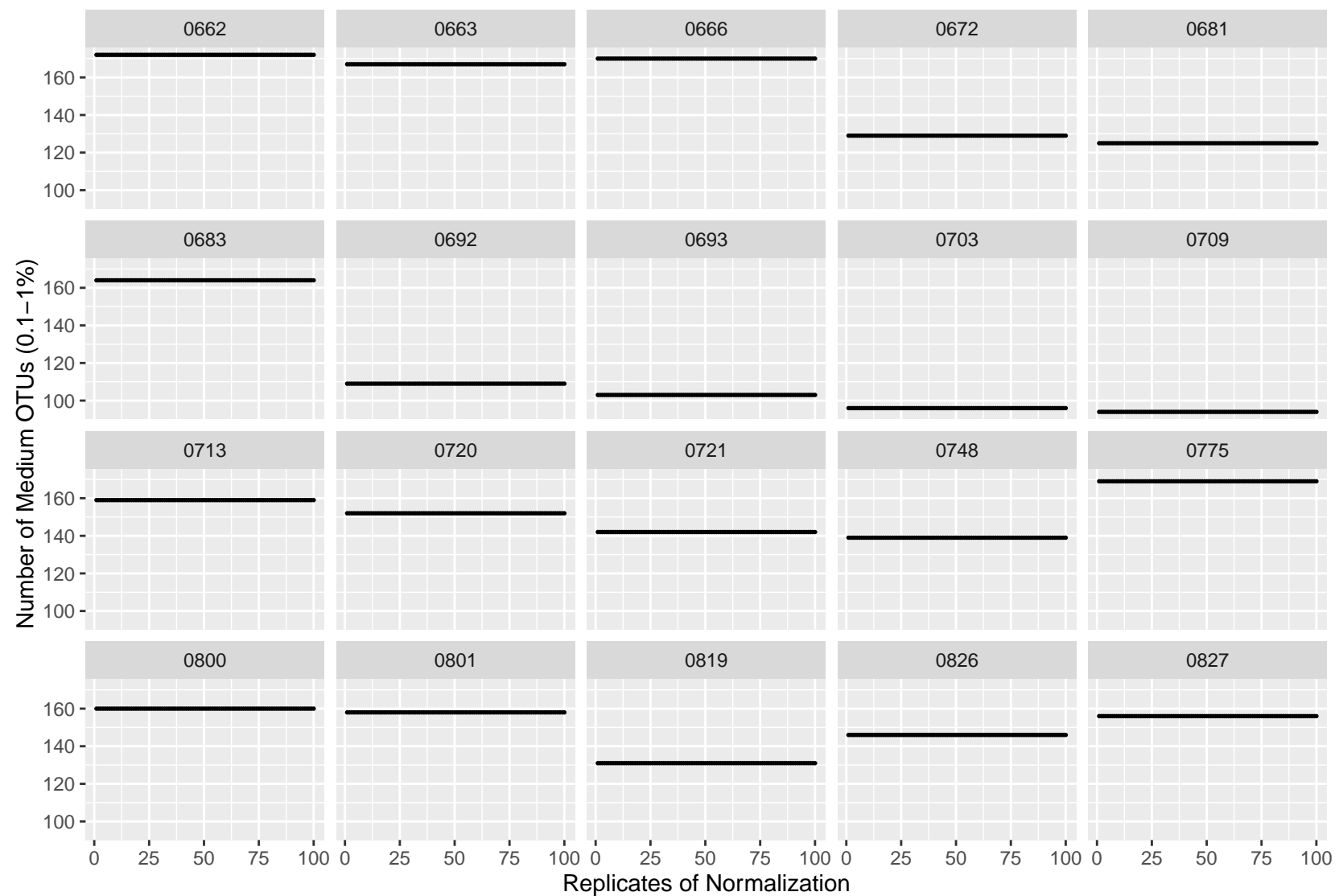

Supplement: S1 File — 100 replicates of the normalization step were done on each of the 200 randomly selected samples (10% of the samples). For each replicate, high-quality-reads were clustered, and obtained OTUs analyzed to determine the impact of the normalization step on OTUs. Four groups of OTUs were considered: Major (composed of more than 1% of reads), Medium (1–0.1% of reads), Low (0.01–0.1% of reads) and Rare (less than 0.01% of reads), showing no impact of the normalization step. (ZIP) [file pone.0186766.s007.zip › Medium_Img_3.pdf]

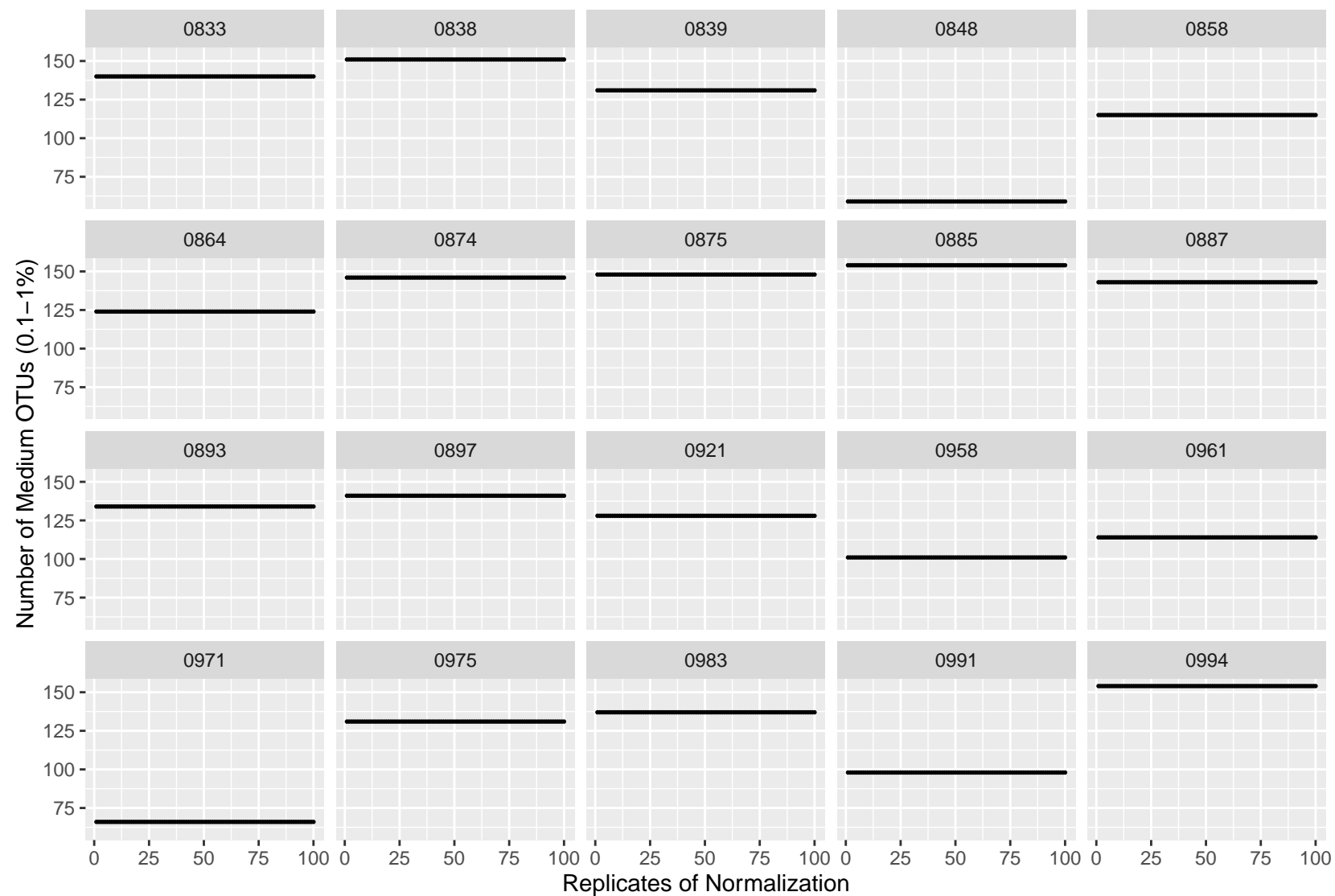

Supplement: S1 File — 100 replicates of the normalization step were done on each of the 200 randomly selected samples (10% of the samples). For each replicate, high-quality-reads were clustered, and obtained OTUs analyzed to determine the impact of the normalization step on OTUs. Four groups of OTUs were considered: Major (composed of more than 1% of reads), Medium (1–0.1% of reads), Low (0.01–0.1% of reads) and Rare (less than 0.01% of reads), showing no impact of the normalization step. (ZIP) [file pone.0186766.s007.zip › Medium_Img_4.pdf]

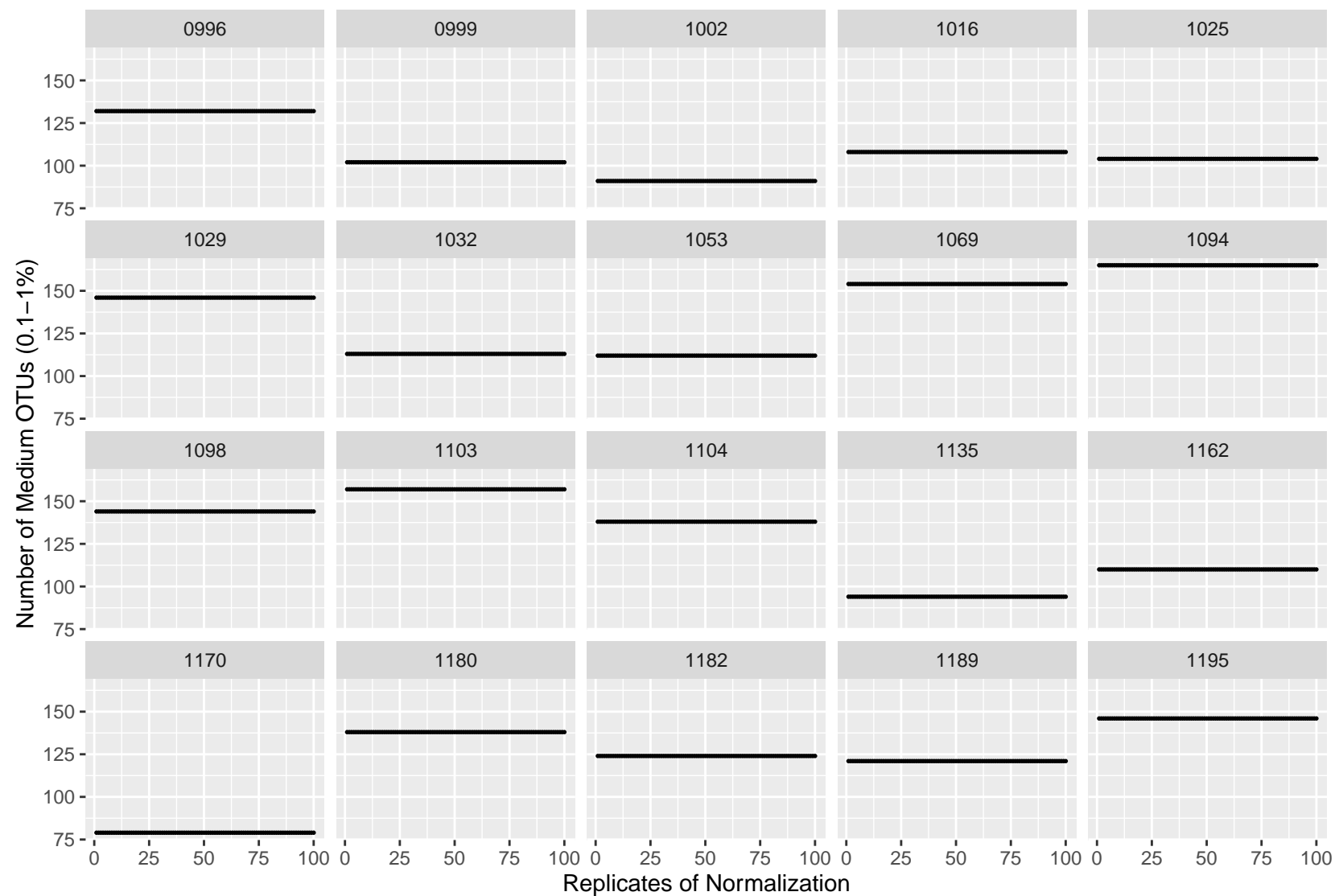

Supplement: S1 File — 100 replicates of the normalization step were done on each of the 200 randomly selected samples (10% of the samples). For each replicate, high-quality-reads were clustered, and obtained OTUs analyzed to determine the impact of the normalization step on OTUs. Four groups of OTUs were considered: Major (composed of more than 1% of reads), Medium (1–0.1% of reads), Low (0.01–0.1% of reads) and Rare (less than 0.01% of reads), showing no impact of the normalization step. (ZIP) [file pone.0186766.s007.zip › Medium_Img_5.pdf]

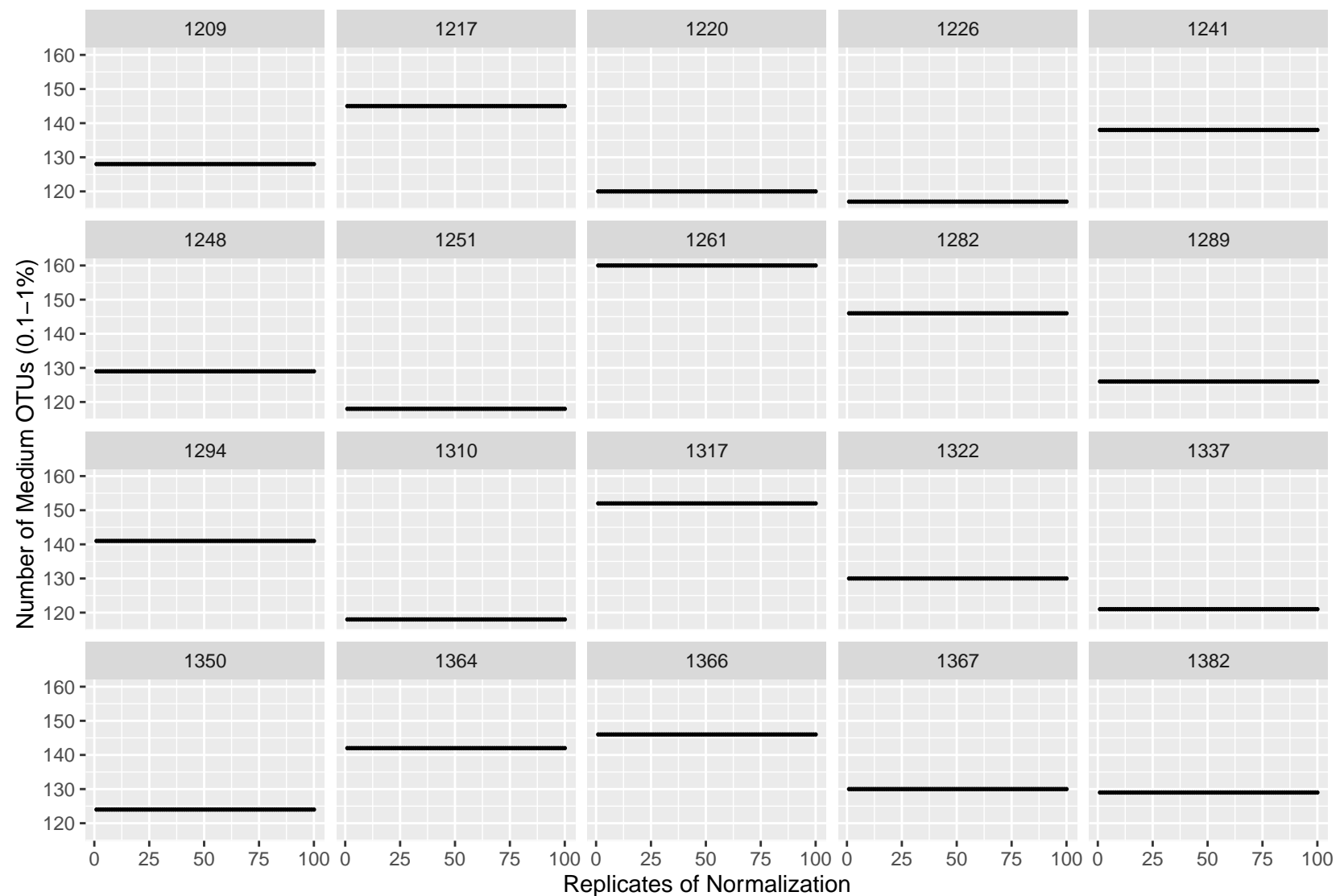

Supplement: S1 File — 100 replicates of the normalization step were done on each of the 200 randomly selected samples (10% of the samples). For each replicate, high-quality-reads were clustered, and obtained OTUs analyzed to determine the impact of the normalization step on OTUs. Four groups of OTUs were considered: Major (composed of more than 1% of reads), Medium (1–0.1% of reads), Low (0.01–0.1% of reads) and Rare (less than 0.01% of reads), showing no impact of the normalization step. (ZIP) [file pone.0186766.s007.zip › Medium_Img_6.pdf]

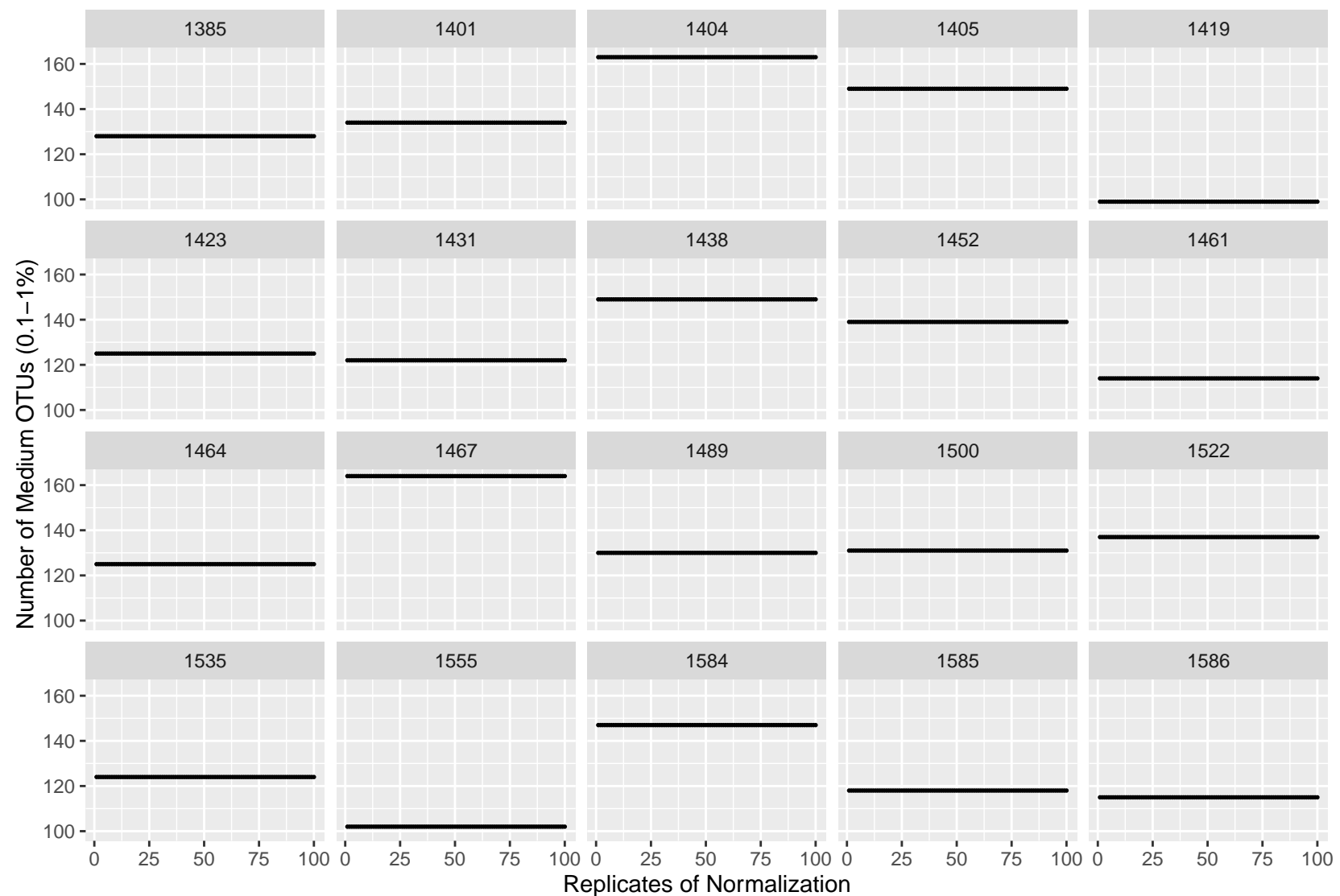

Supplement: S1 File — 100 replicates of the normalization step were done on each of the 200 randomly selected samples (10% of the samples). For each replicate, high-quality-reads were clustered, and obtained OTUs analyzed to determine the impact of the normalization step on OTUs. Four groups of OTUs were considered: Major (composed of more than 1% of reads), Medium (1–0.1% of reads), Low (0.01–0.1% of reads) and Rare (less than 0.01% of reads), showing no impact of the normalization step. (ZIP) [file pone.0186766.s007.zip › Medium_Img_7.pdf]

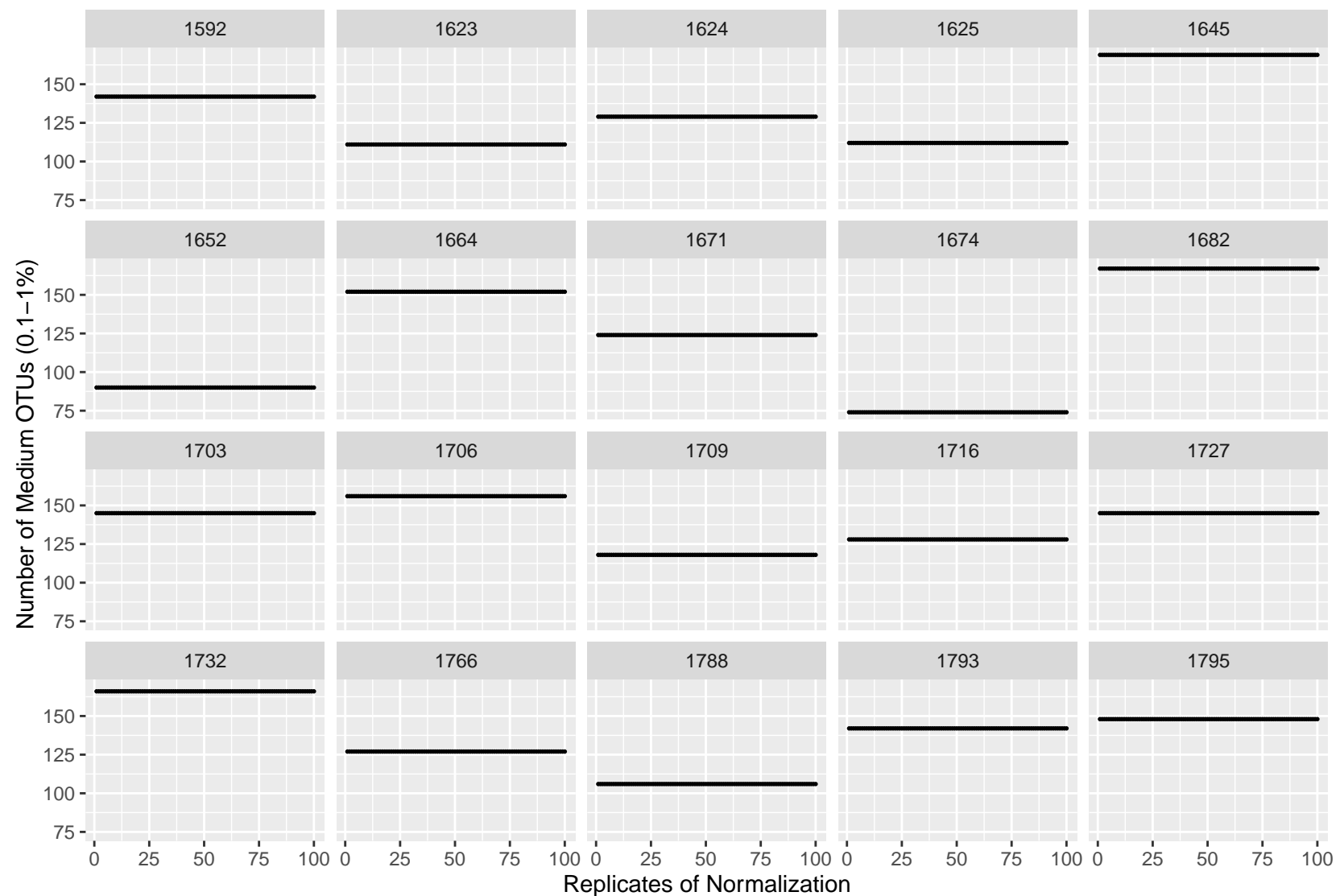

Supplement: S1 File — 100 replicates of the normalization step were done on each of the 200 randomly selected samples (10% of the samples). For each replicate, high-quality-reads were clustered, and obtained OTUs analyzed to determine the impact of the normalization step on OTUs. Four groups of OTUs were considered: Major (composed of more than 1% of reads), Medium (1–0.1% of reads), Low (0.01–0.1% of reads) and Rare (less than 0.01% of reads), showing no impact of the normalization step. (ZIP) [file pone.0186766.s007.zip › Medium_Img_8.pdf]

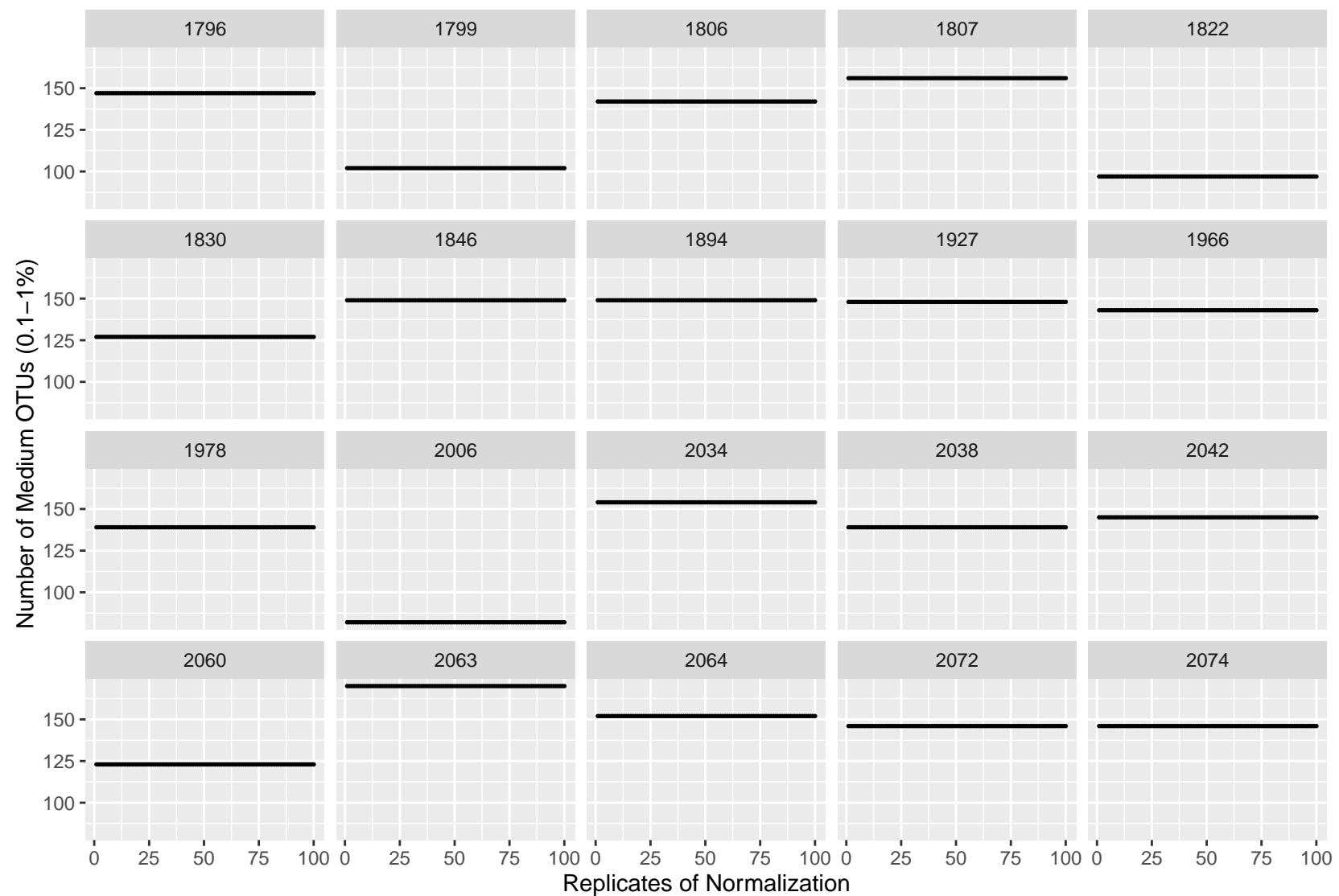

Supplement: S1 File — 100 replicates of the normalization step were done on each of the 200 randomly selected samples (10% of the samples). For each replicate, high-quality-reads were clustered, and obtained OTUs analyzed to determine the impact of the normalization step on OTUs. Four groups of OTUs were considered: Major (composed of more than 1% of reads), Medium (1–0.1% of reads), Low (0.01–0.1% of reads) and Rare (less than 0.01% of reads), showing no impact of the normalization step. (ZIP) [file pone.0186766.s007.zip › Medium_Img_9.pdf]

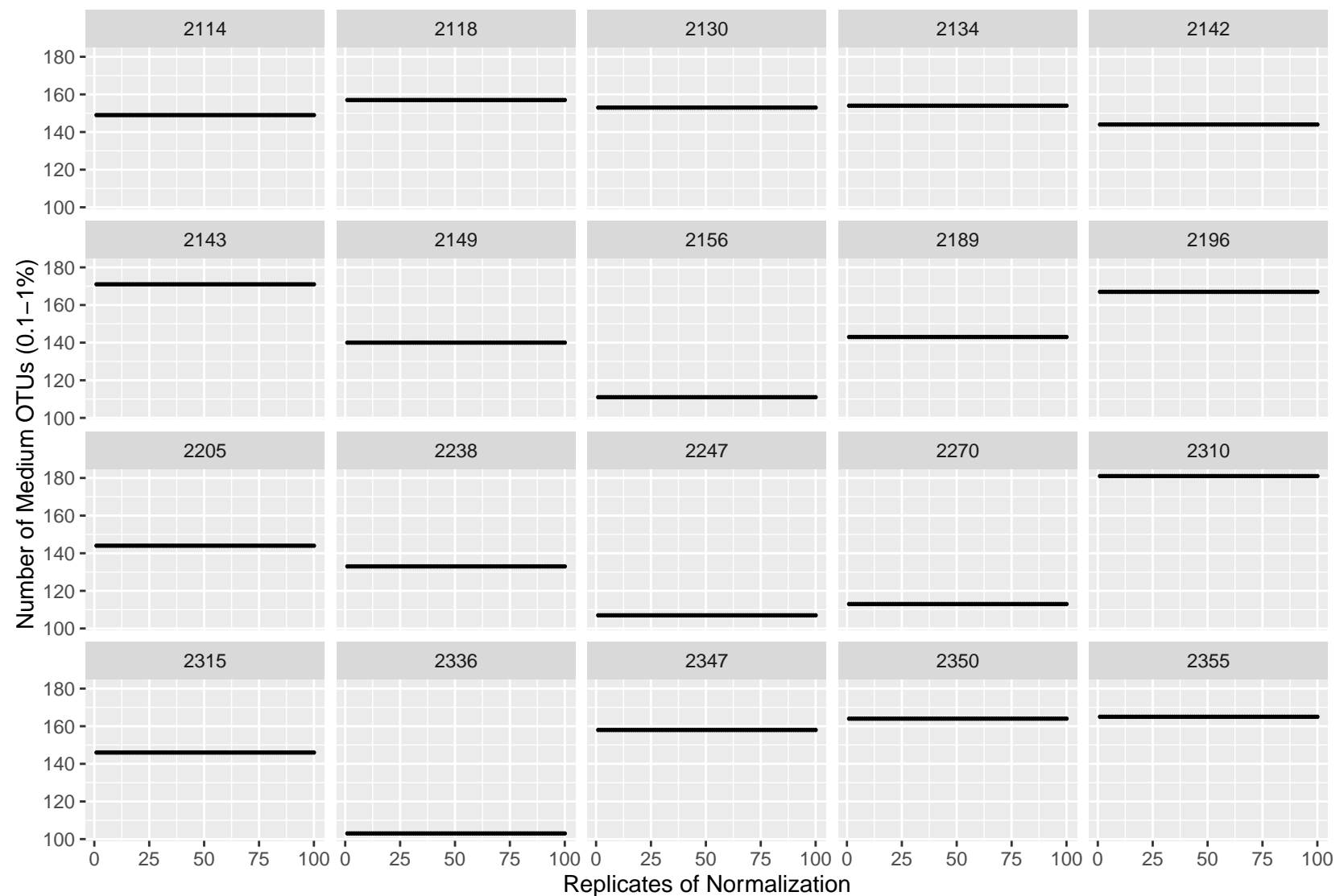

Supplement: S1 File — 100 replicates of the normalization step were done on each of the 200 randomly selected samples (10% of the samples). For each replicate, high-quality-reads were clustered, and obtained OTUs analyzed to determine the impact of the normalization step on OTUs. Four groups of OTUs were considered: Major (composed of more than 1% of reads), Medium (1–0.1% of reads), Low (0.01–0.1% of reads) and Rare (less than 0.01% of reads), showing no impact of the normalization step. (ZIP) [file pone.0186766.s007.zip › Medium_Img_10.pdf]

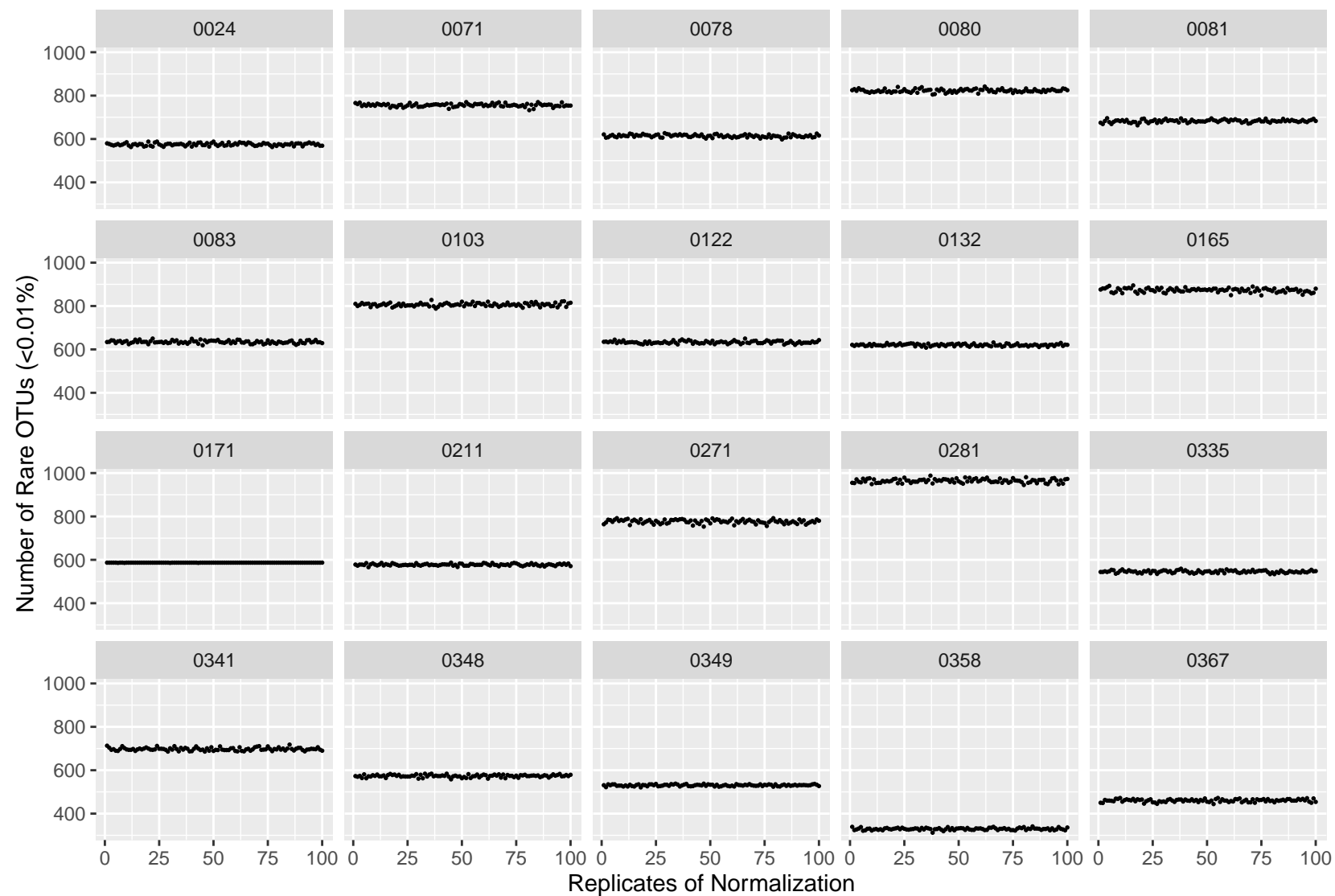

Supplement: S1 File — 100 replicates of the normalization step were done on each of the 200 randomly selected samples (10% of the samples). For each replicate, high-quality-reads were clustered, and obtained OTUs analyzed to determine the impact of the normalization step on OTUs. Four groups of OTUs were considered: Major (composed of more than 1% of reads), Medium (1–0.1% of reads), Low (0.01–0.1% of reads) and Rare (less than 0.01% of reads), showing no impact of the normalization step. (ZIP) [file pone.0186766.s007.zip › Rare_Img_1.pdf]

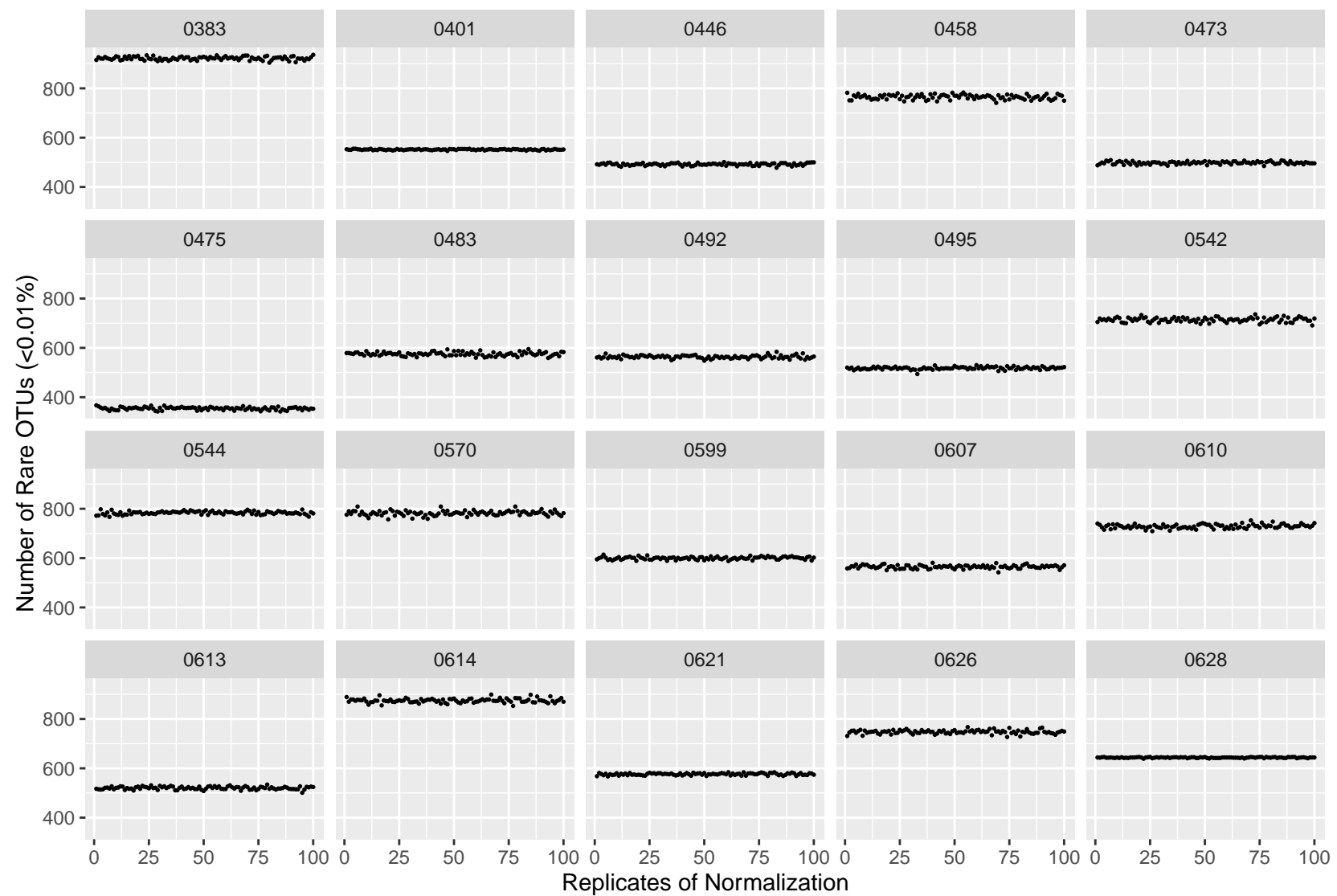

Supplement: S1 File — 100 replicates of the normalization step were done on each of the 200 randomly selected samples (10% of the samples). For each replicate, high-quality-reads were clustered, and obtained OTUs analyzed to determine the impact of the normalization step on OTUs. Four groups of OTUs were considered: Major (composed of more than 1% of reads), Medium (1–0.1% of reads), Low (0.01–0.1% of reads) and Rare (less than 0.01% of reads), showing no impact of the normalization step. (ZIP) [file pone.0186766.s007.zip › Rare_Img_2.pdf]

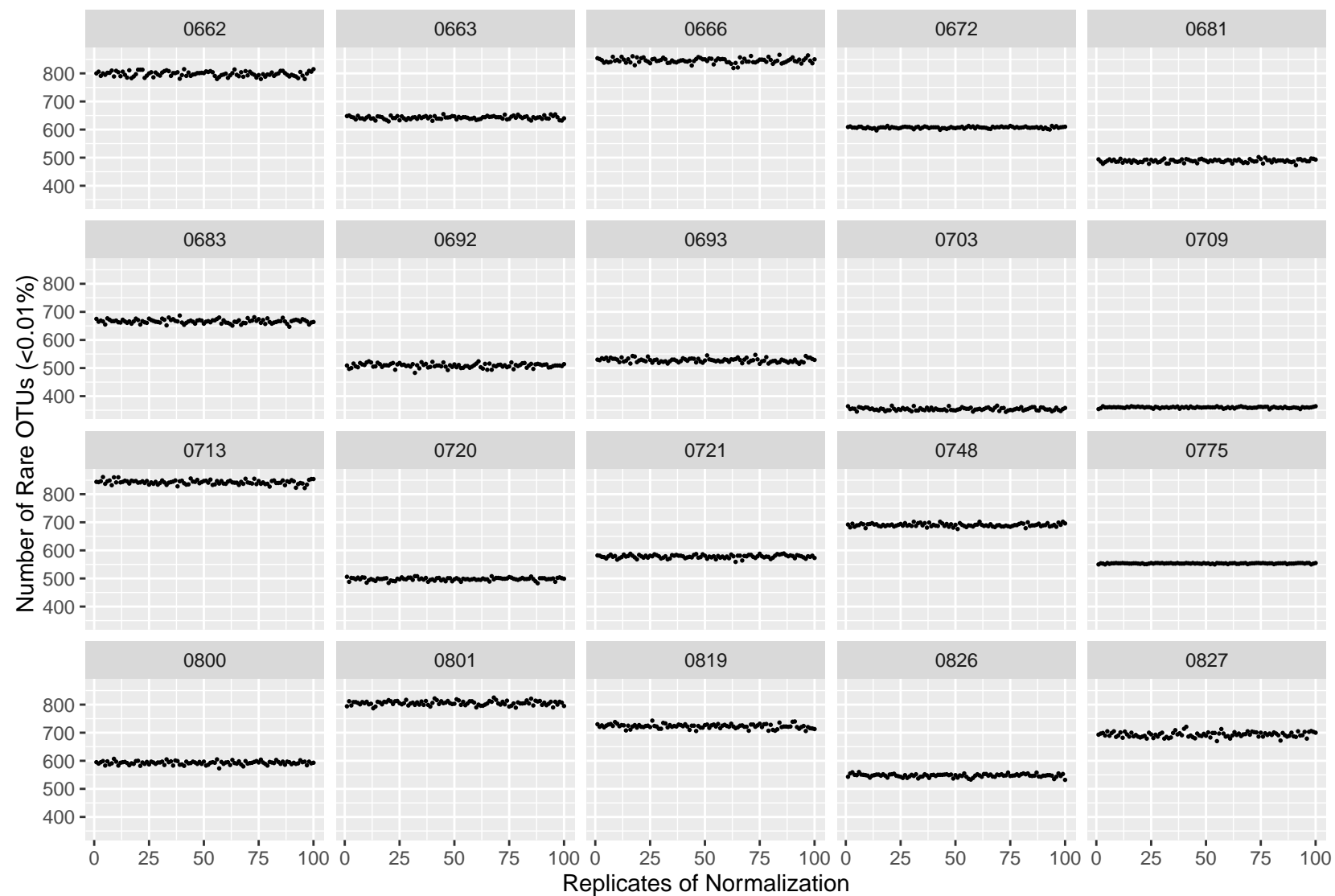

Supplement: S1 File — 100 replicates of the normalization step were done on each of the 200 randomly selected samples (10% of the samples). For each replicate, high-quality-reads were clustered, and obtained OTUs analyzed to determine the impact of the normalization step on OTUs. Four groups of OTUs were considered: Major (composed of more than 1% of reads), Medium (1–0.1% of reads), Low (0.01–0.1% of reads) and Rare (less than 0.01% of reads), showing no impact of the normalization step. (ZIP) [file pone.0186766.s007.zip › Rare_Img_3.pdf]

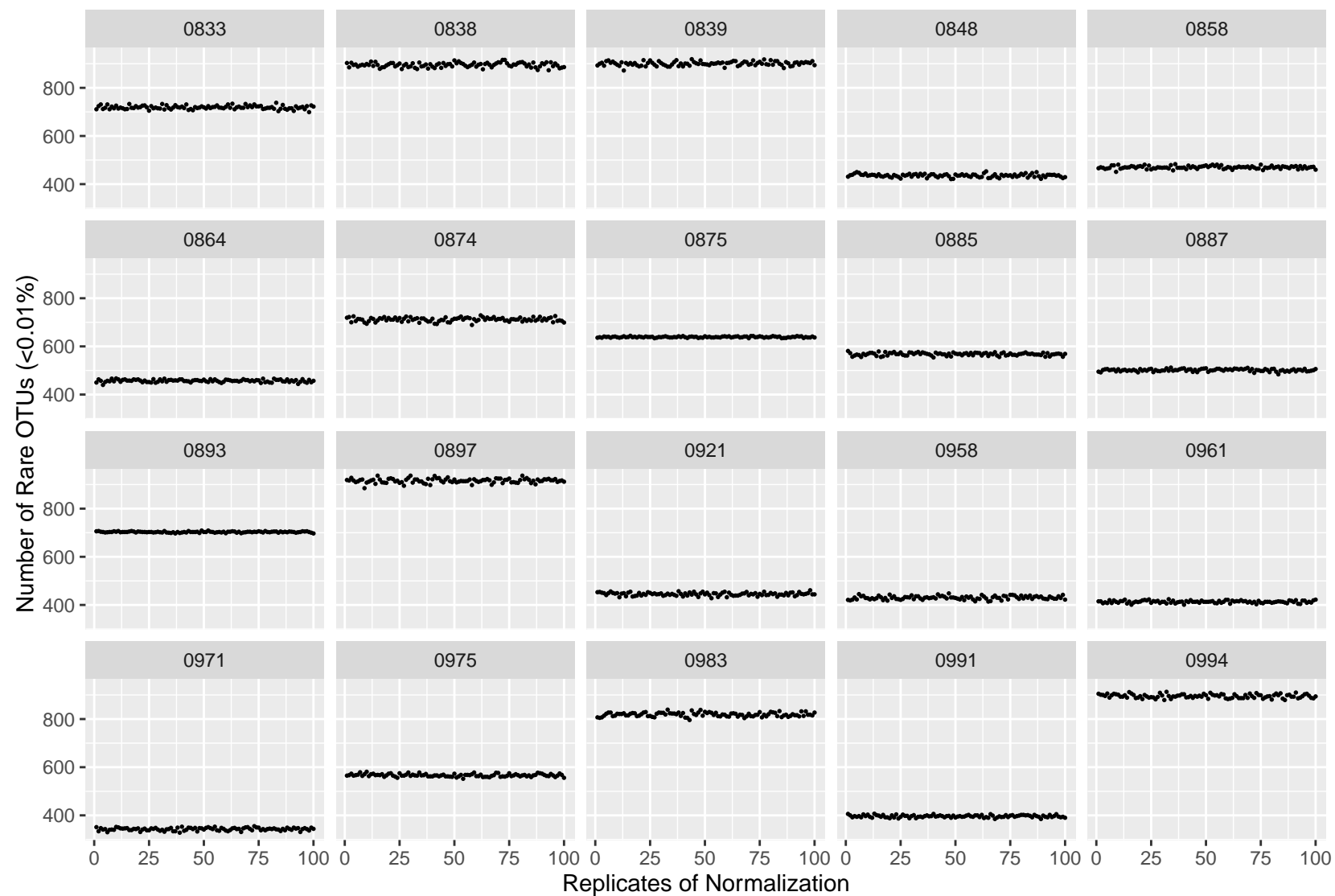

Supplement: S1 File — 100 replicates of the normalization step were done on each of the 200 randomly selected samples (10% of the samples). For each replicate, high-quality-reads were clustered, and obtained OTUs analyzed to determine the impact of the normalization step on OTUs. Four groups of OTUs were considered: Major (composed of more than 1% of reads), Medium (1–0.1% of reads), Low (0.01–0.1% of reads) and Rare (less than 0.01% of reads), showing no impact of the normalization step. (ZIP) [file pone.0186766.s007.zip › Rare_Img_4.pdf]

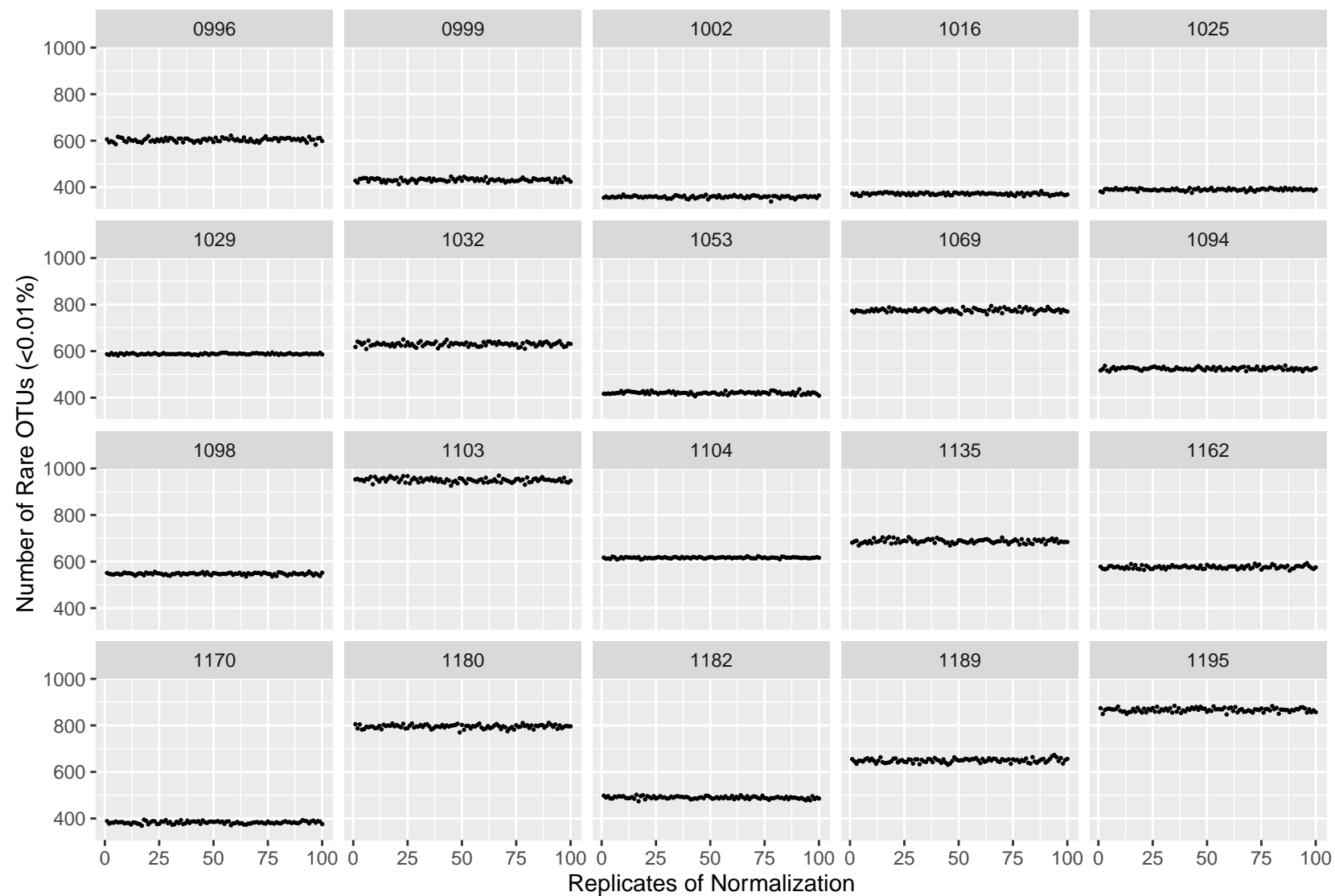

Supplement: S1 File — 100 replicates of the normalization step were done on each of the 200 randomly selected samples (10% of the samples). For each replicate, high-quality-reads were clustered, and obtained OTUs analyzed to determine the impact of the normalization step on OTUs. Four groups of OTUs were considered: Major (composed of more than 1% of reads), Medium (1–0.1% of reads), Low (0.01–0.1% of reads) and Rare (less than 0.01% of reads), showing no impact of the normalization step. (ZIP) [file pone.0186766.s007.zip › Rare_Img_5.pdf]

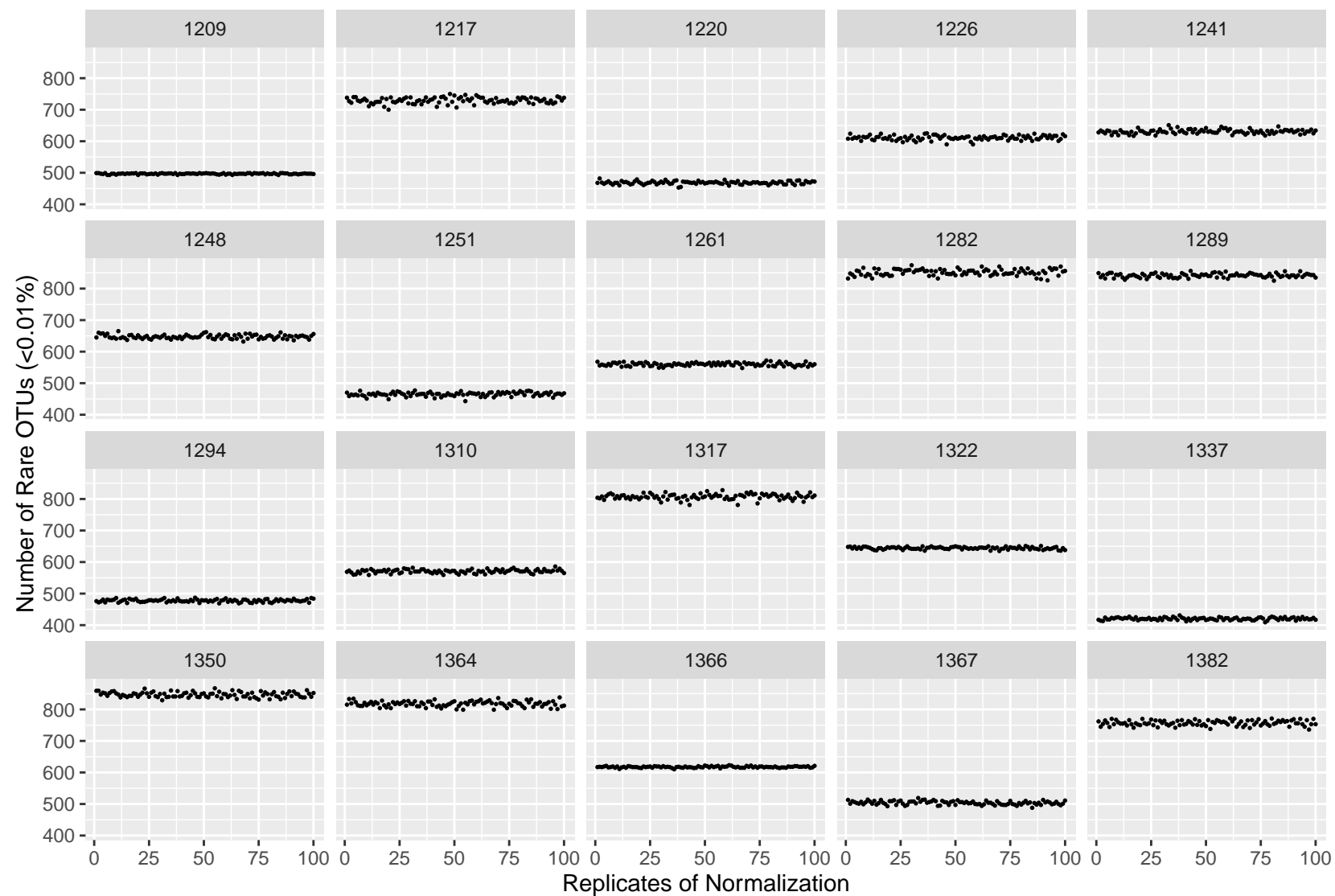

Supplement: S1 File — 100 replicates of the normalization step were done on each of the 200 randomly selected samples (10% of the samples). For each replicate, high-quality-reads were clustered, and obtained OTUs analyzed to determine the impact of the normalization step on OTUs. Four groups of OTUs were considered: Major (composed of more than 1% of reads), Medium (1–0.1% of reads), Low (0.01–0.1% of reads) and Rare (less than 0.01% of reads), showing no impact of the normalization step. (ZIP) [file pone.0186766.s007.zip › Rare_Img_6.pdf]

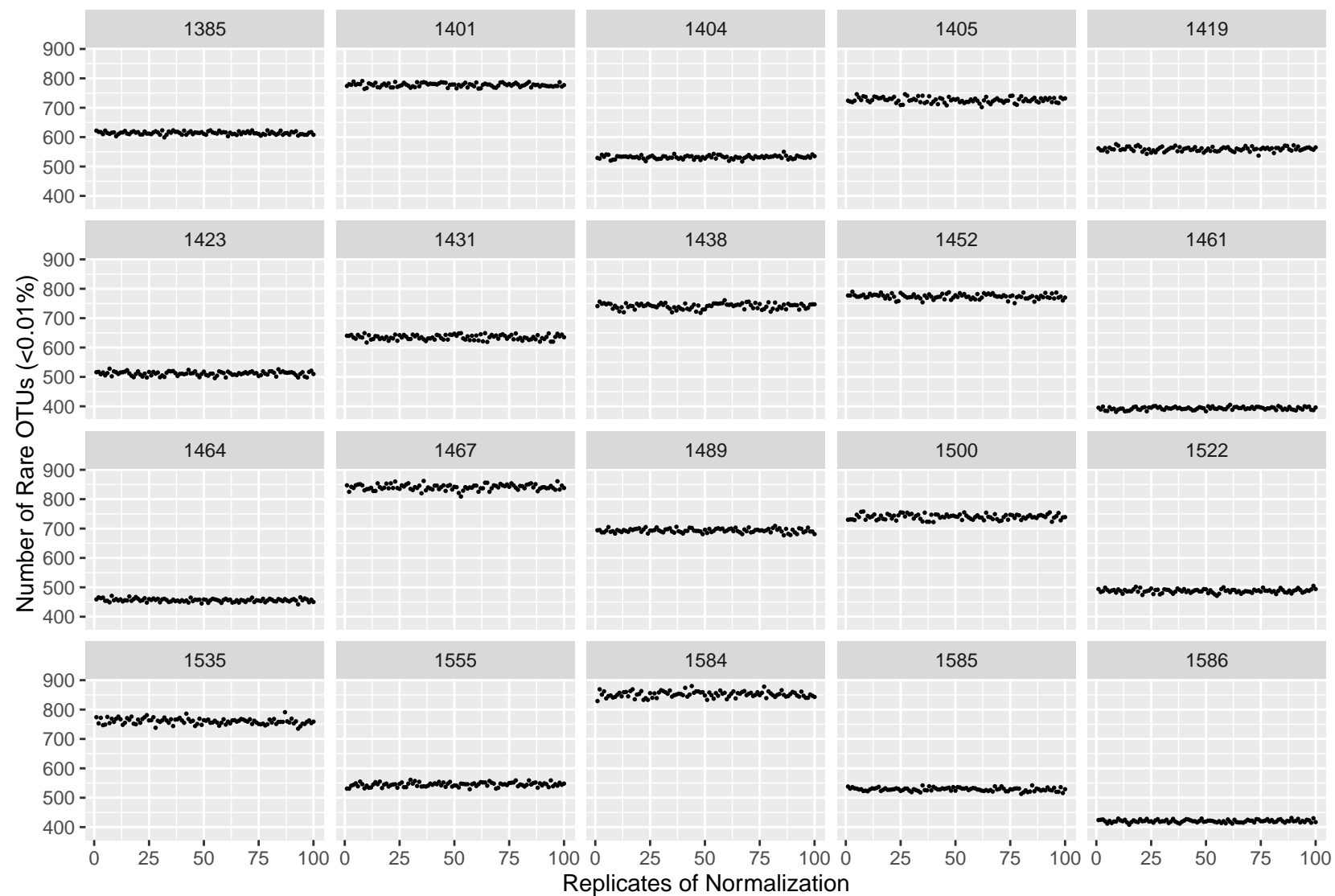

Supplement: S1 File — 100 replicates of the normalization step were done on each of the 200 randomly selected samples (10% of the samples). For each replicate, high-quality-reads were clustered, and obtained OTUs analyzed to determine the impact of the normalization step on OTUs. Four groups of OTUs were considered: Major (composed of more than 1% of reads), Medium (1–0.1% of reads), Low (0.01–0.1% of reads) and Rare (less than 0.01% of reads), showing no impact of the normalization step. (ZIP) [file pone.0186766.s007.zip › Rare_Img_7.pdf]

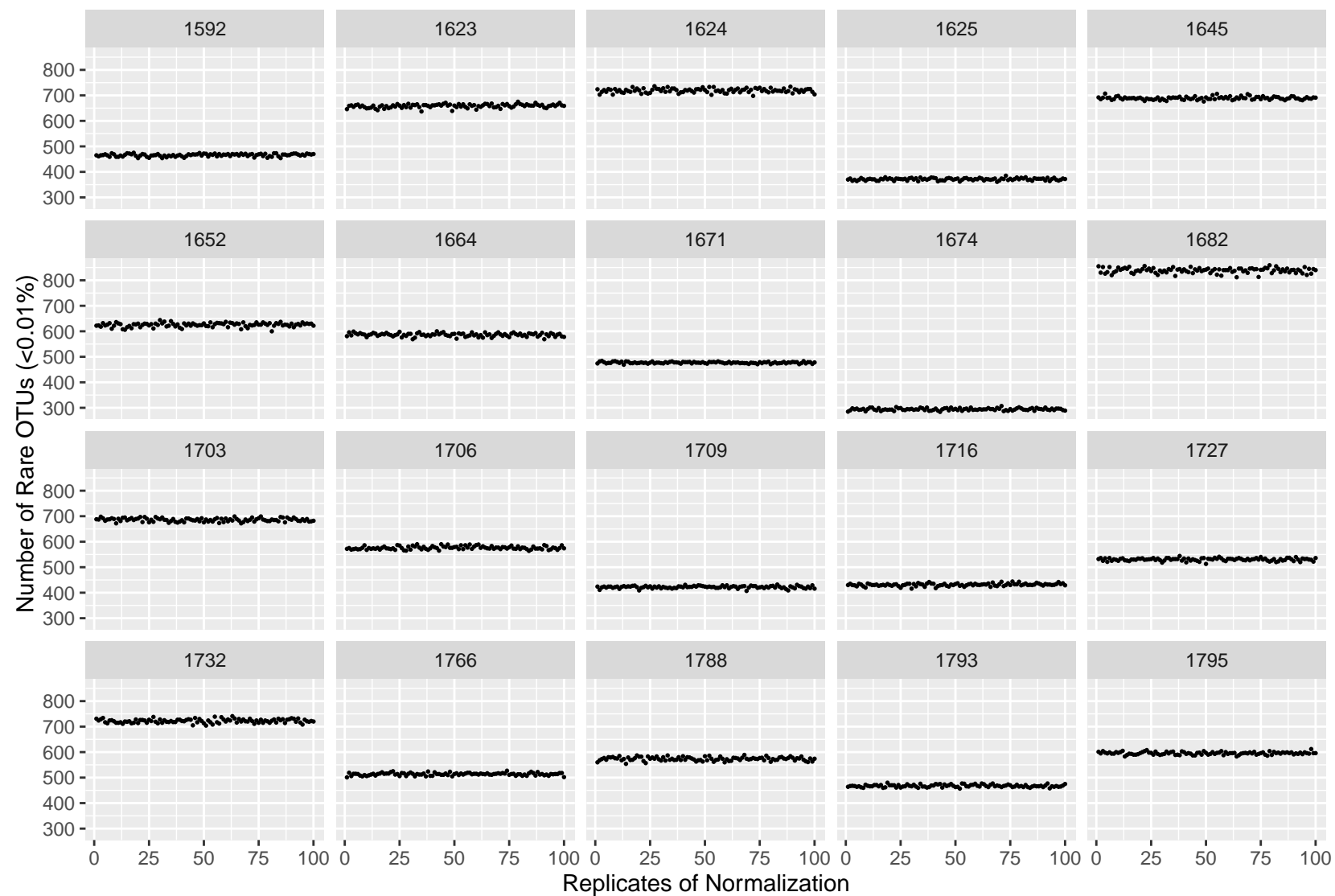

Supplement: S1 File — 100 replicates of the normalization step were done on each of the 200 randomly selected samples (10% of the samples). For each replicate, high-quality-reads were clustered, and obtained OTUs analyzed to determine the impact of the normalization step on OTUs. Four groups of OTUs were considered: Major (composed of more than 1% of reads), Medium (1–0.1% of reads), Low (0.01–0.1% of reads) and Rare (less than 0.01% of reads), showing no impact of the normalization step. (ZIP) [file pone.0186766.s007.zip › Rare_Img_8.pdf]

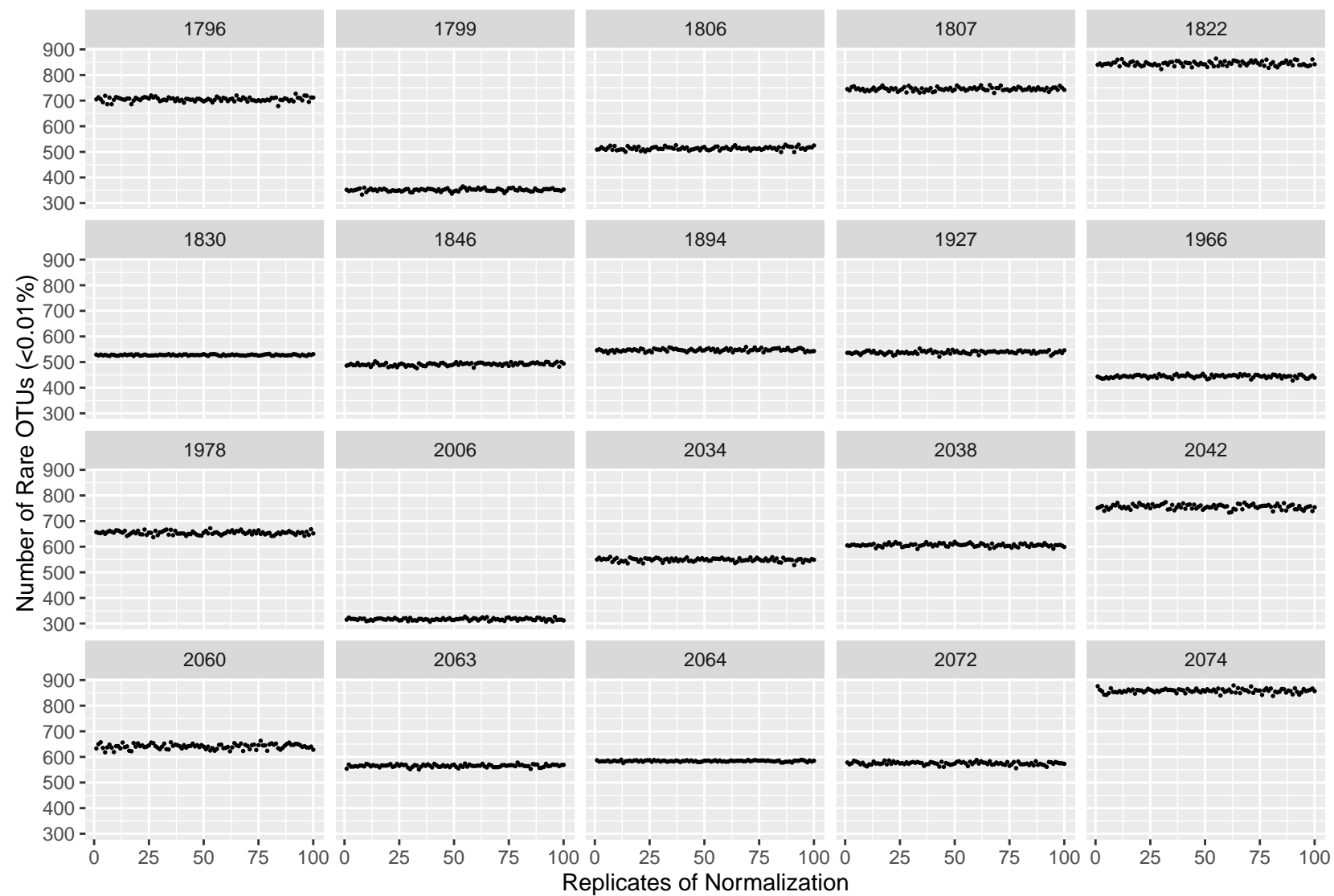

Supplement: S1 File — 100 replicates of the normalization step were done on each of the 200 randomly selected samples (10% of the samples). For each replicate, high-quality-reads were clustered, and obtained OTUs analyzed to determine the impact of the normalization step on OTUs. Four groups of OTUs were considered: Major (composed of more than 1% of reads), Medium (1–0.1% of reads), Low (0.01–0.1% of reads) and Rare (less than 0.01% of reads), showing no impact of the normalization step. (ZIP) [file pone.0186766.s007.zip › Rare_Img_9.pdf]

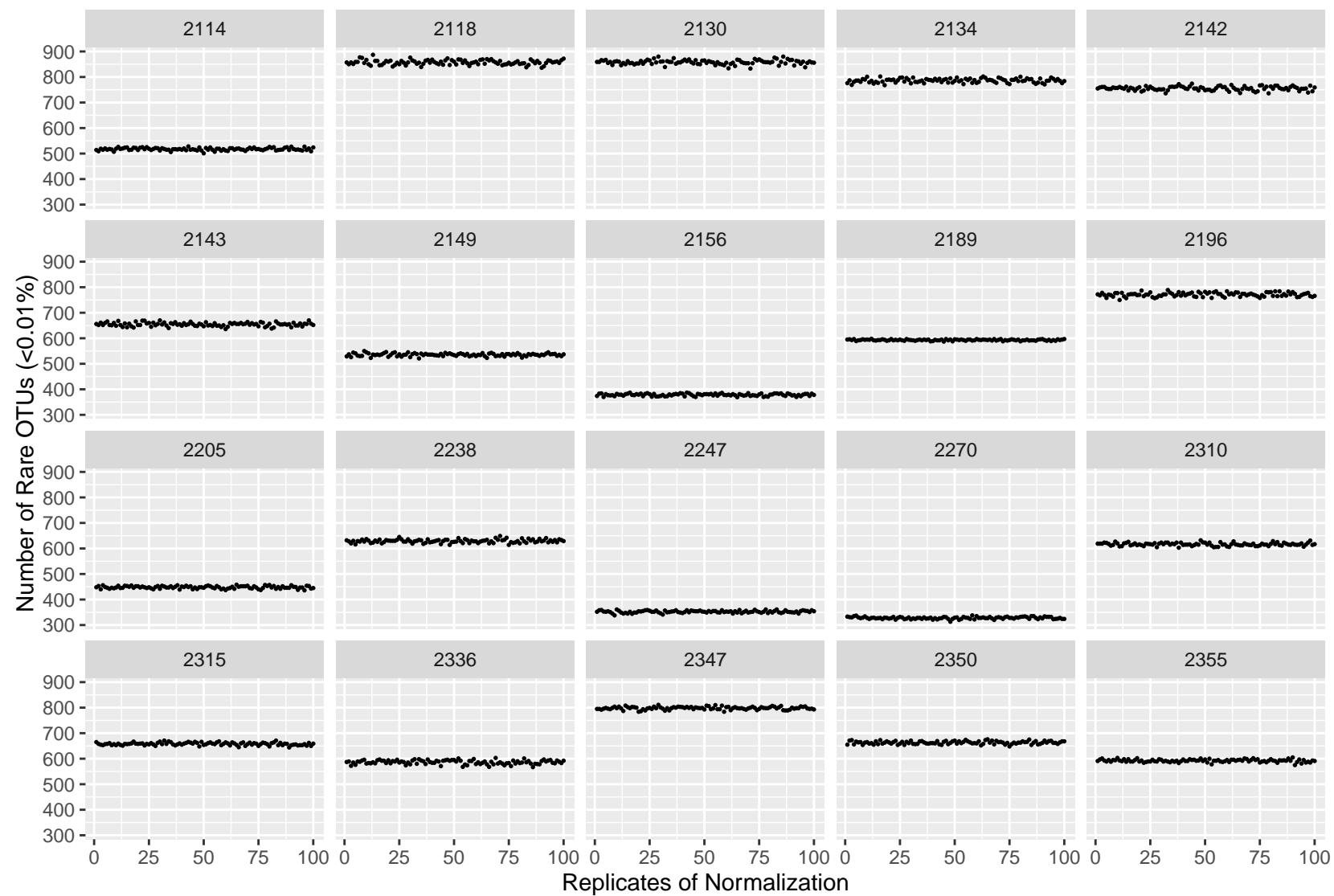

Supplement: S1 File — 100 replicates of the normalization step were done on each of the 200 randomly selected samples (10% of the samples). For each replicate, high-quality-reads were clustered, and obtained OTUs analyzed to determine the impact of the normalization step on OTUs. Four groups of OTUs were considered: Major (composed of more than 1% of reads), Medium (1–0.1% of reads), Low (0.01–0.1% of reads) and Rare (less than 0.01% of reads), showing no impact of the normalization step. (ZIP) [file pone.0186766.s007.zip › Rare_Img_10.pdf]

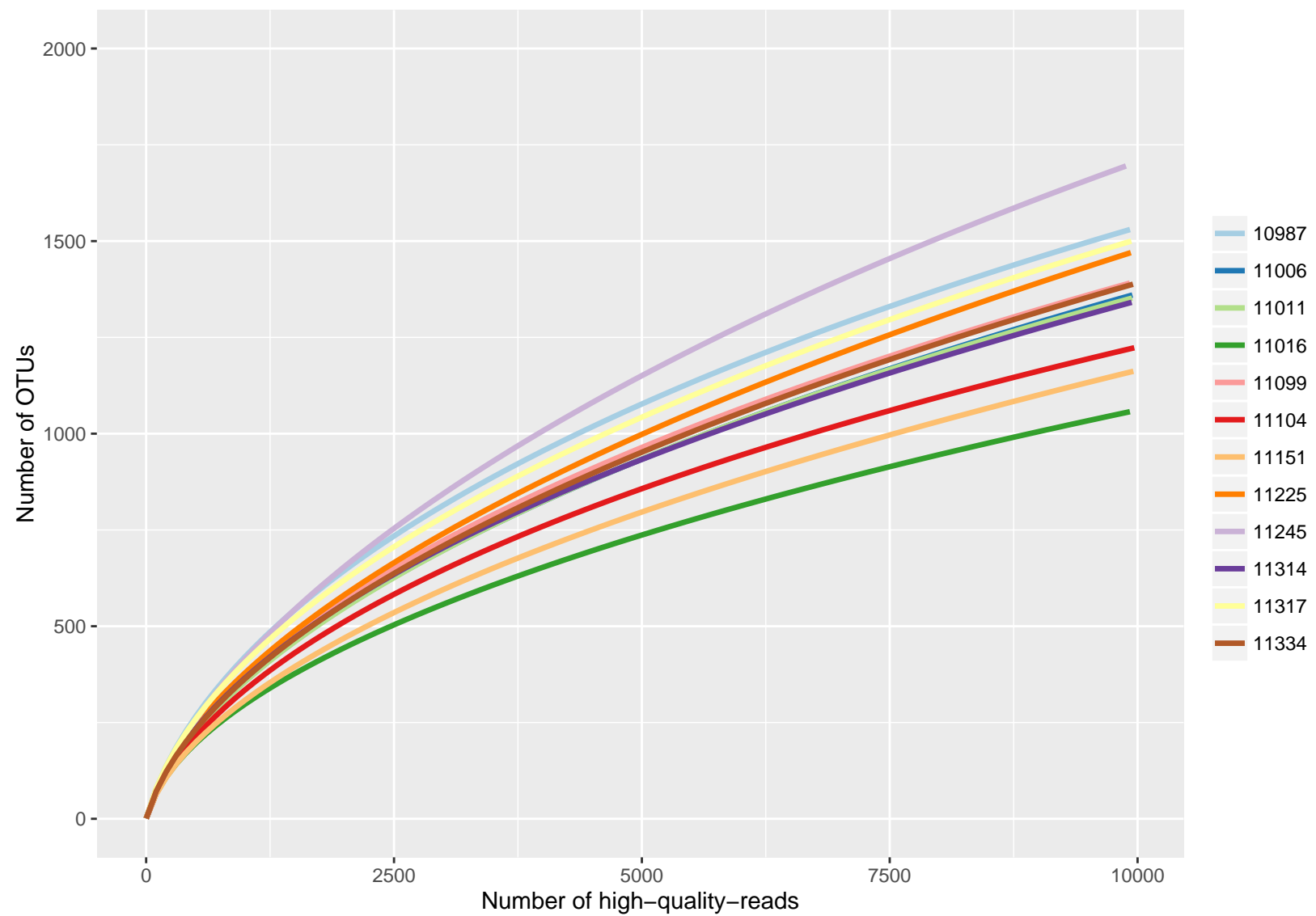

Supplement: S2 File — (ZIP) [file pone.0186766.s008.zip › Rarefact_curves_149.pdf]

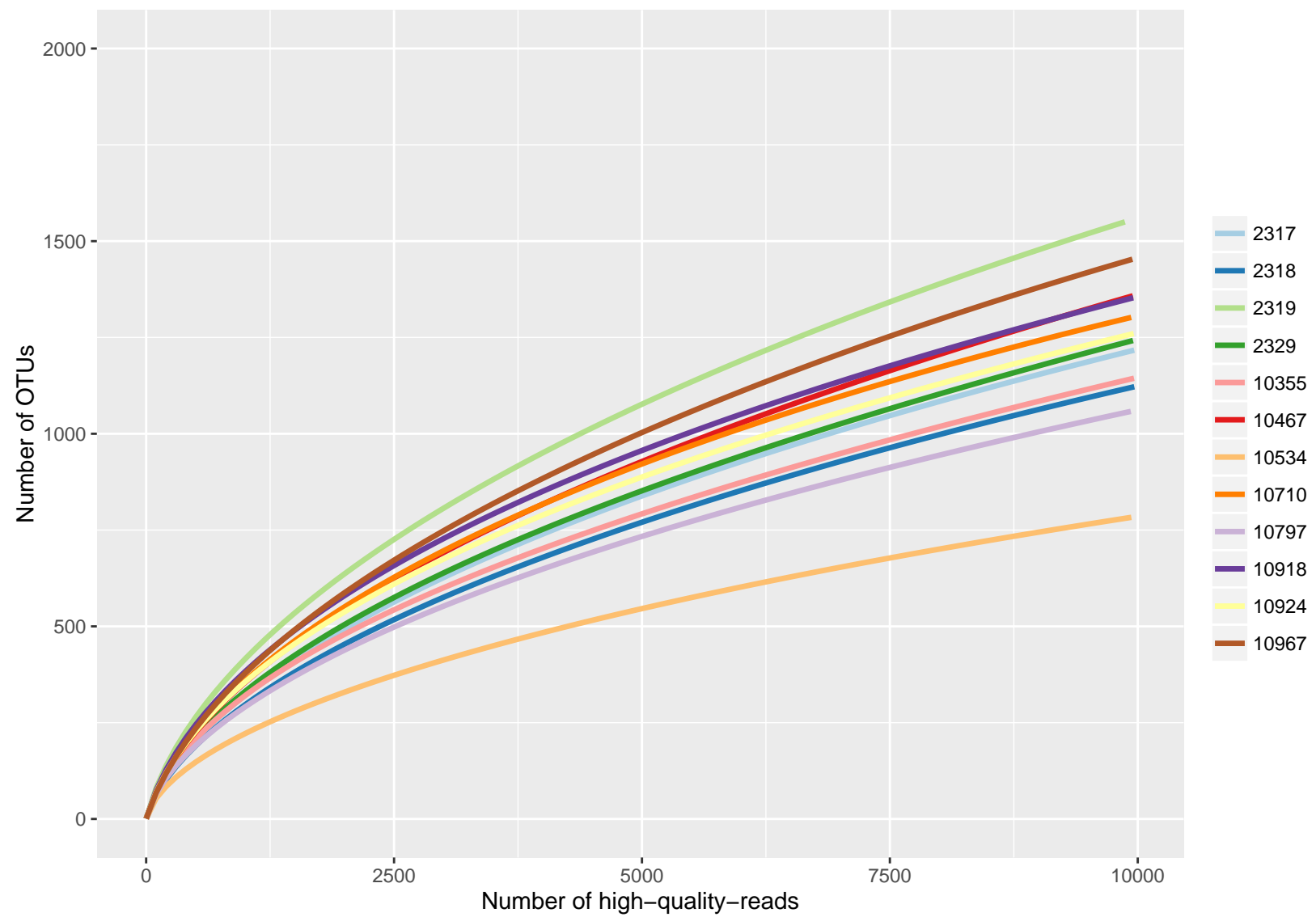

Supplement: S2 File — (ZIP) [file pone.0186766.s008.zip › Rarefact_curves_148.pdf]

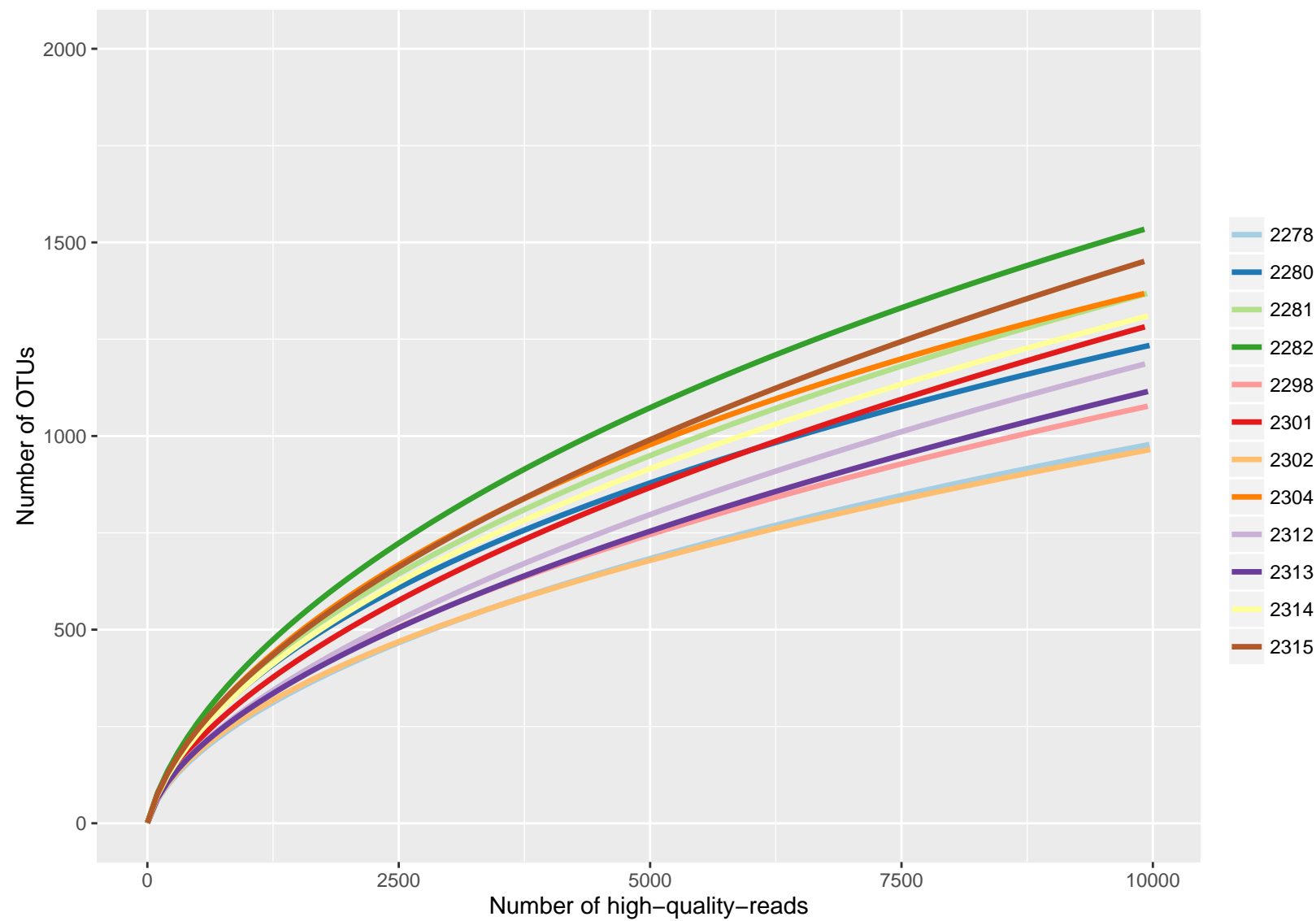

Supplement: S2 File — (ZIP) [file pone.0186766.s008.zip › Rarefact_curves_147.pdf]

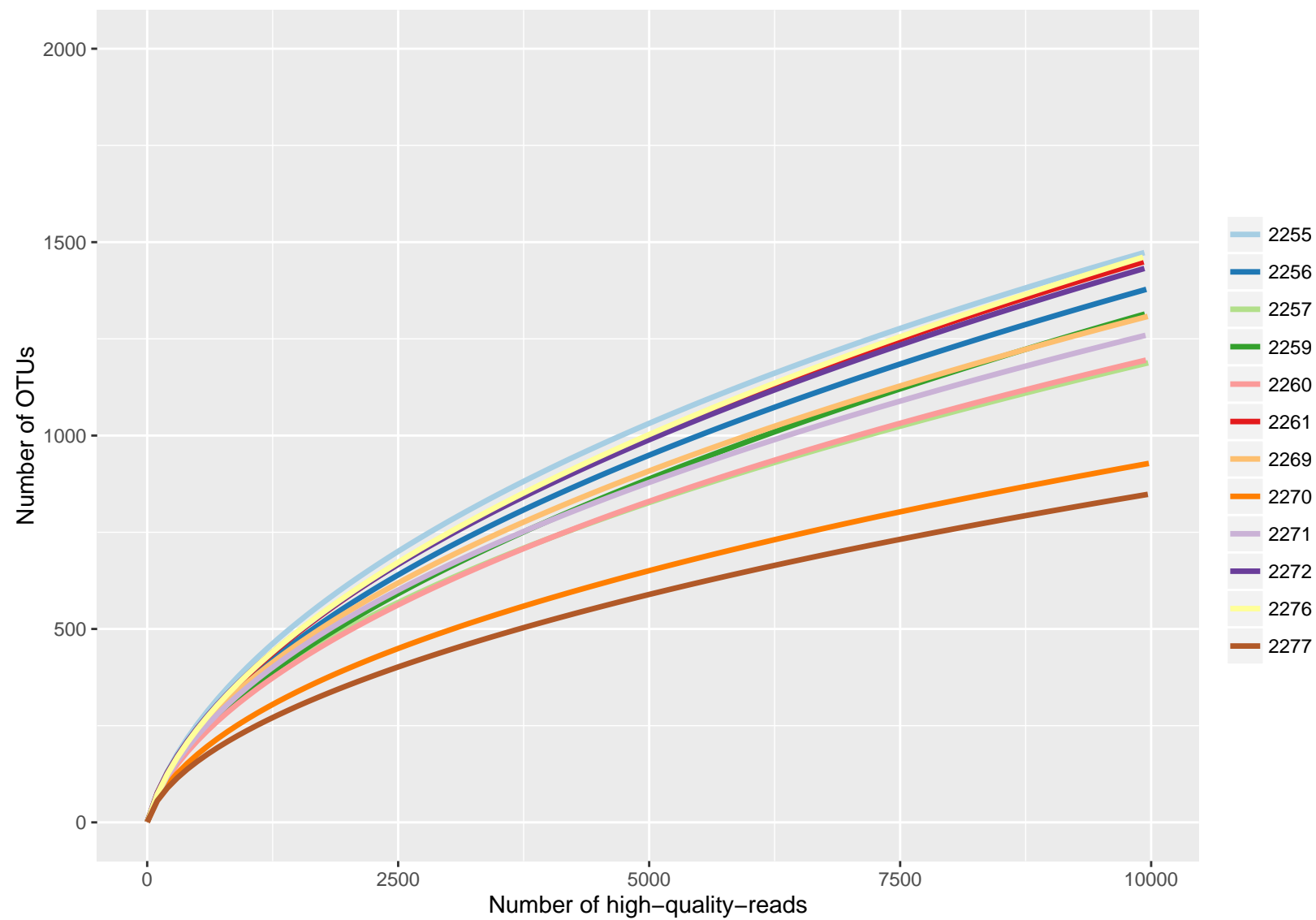

Supplement: S2 File — (ZIP) [file pone.0186766.s008.zip › Rarefact_curves_146.pdf]

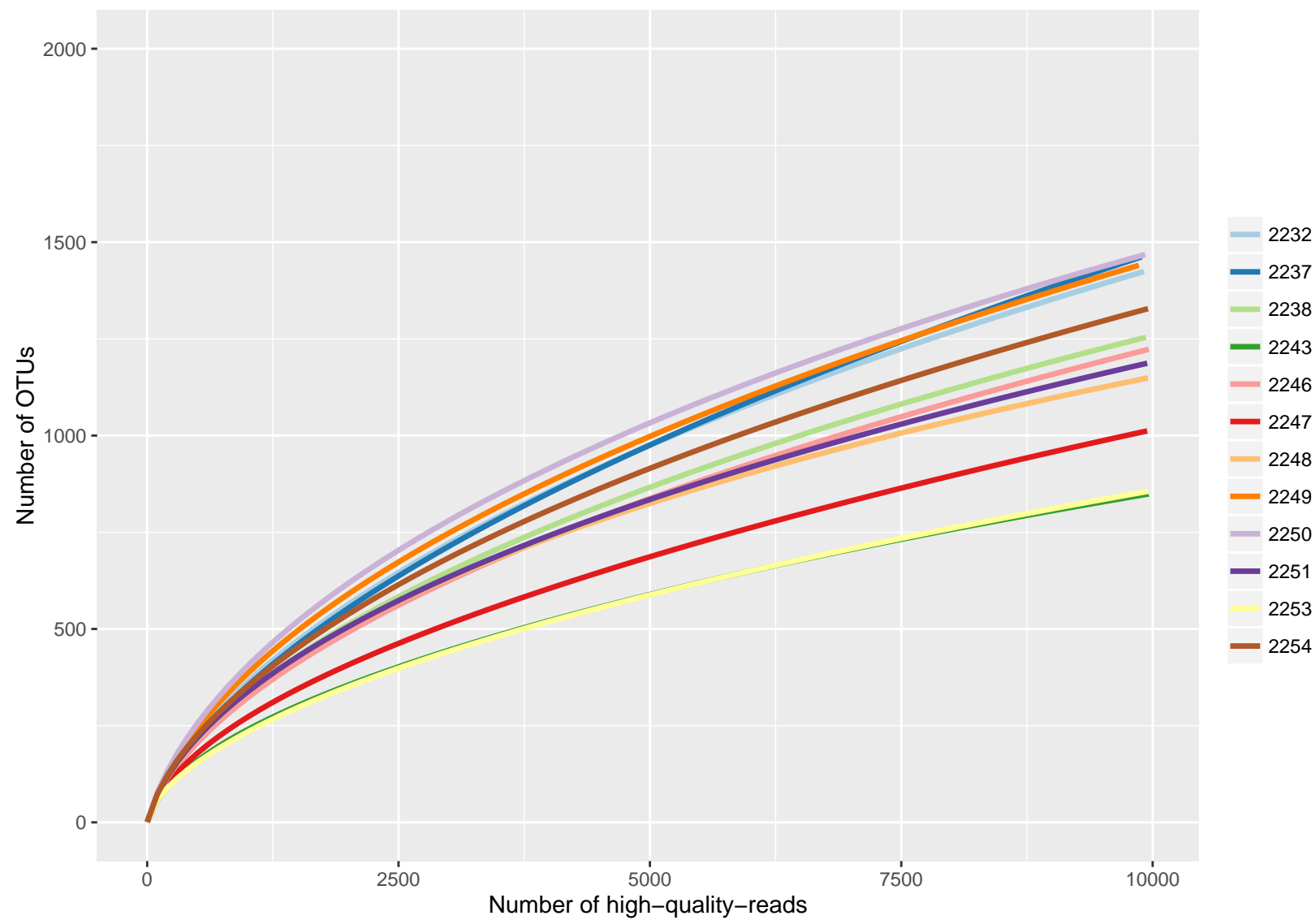

Supplement: S2 File — (ZIP) [file pone.0186766.s008.zip › Rarefact_curves_145.pdf]

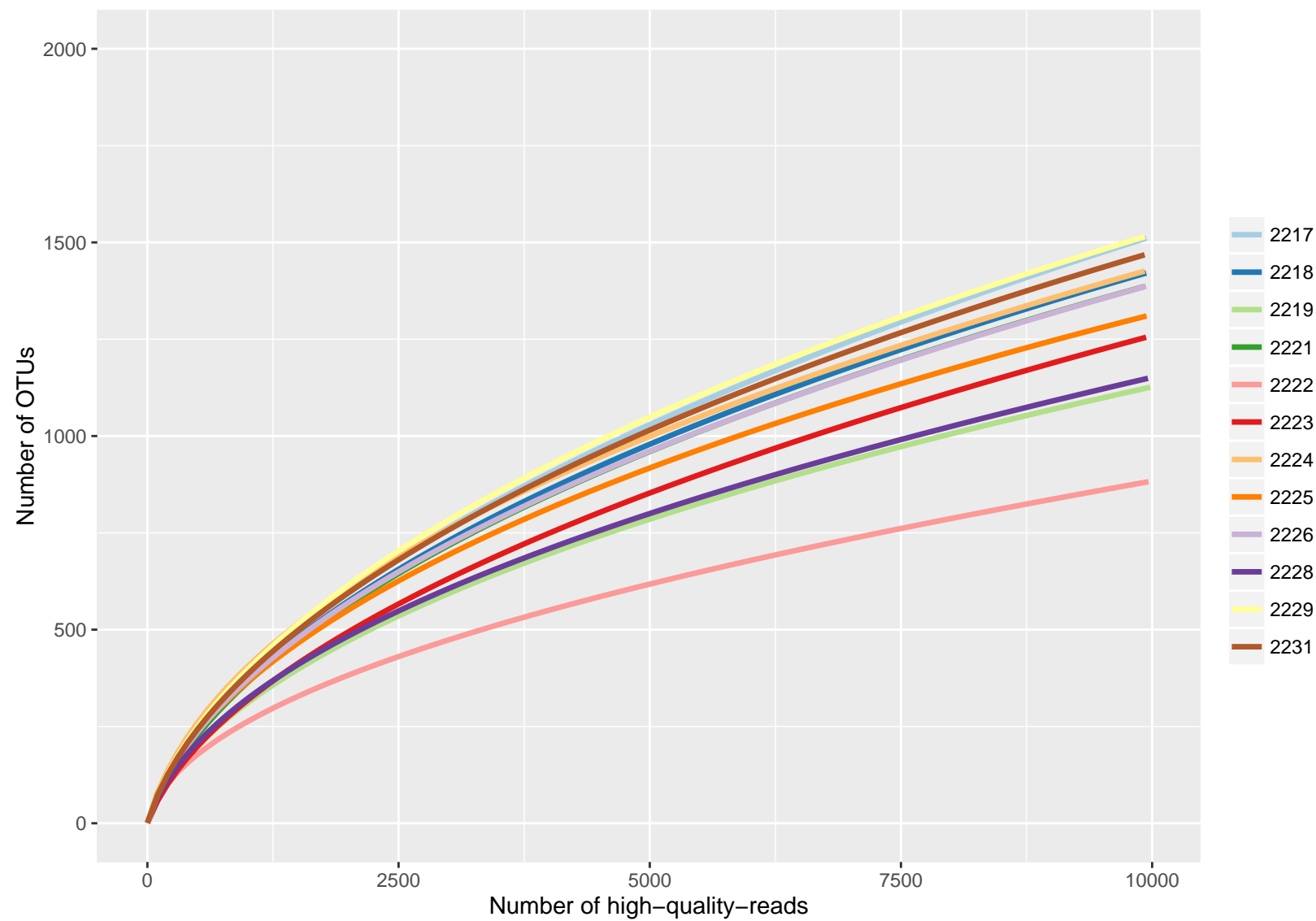

Supplement: S2 File — (ZIP) [file pone.0186766.s008.zip › Rarefact_curves_144.pdf]

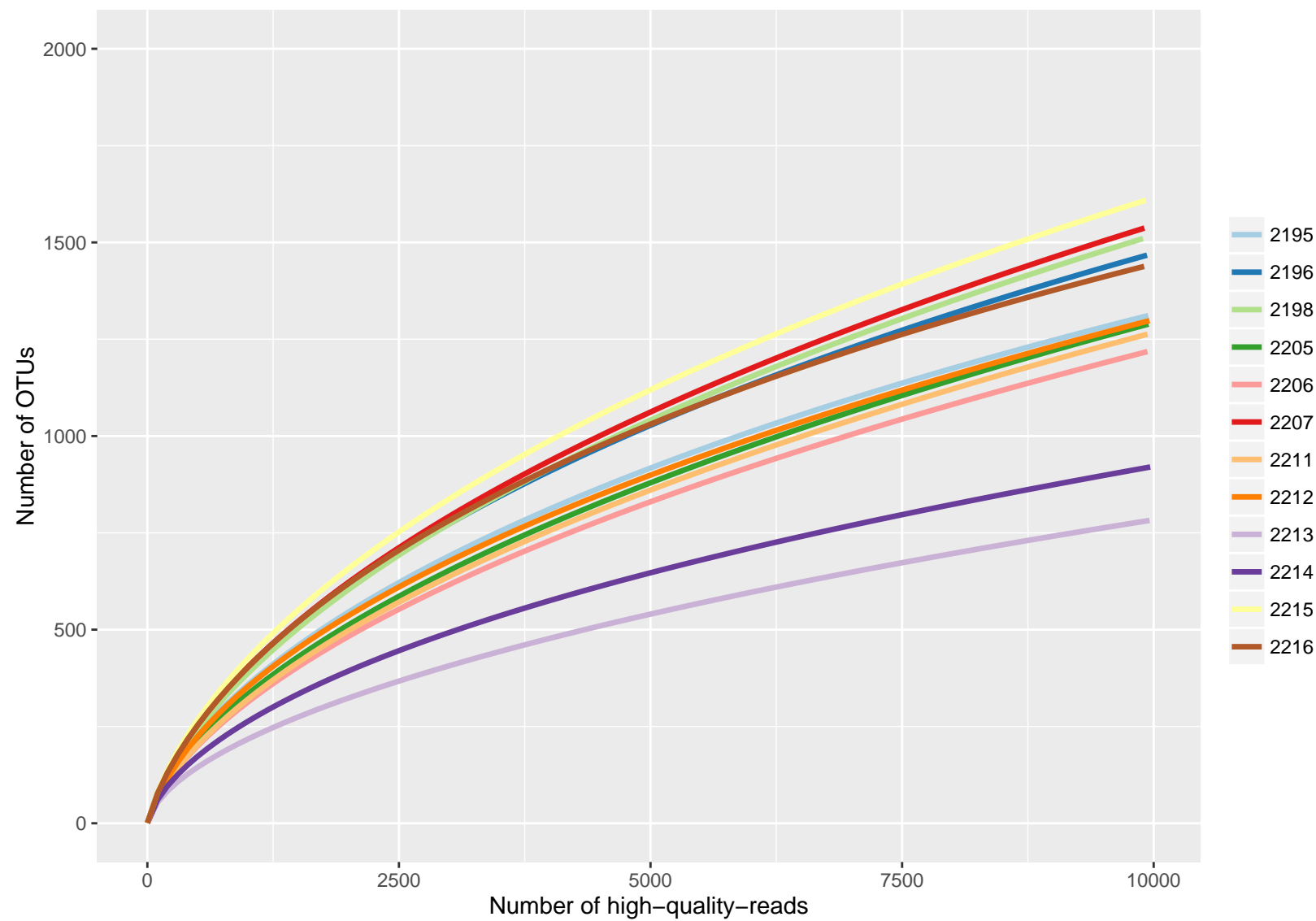

Supplement: S2 File — (ZIP) [file pone.0186766.s008.zip › Rarefact_curves_143.pdf]

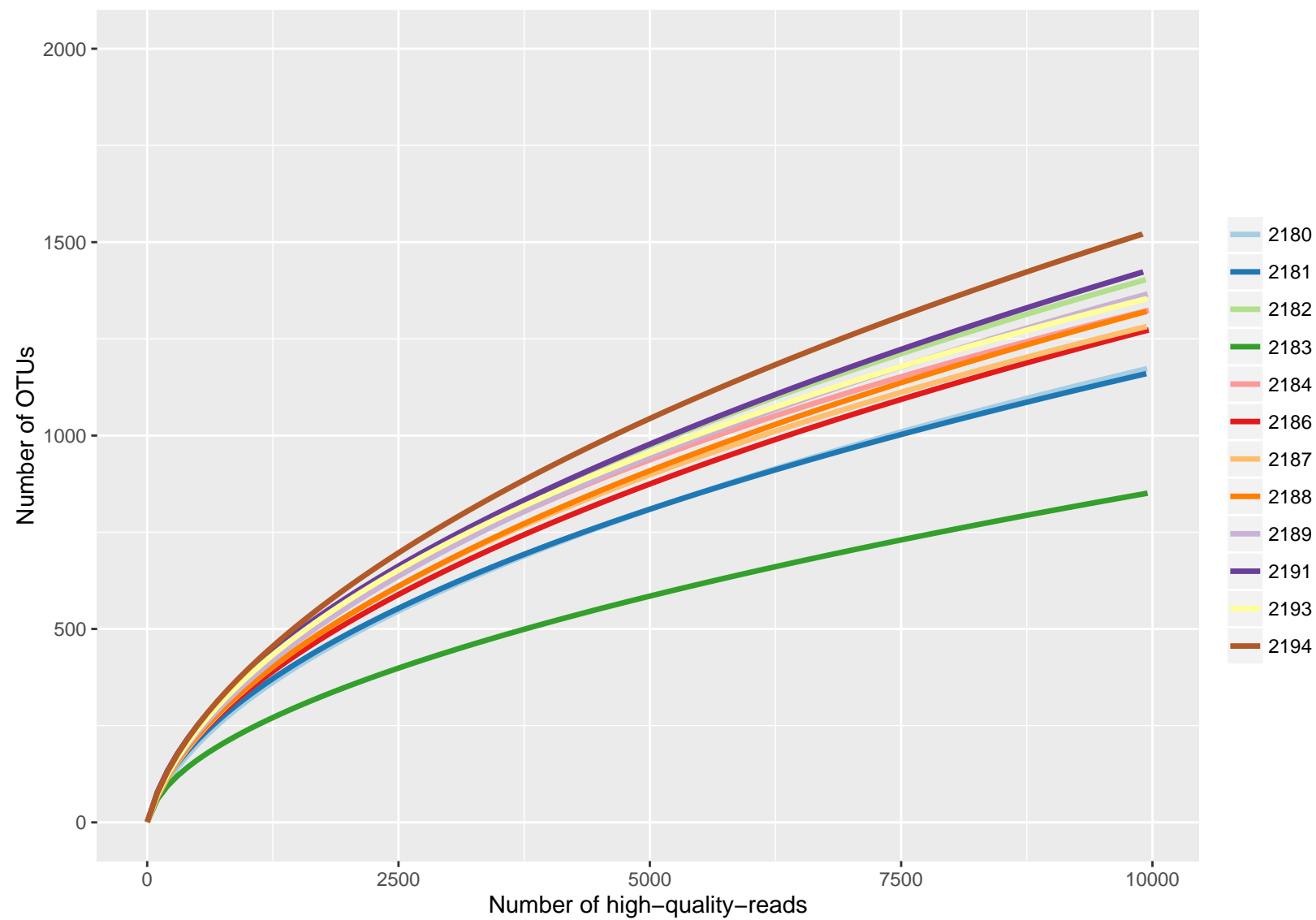

Supplement: S2 File — (ZIP) [file pone.0186766.s008.zip › Rarefact_curves_142.pdf]

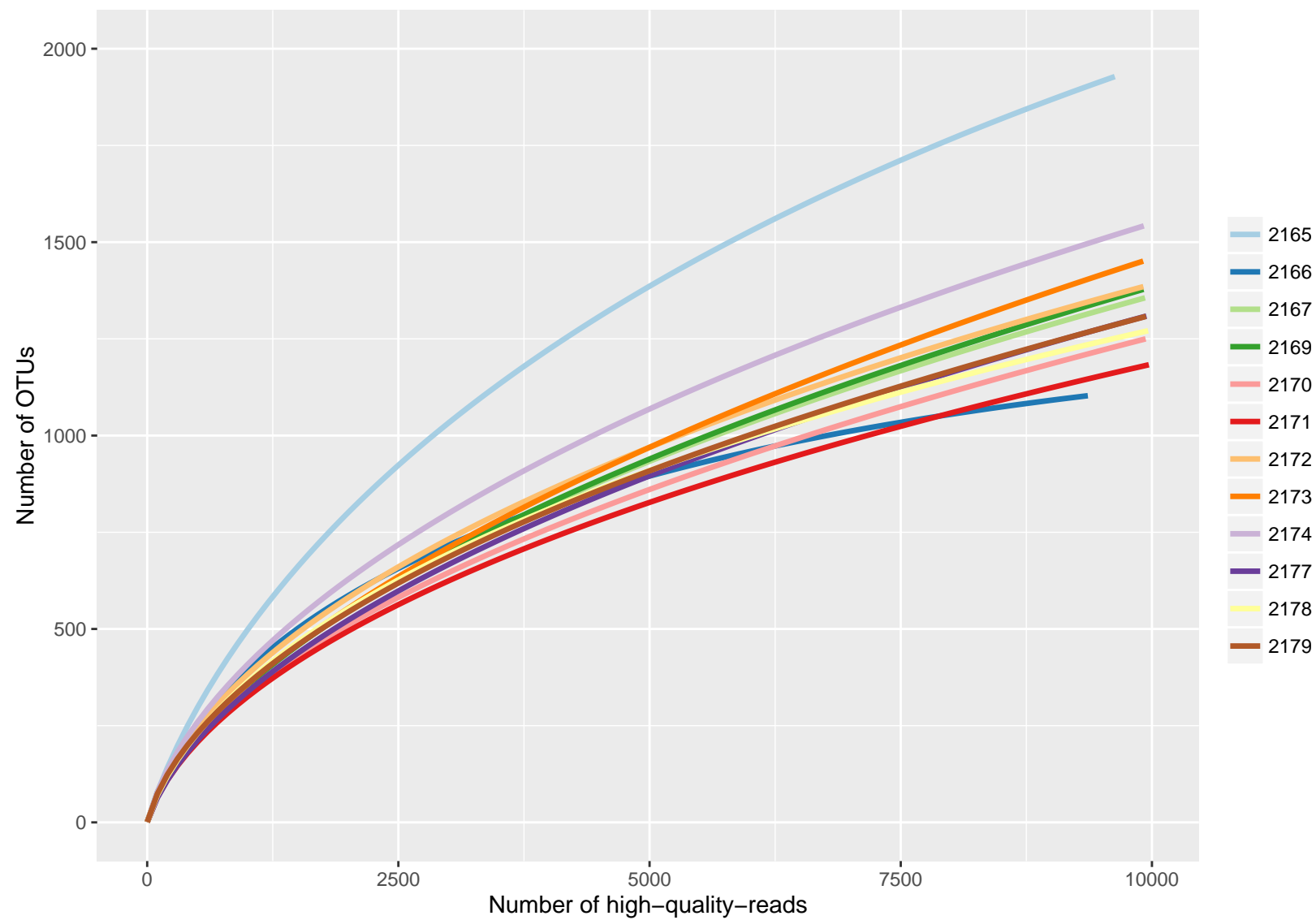

Supplement: S2 File — (ZIP) [file pone.0186766.s008.zip › Rarefact_curves_141.pdf]

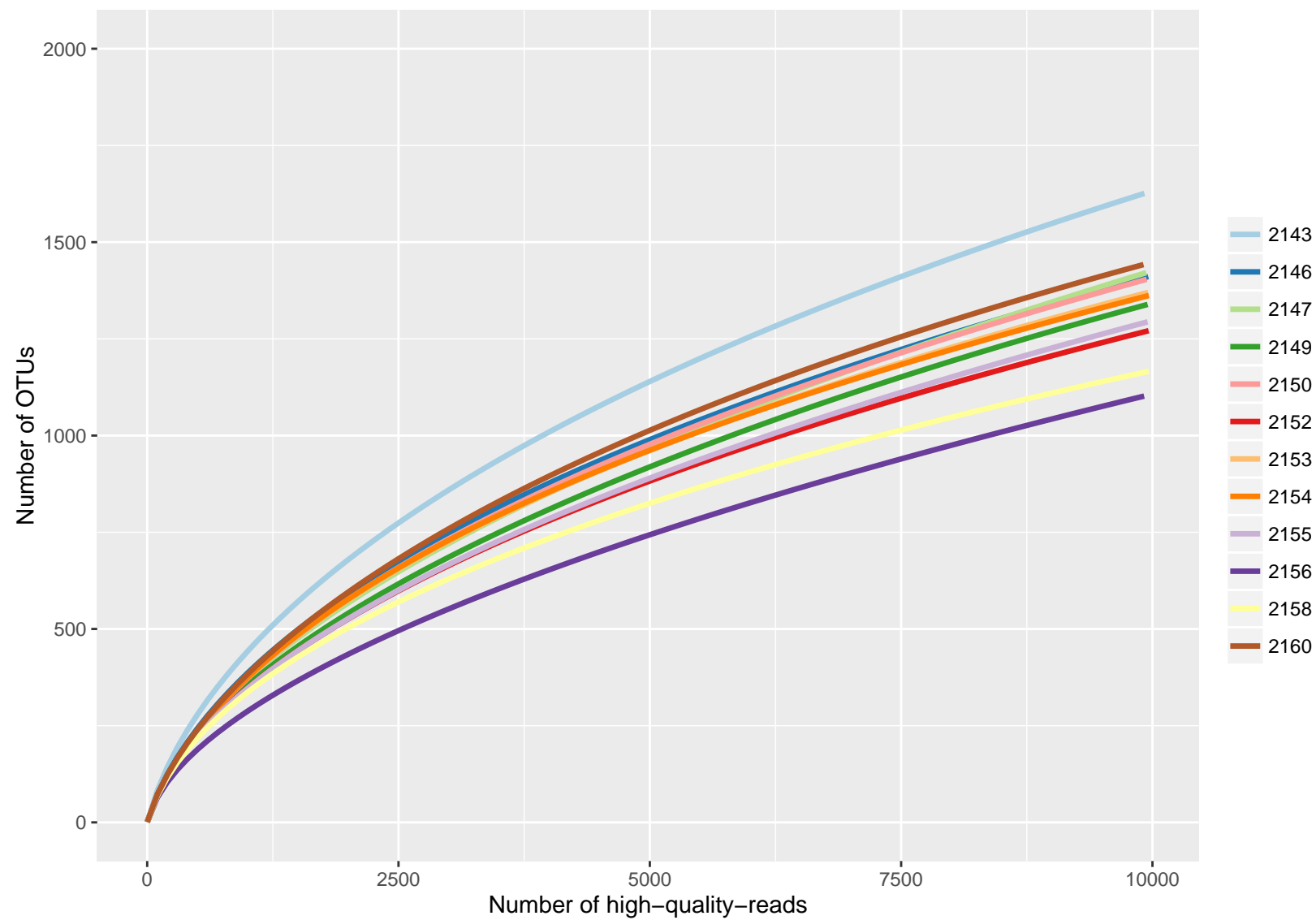

Supplement: S2 File — (ZIP) [file pone.0186766.s008.zip › Rarefact_curves_140.pdf]

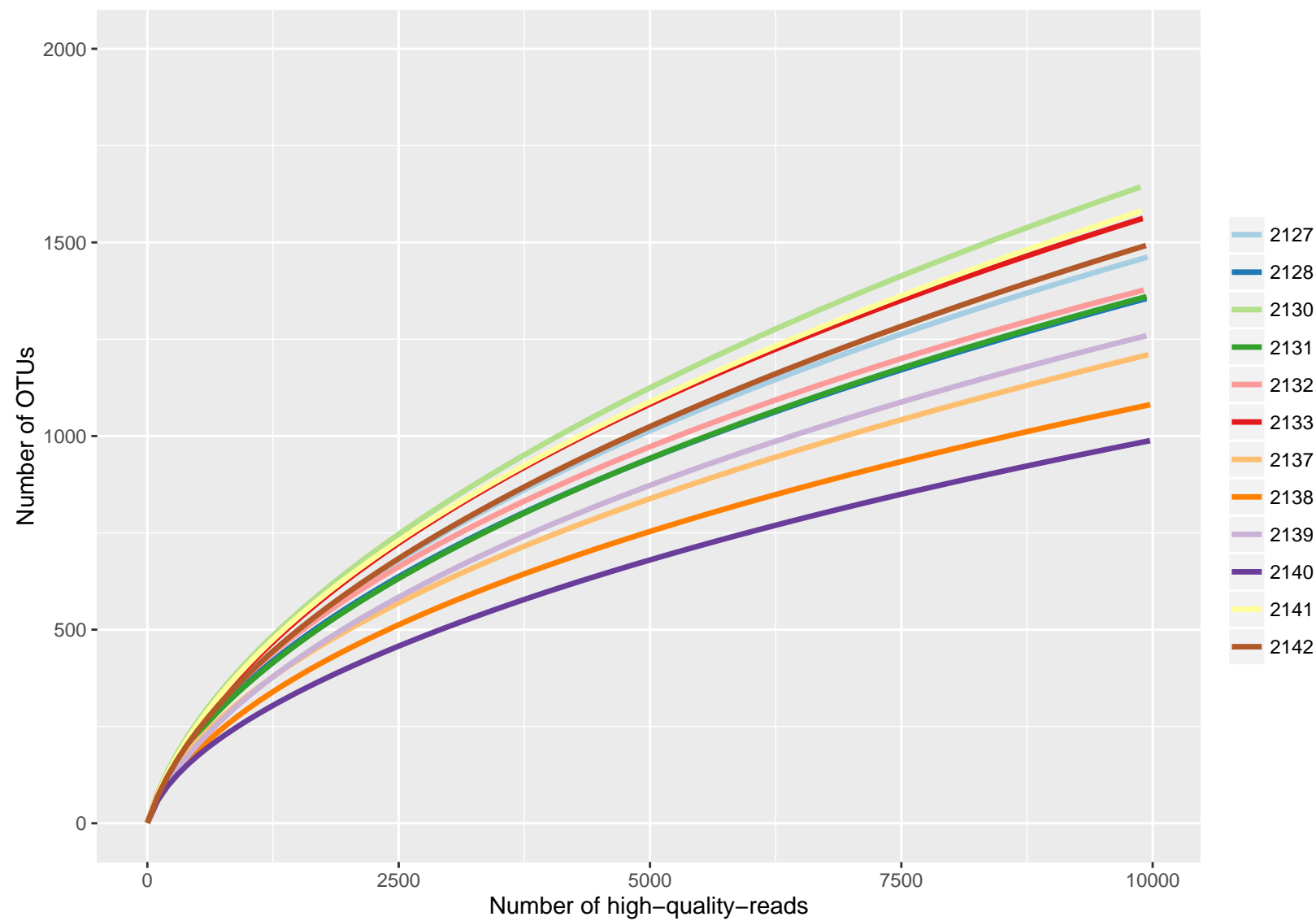

Supplement: S2 File — (ZIP) [file pone.0186766.s008.zip › Rarefact_curves_139.pdf]

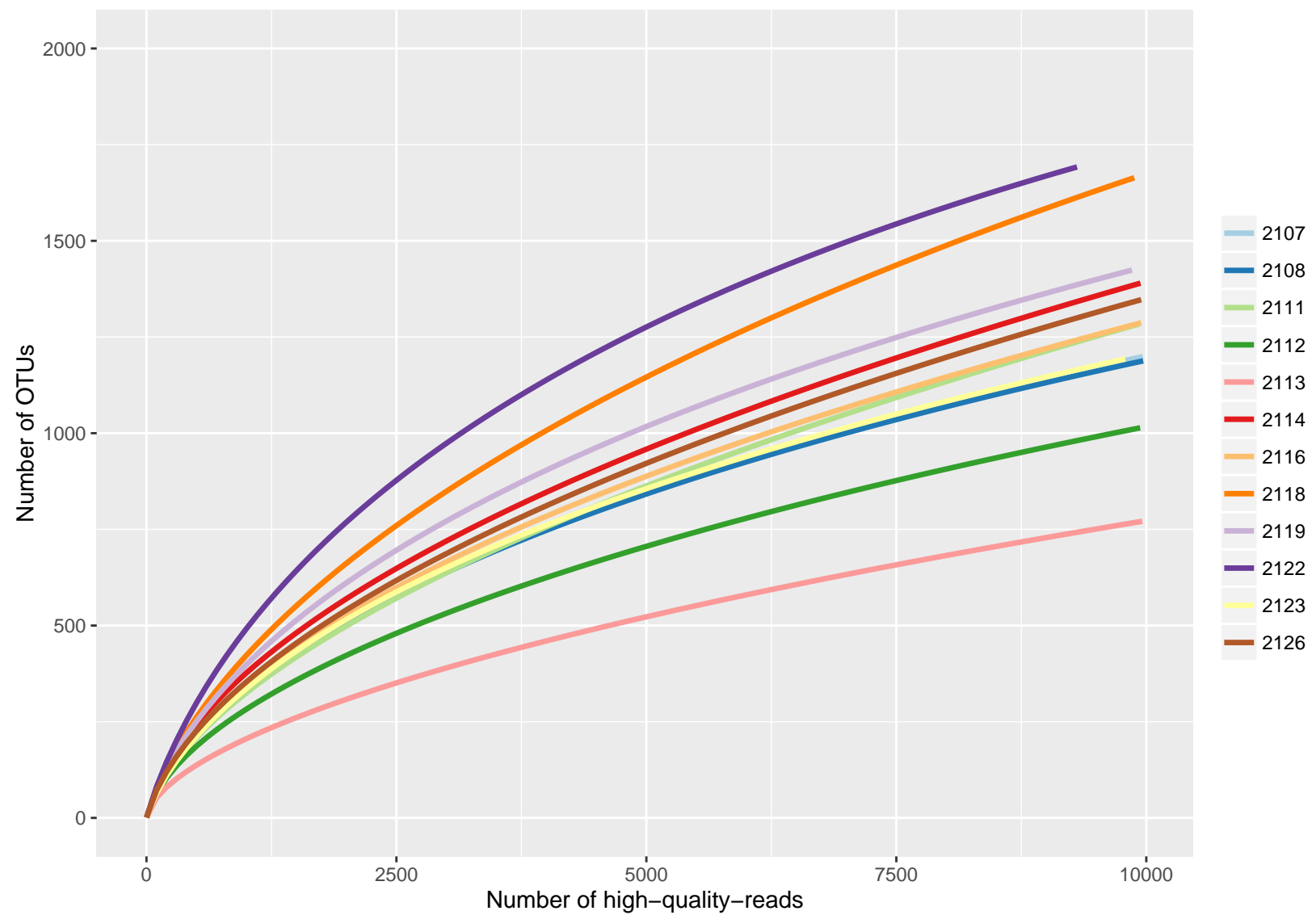

Supplement: S2 File — (ZIP) [file pone.0186766.s008.zip › Rarefact_curves_138.pdf]

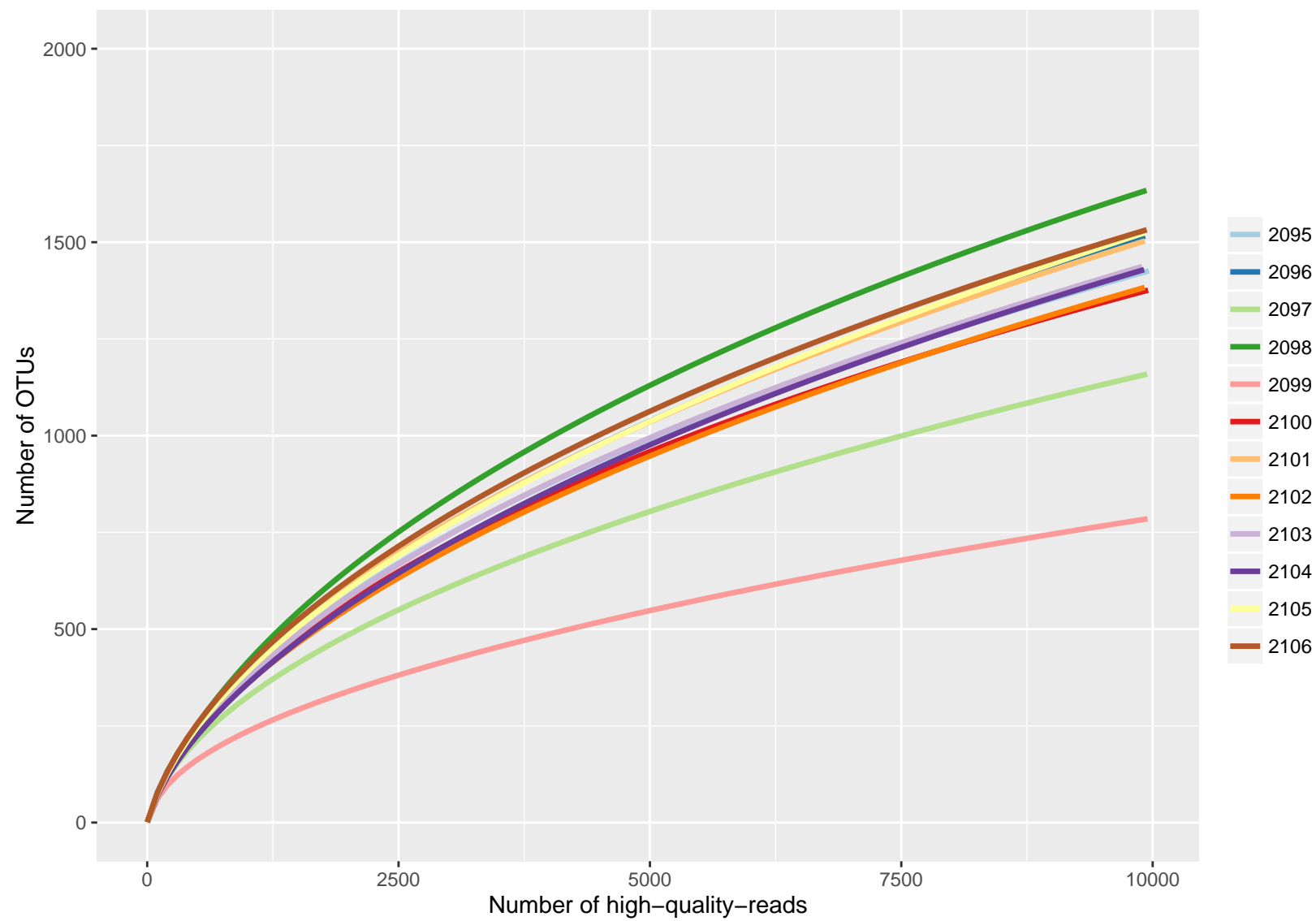

Supplement: S2 File — (ZIP) [file pone.0186766.s008.zip › Rarefact_curves_137.pdf]

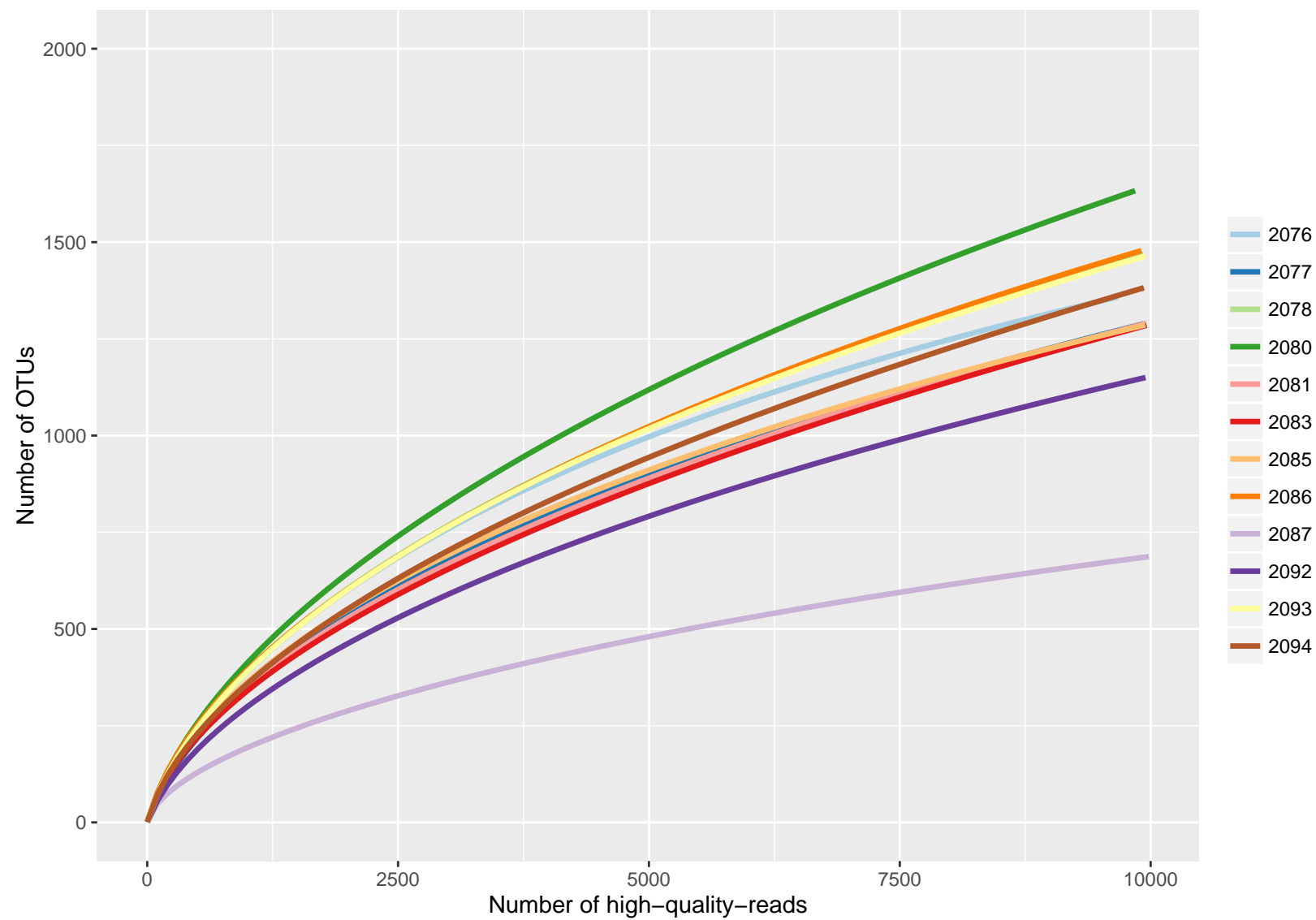

Supplement: S2 File — (ZIP) [file pone.0186766.s008.zip › Rarefact_curves_136.pdf]

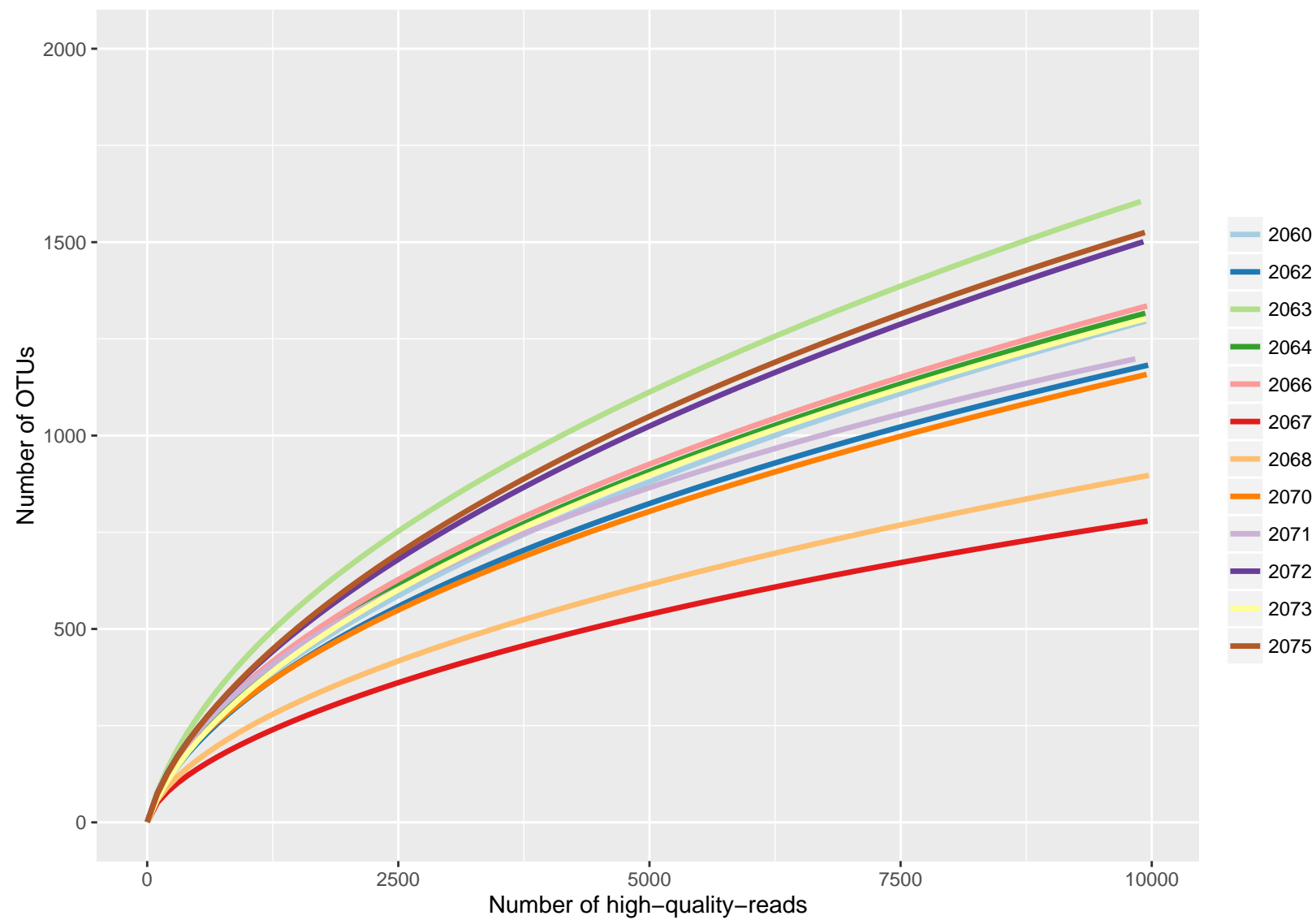

Supplement: S2 File — (ZIP) [file pone.0186766.s008.zip › Rarefact_curves_135.pdf]

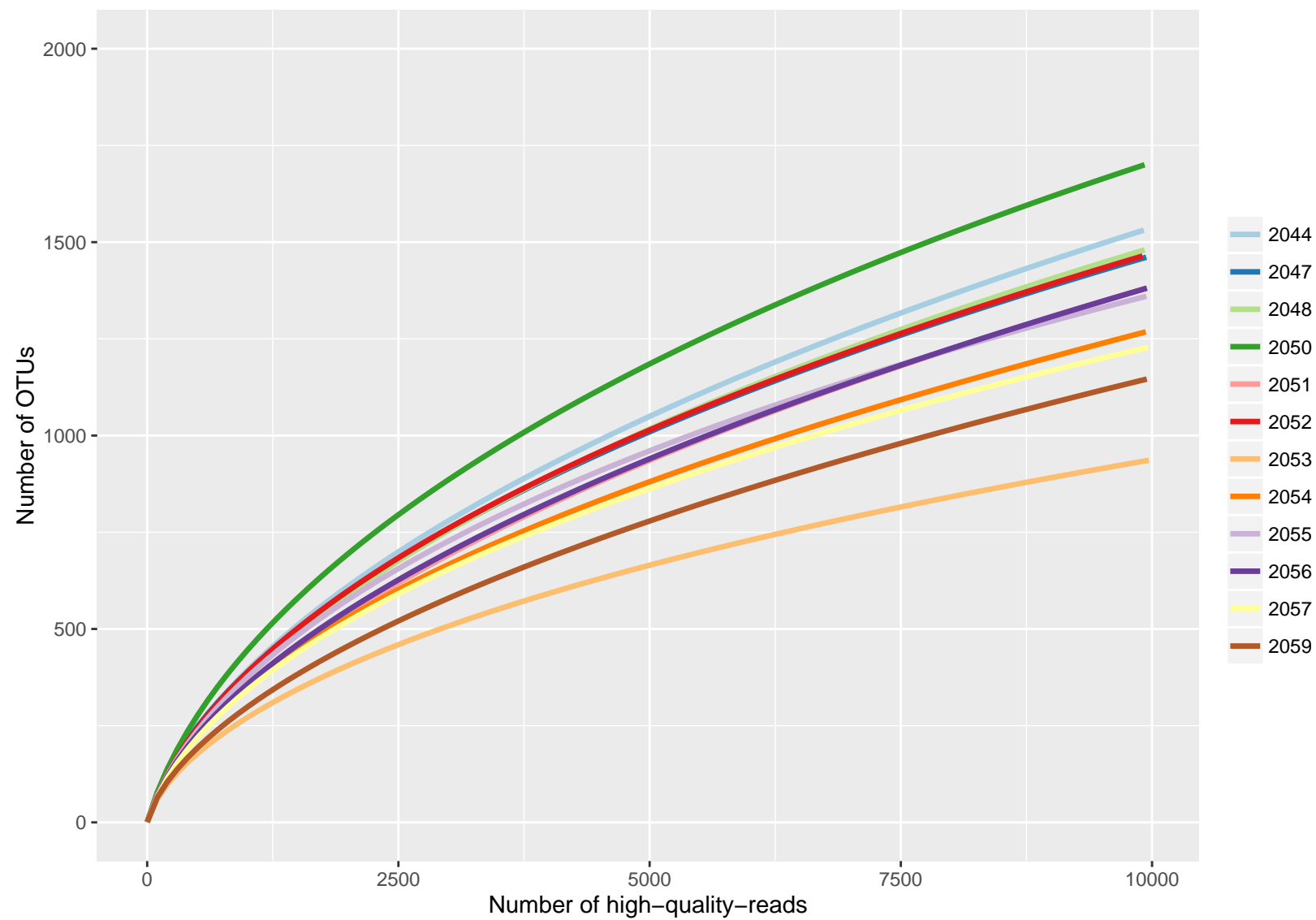

Supplement: S2 File — (ZIP) [file pone.0186766.s008.zip › Rarefact_curves_134.pdf]

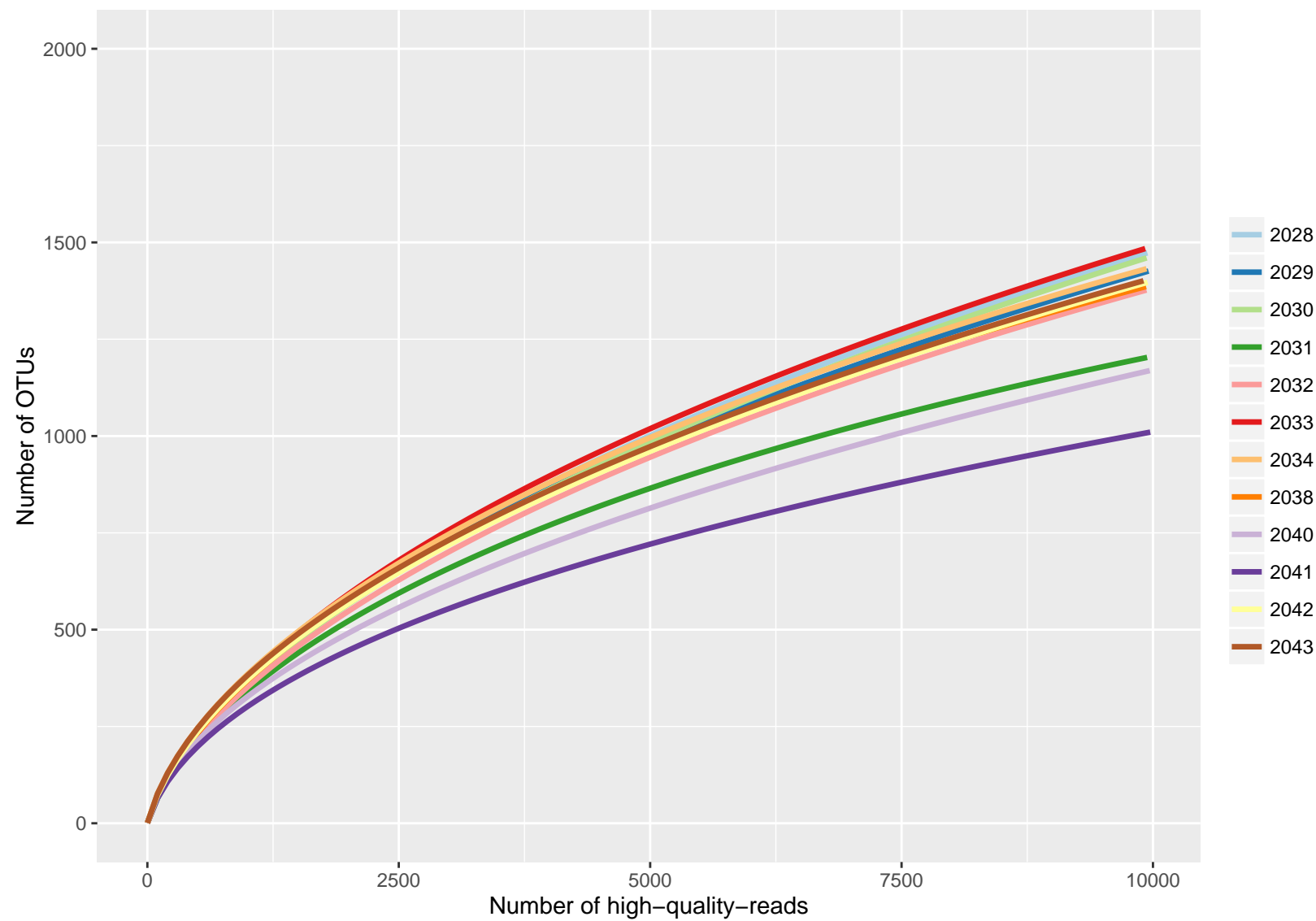

Supplement: S2 File — (ZIP) [file pone.0186766.s008.zip › Rarefact_curves_133.pdf]

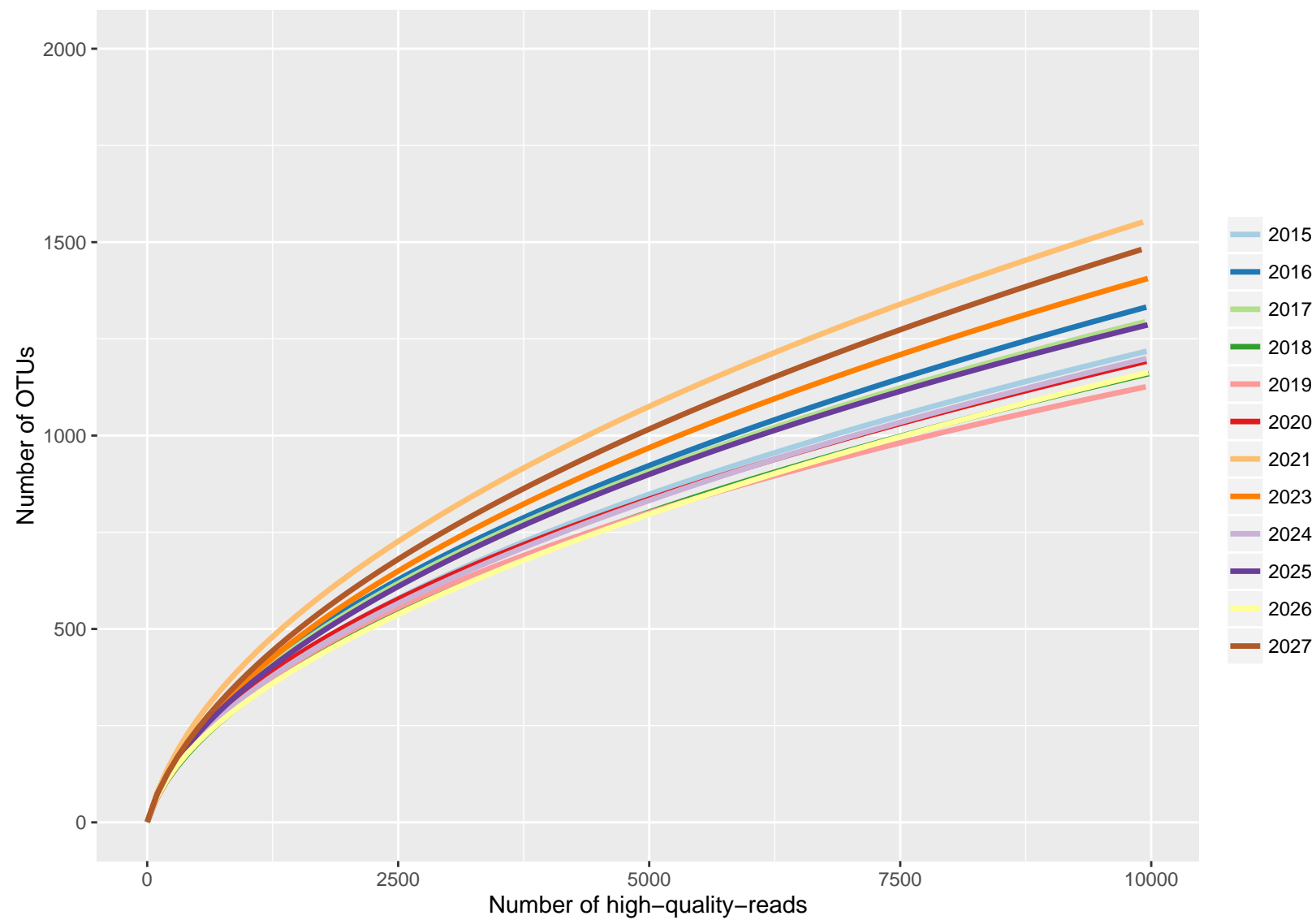

Supplement: S2 File — (ZIP) [file pone.0186766.s008.zip › Rarefact_curves_132.pdf]

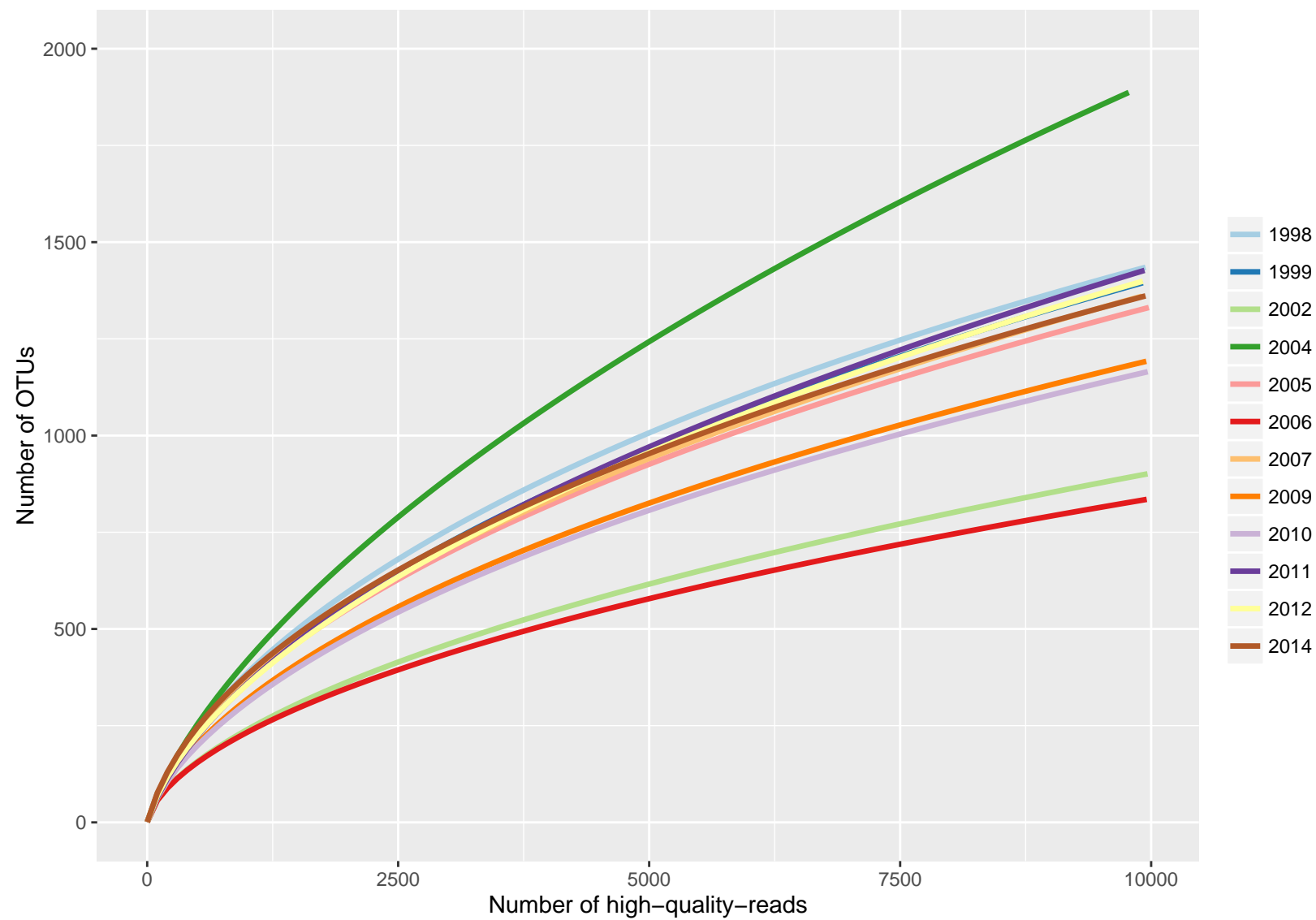

Supplement: S2 File — (ZIP) [file pone.0186766.s008.zip › Rarefact_curves_131.pdf]

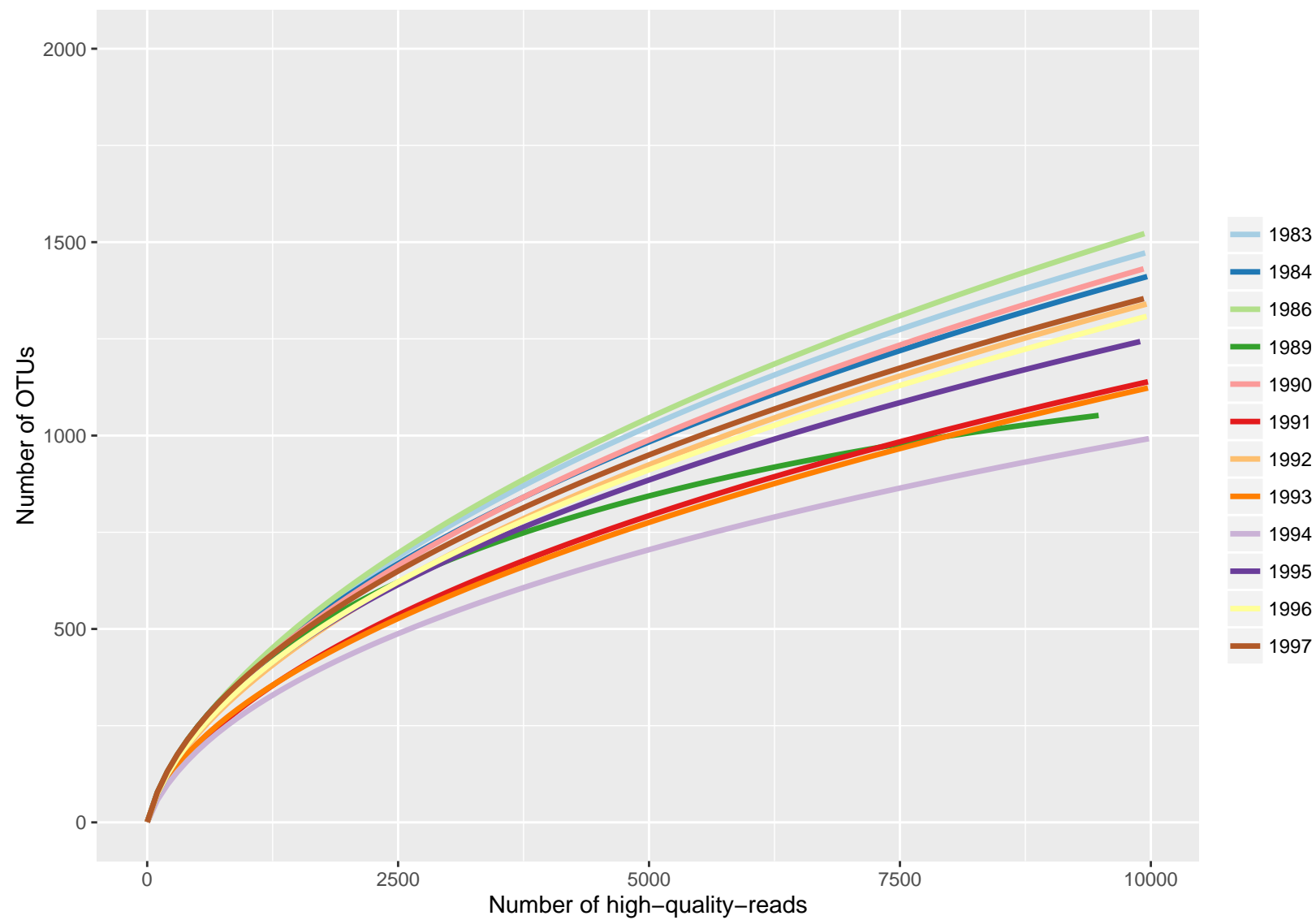

Supplement: S2 File — (ZIP) [file pone.0186766.s008.zip › Rarefact_curves_130.pdf]

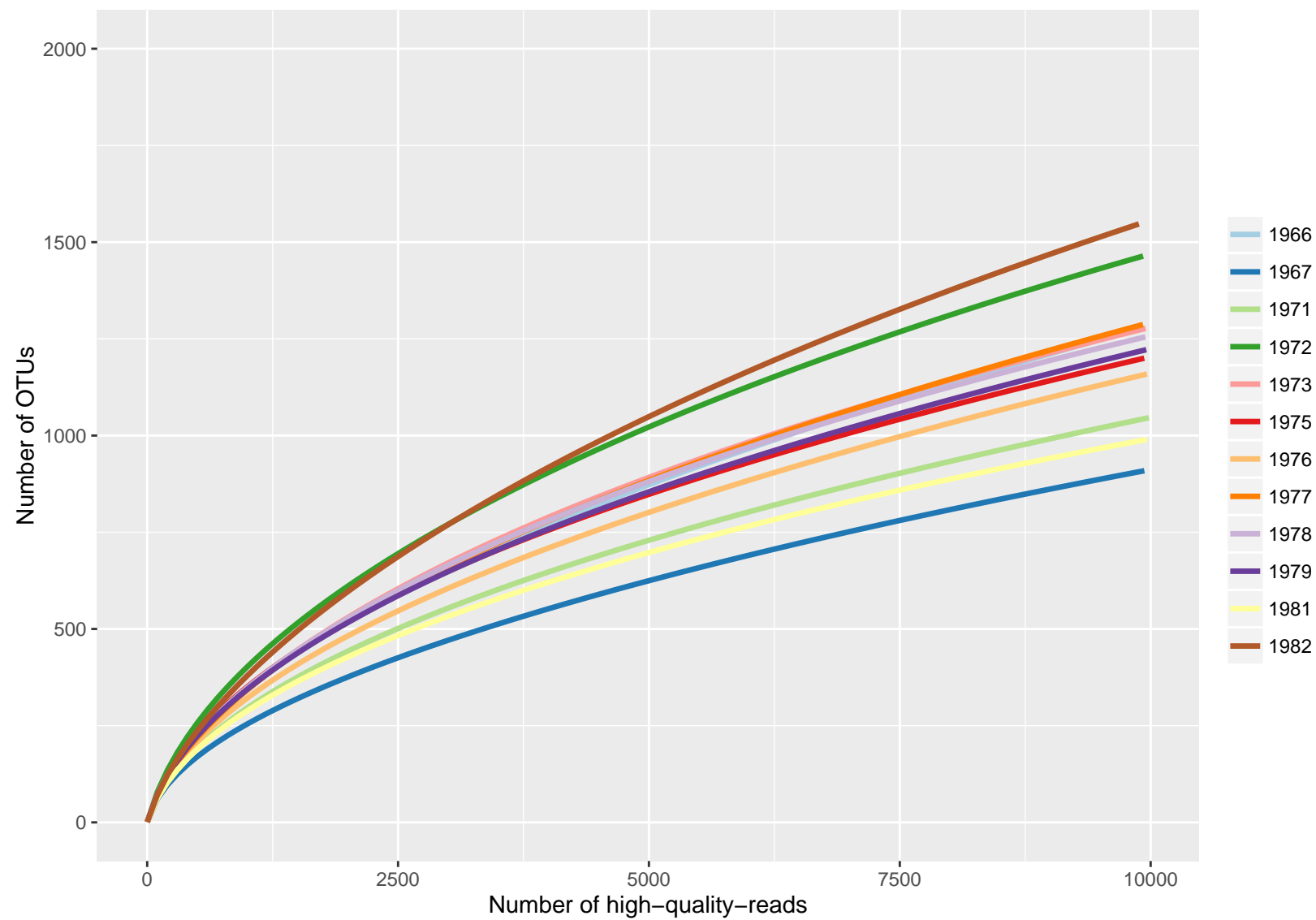

Supplement: S2 File — (ZIP) [file pone.0186766.s008.zip › Rarefact_curves_129.pdf]

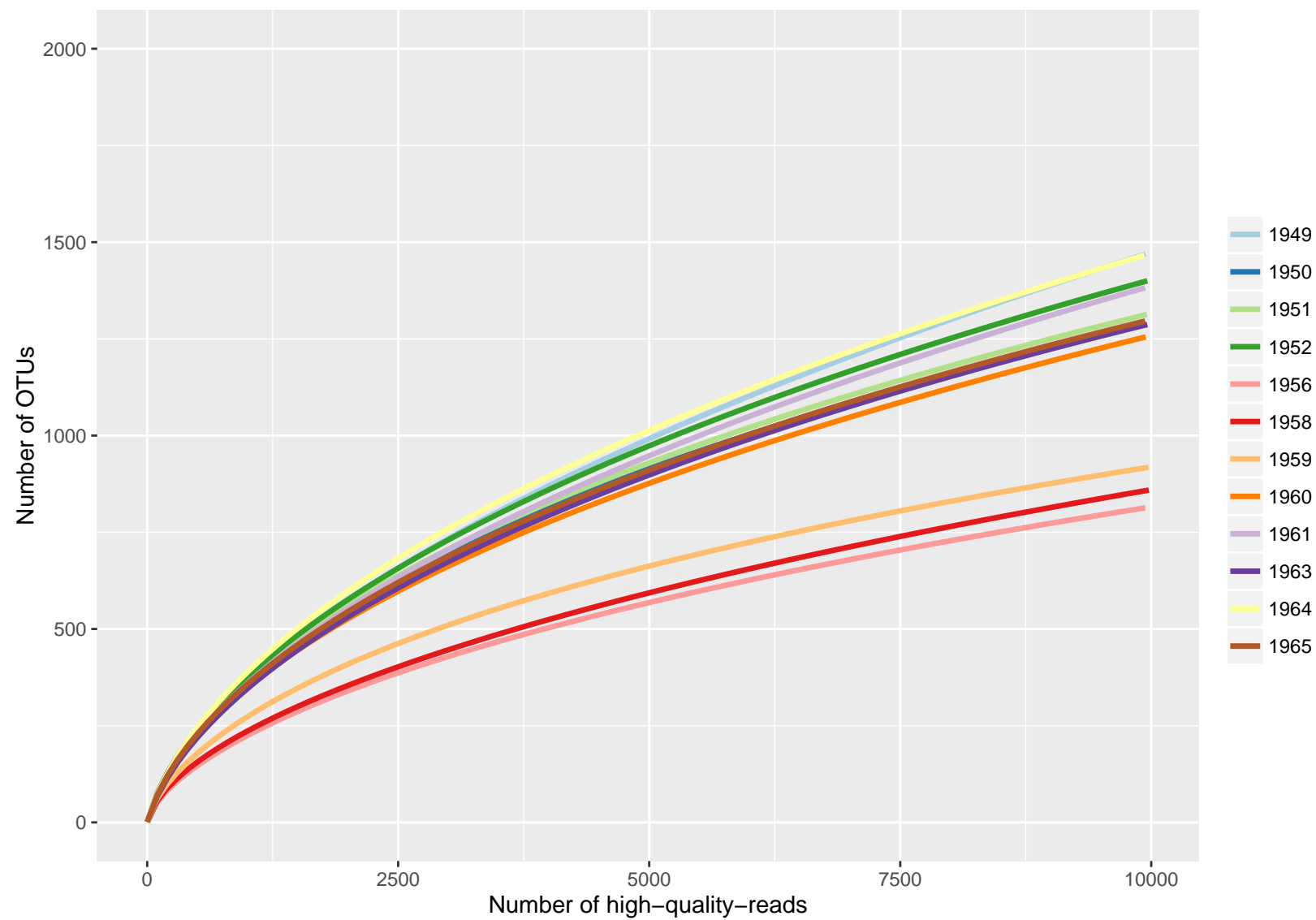

Supplement: S2 File — (ZIP) [file pone.0186766.s008.zip › Rarefact_curves_128.pdf]

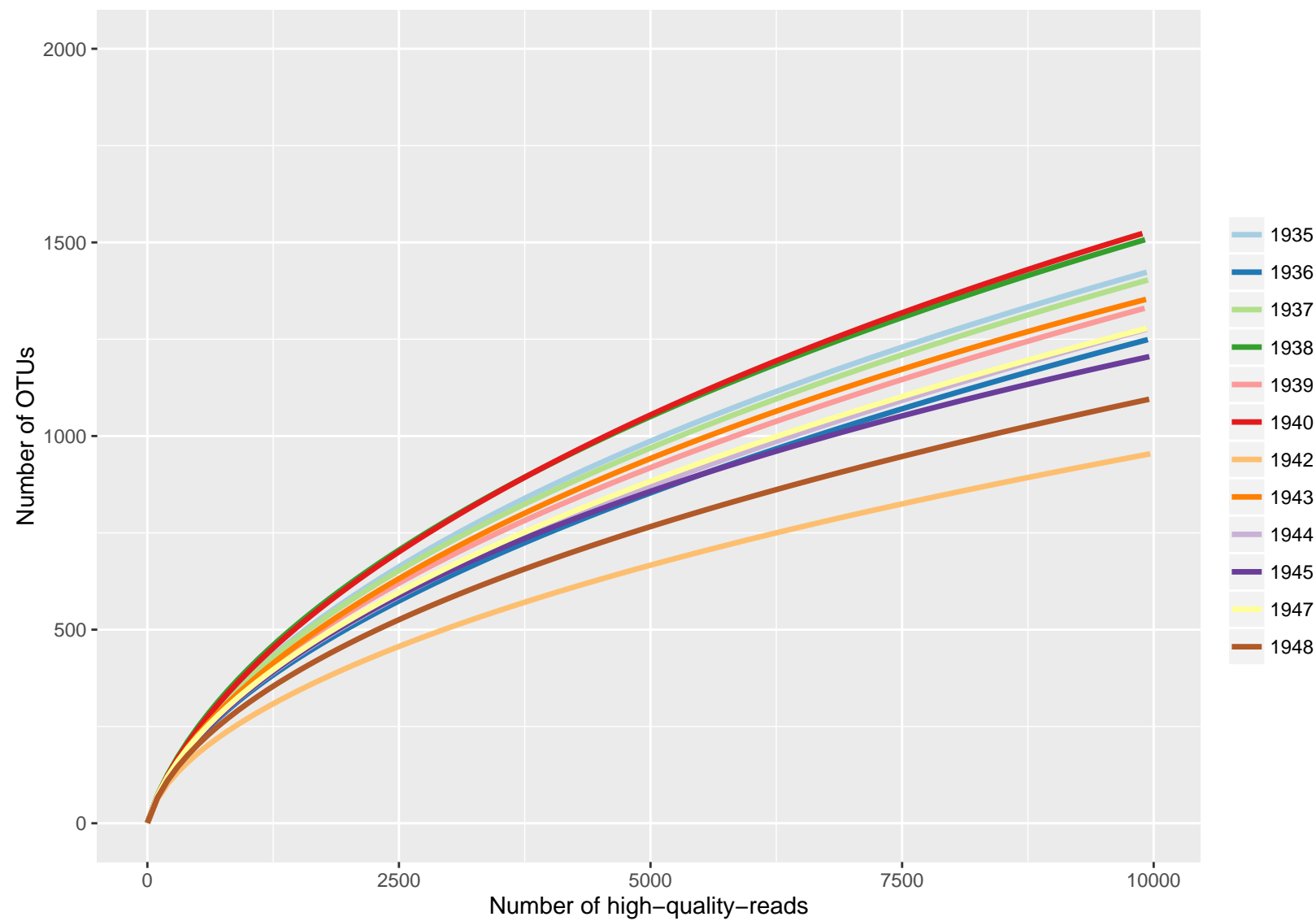

Supplement: S2 File — (ZIP) [file pone.0186766.s008.zip › Rarefact_curves_127.pdf]

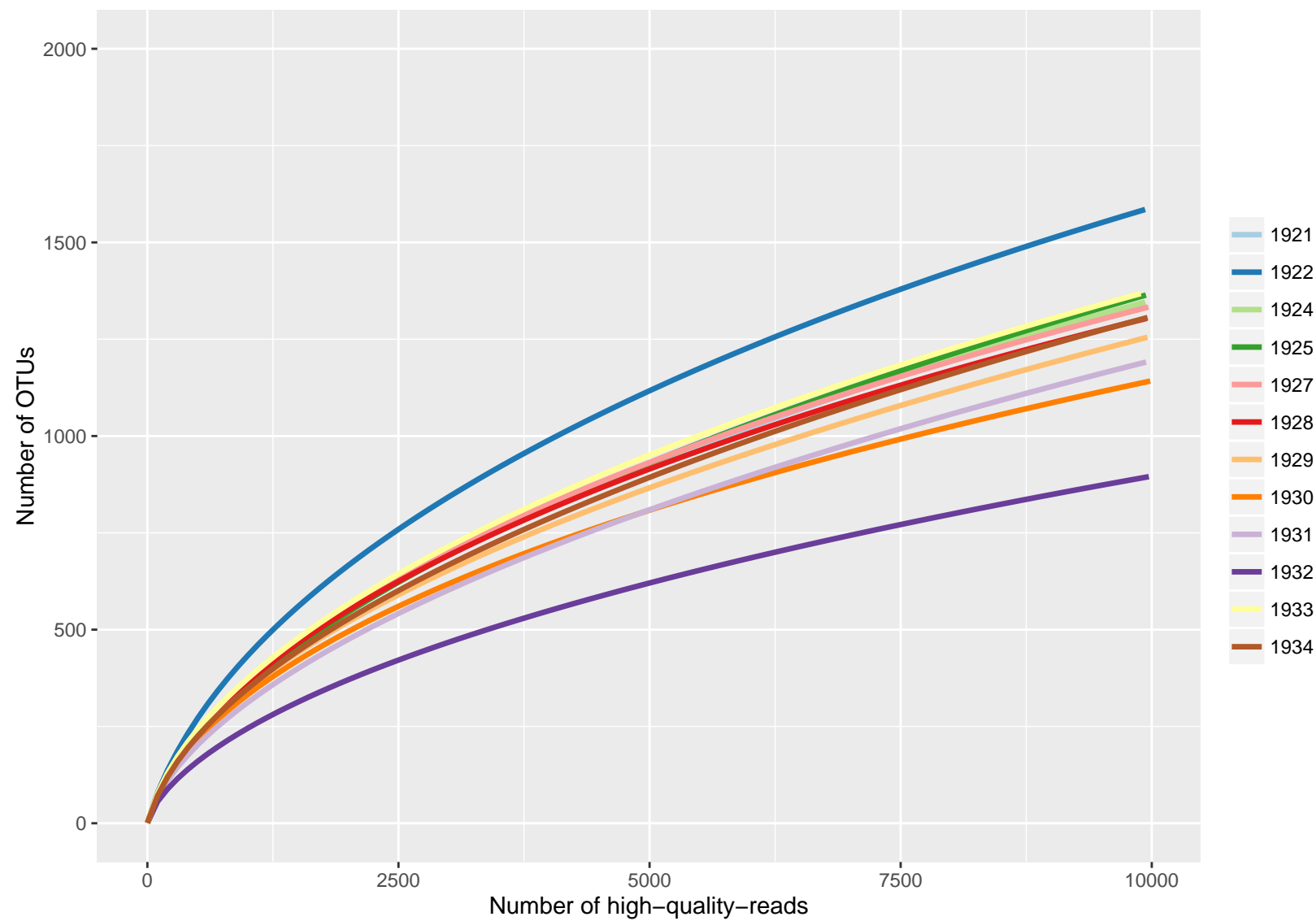

Supplement: S2 File — (ZIP) [file pone.0186766.s008.zip › Rarefact_curves_126.pdf]

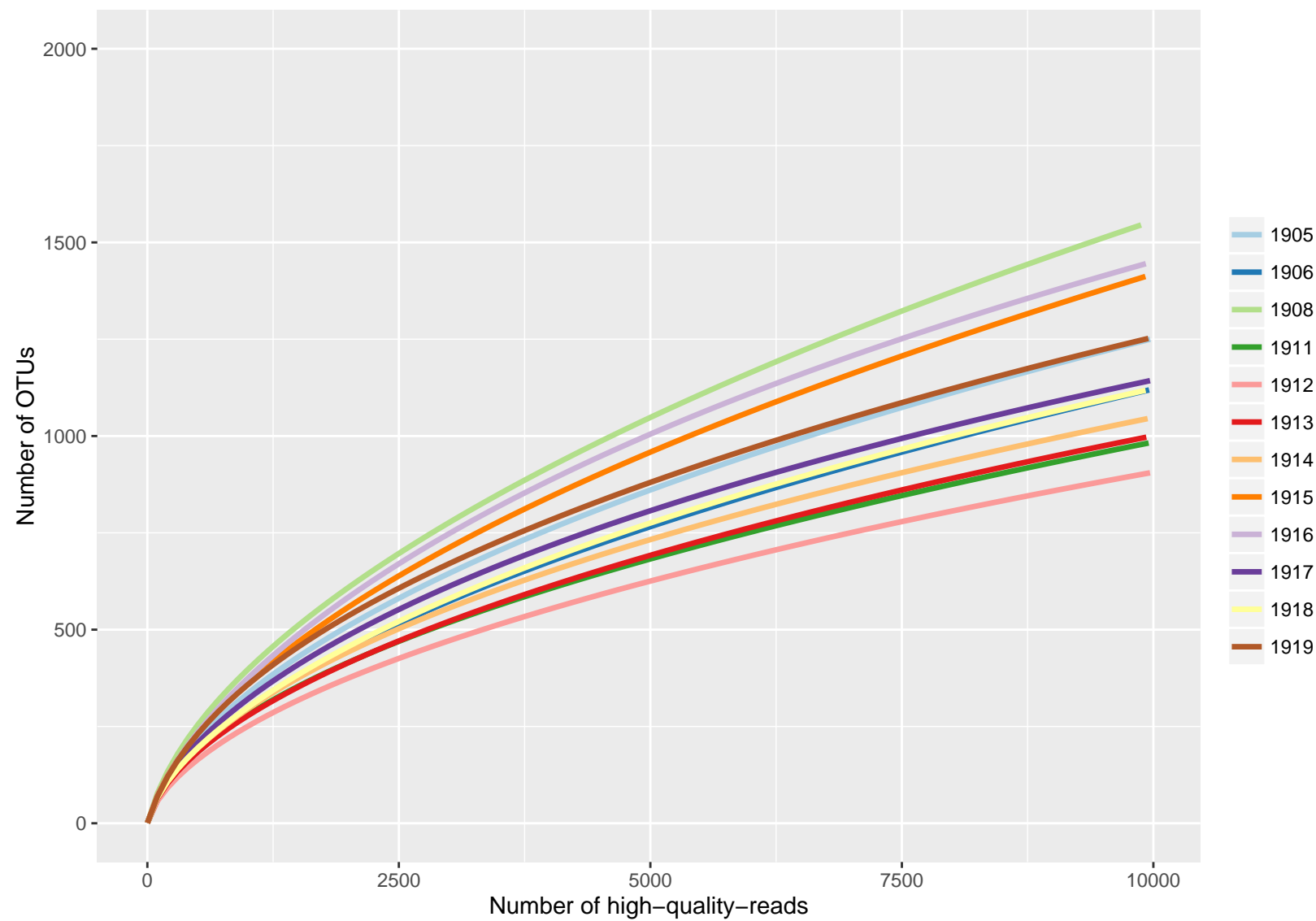

Supplement: S2 File — (ZIP) [file pone.0186766.s008.zip › Rarefact_curves_125.pdf]

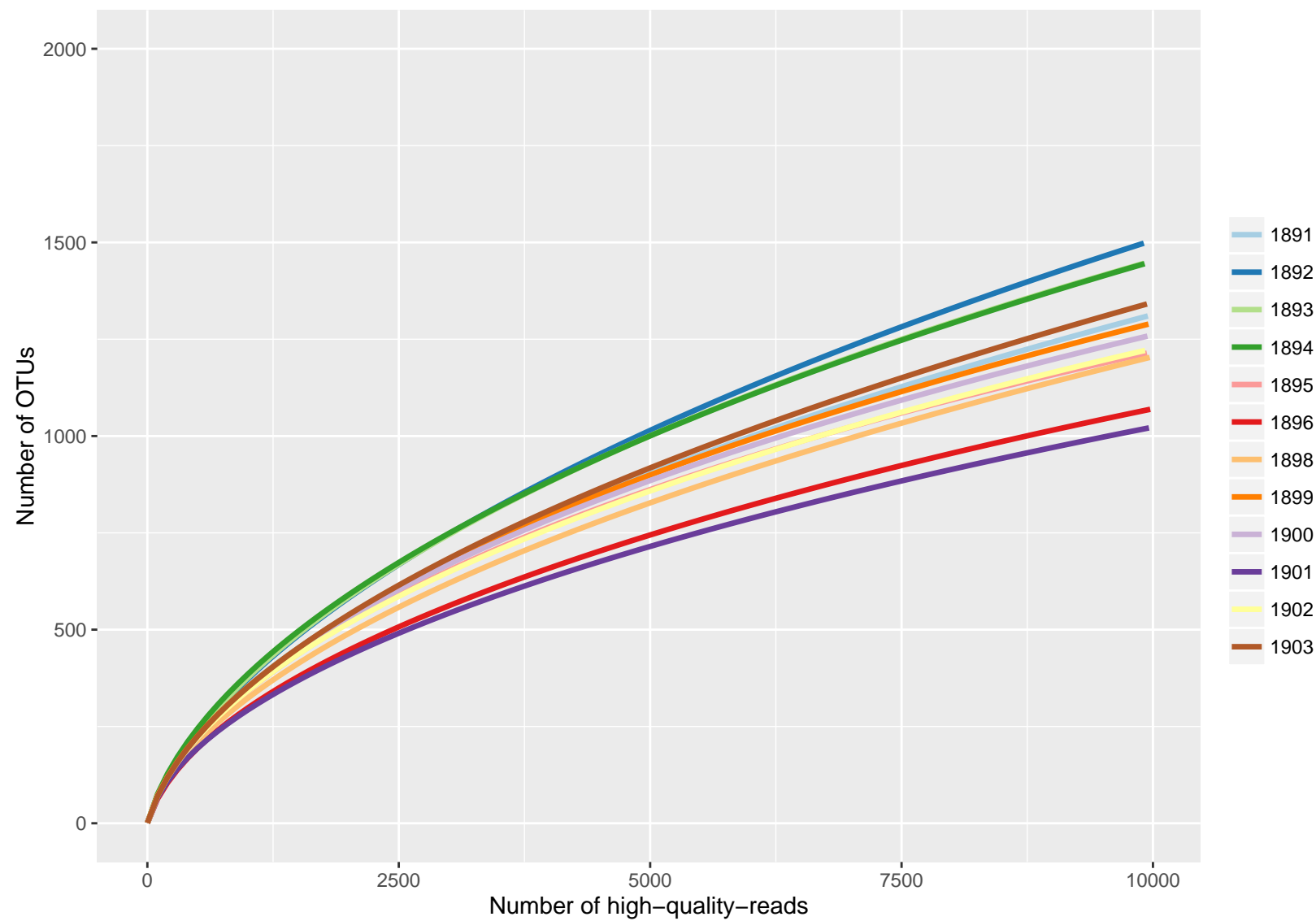

Supplement: S2 File — (ZIP) [file pone.0186766.s008.zip › Rarefact_curves_124.pdf]

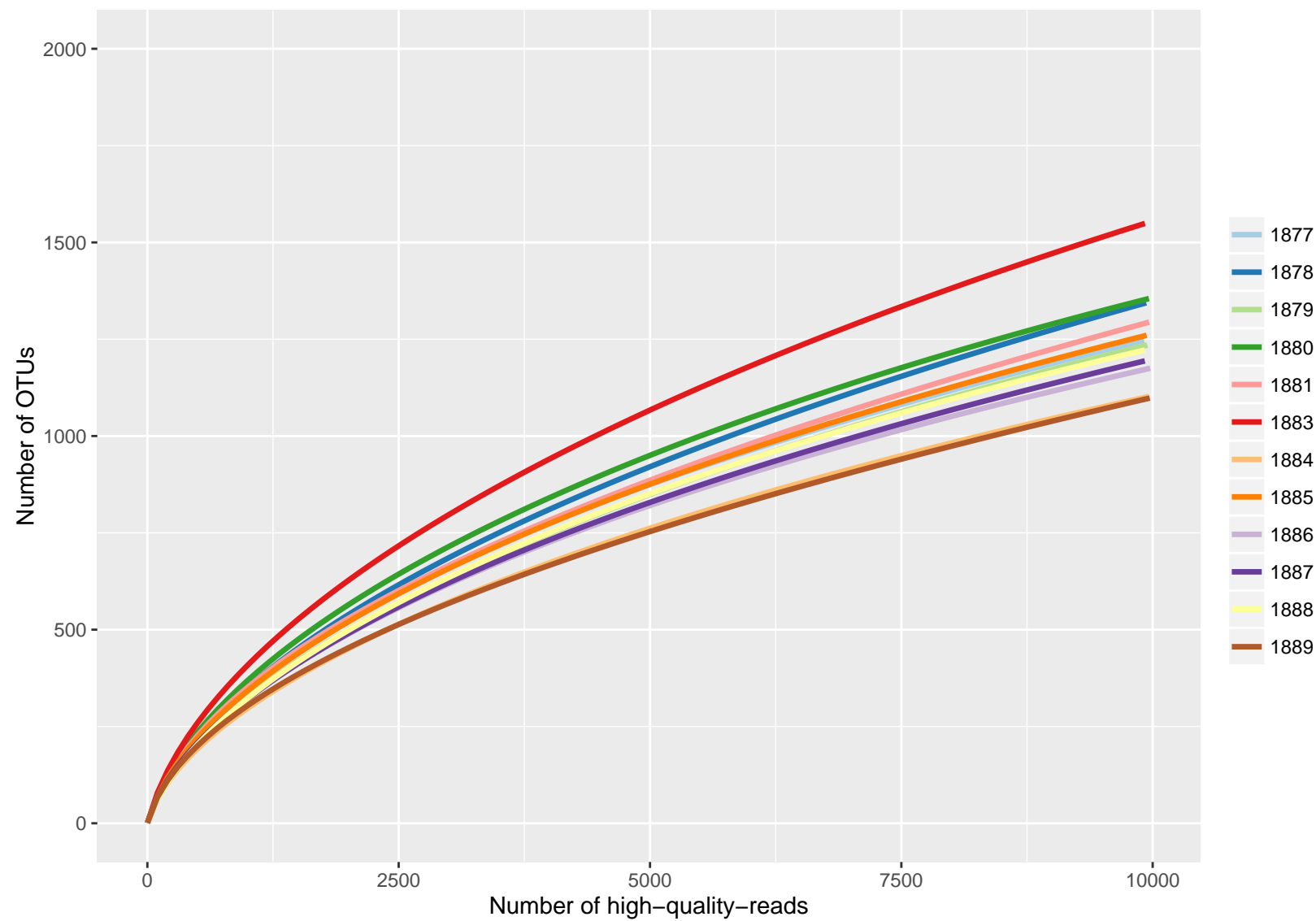

Supplement: S2 File — (ZIP) [file pone.0186766.s008.zip › Rarefact_curves_123.pdf]

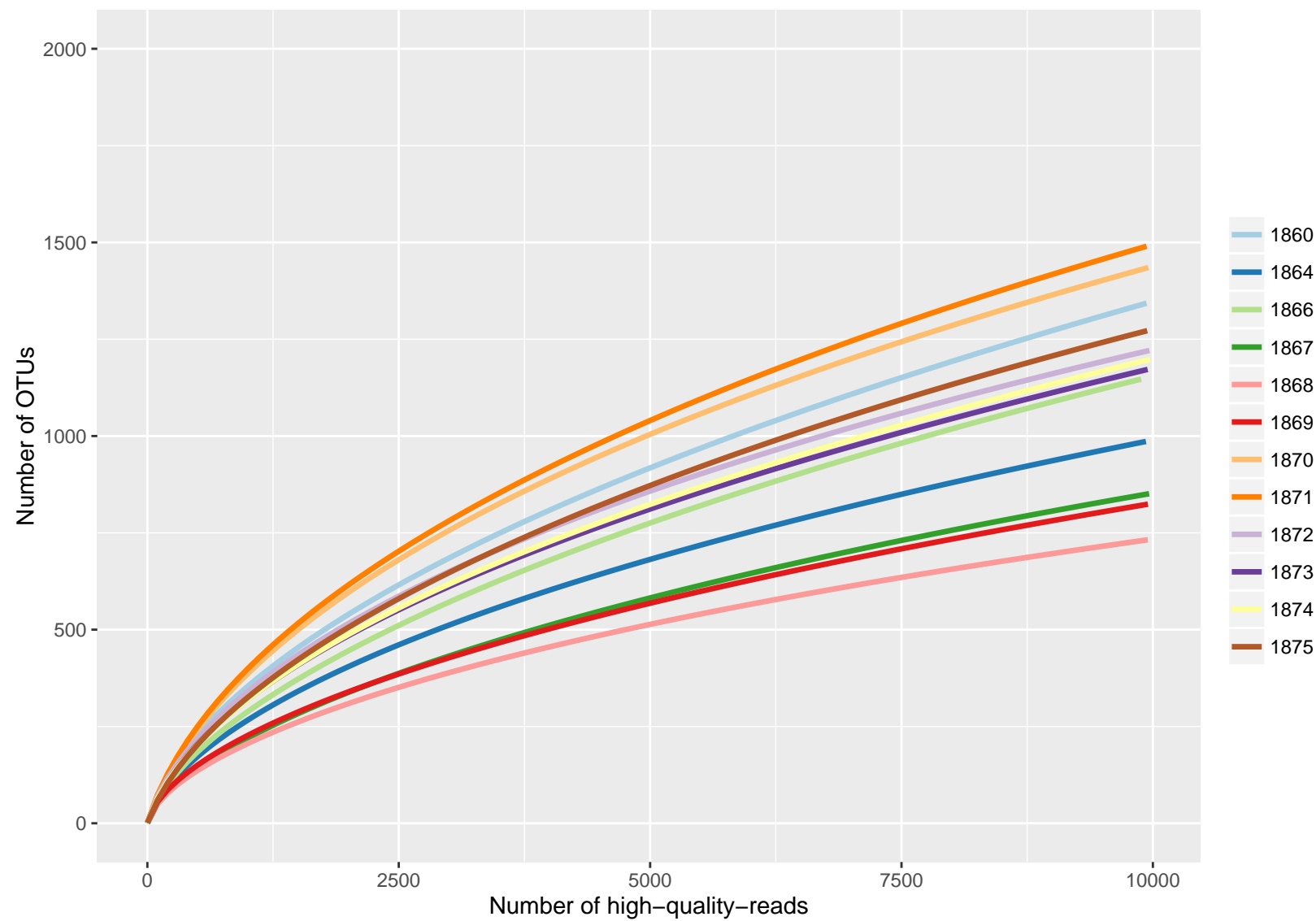

Supplement: S2 File — (ZIP) [file pone.0186766.s008.zip › Rarefact_curves_122.pdf]

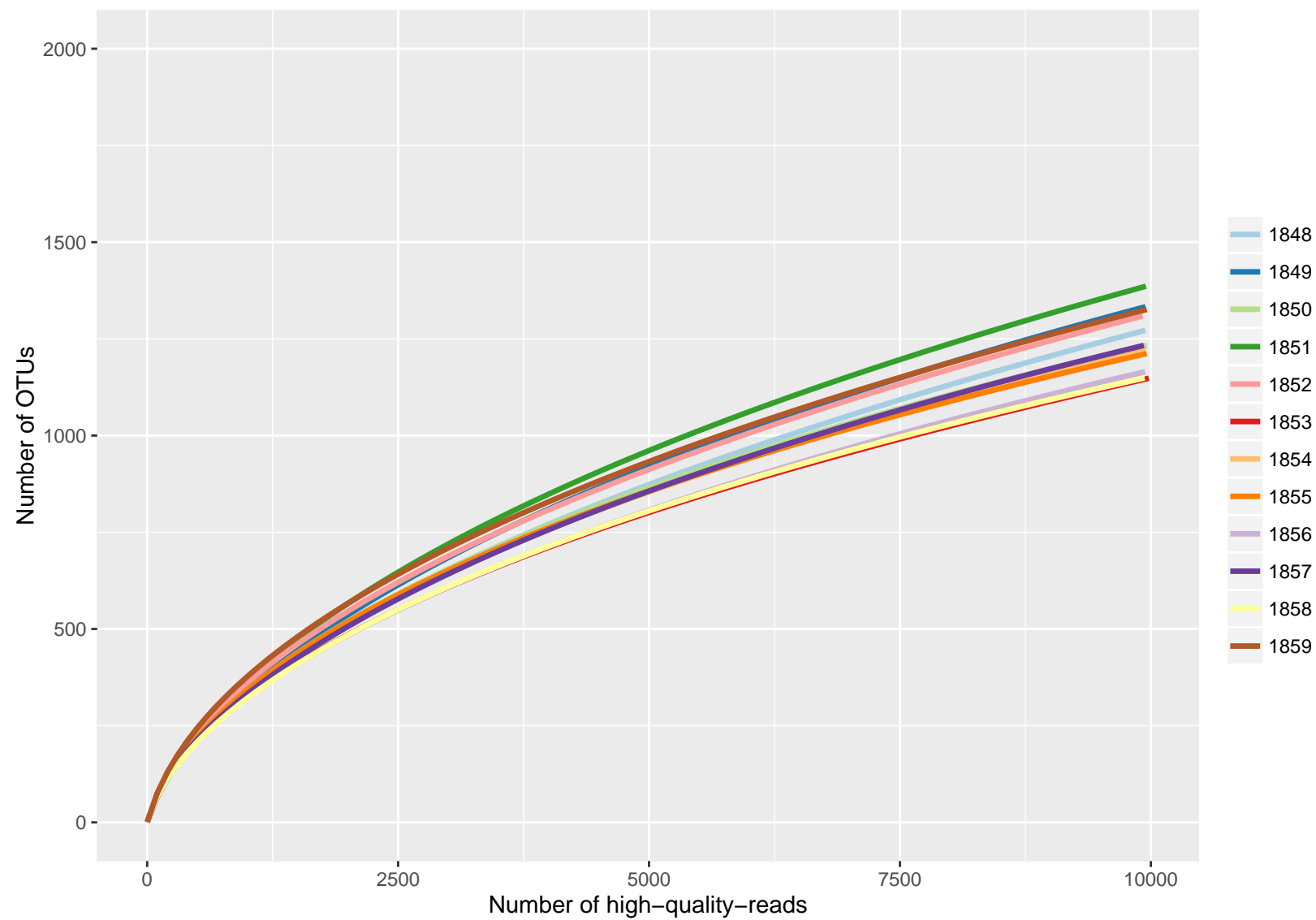

Supplement: S2 File — (ZIP) [file pone.0186766.s008.zip › Rarefact_curves_121.pdf]

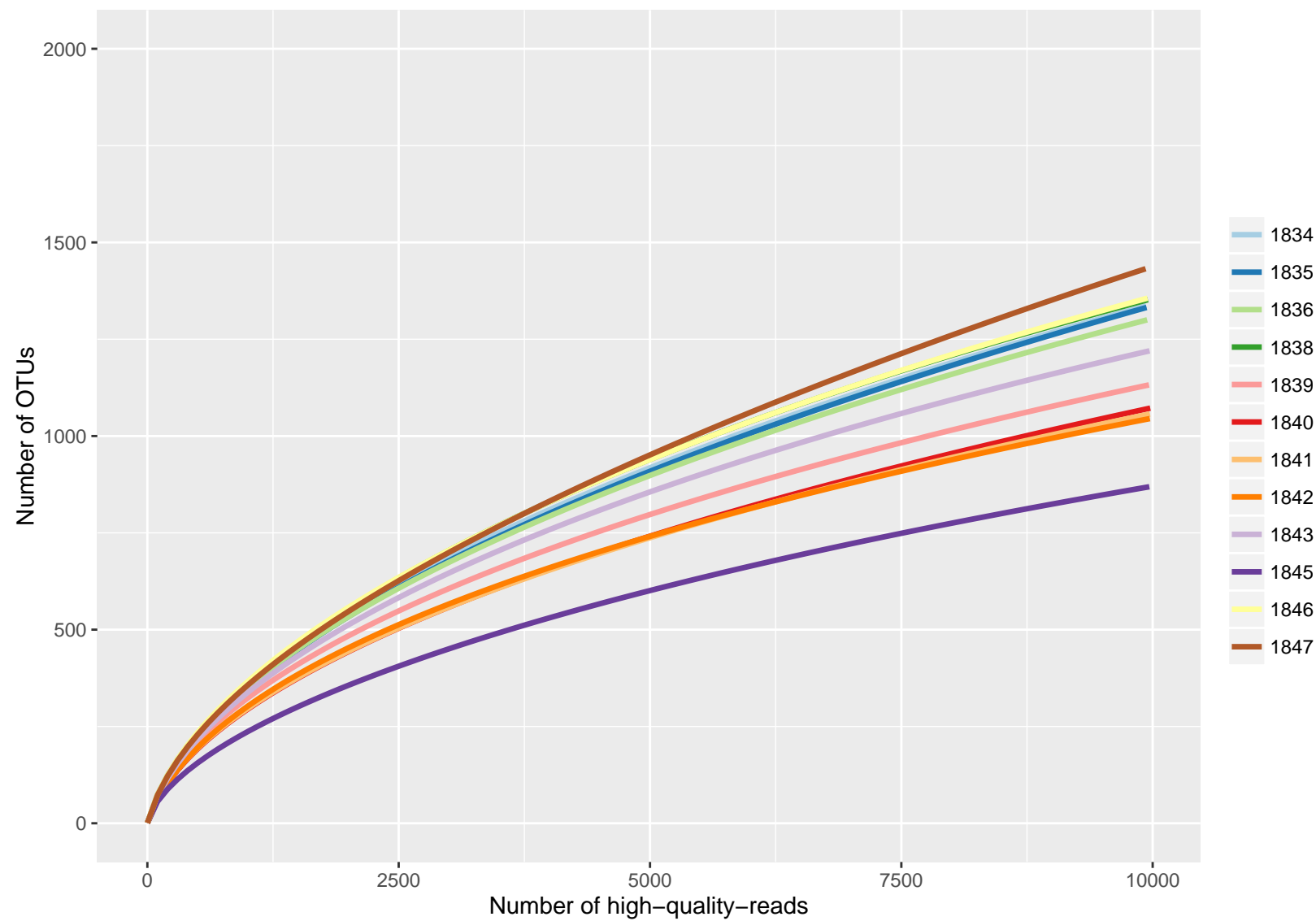

Supplement: S2 File — (ZIP) [file pone.0186766.s008.zip › Rarefact_curves_120.pdf]

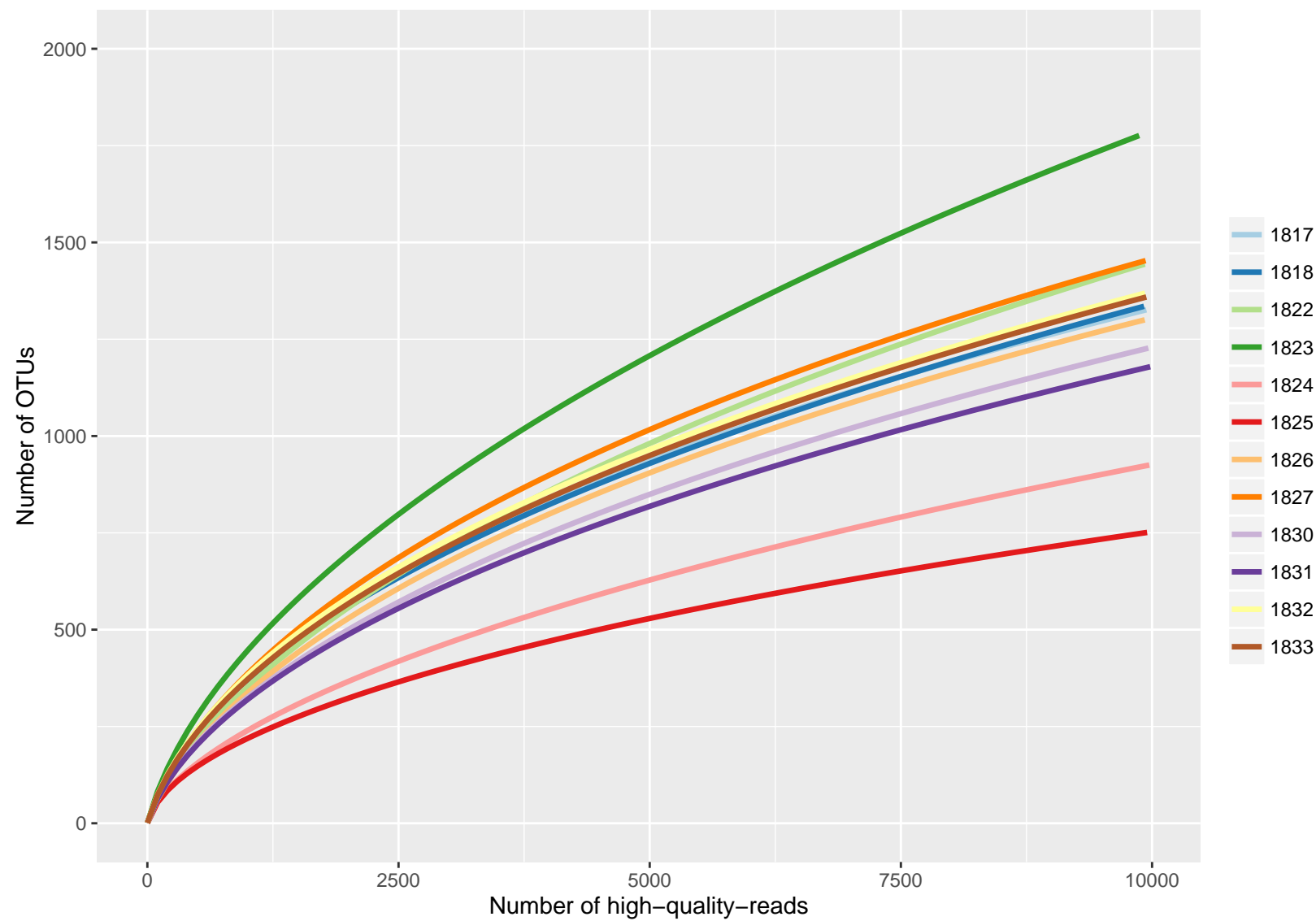

Supplement: S2 File — (ZIP) [file pone.0186766.s008.zip › Rarefact_curves_119.pdf]

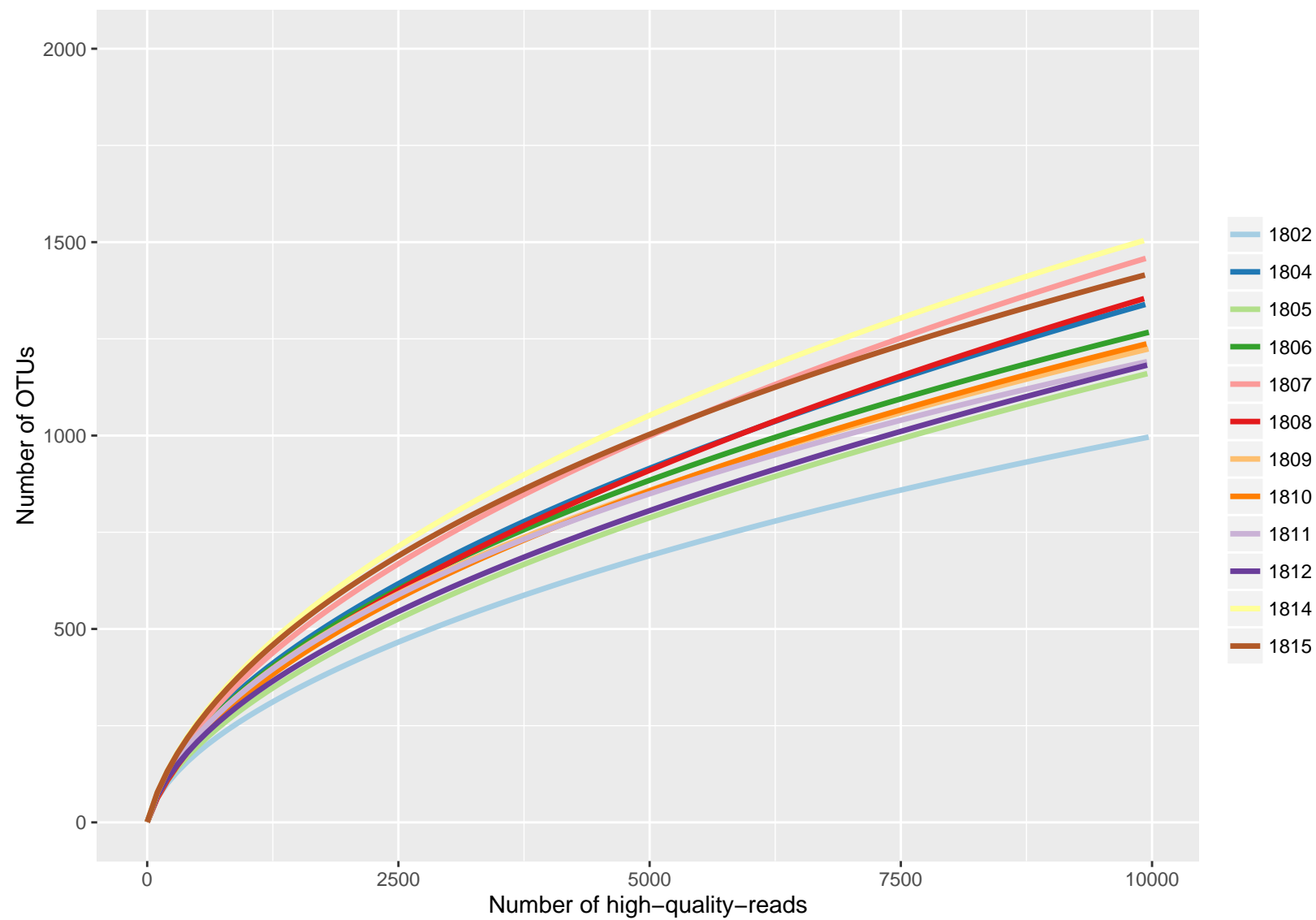

Supplement: S2 File — (ZIP) [file pone.0186766.s008.zip › Rarefact_curves_118.pdf]

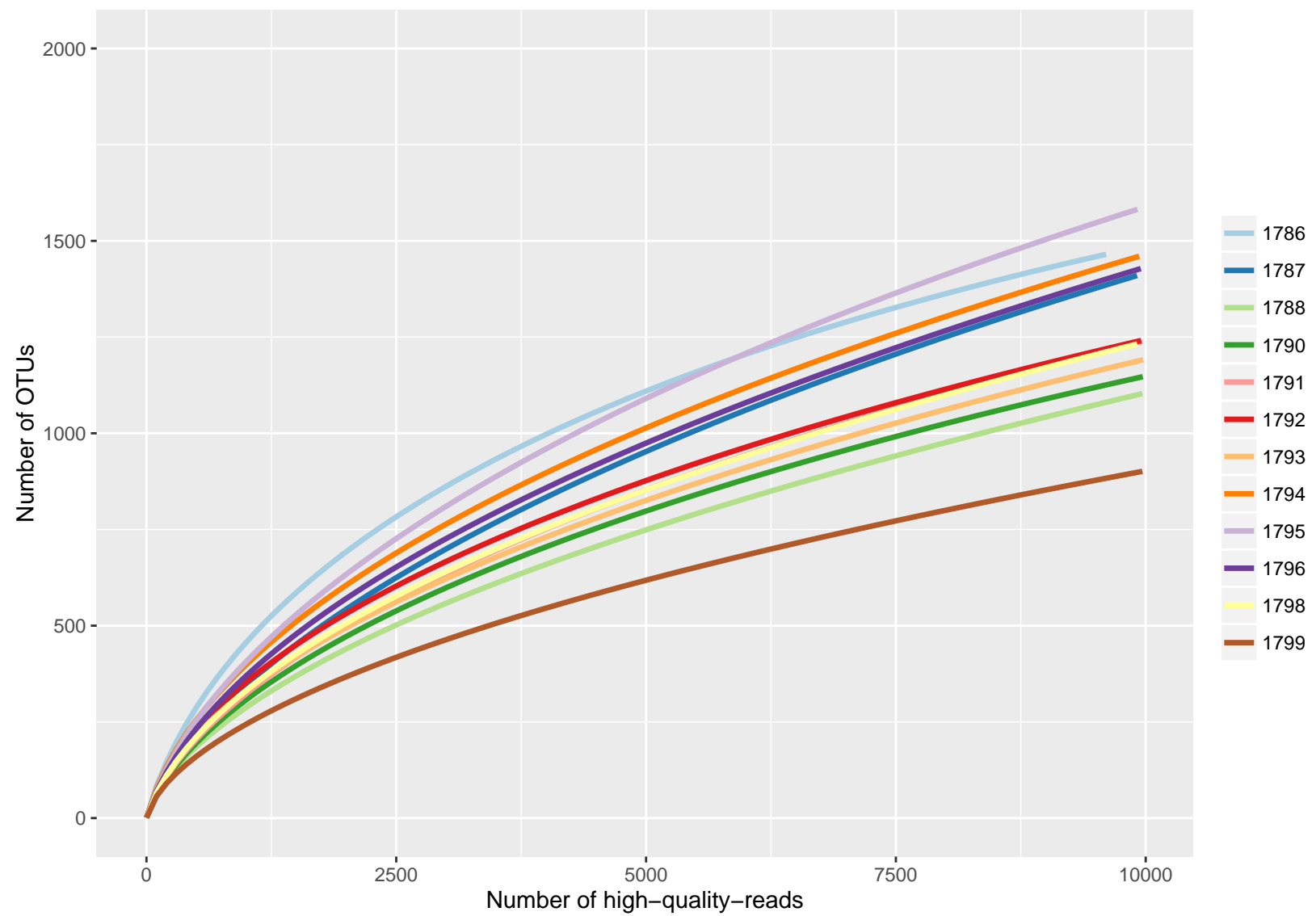

Supplement: S2 File — (ZIP) [file pone.0186766.s008.zip › Rarefact_curves_117.pdf]

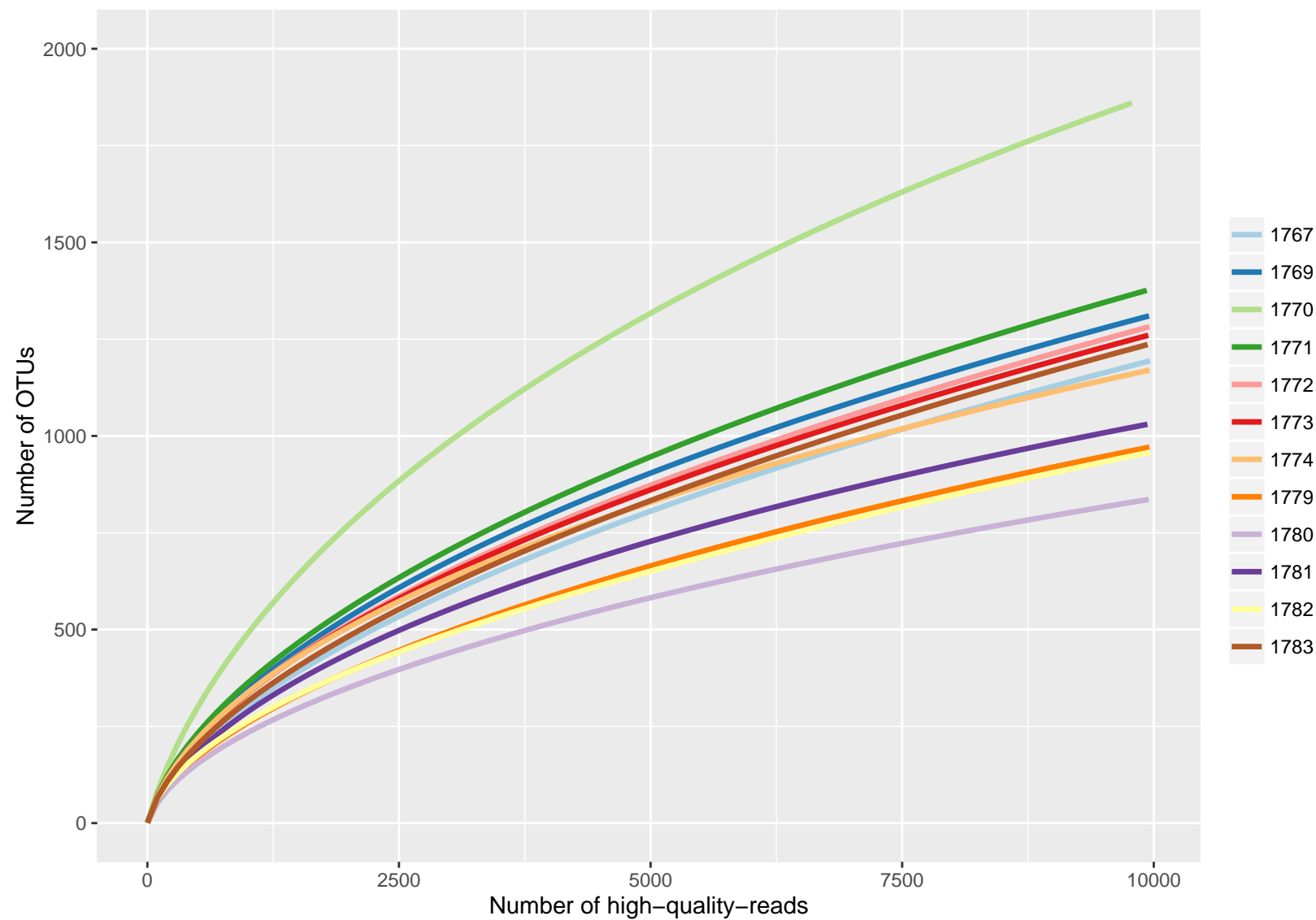

Supplement: S2 File — (ZIP) [file pone.0186766.s008.zip › Rarefact_curves_116.pdf]

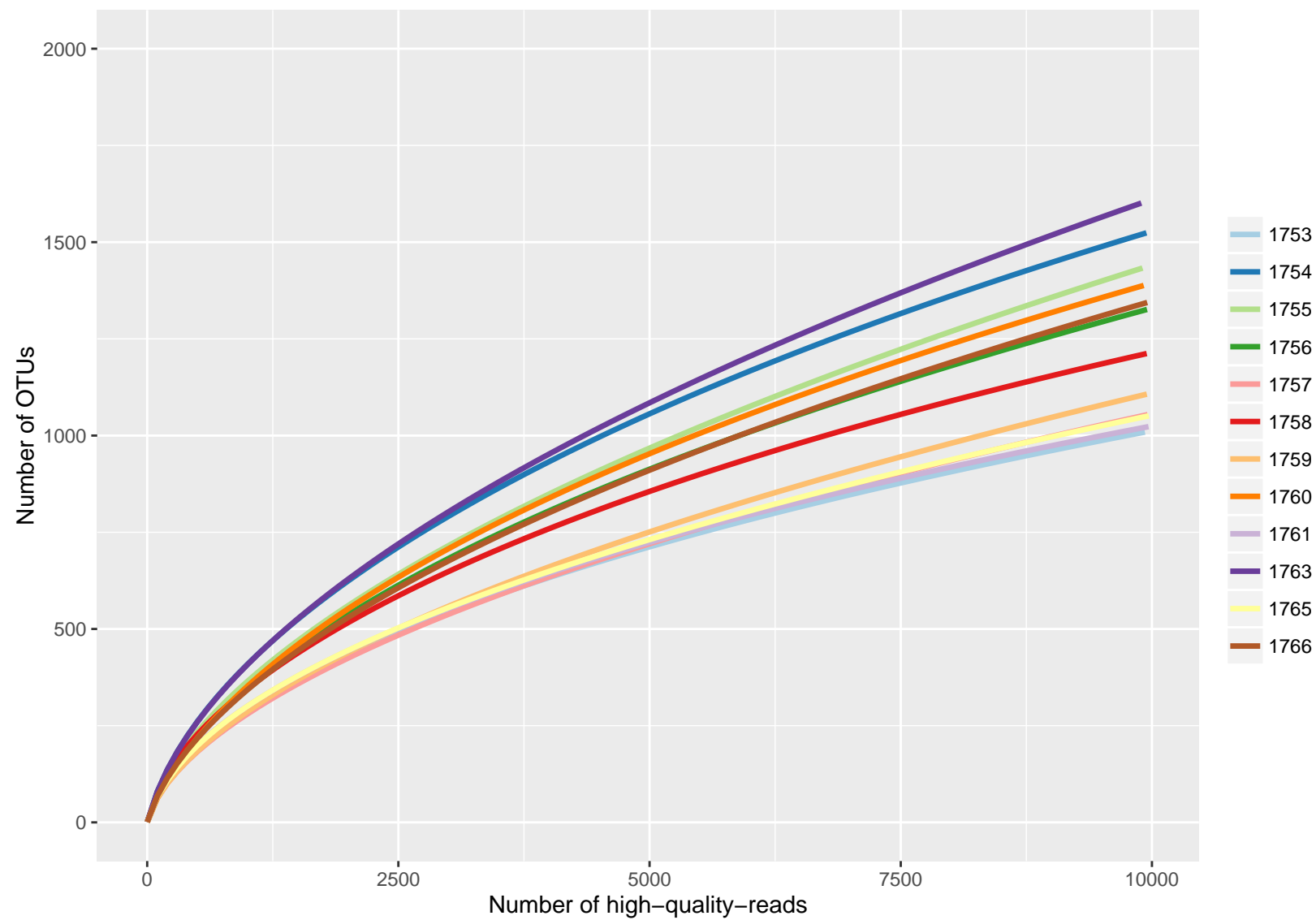

Supplement: S2 File — (ZIP) [file pone.0186766.s008.zip › Rarefact_curves_115.pdf]

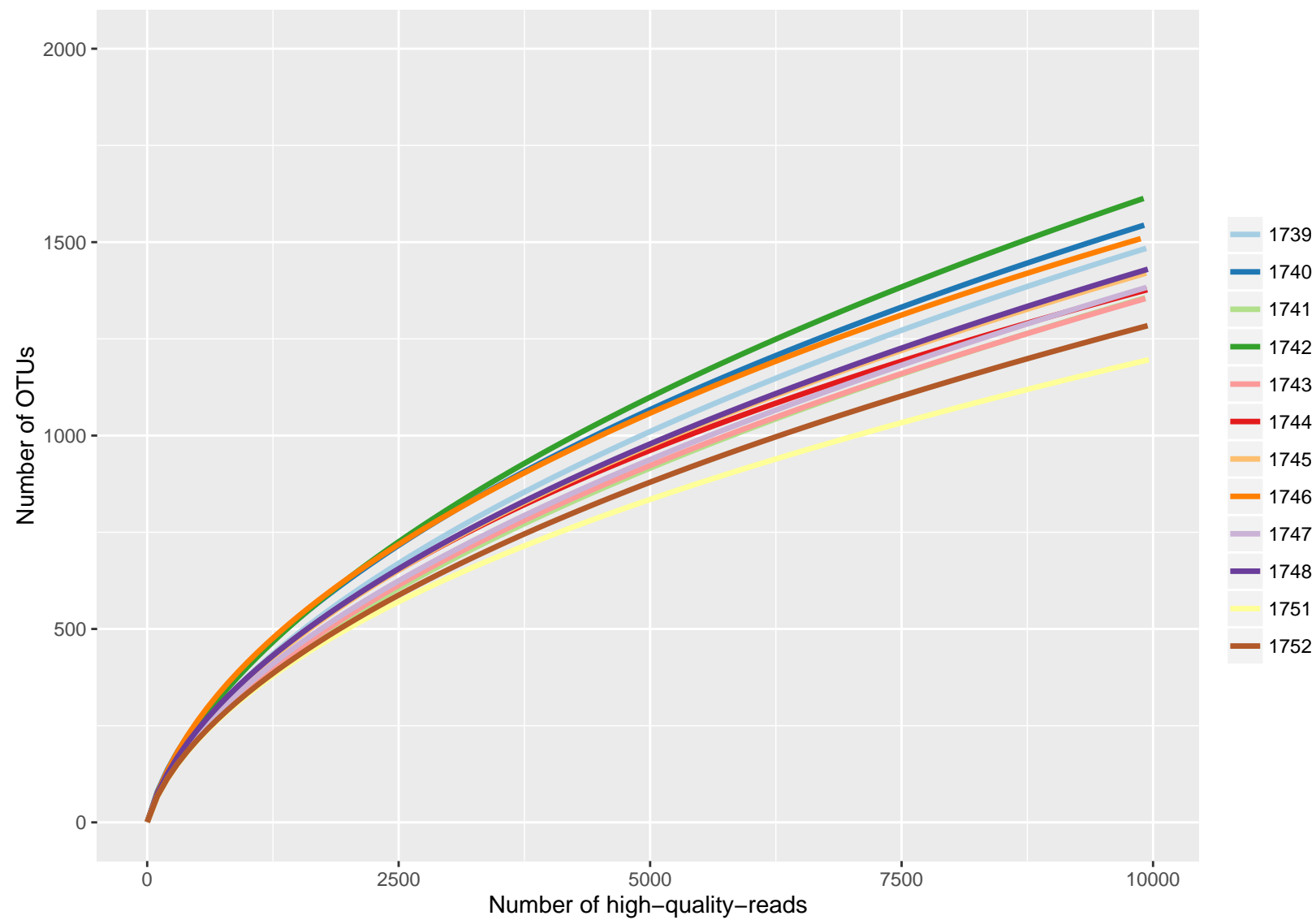

Supplement: S2 File — (ZIP) [file pone.0186766.s008.zip › Rarefact_curves_114.pdf]

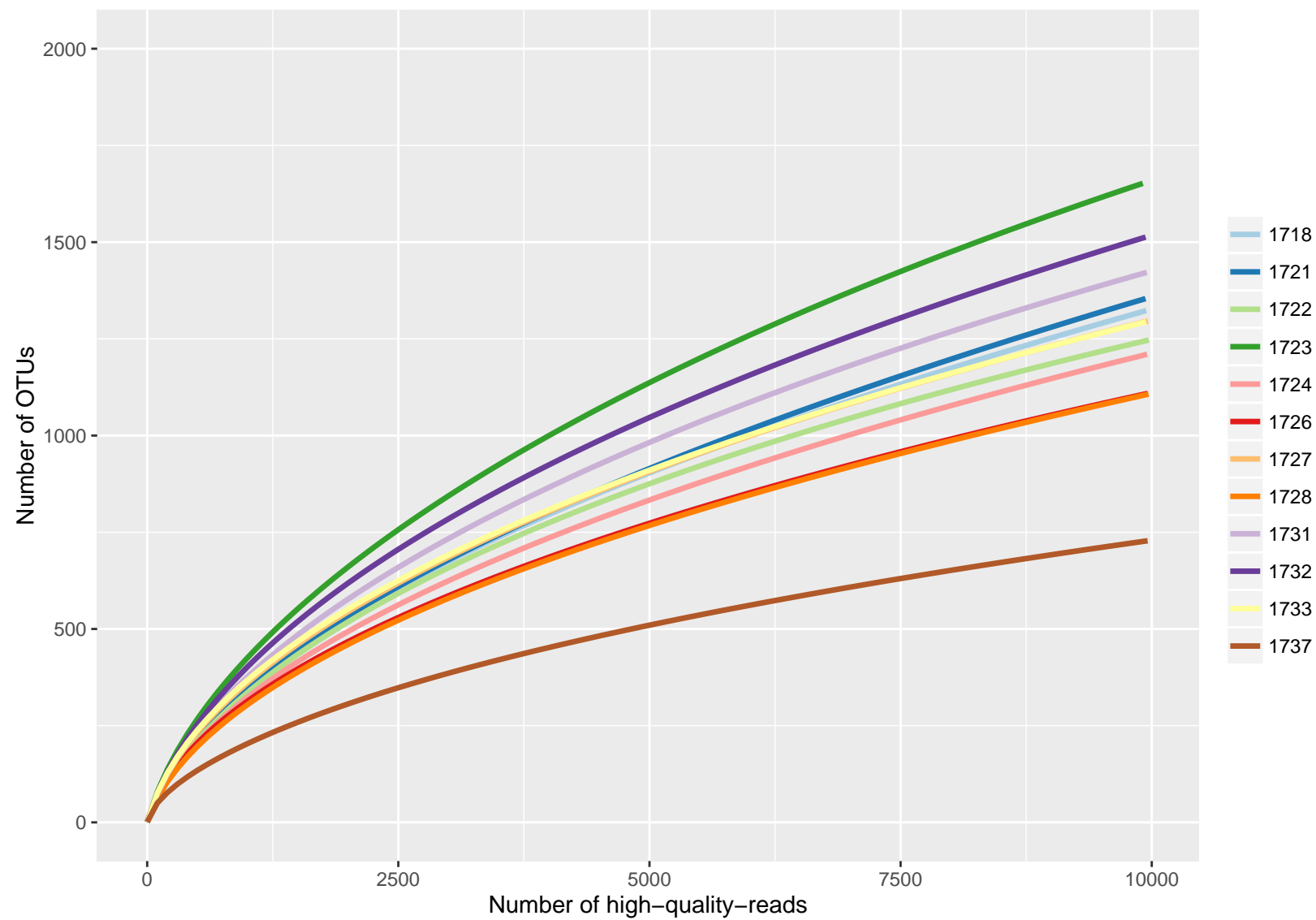

Supplement: S2 File — (ZIP) [file pone.0186766.s008.zip › Rarefact_curves_113.pdf]

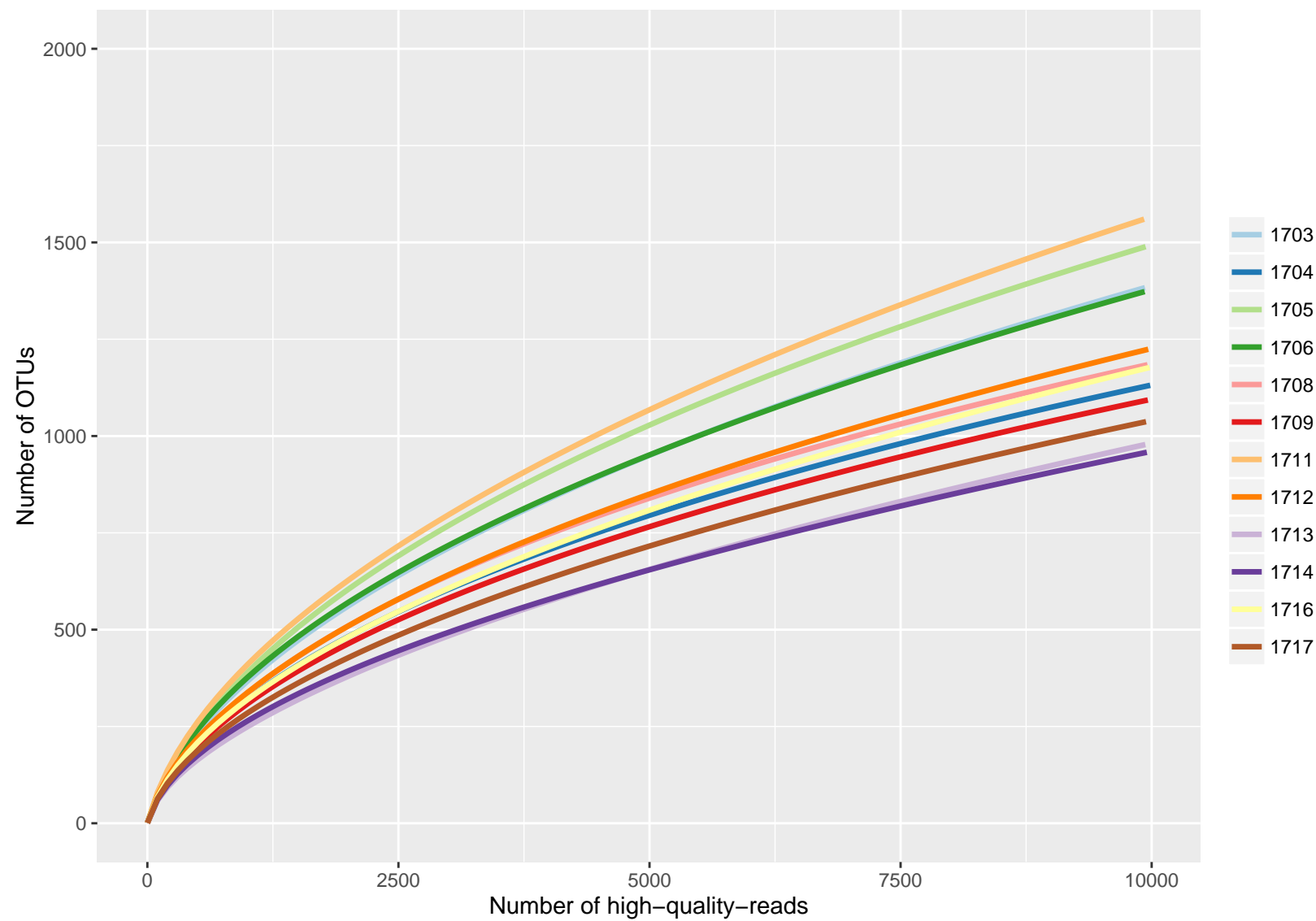

Supplement: S2 File — (ZIP) [file pone.0186766.s008.zip › Rarefact_curves_112.pdf]

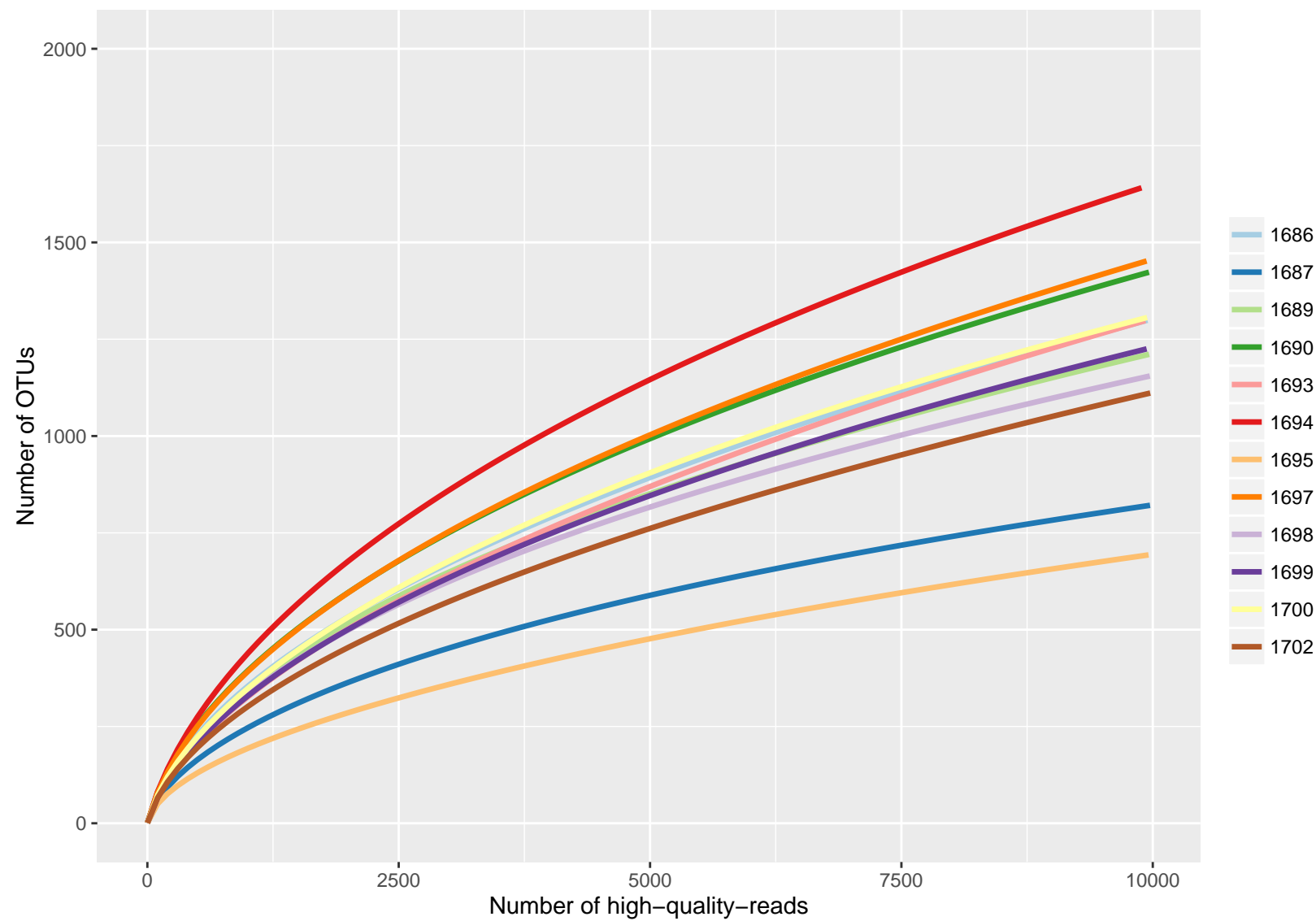

Supplement: S2 File — (ZIP) [file pone.0186766.s008.zip › Rarefact_curves_111.pdf]

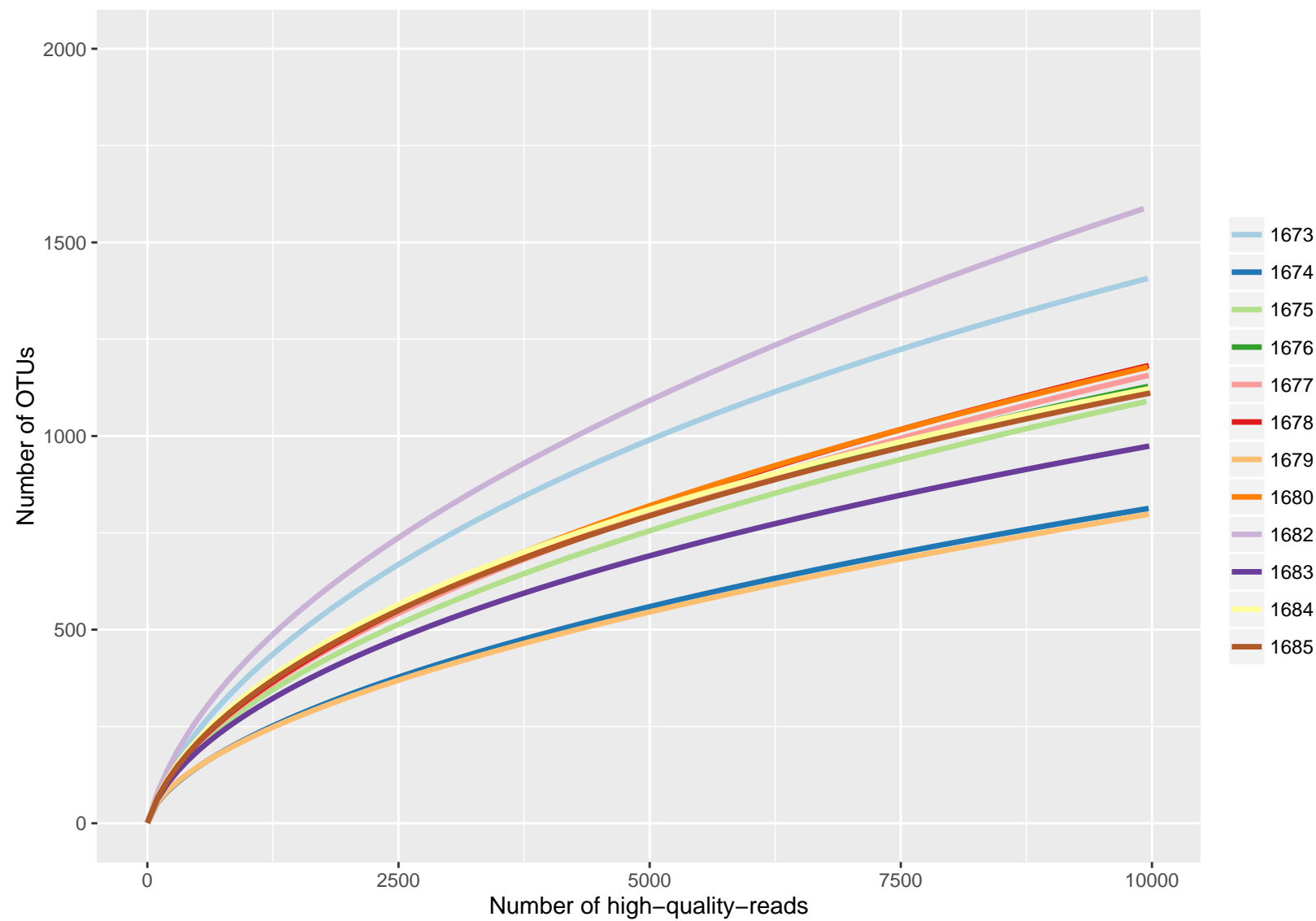

Supplement: S2 File — (ZIP) [file pone.0186766.s008.zip › Rarefact_curves_110.pdf]

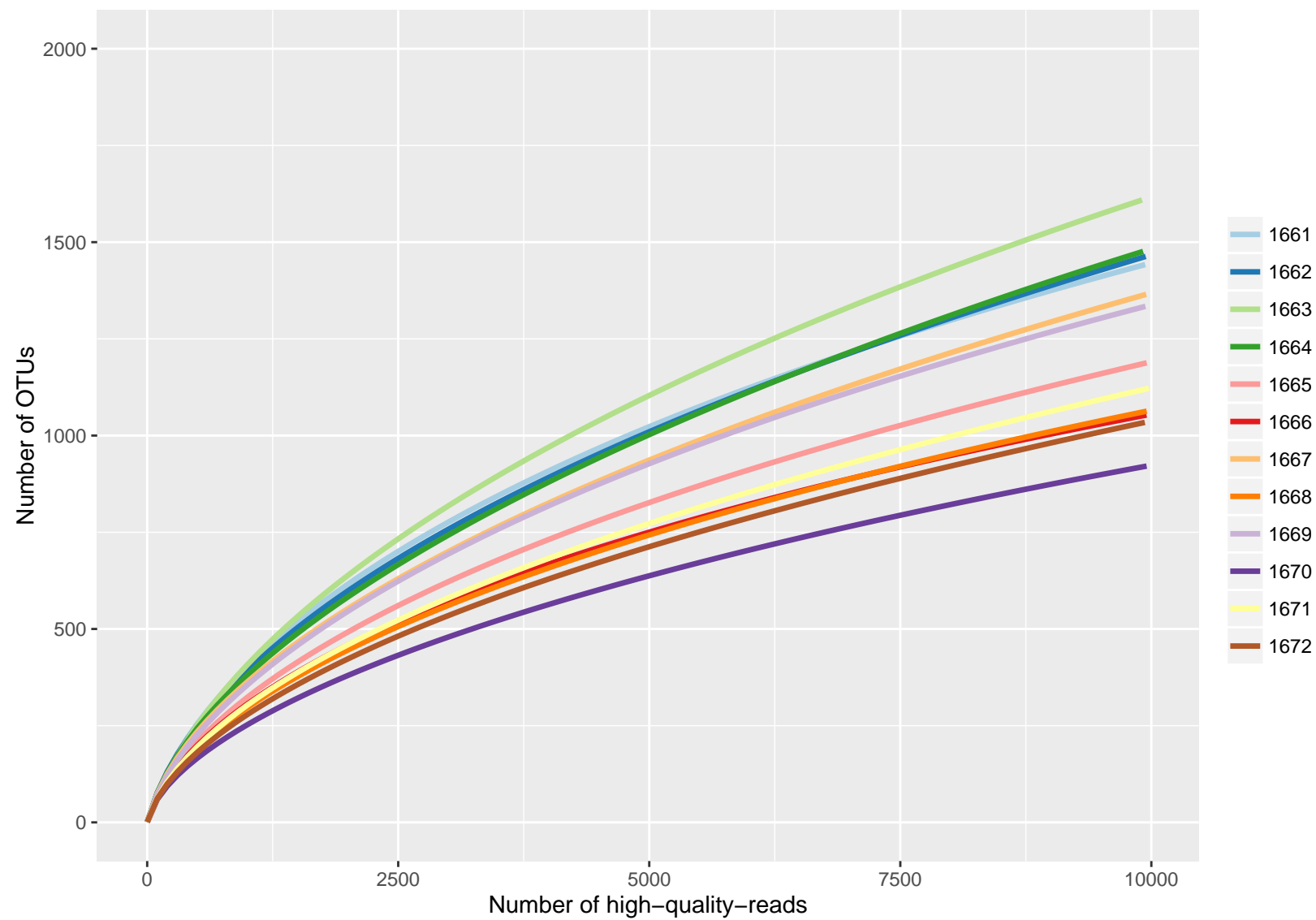

Supplement: S2 File — (ZIP) [file pone.0186766.s008.zip › Rarefact_curves_109.pdf]

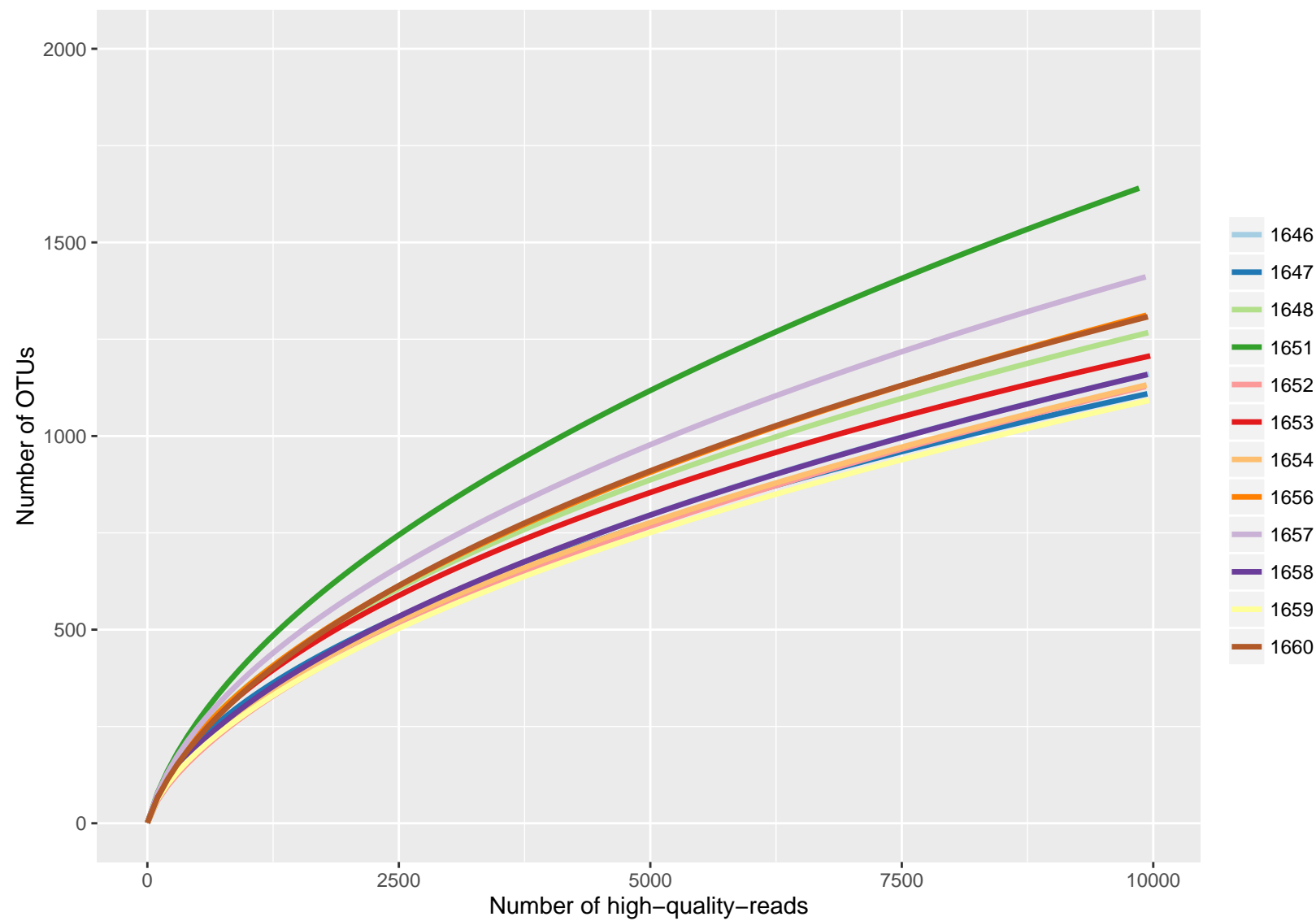

Supplement: S2 File — (ZIP) [file pone.0186766.s008.zip › Rarefact_curves_108.pdf]

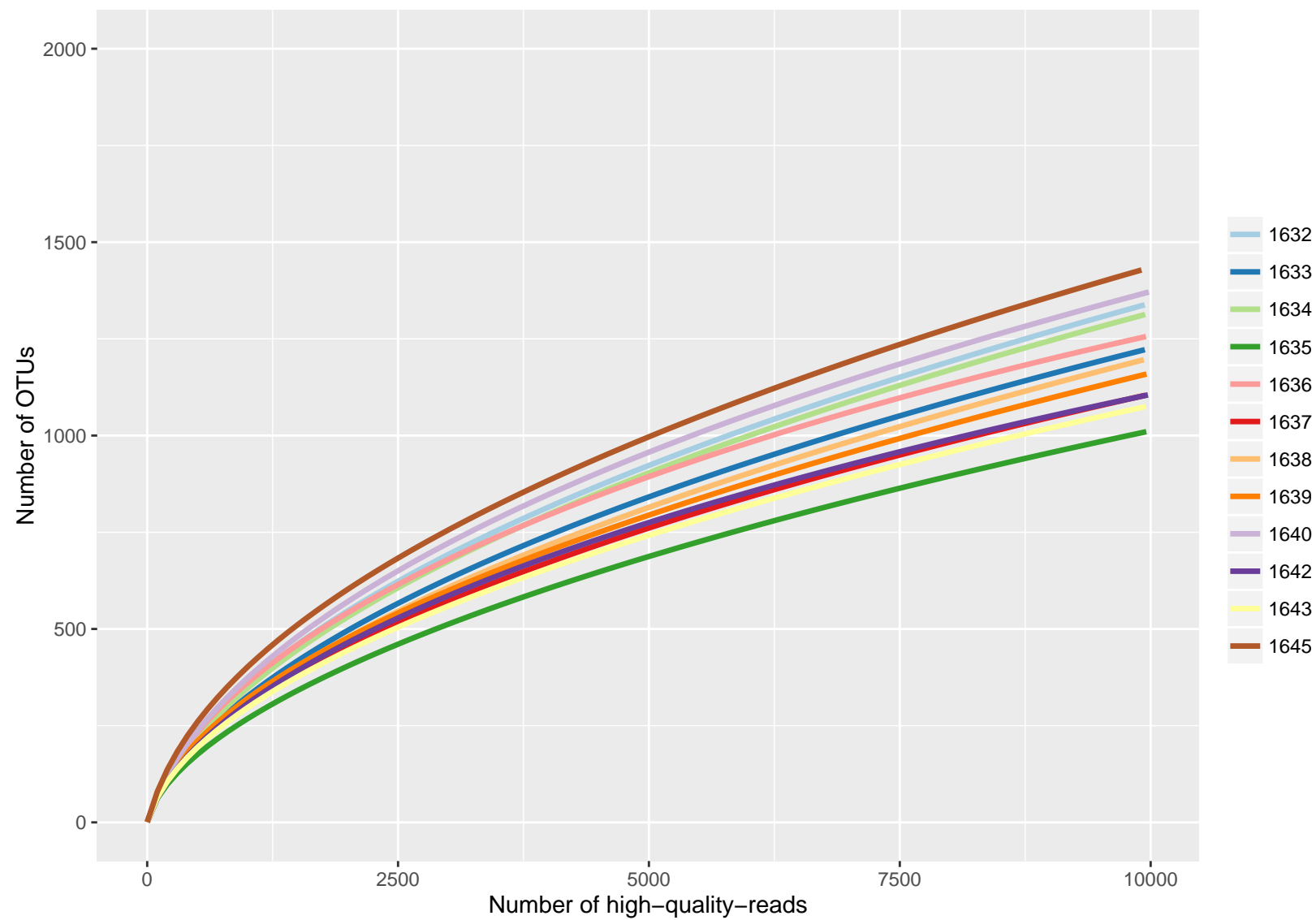

Supplement: S2 File — (ZIP) [file pone.0186766.s008.zip › Rarefact_curves_107.pdf]

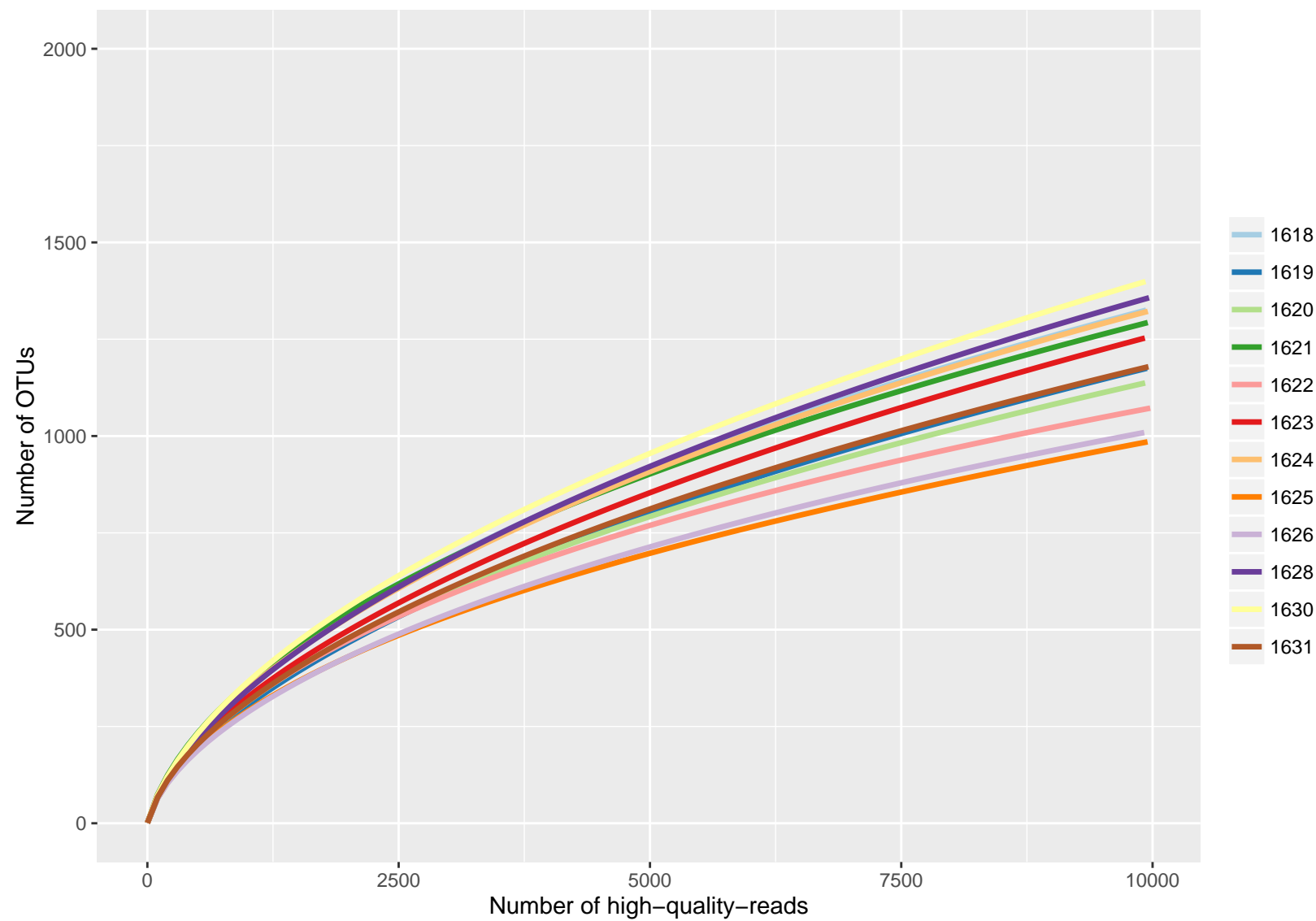

Supplement: S2 File — (ZIP) [file pone.0186766.s008.zip › Rarefact_curves_106.pdf]

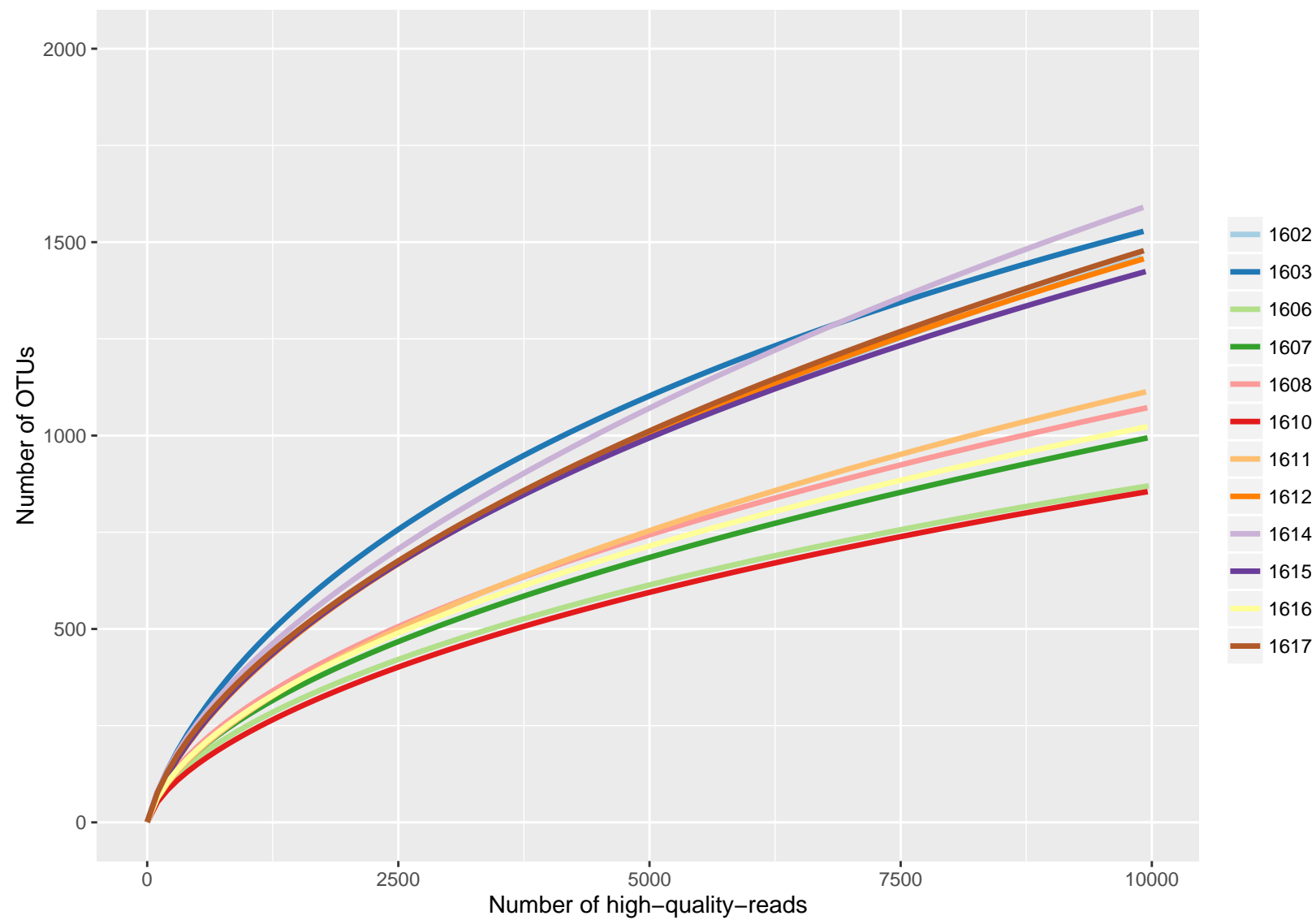

Supplement: S2 File — (ZIP) [file pone.0186766.s008.zip › Rarefact_curves_105.pdf]

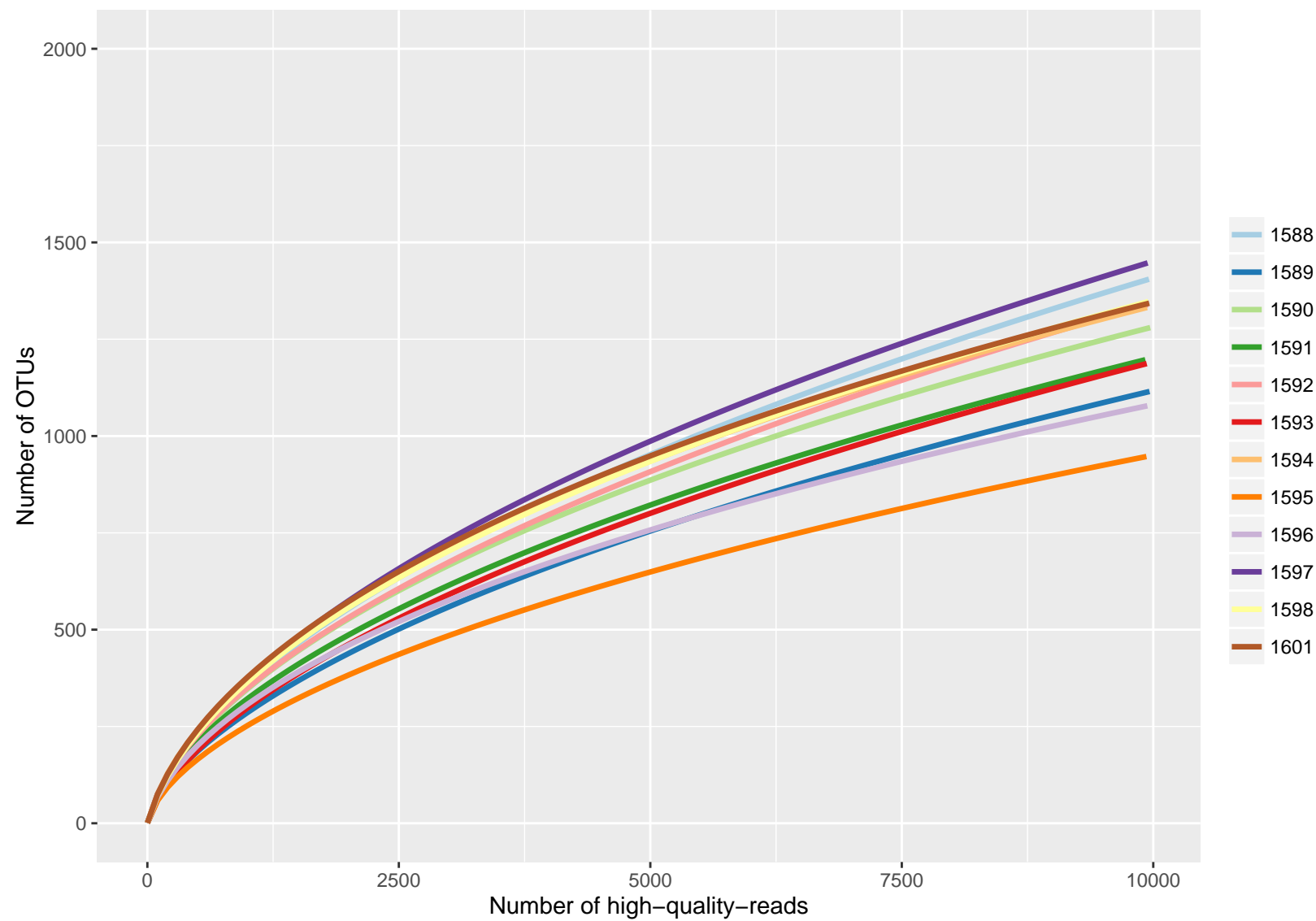

Supplement: S2 File — (ZIP) [file pone.0186766.s008.zip › Rarefact_curves_104.pdf]

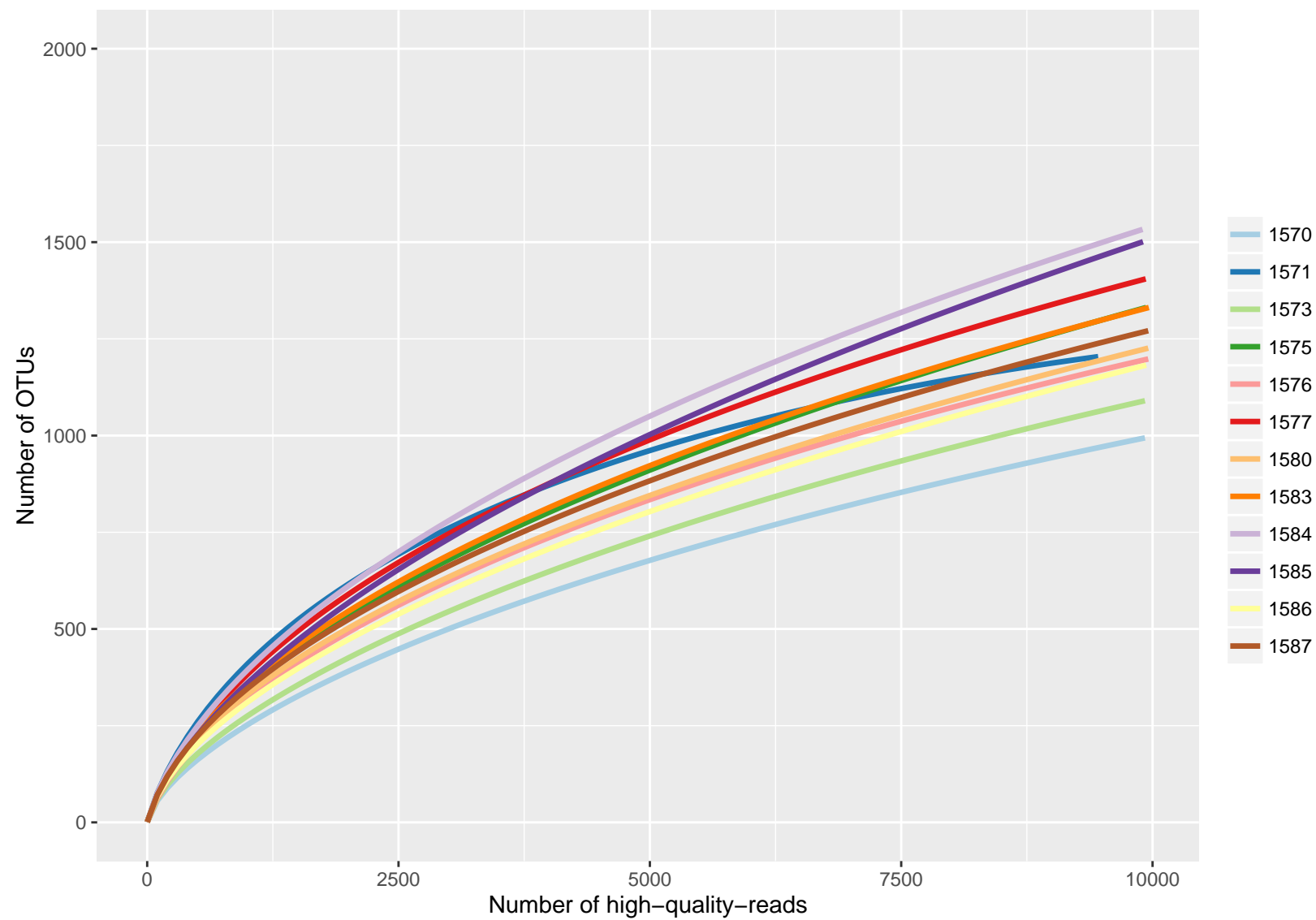

Supplement: S2 File — (ZIP) [file pone.0186766.s008.zip › Rarefact_curves_103.pdf]

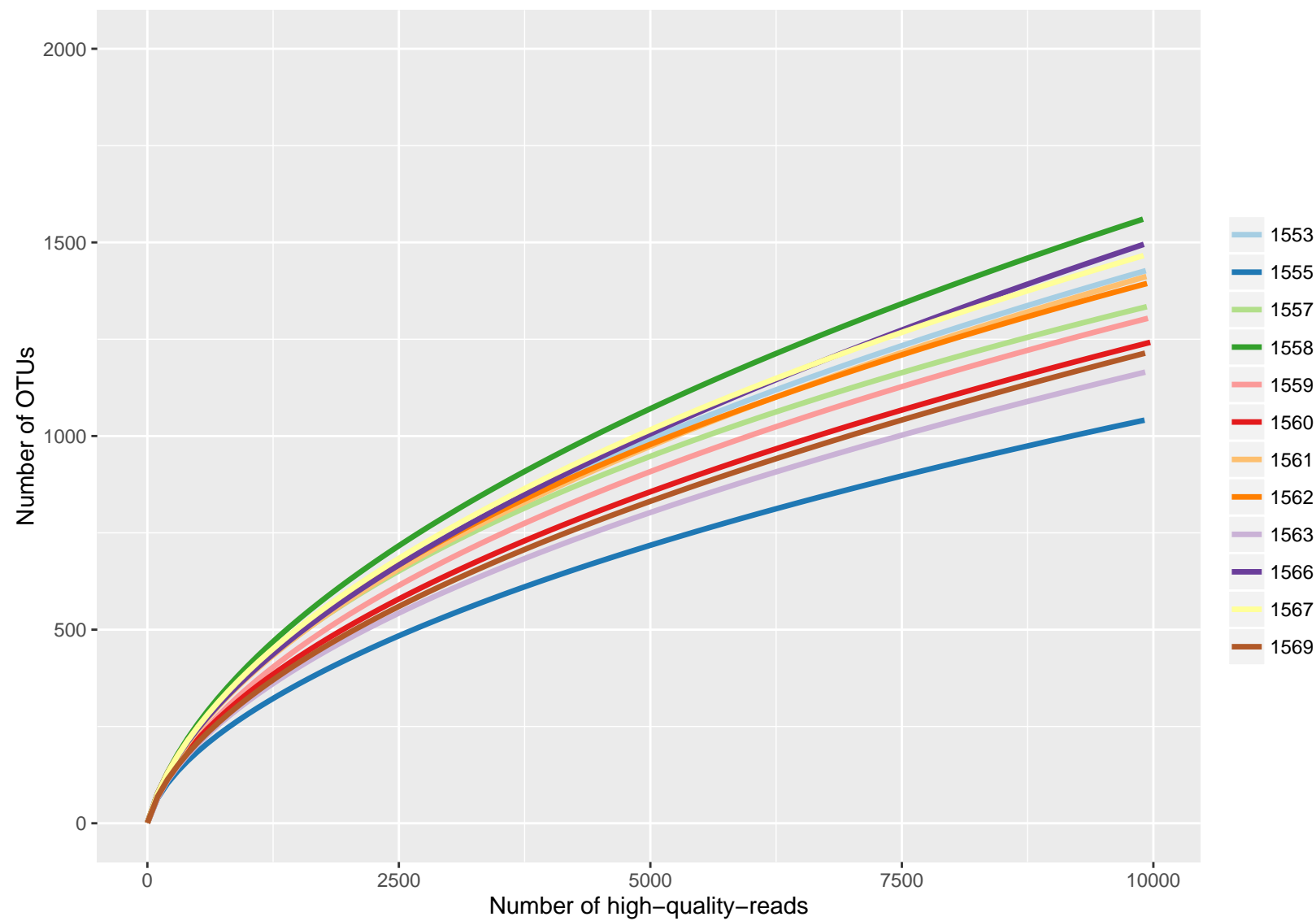

Supplement: S2 File — (ZIP) [file pone.0186766.s008.zip › Rarefact_curves_102.pdf]

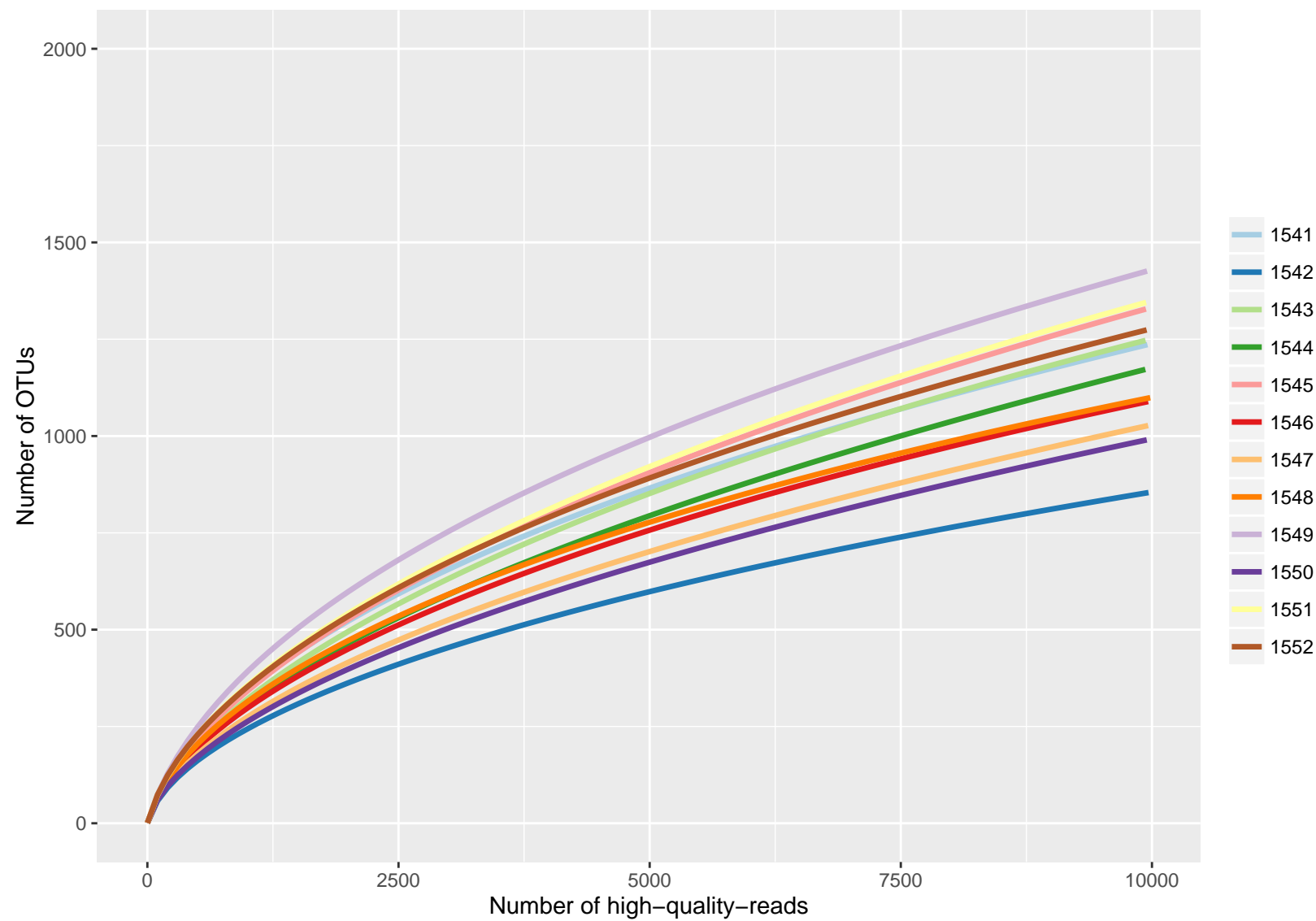

Supplement: S2 File — (ZIP) [file pone.0186766.s008.zip › Rarefact_curves_101.pdf]

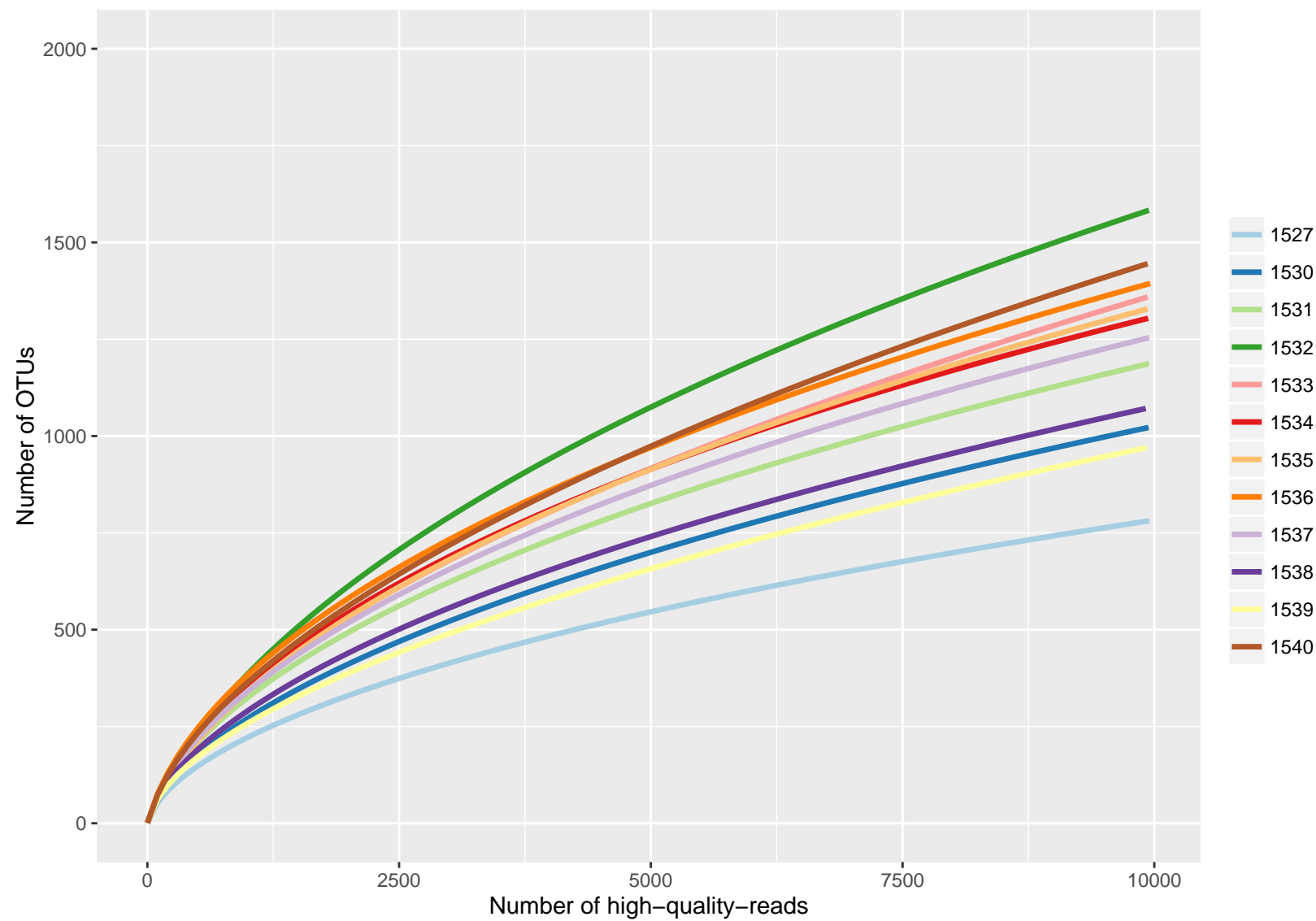

Supplement: S2 File — (ZIP) [file pone.0186766.s008.zip › Rarefact_curves_100.pdf]

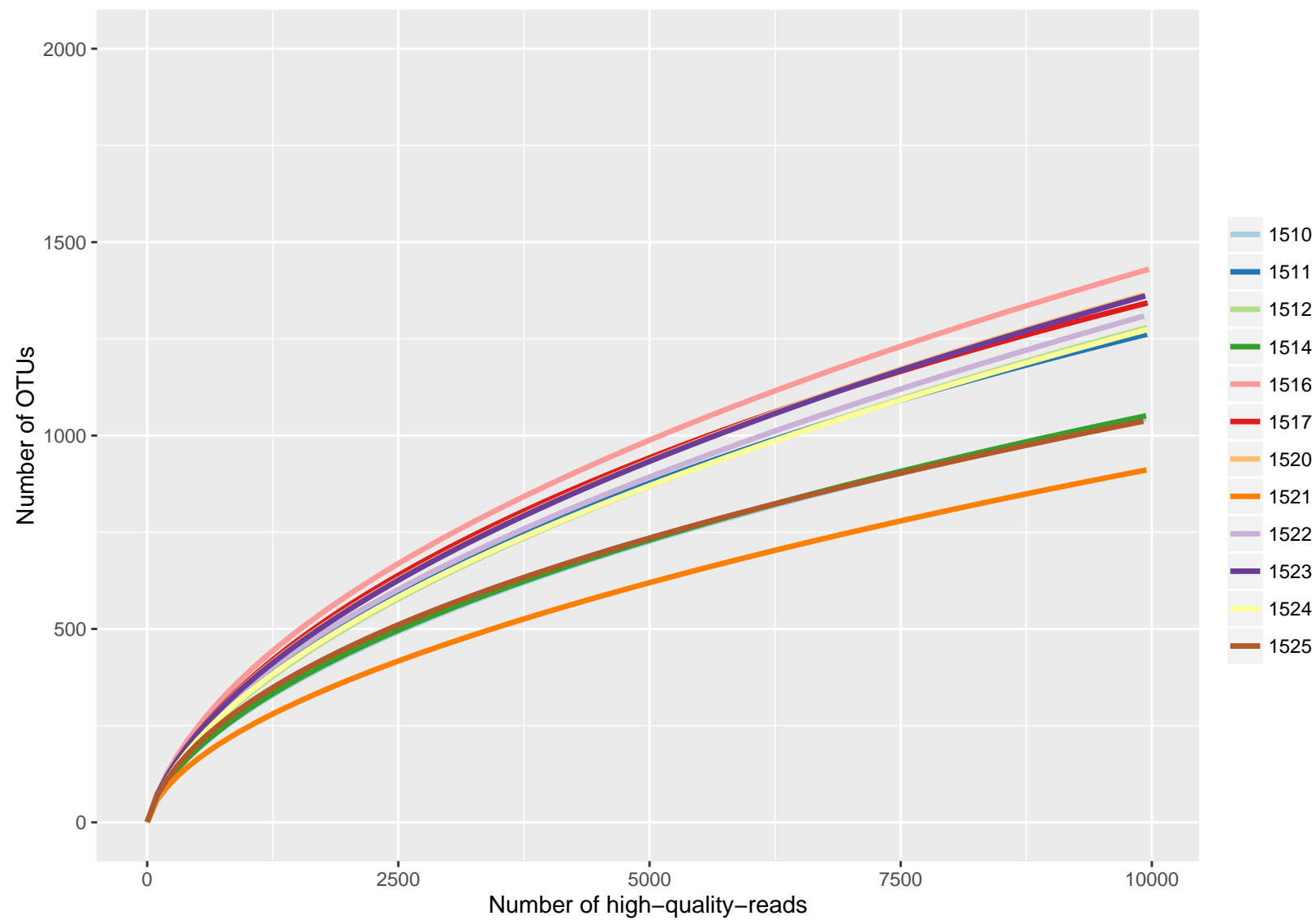

Supplement: S2 File — (ZIP) [file pone.0186766.s008.zip › Rarefact_curves_99.pdf]

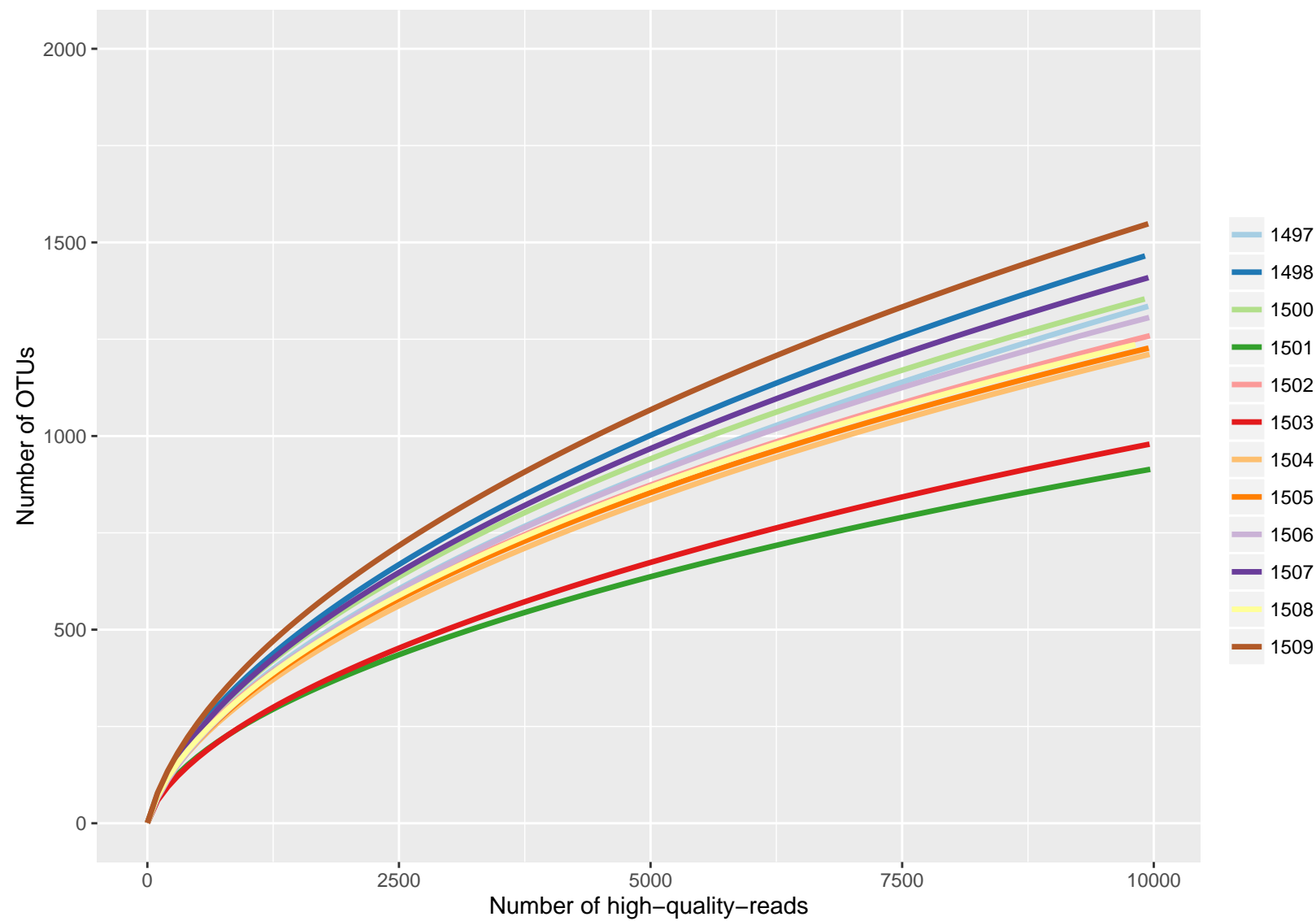

Supplement: S2 File — (ZIP) [file pone.0186766.s008.zip › Rarefact_curves_98.pdf]

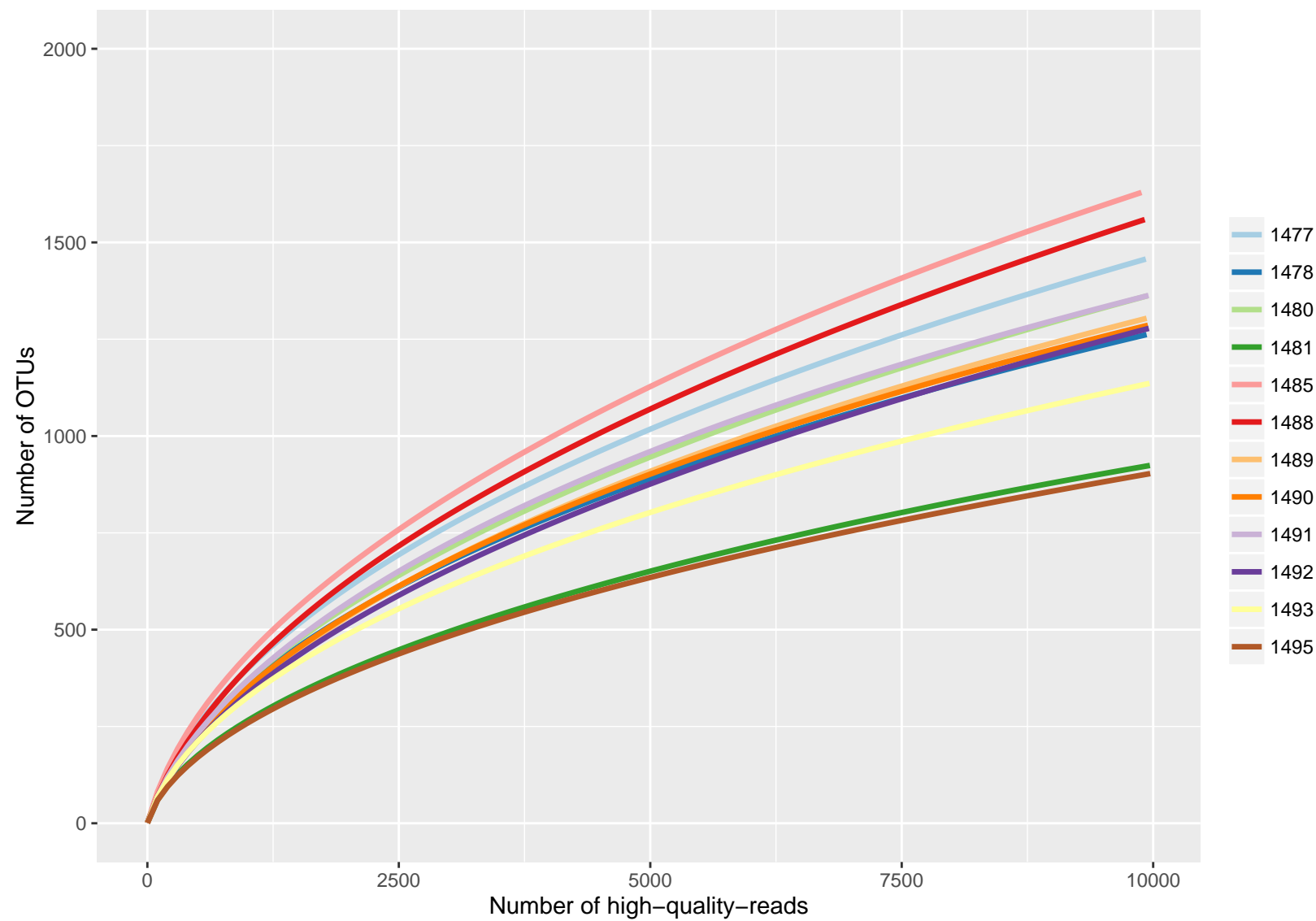

Supplement: S2 File — (ZIP) [file pone.0186766.s008.zip › Rarefact_curves_97.pdf]

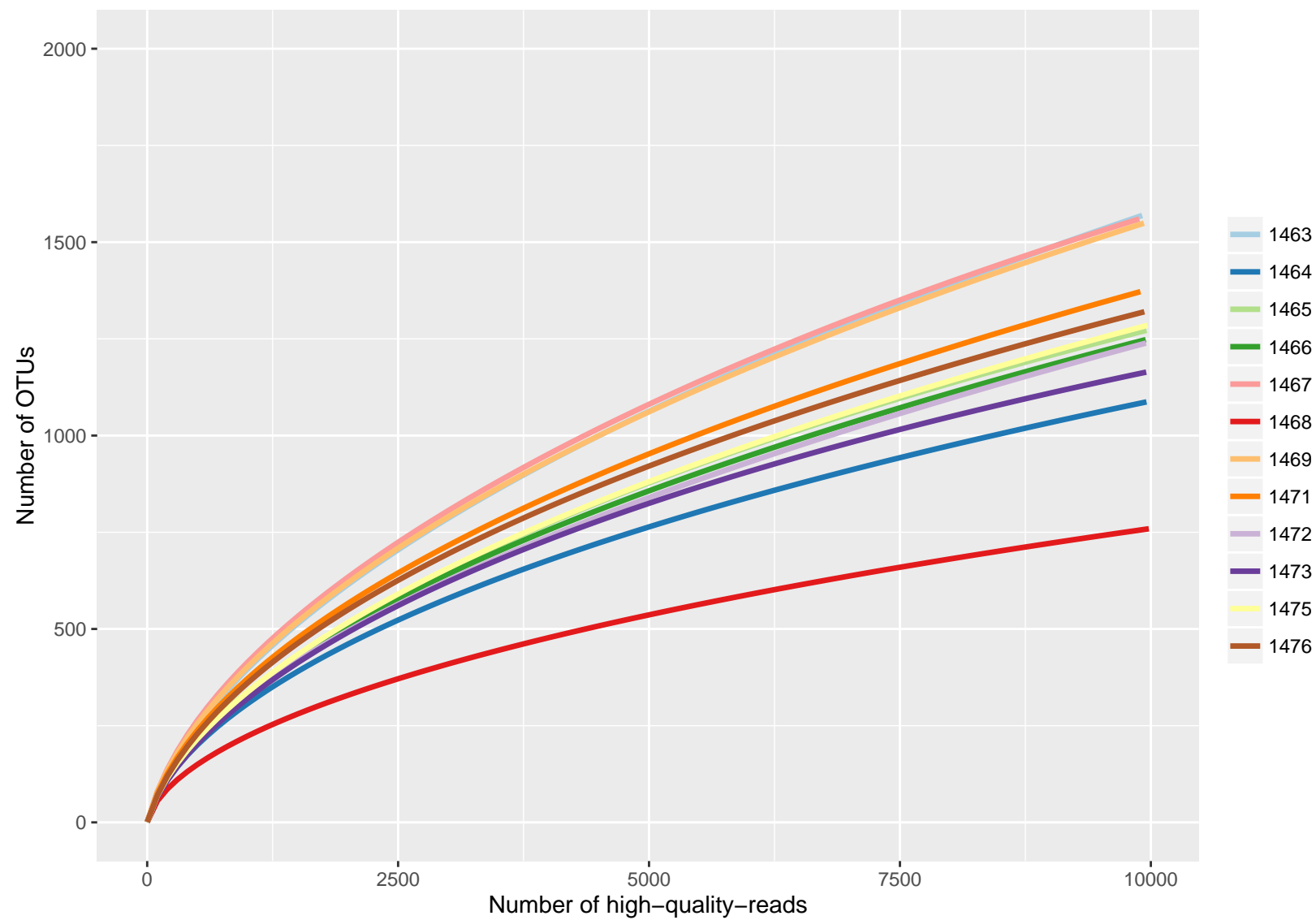

Supplement: S2 File — (ZIP) [file pone.0186766.s008.zip › Rarefact_curves_96.pdf]

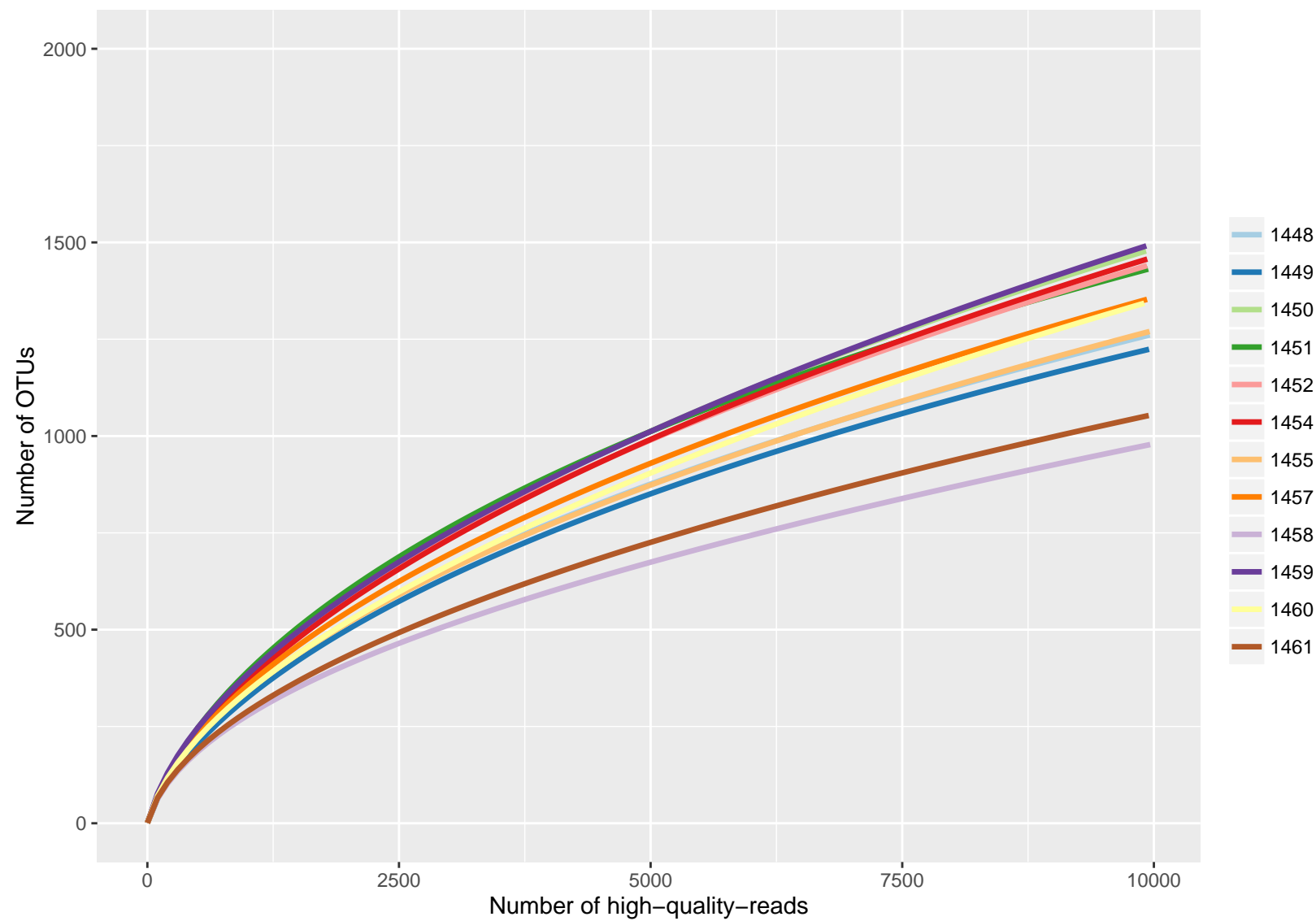

Supplement: S2 File — (ZIP) [file pone.0186766.s008.zip › Rarefact_curves_95.pdf]

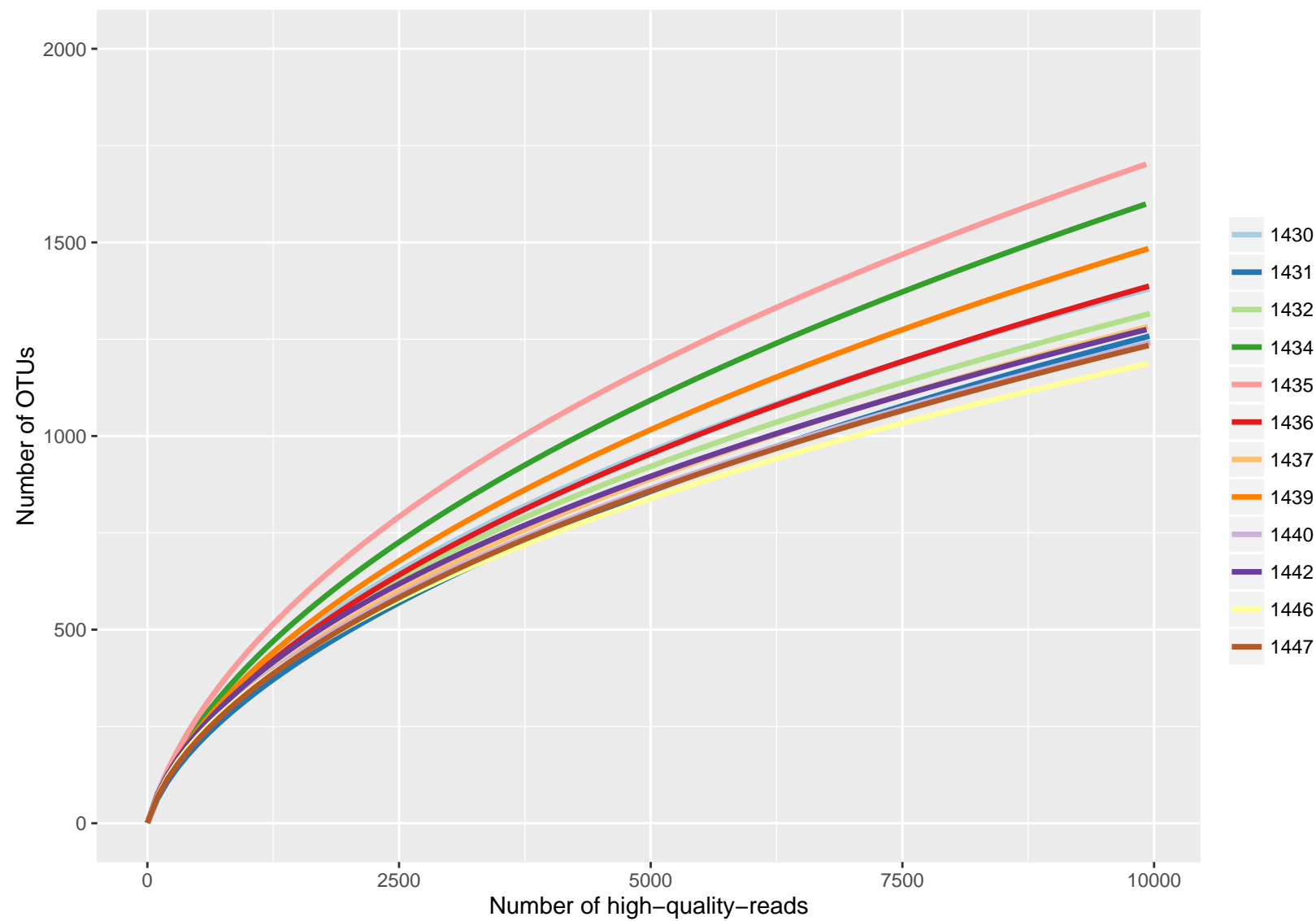

Supplement: S2 File — (ZIP) [file pone.0186766.s008.zip › Rarefact_curves_94.pdf]

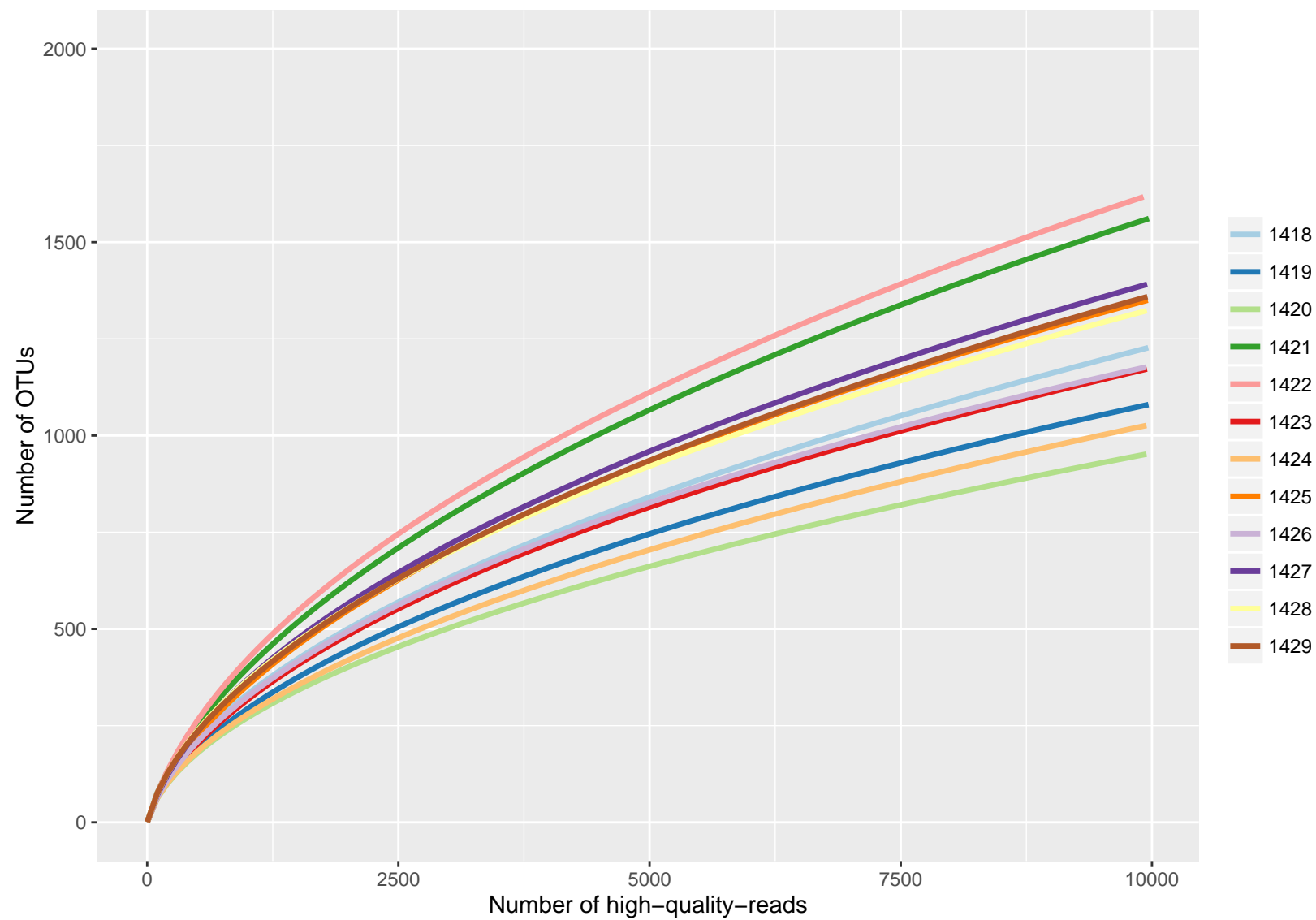

Supplement: S2 File — (ZIP) [file pone.0186766.s008.zip › Rarefact_curves_93.pdf]
