# Supplementary material for: Mapping and predictive variations of soil bacterial richness across France
Source: PLoS One. 2017 Oct 23;12(10):e0186766. doi: 10.1371/journal.pone.0186766 (PMC5653302; doi:10.1371/journal.pone.0186766)
Supplement: S1 Table — (DOCX) [file pone.0186766.s004.docx]

| **STEP** | **PARAMETER DESCRIPTION** | **PARAMETER(S)** |
| --- | --- | --- |
| **PREPROCESSING** | Minimum length threshold | 350 |
|  | Number of ambiguities tolerated | 0 |
|  | Detection of proximal primer sequence | Complete and perfect |
|  | Detection of distal primer sequence | Incomplete, with a maximum of two mismatches |
| **CLUSTERING** | Chosen level of similarity (%) | 95 |
|  | Ignoring differences in homopolymer lengths | Yes |
| **FILTERING** | Chosen clustering similarity threshold | 95 |
|  | Used taxonomic database | SILVA (r114) |
|  | Chosen taxonomic level | Phylum |
|  | Similarity or confidence threshold (%) | 90 |
| **HOMOGENIZATION** | High-quality reads kept for each sample | 10,000 |
| **GLOBAL ANALYSIS** | Chosen level of similarity (%) | 95 |
|  | Ignoring differences in homopolymer lengths | Yes |
