# Supplementary material for: Mapping and predictive variations of soil bacterial richness across France
Source: PLoS One. 2017 Oct 23;12(10):e0186766. doi: 10.1371/journal.pone.0186766 (PMC5653302; doi:10.1371/journal.pone.0186766)
Supplement: S2 Table — This table describes the Coefficients β^ of the fourth degree polynomial model for each of its components. The standard error of each coefficient and its significance is also provided (P < 0.05). (DOCX) [file pone.0186766.s005.docx]

| **Drivers** | **Coefficients $\hat{\beta}$** | **Standard error** | **t-student** | **P values** |
| --- | --- | --- | --- | --- |
| Intercept | 1.044e+03 | 1.254e+02 | 0.083 | 0.933 |
| pH | -2.884e+02 | 6.130e+01 | -0.047 | 0.962 |
| pH^2^ | 3.011e+02 | 1.171e+01 | 0.257 | 0.797 |
| pH^3^ | -5.745e+01 | 1.027e+01 | -0.559 | 0.576 |
| pH^4^ | 3.305e+00 | 3.516e-01 | 0.940 | 0.047 |
| Clay | 2.946e+01 | 1.957e+00 | 1.505 | 0.132 |
| pH*Clay | -8.325e+00 | 6.590e-01 | -1.263 | 0.206 |
| pH^2^*Clay | 6.718e-01 | 8.296e-01 | 0.810 | 0.418 |
| pH^3^*Clay | -7.698e-03 | 3.853e-02 | -0.200 | 0.841 |
| Clay^2^ | -4.574e-02 | 2.051e-02 | -2.230 | 0.025 |
| pH*Clay^2^ | 6.733e-03 | 3.524e-03 | 1.910 | 0.056 |
| pH^2^*Clay^2^ | -3.323e-04 | 2.184e-04 | -1.521 | 0.128 |
| Clay^3^ | 2.280e-05 | 1.466e-05 | -1.724 | 0.120 |
| pH*Clay^3^ | -1.870e-06 | 1.085e-06 | 0.177 | 0.084 |
| Clay^4^ | 9.276e-10 | 5.241e-09 | -0.186 | 0.859 |
| C:N | -1.735e+02 | 9.335e+02 | -0.111 | 0.852 |
| pH*C:N | -3.641e+01 | 3.281e+02 | 0.590 | 0.911 |
| pH^2^*C:N | 2.398e+01 | 4.065e+01 | -1.115 | 0.555 |
| pH^3^*C:N | -1.988e+00 | 1.783e+00 | -1.225 | 0.264 |
| Clay*C:N | -1.897e+00 | 1.549e+00 | 1.007 | 0.220 |
| pH*Clay*C:N | 3.009e-01 | 2.987e-01 | -1.246 | 0.313 |
| pH^2^*Clay*C:N | -2.210e-02 | 1.774e-02 | 1.977 | 0.212 |
| Clay^2^*C:N | 2.928e-03 | 1.481e-03 | -0.342 | 0.048 |
| pH*Clay^2^*C:N | -3.768e-05 | 1.101e-04 | -2.587 | 0.732 |
| Clay^3^*C:N | -1.546e-06 | 5.976e-07 | 0.164 | 0.009 |
| C:N^2^ | 5.189e+00 | 3.172e+01 | -0.249 | 0.870 |
| pH*C:N^2^ | -1.914e+00 | 7.690e+00 | -0.021 | 0.803 |
| pH^2^*C:N^2^ | -9.851e-03 | 4.681e-03 | 0.745 | 0.983 |
| Clay*C:N^2^ | 4.189e-02 | 5.619e-02 | 0.250 | 0.456 |
| pH*Clay*C:N^2^ | 1.287e-03 | 5.157e-03 | -1.431 | 0.802 |
| Clay^2^*C:N^2^ | -5.711e-05 | 3.991e-05 | -0.176 | 0.152 |
| C:N^3^ | -8.280e-02 | 4.696e-01 | 0.572 | 0.860 |
| pH*C:N^3^ | 3.636e-02 | 6.359e-02 | -0.644 | 0.567 |
| X | -4.884e-04 | 7.579e-04 | -0.178 | 0.519 |
| pH*X | -4.799e-04 | 2.699e-03 | -1.089 | 0.858 |
| pH^2^*X | -1.358e-02 | 1.247e-02 | 0.811 | 0.276 |
| pH^3^*X | 3.213e-03 | 3.961e-03 | -0.844 | 0.417 |
| Clay*X | -3.992e-04 | 4.729e-04 | 0.771 | 0.398 |
| pH*Clay*X | 1.620e-05 | 2.102e-05 | 1.248 | 0.441 |
| Clay^2^*X | 2.305e-05 | 1.846e-05 | -0.297 | 0.212 |
| Clay^3^*X | -1.001e-06 | 3.369e-06 | -0.265 | 0.766 |
| C:N*X | -4.915e-08 | 1.854e-07 | -1.075 | 0.790 |
| pH*C:N*X | -1.533e-08 | 1.426e-08 | 1.190 | 0.282 |
| pH^2^*C:N*X | 1.235e-09 | 1.038e-09 | 0.969 | 0.234 |
| Clay*C:N*X | 5.853e-12 | 6.041e-12 | 1.308 | 0.332 |
| pH*Clay*C:N*X | 1.235e-03 | 9.441e-04 | -1.523 | 0.191 |
| Clay^2^*C:N*X | -2.756e-04 | 1.810e-04 | 2.261 | 0.128 |
| C:N^2^*X | 2.336e-05 | 1.033e-06 | -0.943 | 0.023 |
| pH*C:N^2^*X | -1.204e-06 | 1.276e-06 | -0.559 | 0.345 |
| C:N^3^*X | -5.758e-08 | 1.030e-07 | 0.950 | 0.576 |
| X^2^ | 6.307e-10 | 6.638e-10 | -0.056 | 0.342 |
| pH*X^2^ | -1.487e-06 | 2.650e-05 | -0.113 | 0.955 |
| pH^2^*X^2^ | -2.952e-07 | 2.619e-06 | 0.282 | 0.910 |
| Clay*X^2^ | 8.483e-09 | 3.009e-08 | 0.112 | 0.778 |
| pH*Clay*X^2^ | 2.852e-08 | 2.544e-08 | 0.390 | 0.910 |
| Clay^2^*X^2^ | 2.806e-09 | 7.195e-09 | 0.823 | 0.696 |
| C:N*X^2^ | 1.166e-09 | 1.418e-09 | -1.102 | 0.410 |
| pH*C:N*X^2^ | -1.020e-10 | 9.262e-11 | -1.078 | 0.270 |
| Clay*C:N*X^2^ | -1.131e-11 | 1.049e-11 | 1.160 | 0.281 |
| C:N^2^*X^2^ | 1.009e-12 | 8.700e-13 | -1.098 | 0.246 |
| X^3^ | -5.360e-15 | 4.881e-16 | -0.960 | 0.272 |
| pH*X^3^ | -4.146e-10 | 4.317e-11 | 0.230 | 0.337 |
| C:N*X^3^ | 1.123e-11 | 4.882e-12 | 1.390 | 0.818 |
| X^4^ | 6.064e-13 | 4.362e-14 | -0.317 | 0.164 |
