# Supplementary material for: Mapping and predictive variations of soil bacterial richness across France
Source: PLoS One. 2017 Oct 23;12(10):e0186766. doi: 10.1371/journal.pone.0186766 (PMC5653302; doi:10.1371/journal.pone.0186766)
Supplement: S3 Table — The Standardized Regression Coefficients (SRC) of the variables to which the model is most sensitive are presented here. The variables are organized according to the absolute value of their associated SRC from the highest to the lowest. (DOCX) [file pone.0186766.s006.docx]

| **Parameters** | **SRC** |
| --- | --- |
| Interaction (Clay ^3^, C:N) | 3.54 |
| pH | 2.09 |
| Interaction (C:N, X) | -1.54 |
| Interaction (Clay, pH^2^) | 1.35 |
| pH (cubic effect) | 1.13 |
| Interaction(C:N, pH^2^) | -0.84 |
| Clay | 0.52 |
