# Supplementary figures and images for: Mapping and predictive variations of soil bacterial richness across France (part 2 of 2)
Source: PLoS One. 2017 Oct 23;12(10):e0186766. doi: 10.1371/journal.pone.0186766 (PMC5653302; doi:10.1371/journal.pone.0186766)

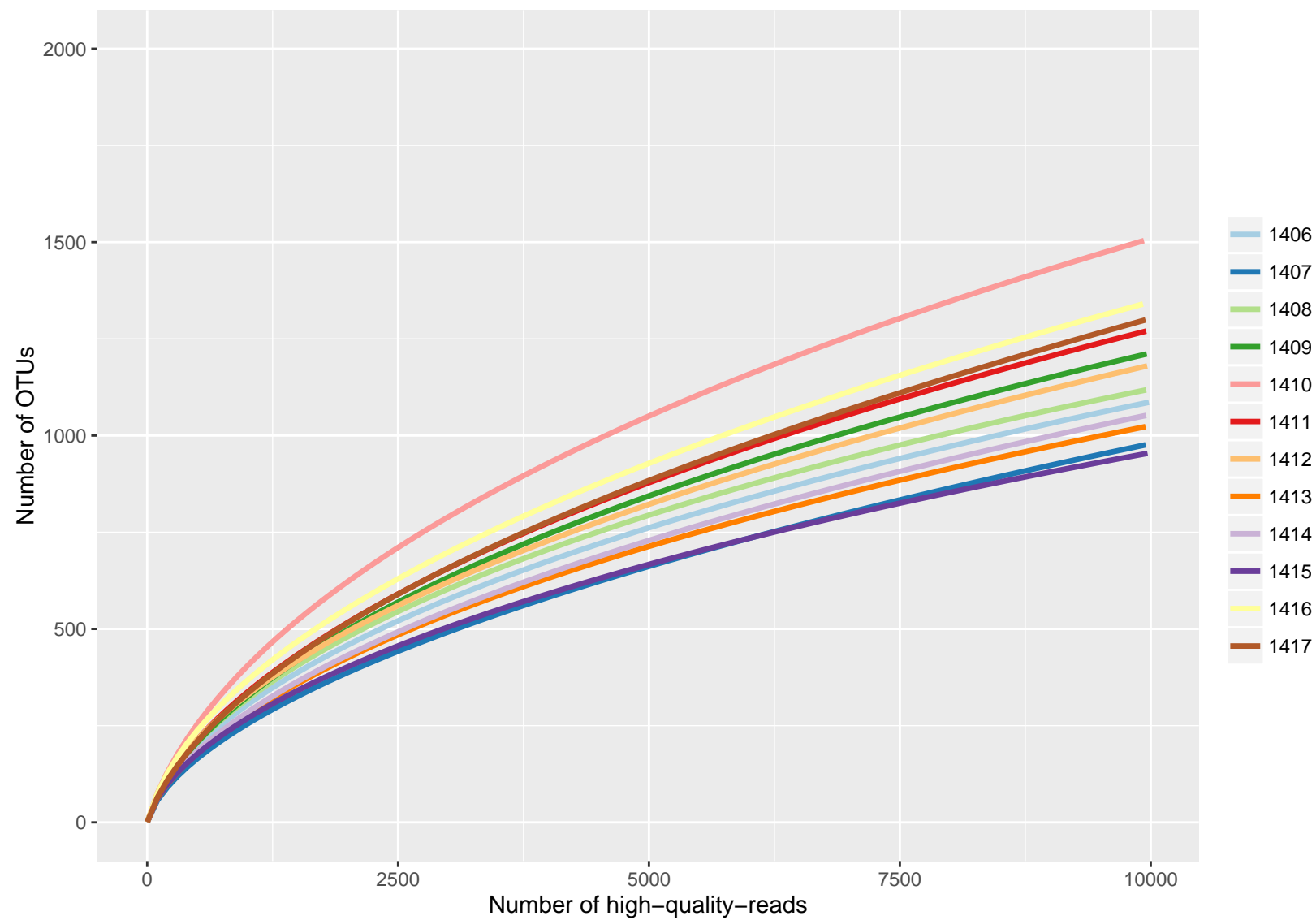

Supplement: S2 File — (ZIP) [file pone.0186766.s008.zip › Rarefact_curves_92.pdf]

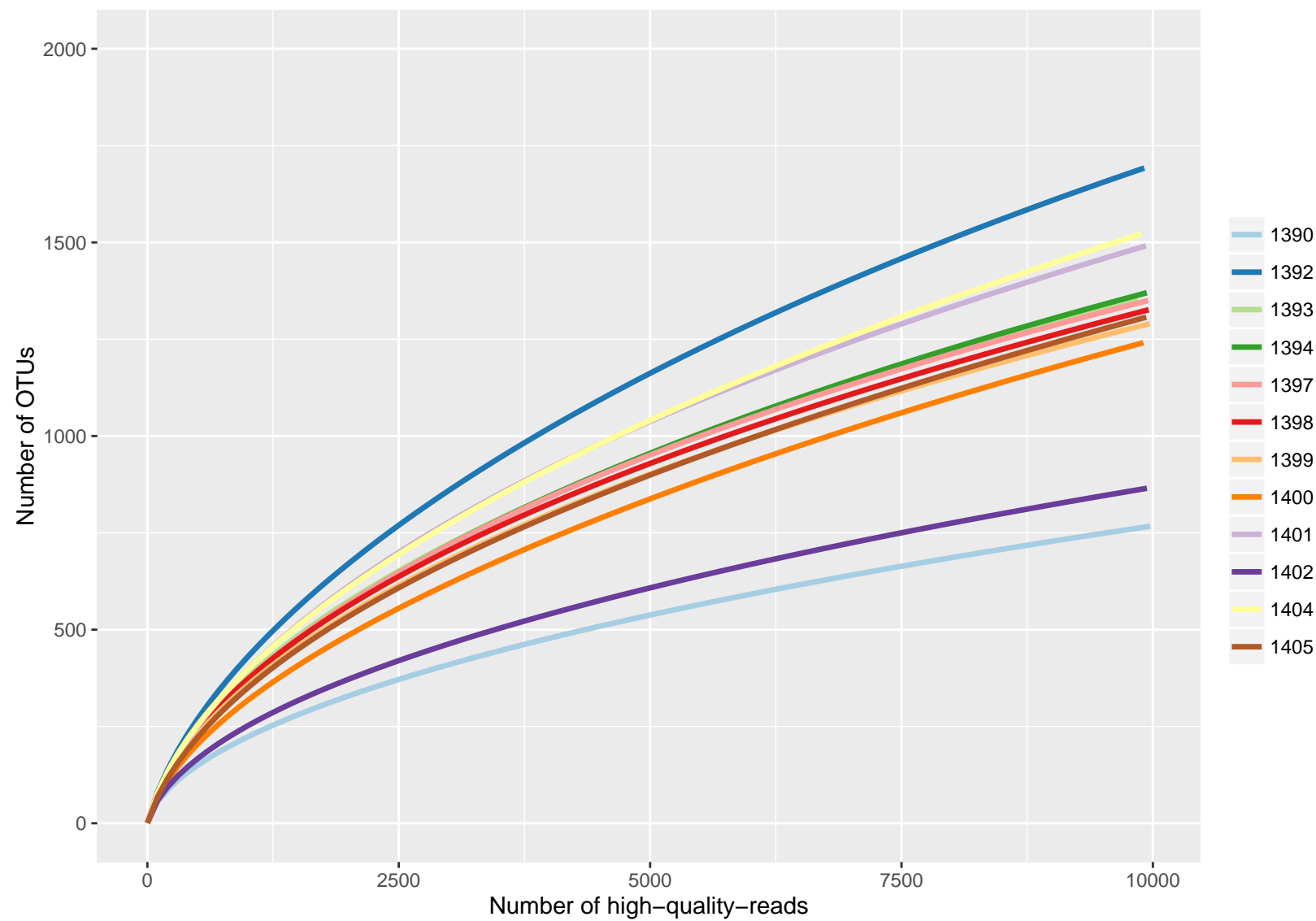

Supplement: S2 File — (ZIP) [file pone.0186766.s008.zip › Rarefact_curves_91.pdf]

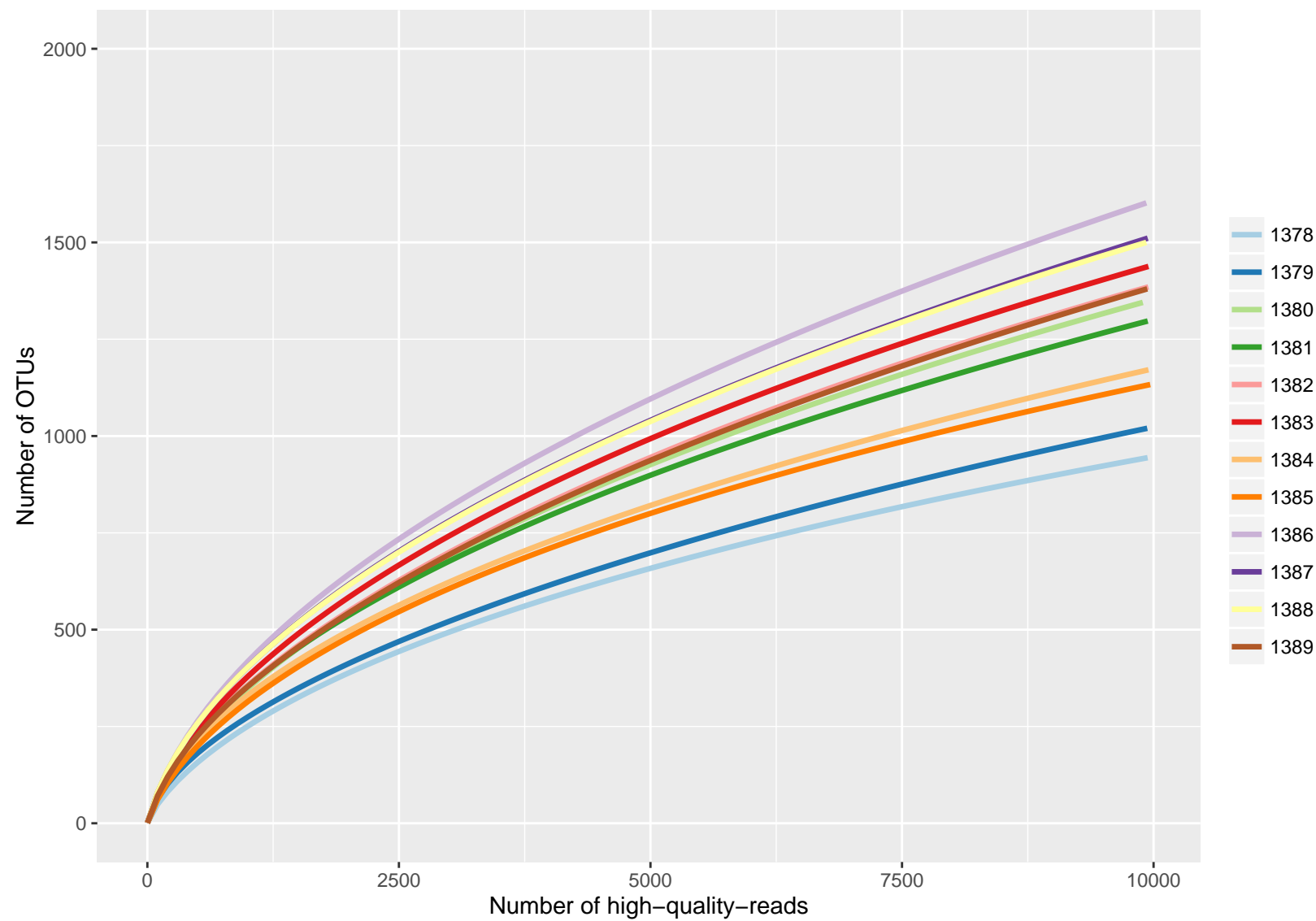

Supplement: S2 File — (ZIP) [file pone.0186766.s008.zip › Rarefact_curves_90.pdf]

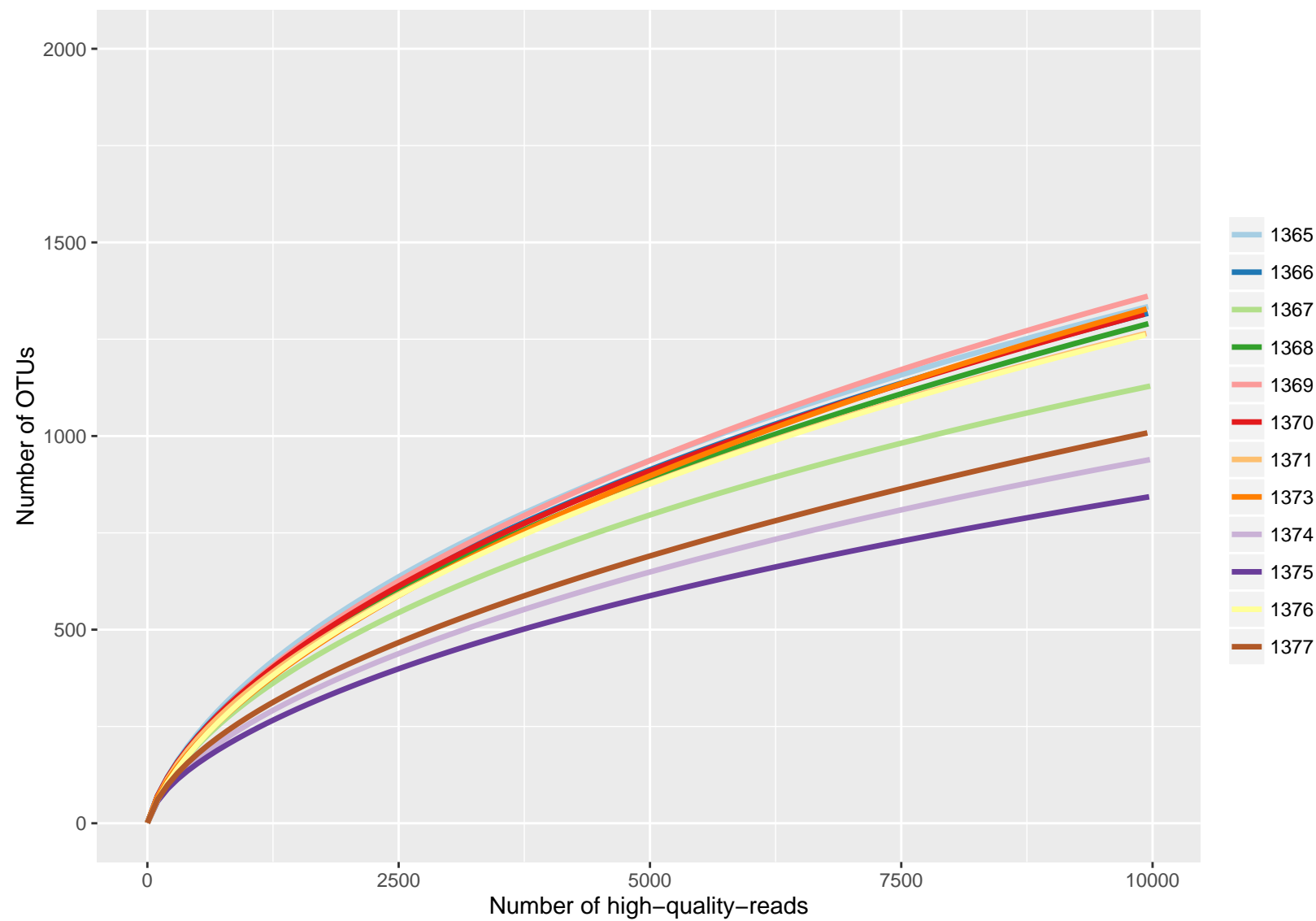

Supplement: S2 File — (ZIP) [file pone.0186766.s008.zip › Rarefact_curves_89.pdf]

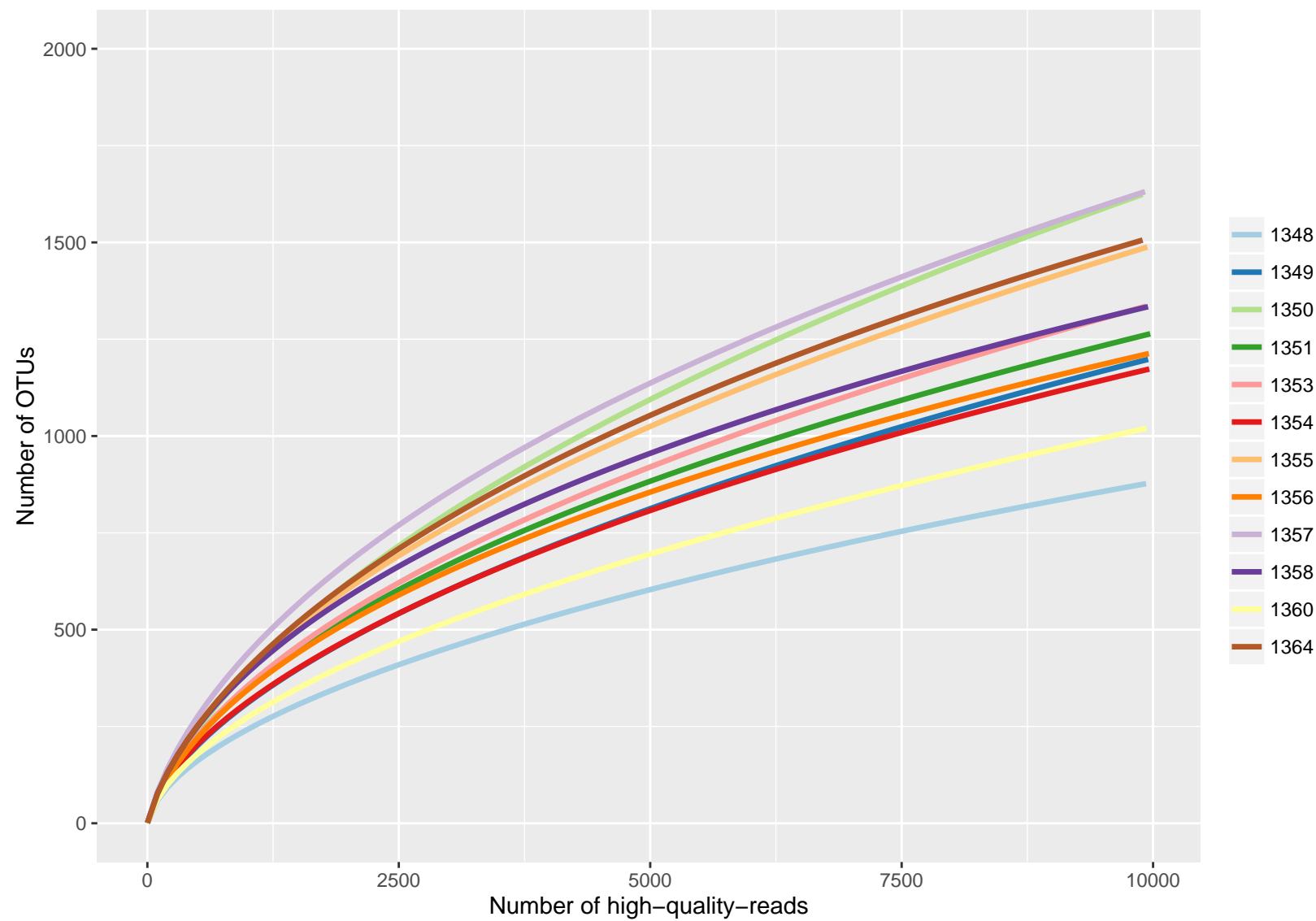

Supplement: S2 File — (ZIP) [file pone.0186766.s008.zip › Rarefact_curves_88.pdf]

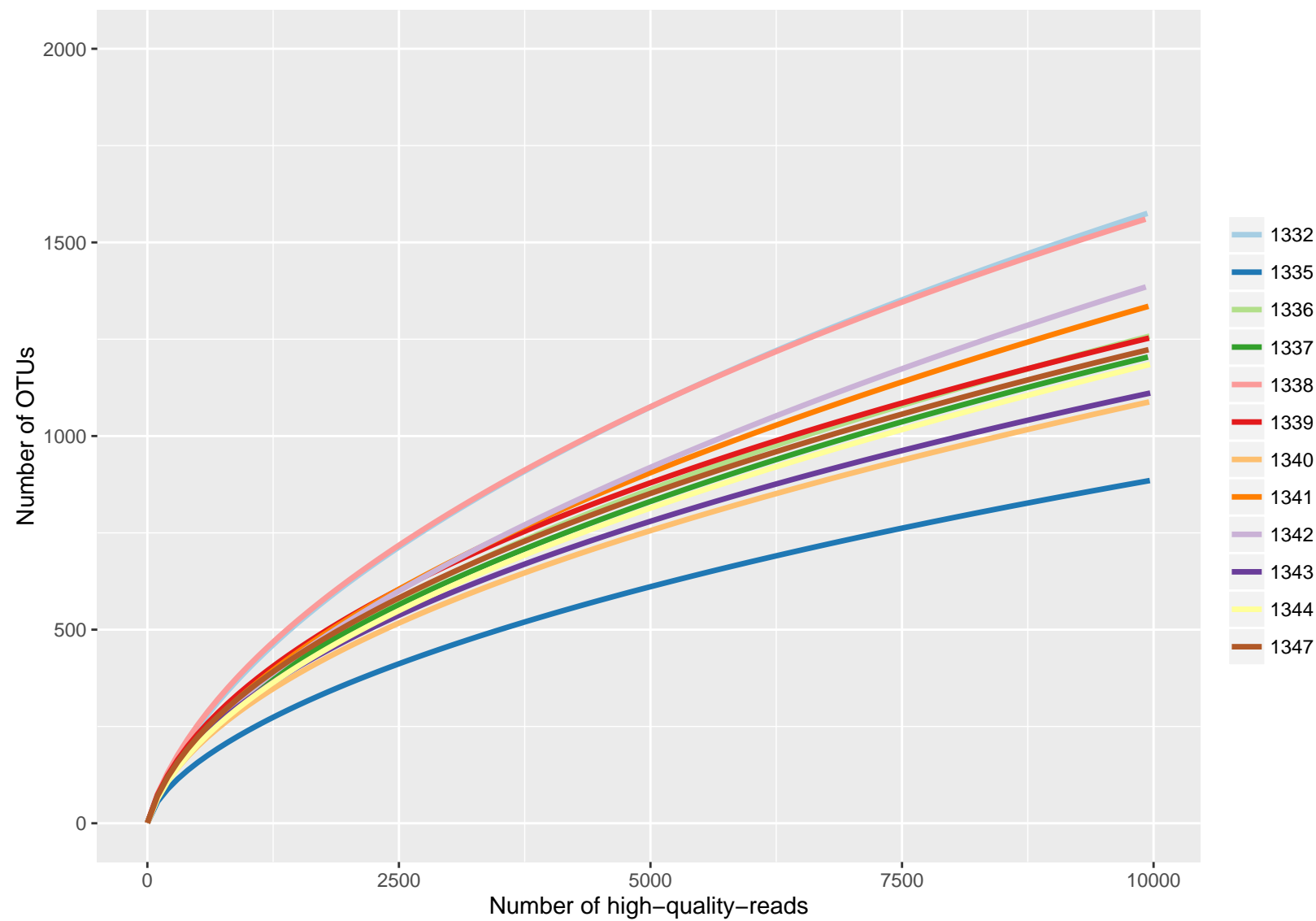

Supplement: S2 File — (ZIP) [file pone.0186766.s008.zip › Rarefact_curves_87.pdf]

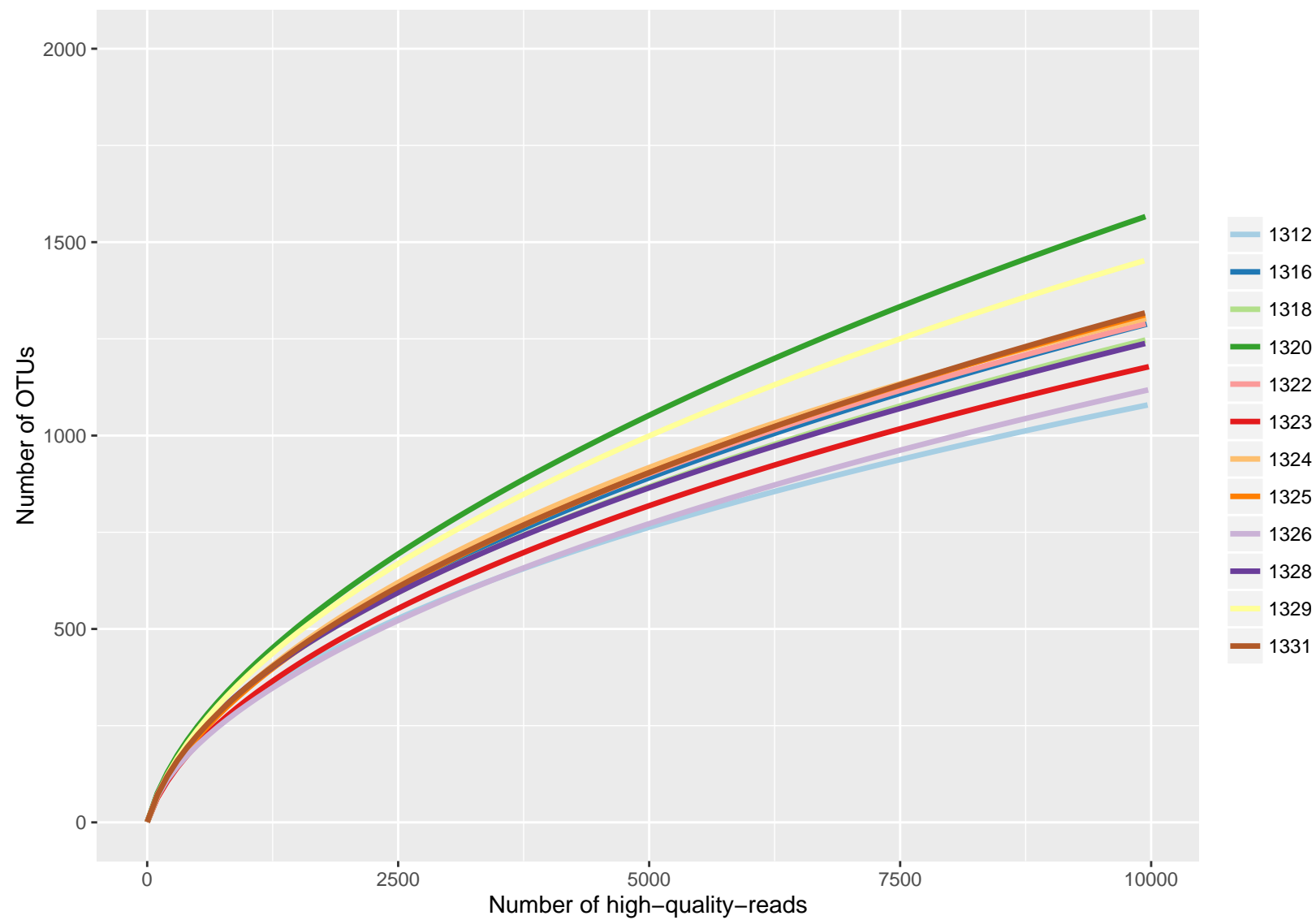

Supplement: S2 File — (ZIP) [file pone.0186766.s008.zip › Rarefact_curves_86.pdf]

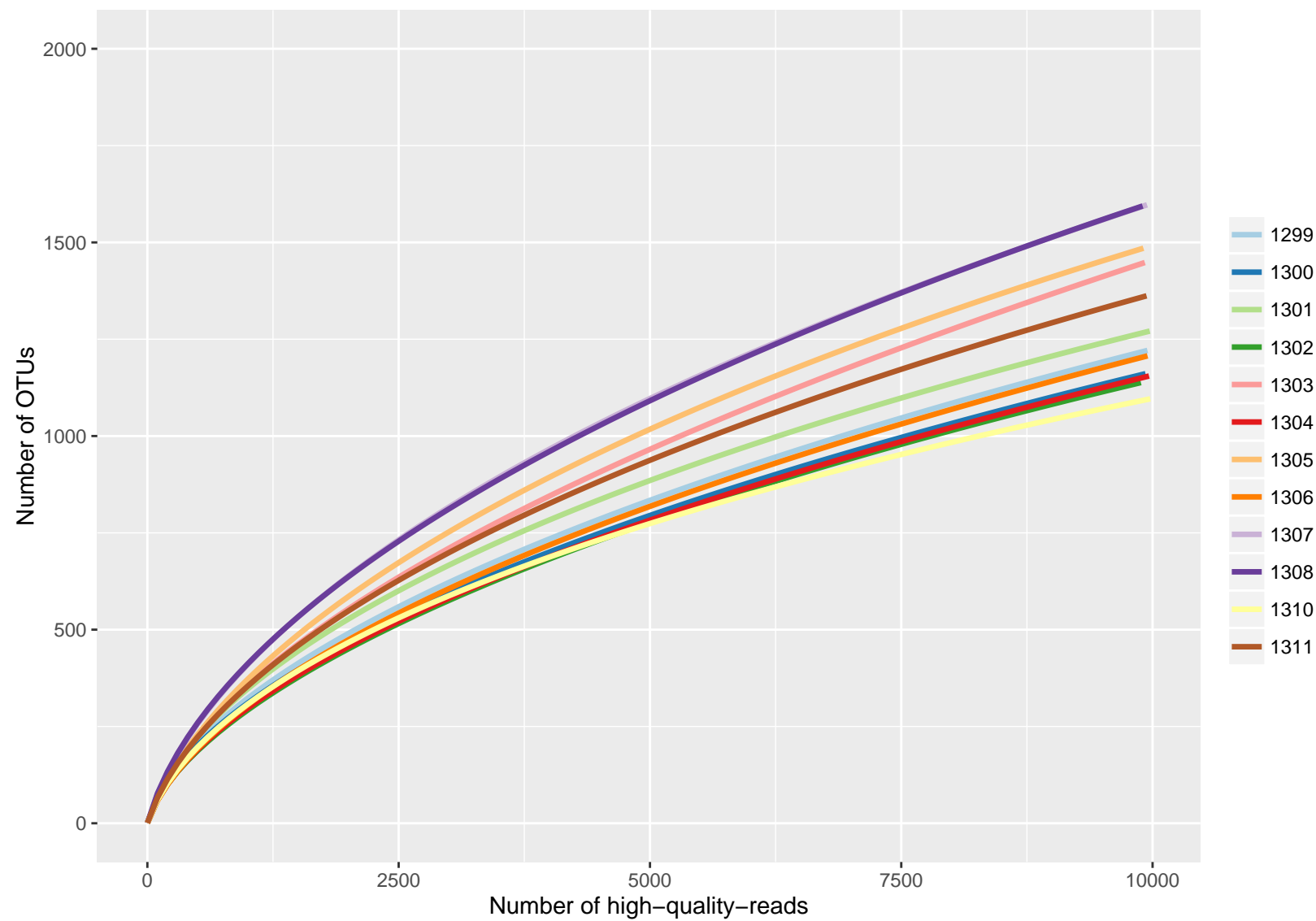

Supplement: S2 File — (ZIP) [file pone.0186766.s008.zip › Rarefact_curves_85.pdf]

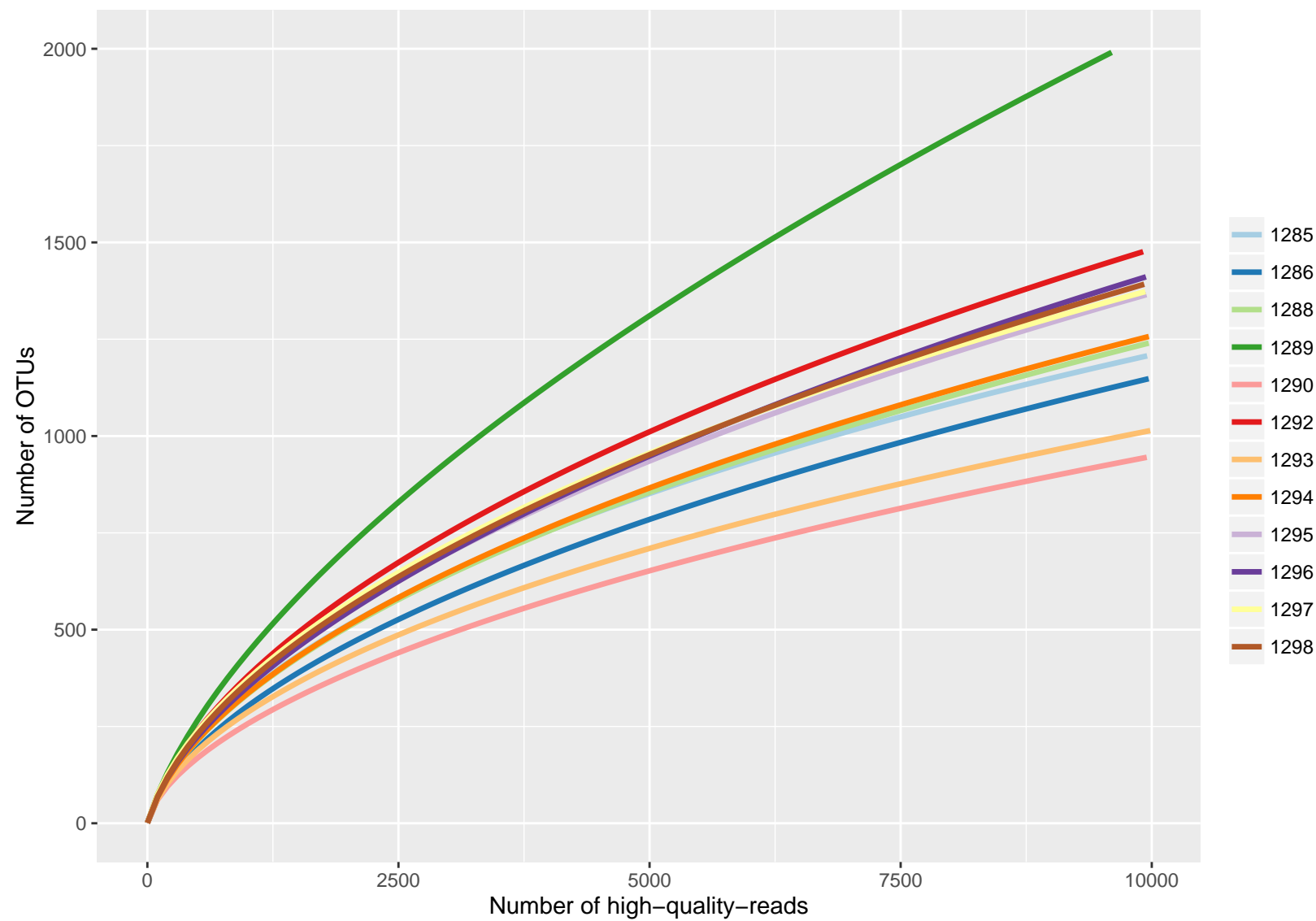

Supplement: S2 File — (ZIP) [file pone.0186766.s008.zip › Rarefact_curves_84.pdf]

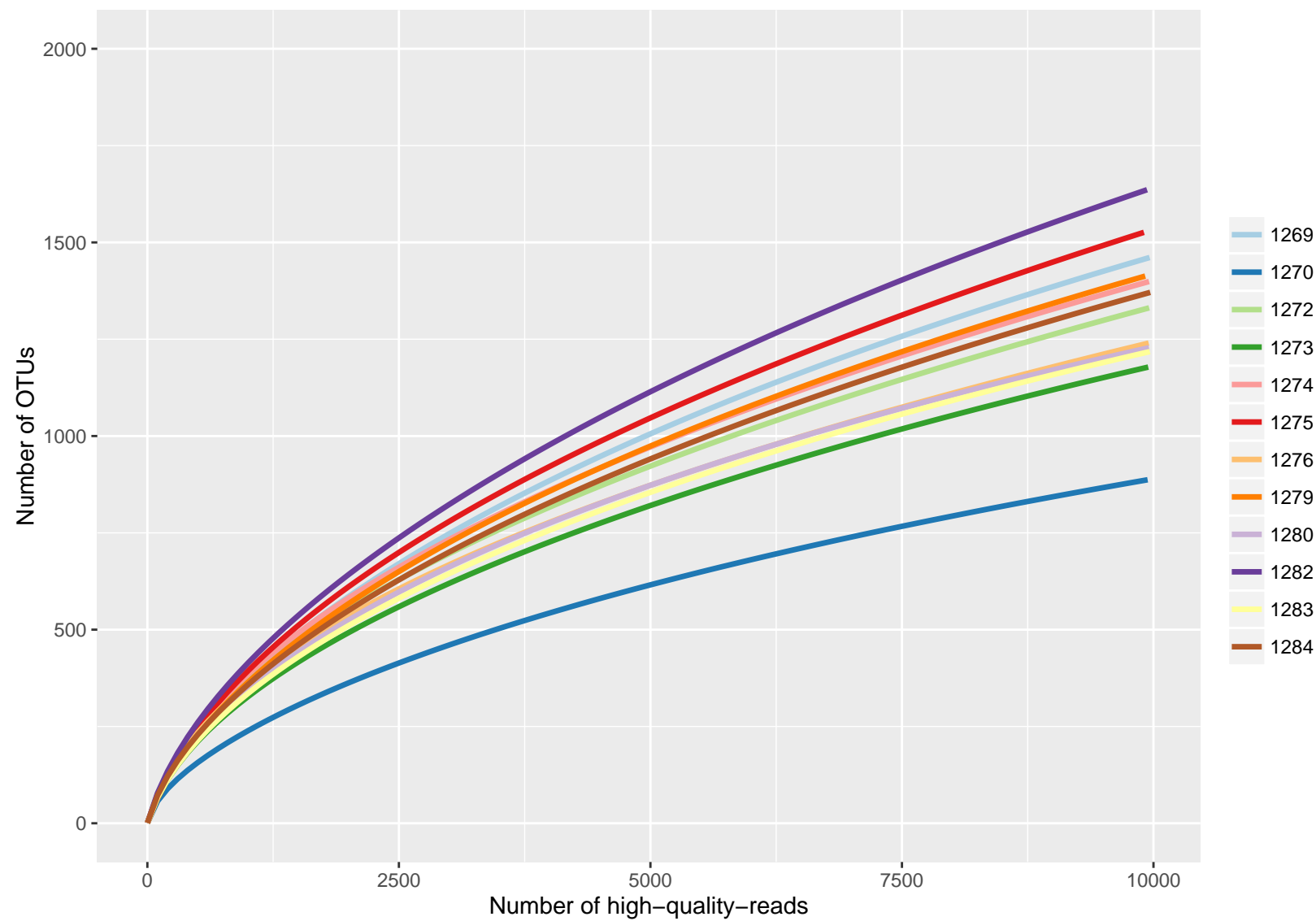

Supplement: S2 File — (ZIP) [file pone.0186766.s008.zip › Rarefact_curves_83.pdf]

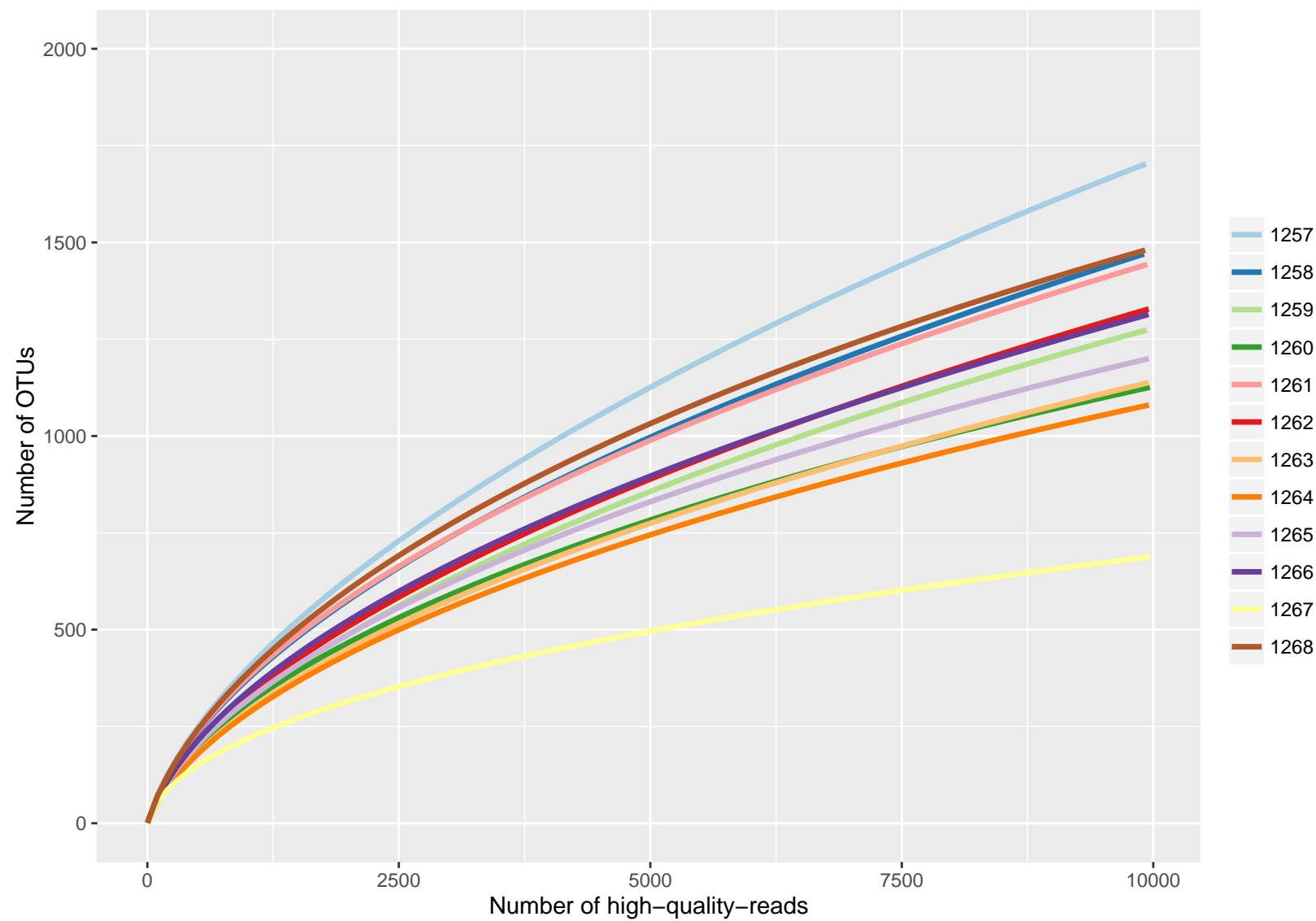

Supplement: S2 File — (ZIP) [file pone.0186766.s008.zip › Rarefact_curves_82.pdf]

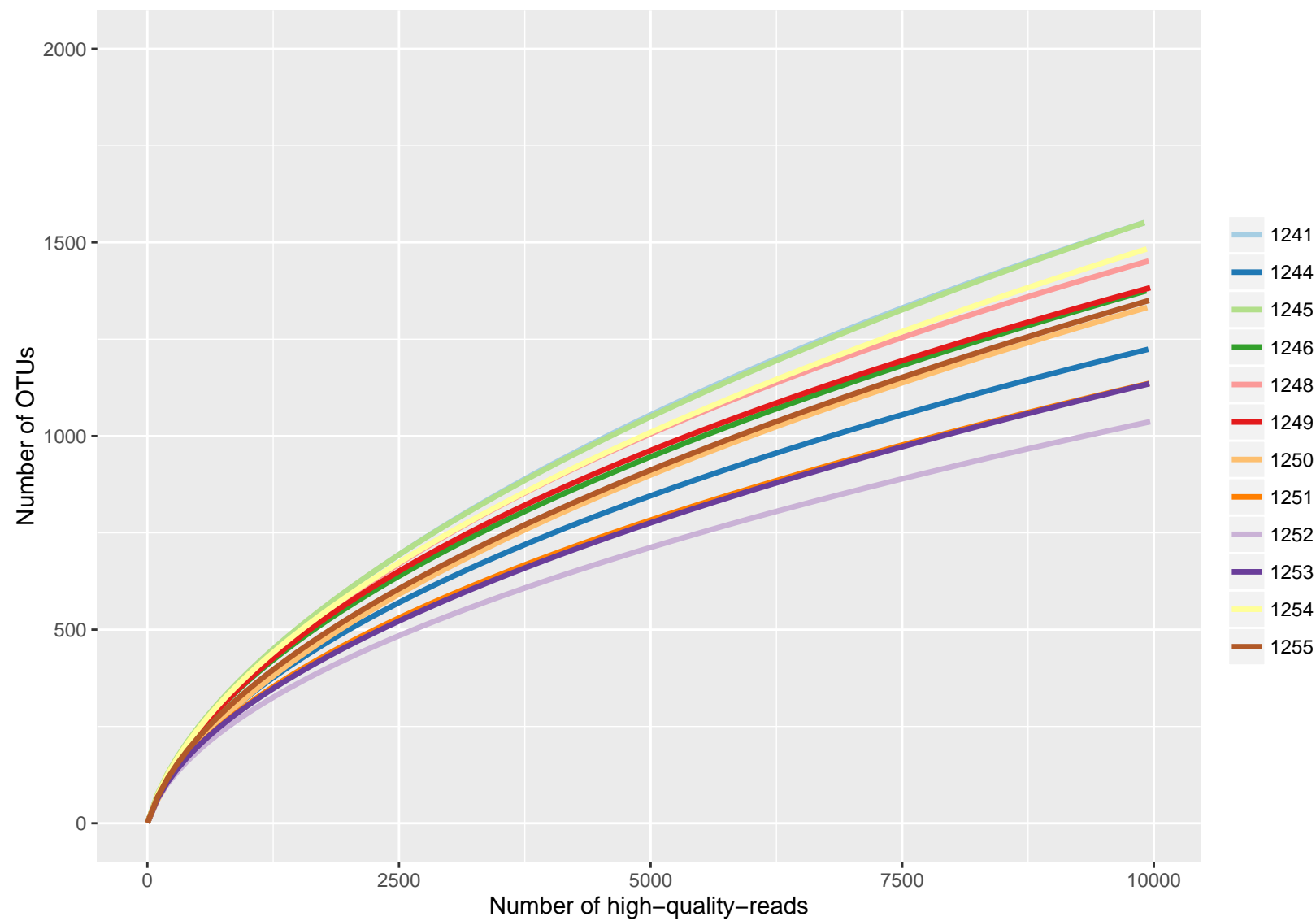

Supplement: S2 File — (ZIP) [file pone.0186766.s008.zip › Rarefact_curves_81.pdf]

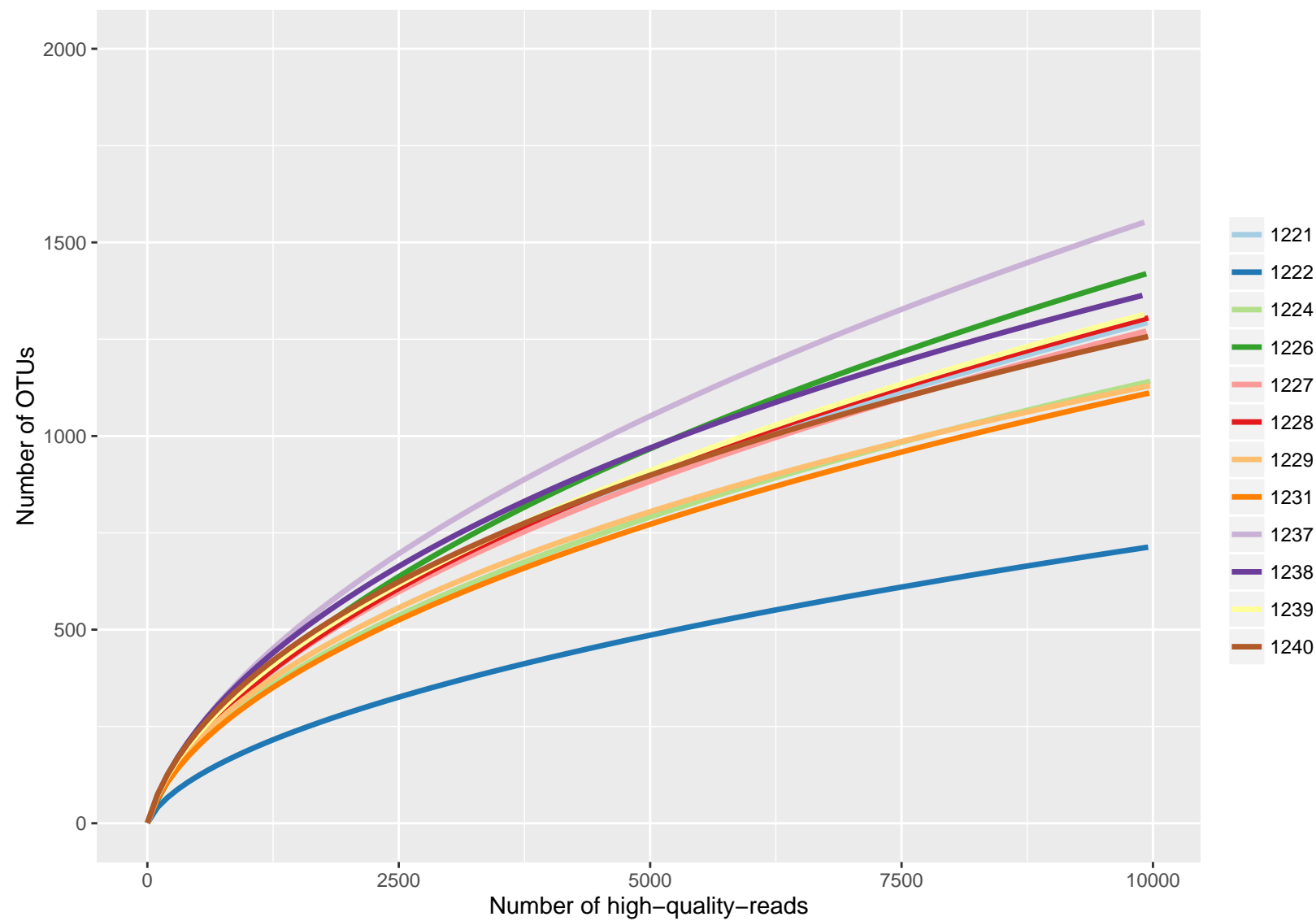

Supplement: S2 File — (ZIP) [file pone.0186766.s008.zip › Rarefact_curves_80.pdf]

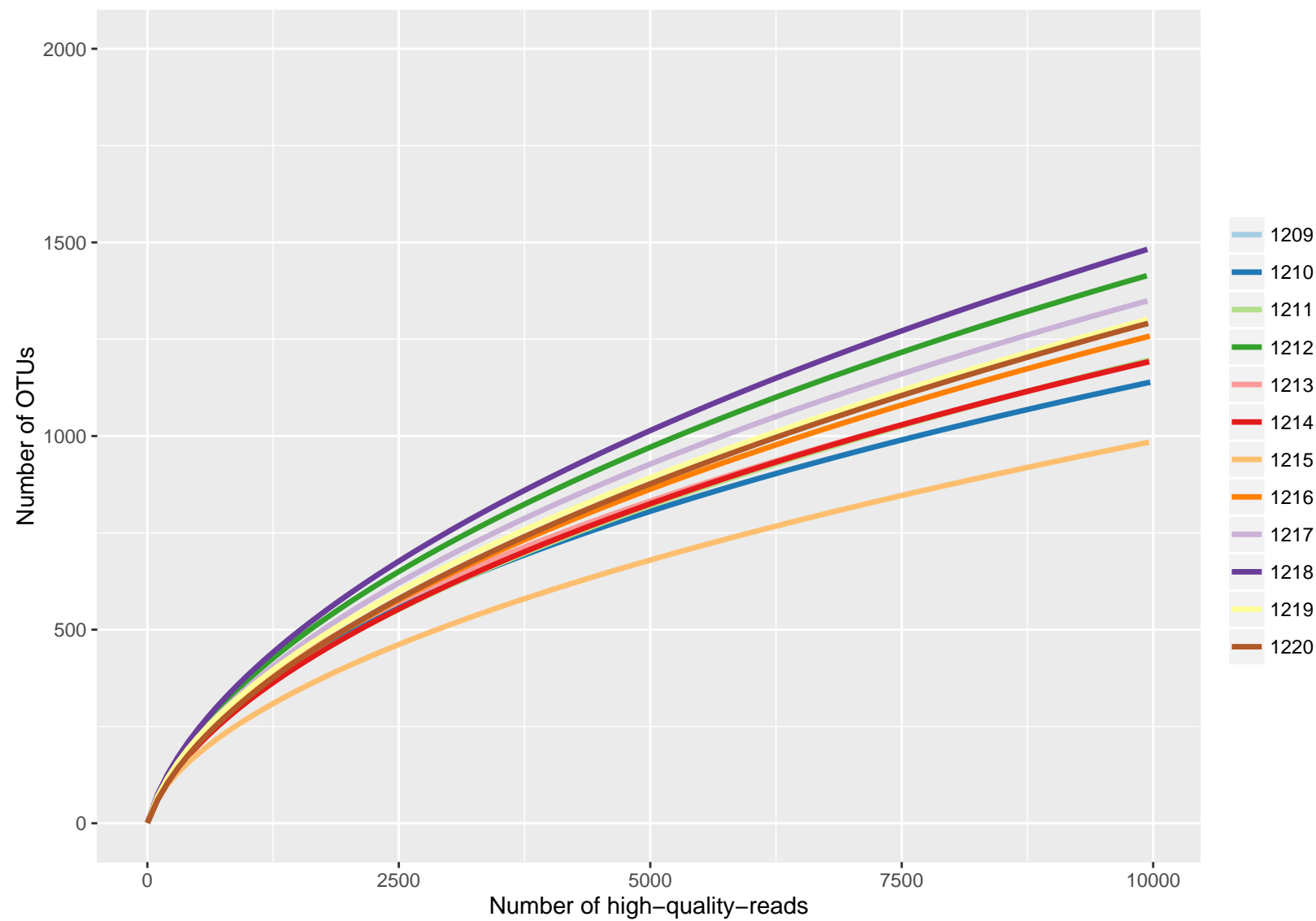

Supplement: S2 File — (ZIP) [file pone.0186766.s008.zip › Rarefact_curves_79.pdf]

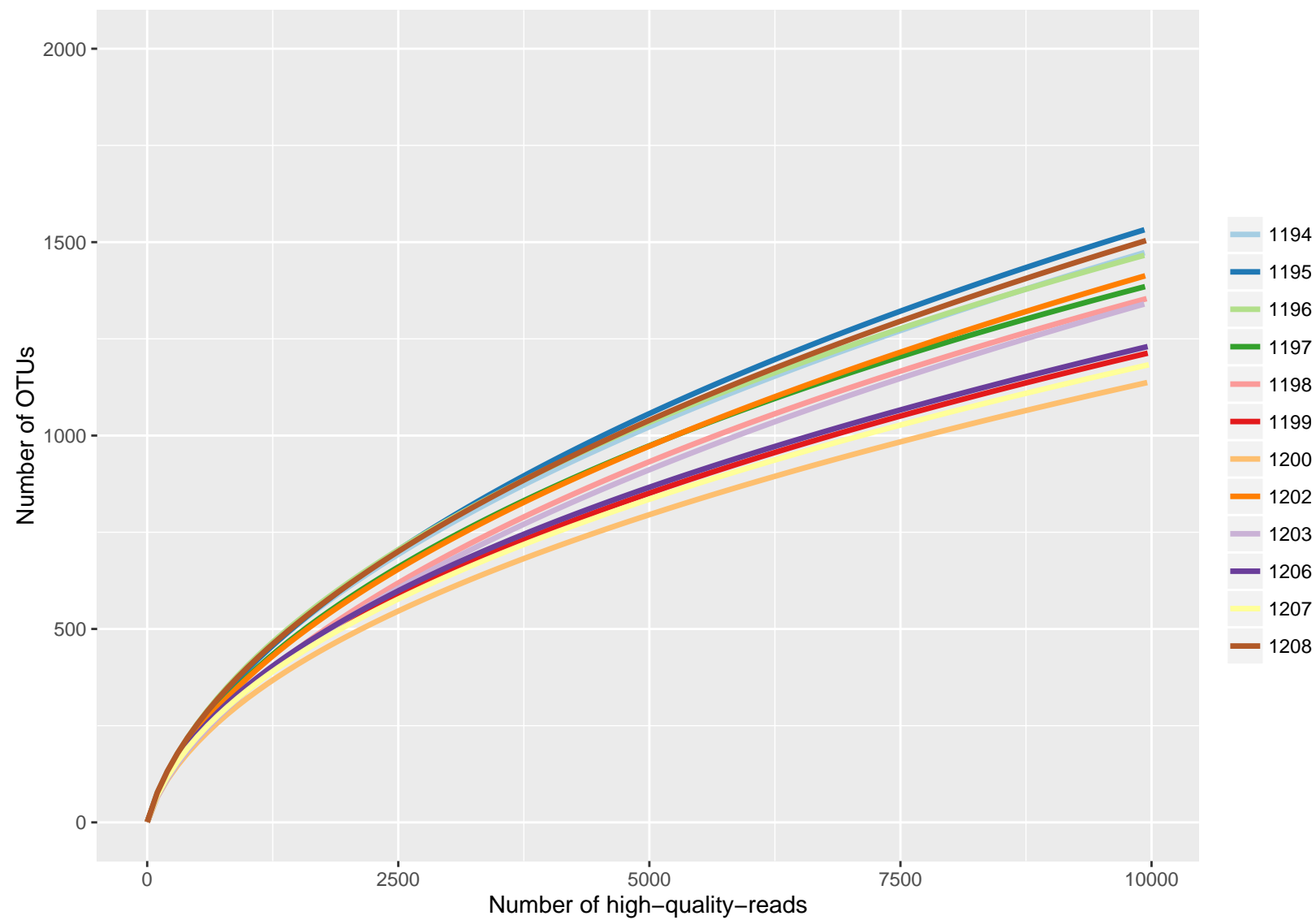

Supplement: S2 File — (ZIP) [file pone.0186766.s008.zip › Rarefact_curves_78.pdf]

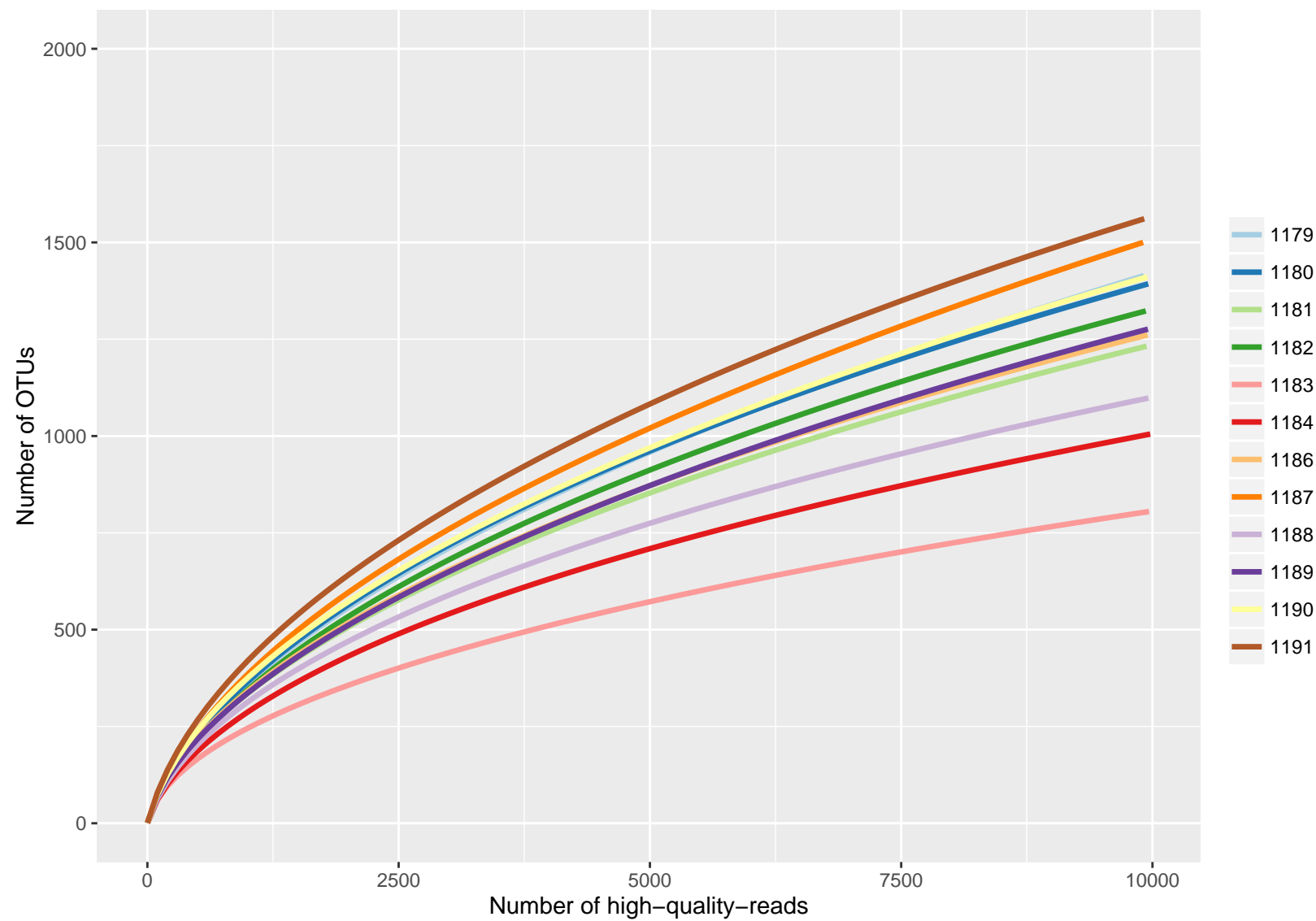

Supplement: S2 File — (ZIP) [file pone.0186766.s008.zip › Rarefact_curves_77.pdf]

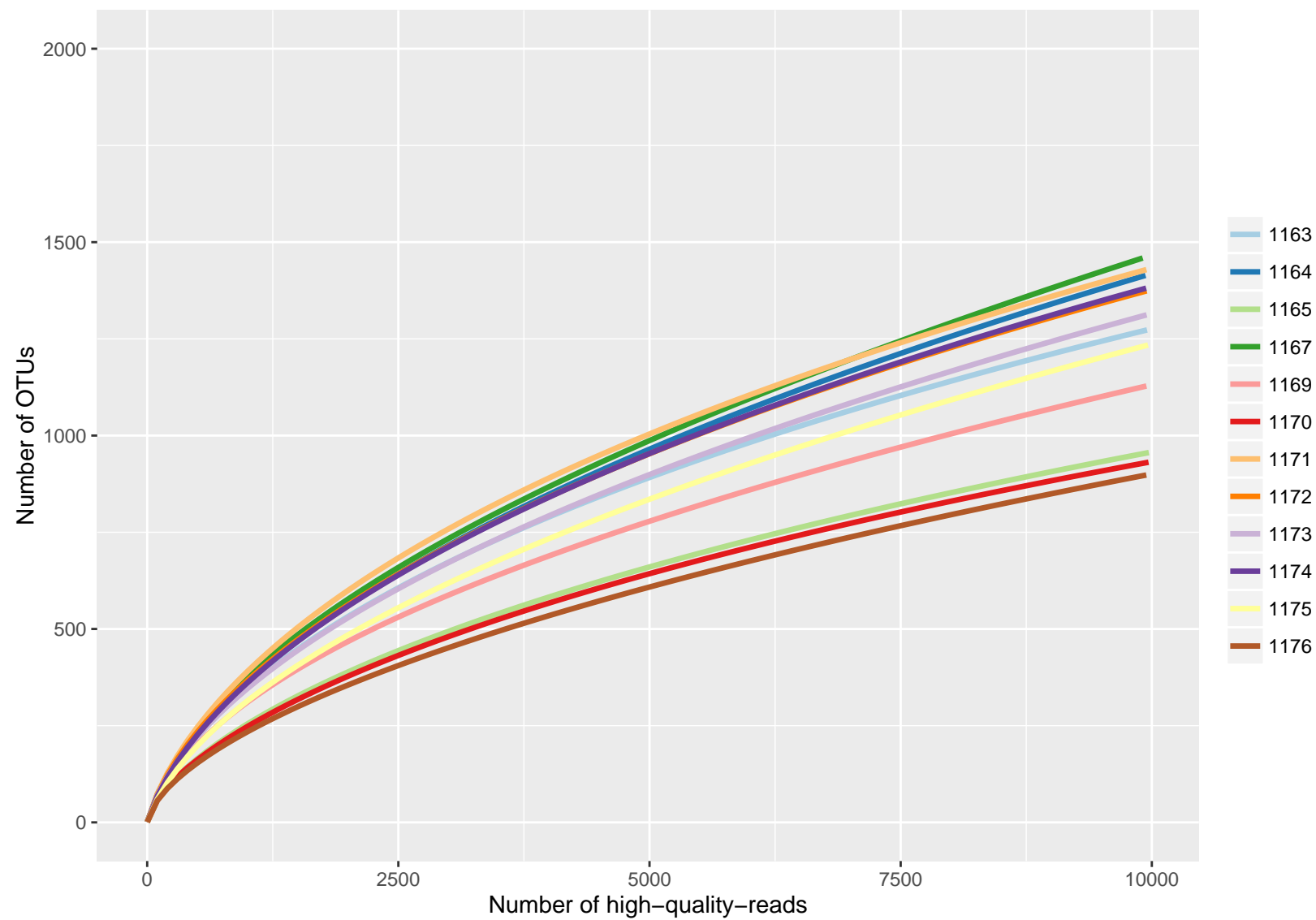

Supplement: S2 File — (ZIP) [file pone.0186766.s008.zip › Rarefact_curves_76.pdf]

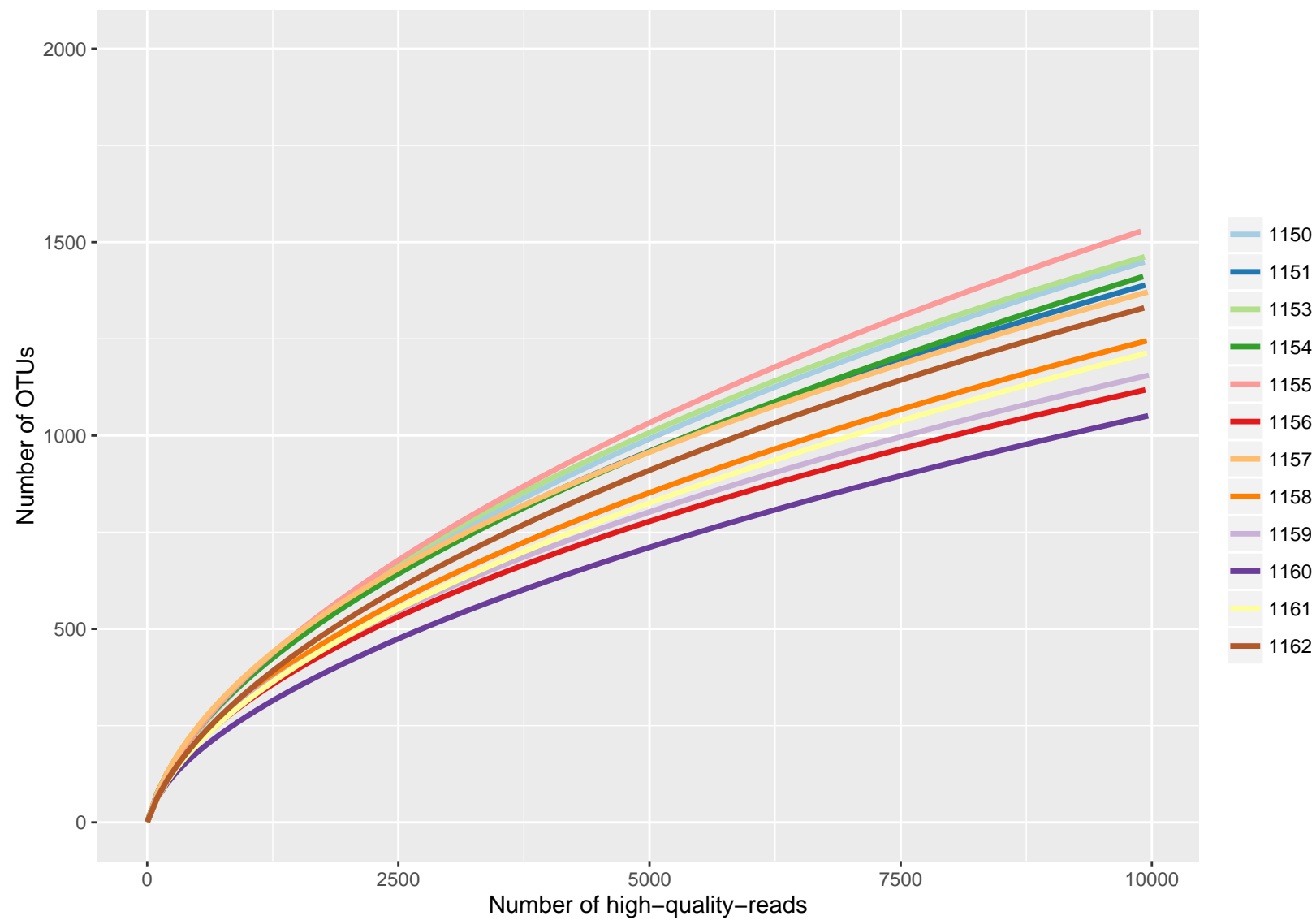

Supplement: S2 File — (ZIP) [file pone.0186766.s008.zip › Rarefact_curves_75.pdf]

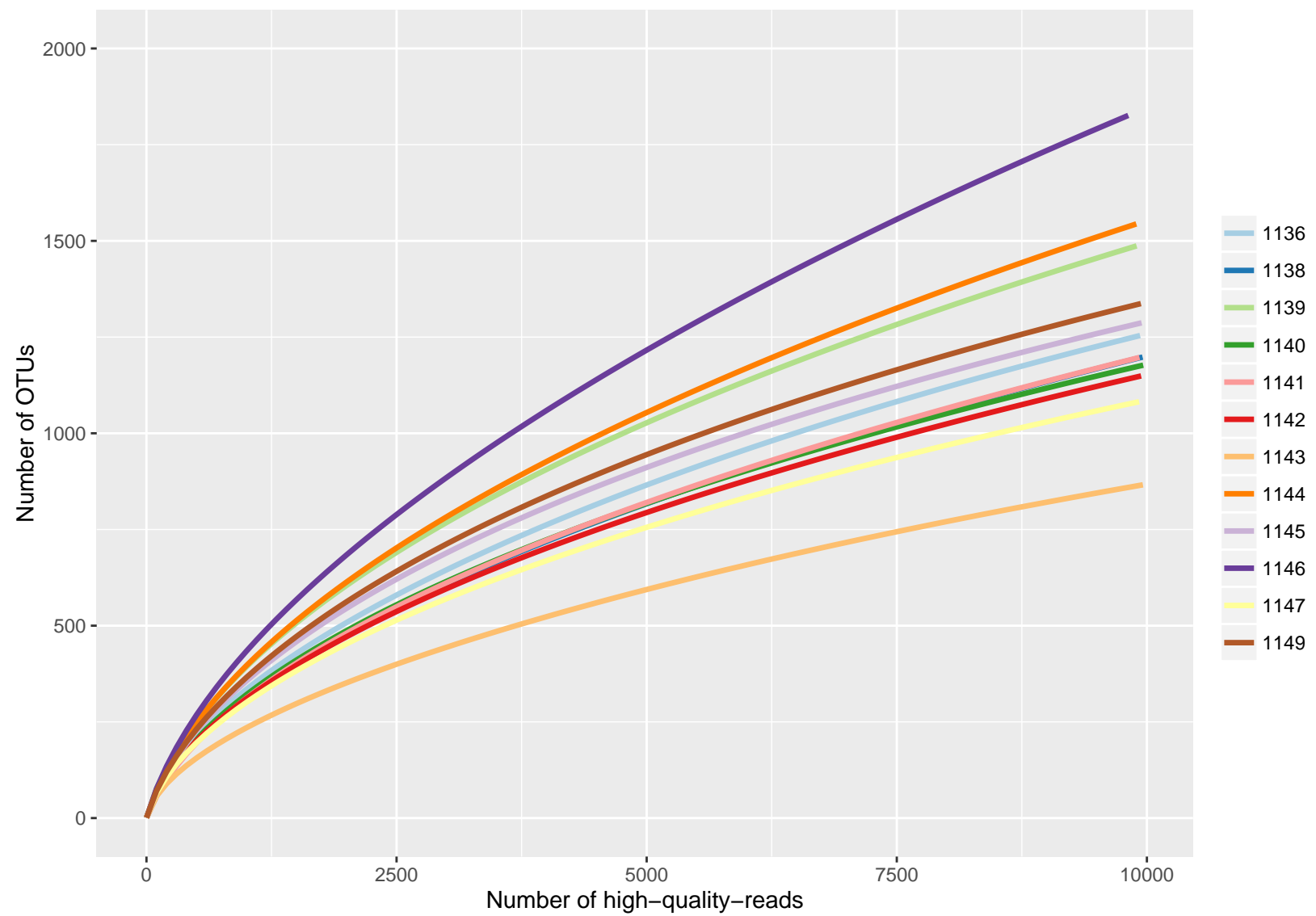

Supplement: S2 File — (ZIP) [file pone.0186766.s008.zip › Rarefact_curves_74.pdf]

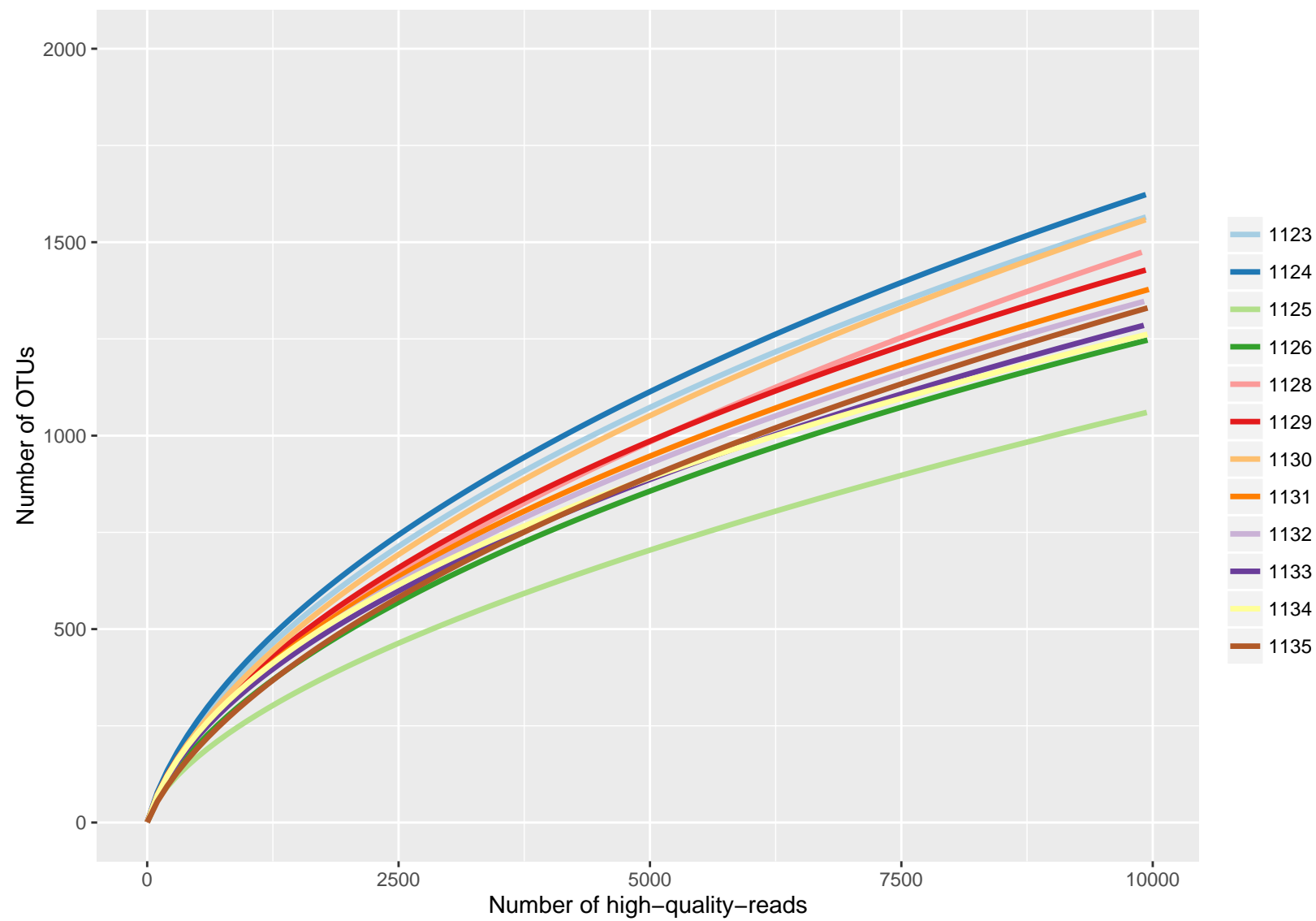

Supplement: S2 File — (ZIP) [file pone.0186766.s008.zip › Rarefact_curves_73.pdf]

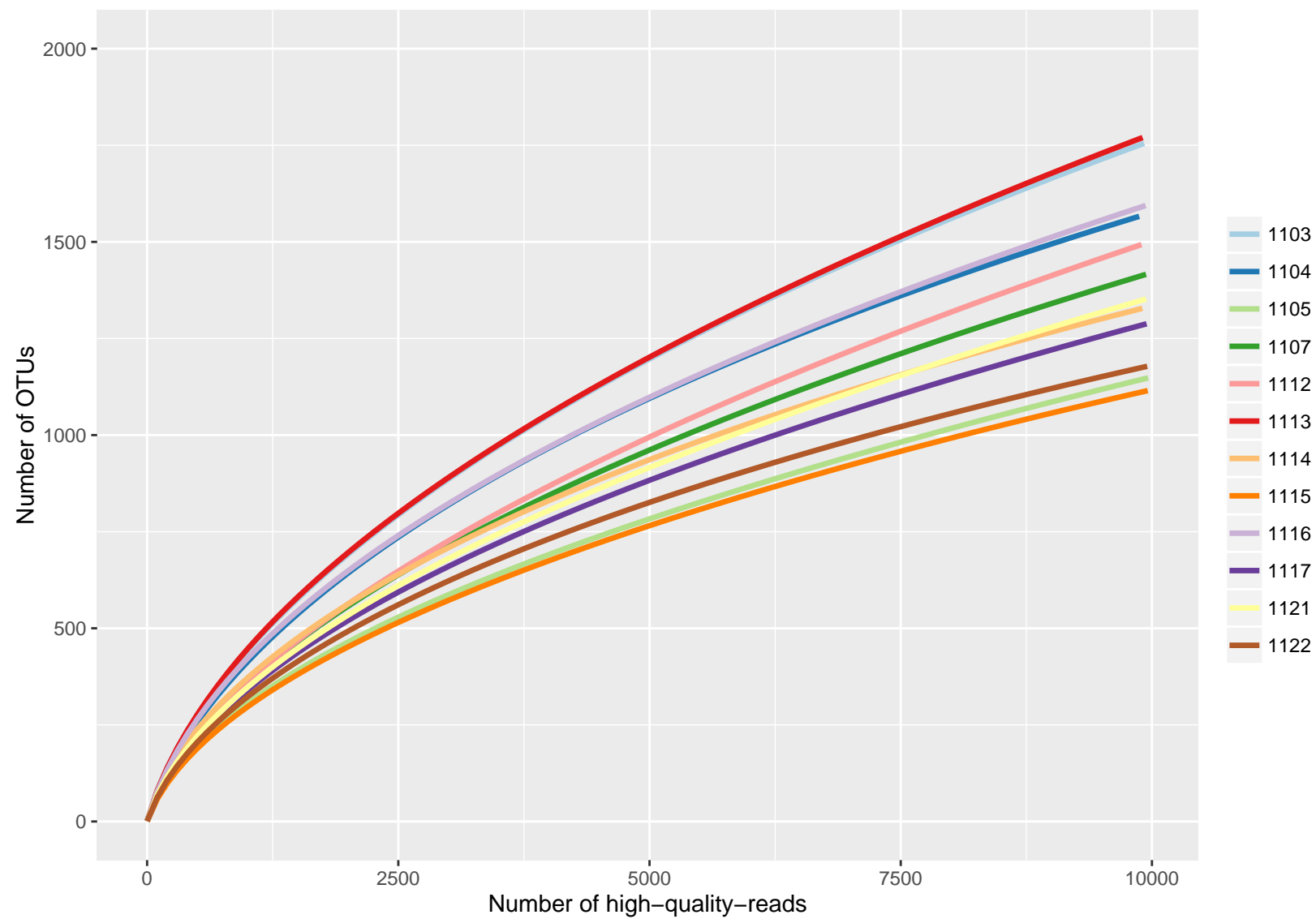

Supplement: S2 File — (ZIP) [file pone.0186766.s008.zip › Rarefact_curves_72.pdf]

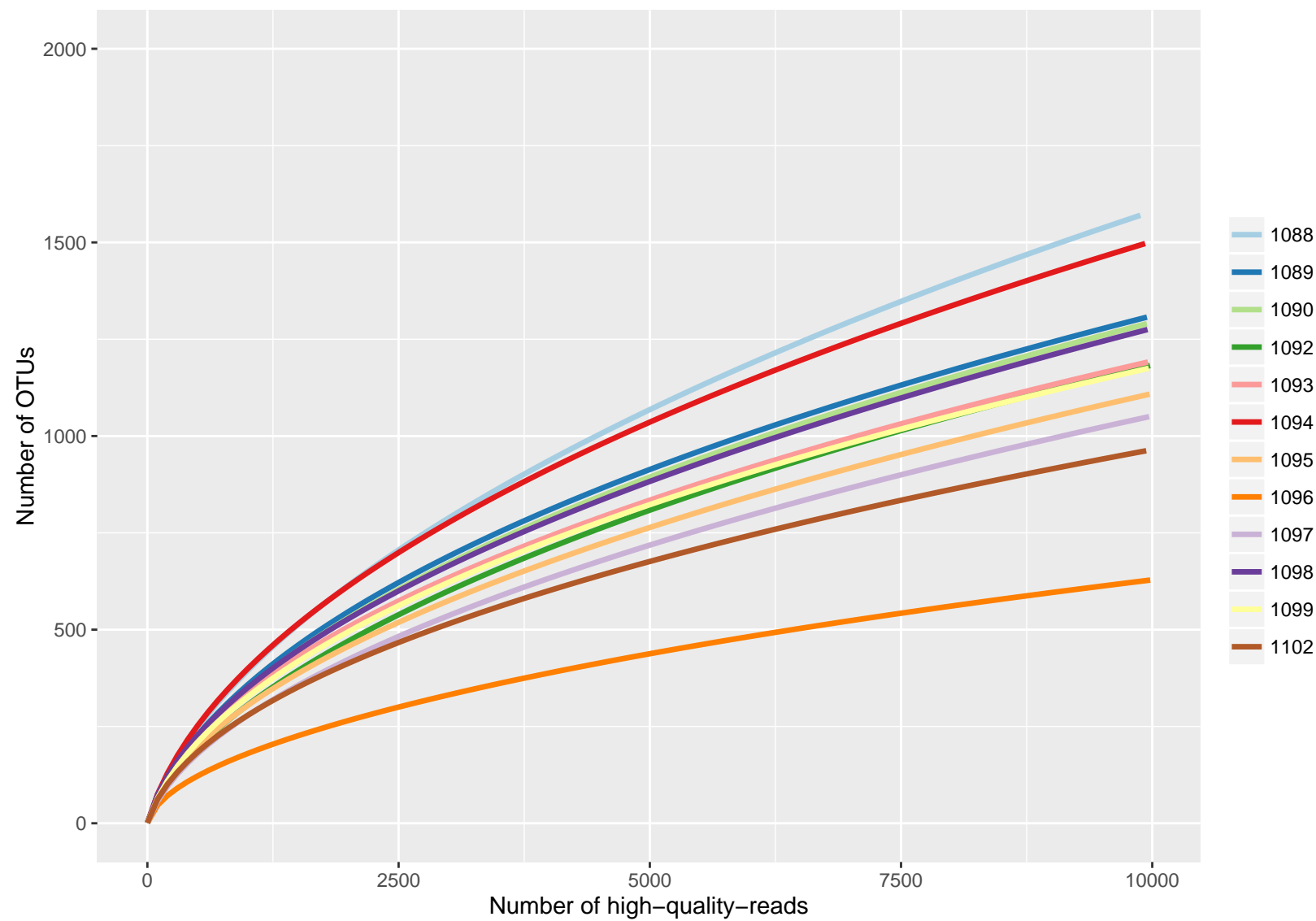

Supplement: S2 File — (ZIP) [file pone.0186766.s008.zip › Rarefact_curves_71.pdf]

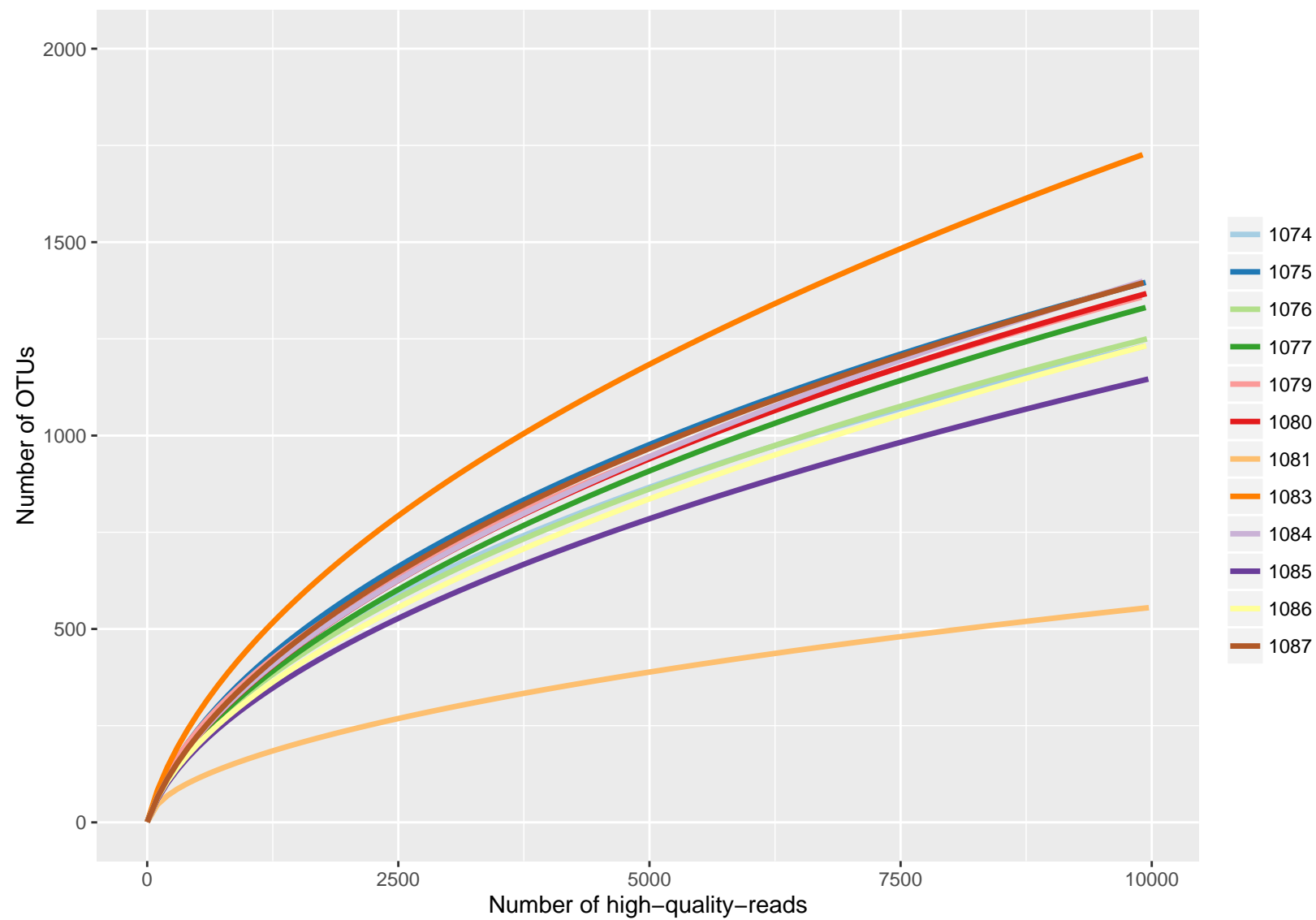

Supplement: S2 File — (ZIP) [file pone.0186766.s008.zip › Rarefact_curves_70.pdf]

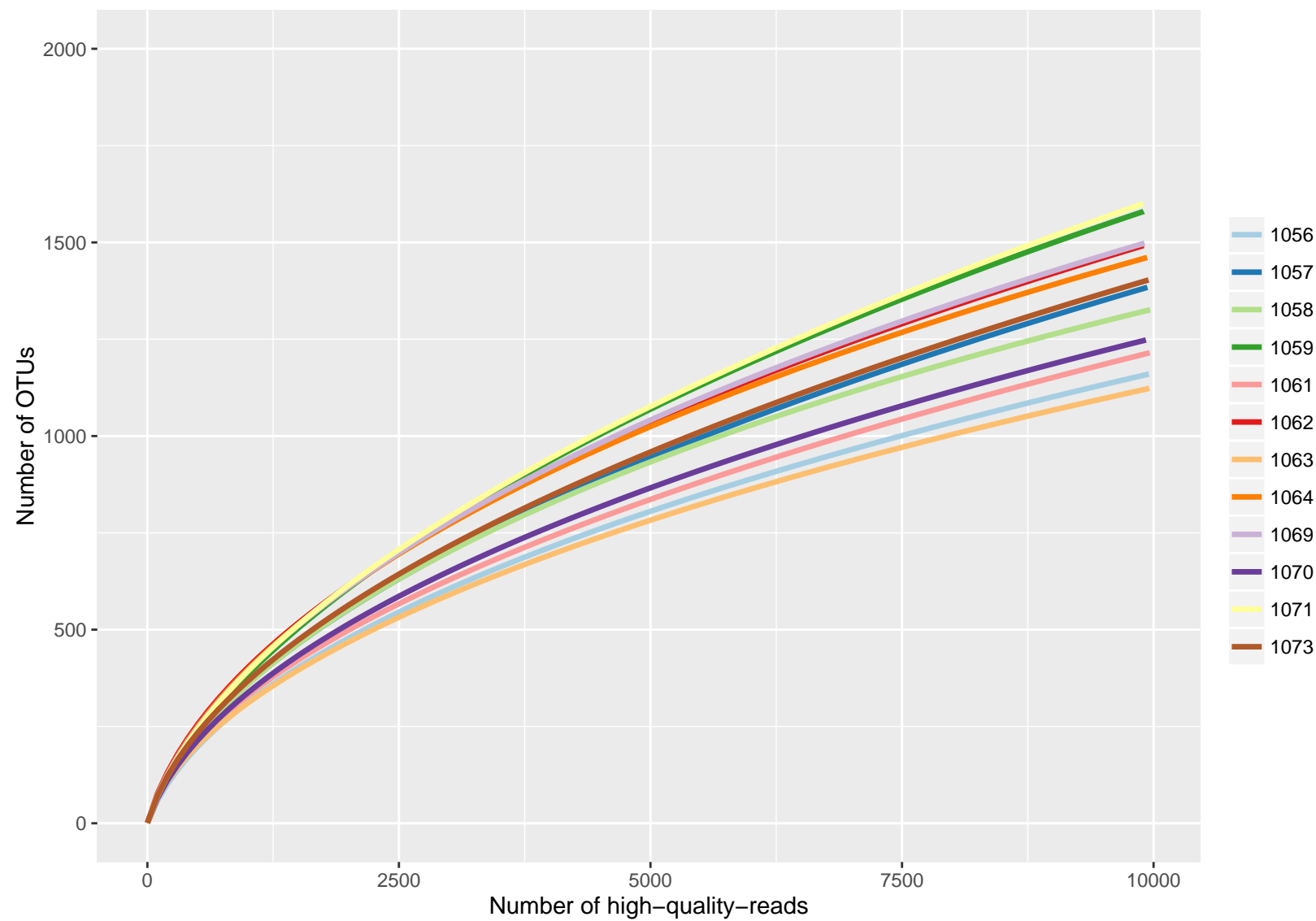

Supplement: S2 File — (ZIP) [file pone.0186766.s008.zip › Rarefact_curves_69.pdf]

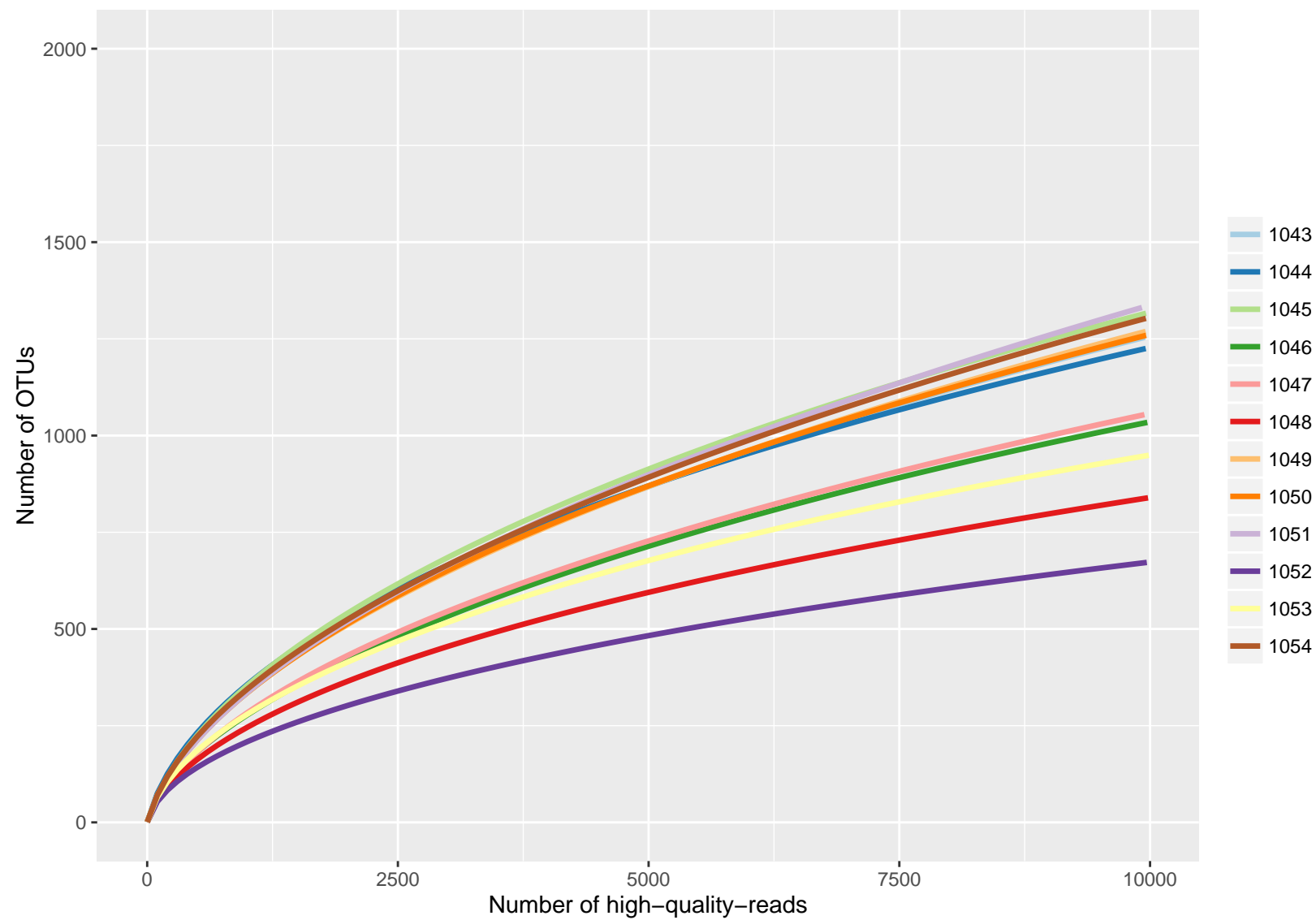

Supplement: S2 File — (ZIP) [file pone.0186766.s008.zip › Rarefact_curves_68.pdf]

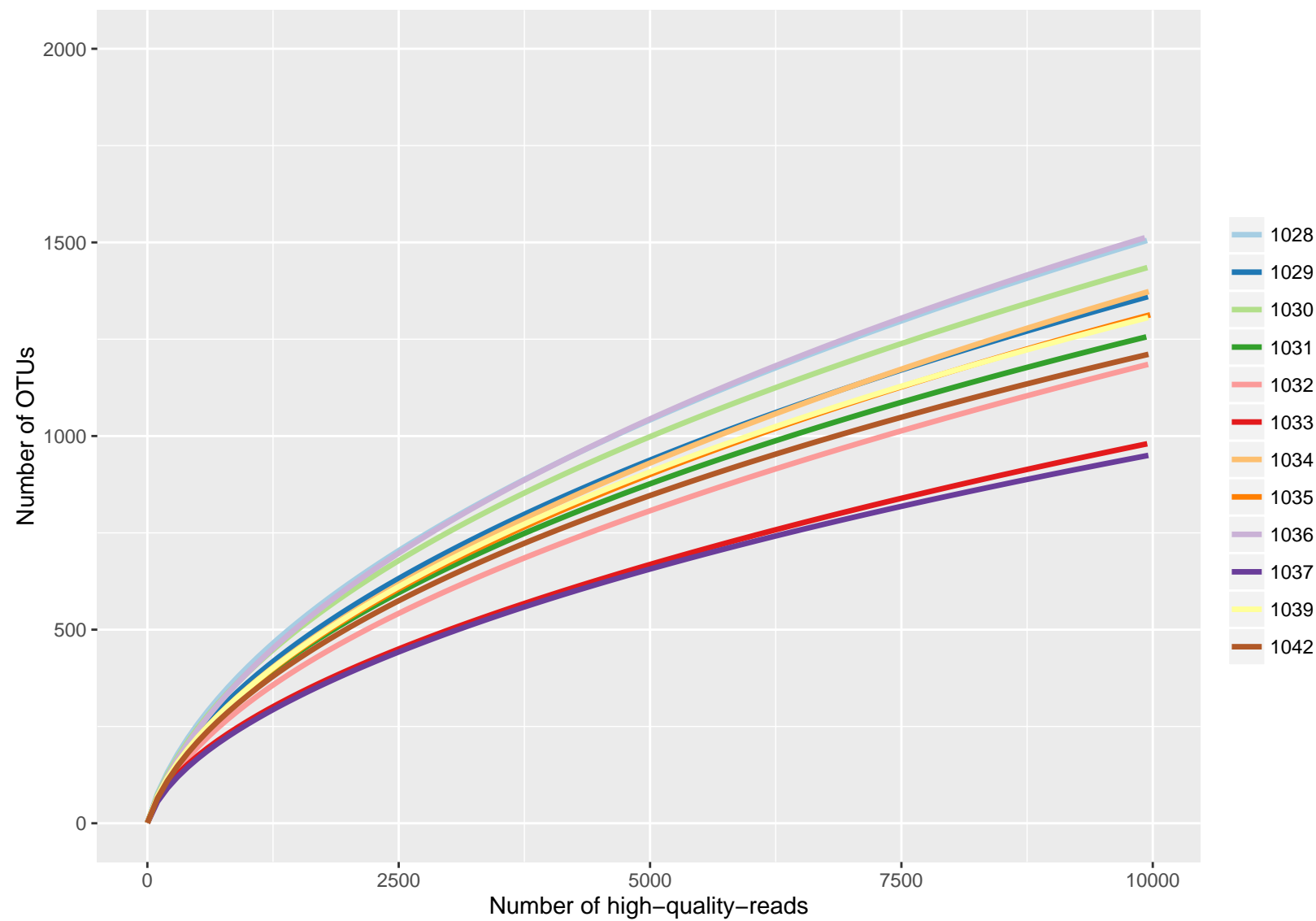

Supplement: S2 File — (ZIP) [file pone.0186766.s008.zip › Rarefact_curves_67.pdf]

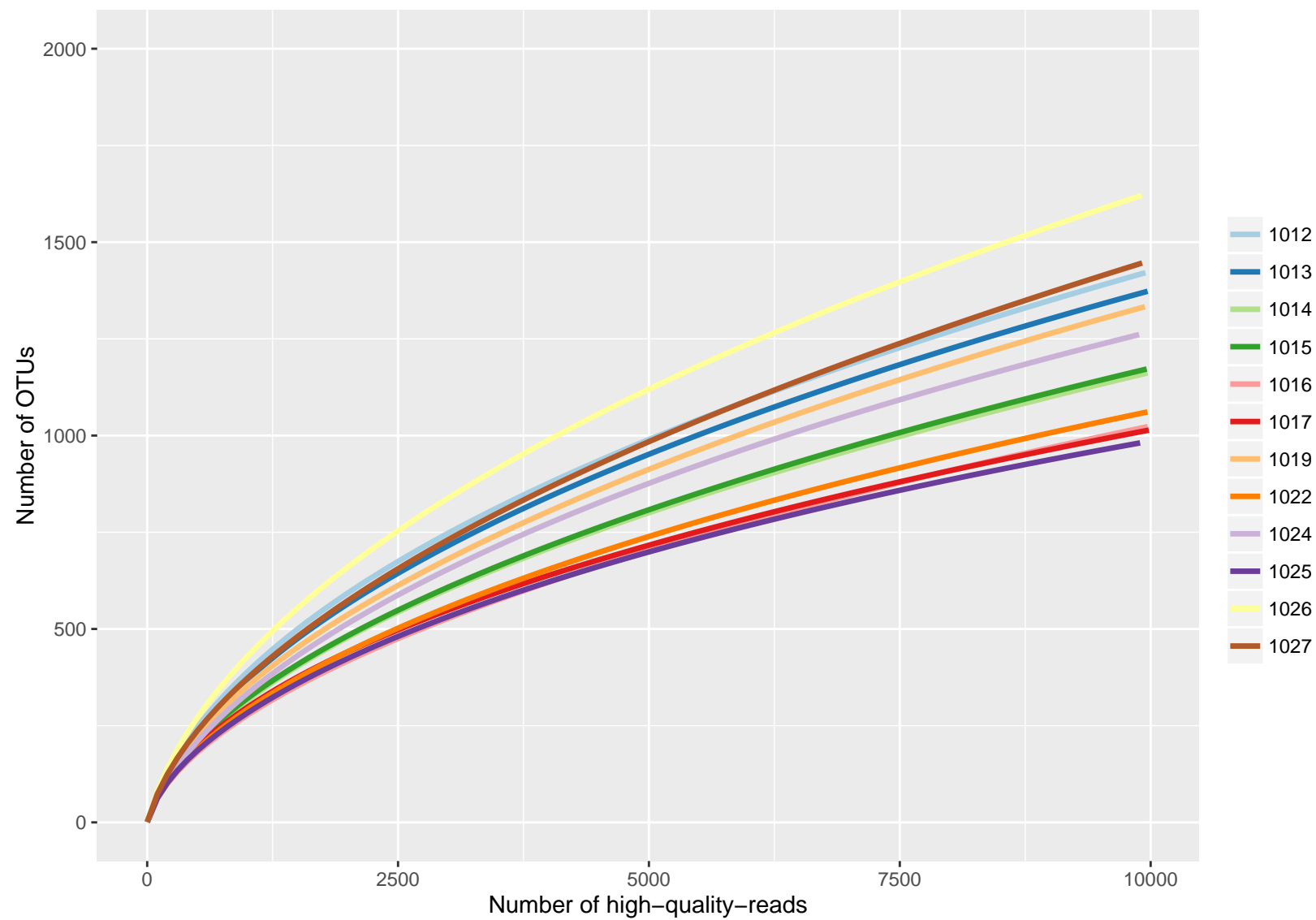

Supplement: S2 File — (ZIP) [file pone.0186766.s008.zip › Rarefact_curves_66.pdf]

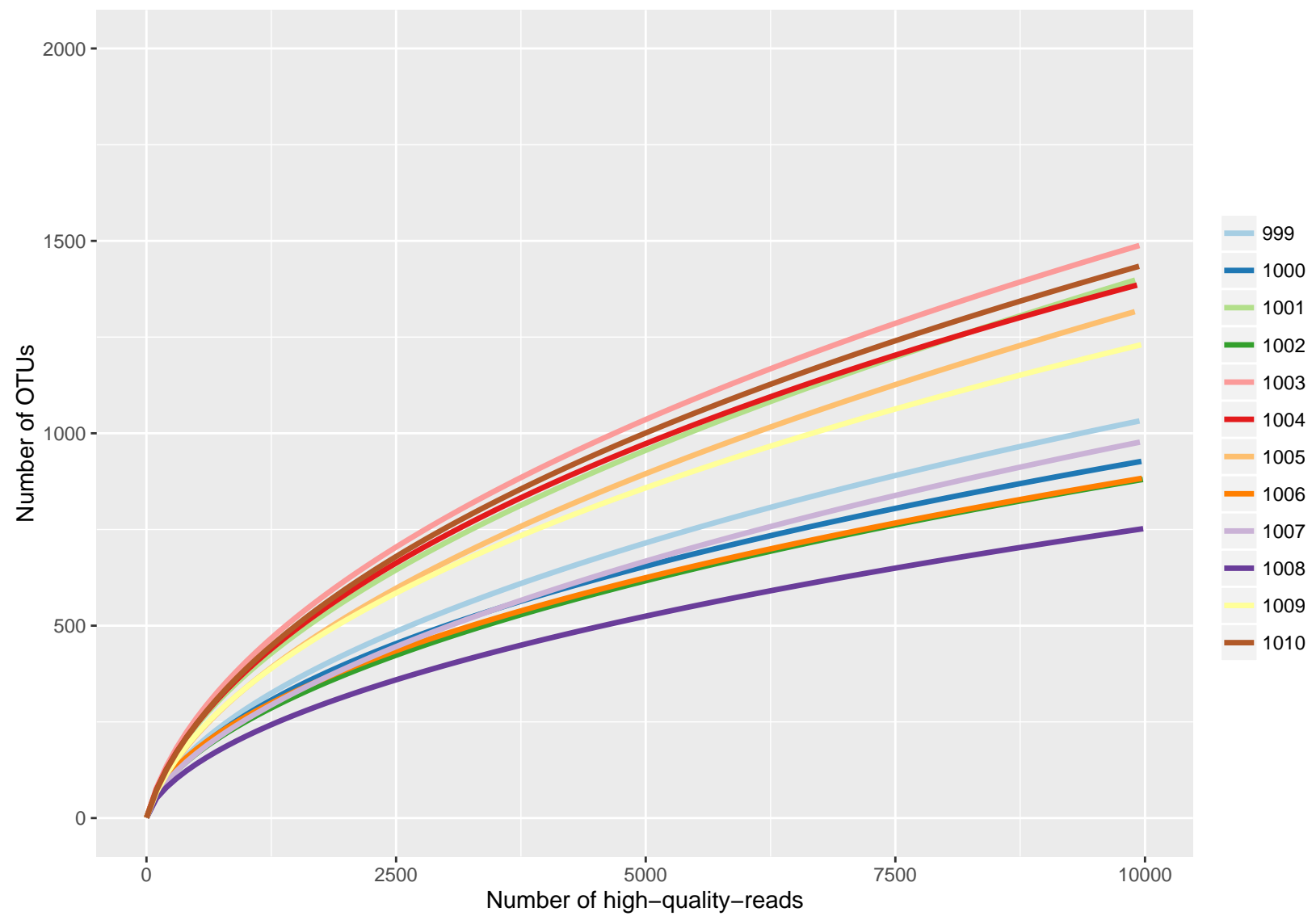

Supplement: S2 File — (ZIP) [file pone.0186766.s008.zip › Rarefact_curves_65.pdf]

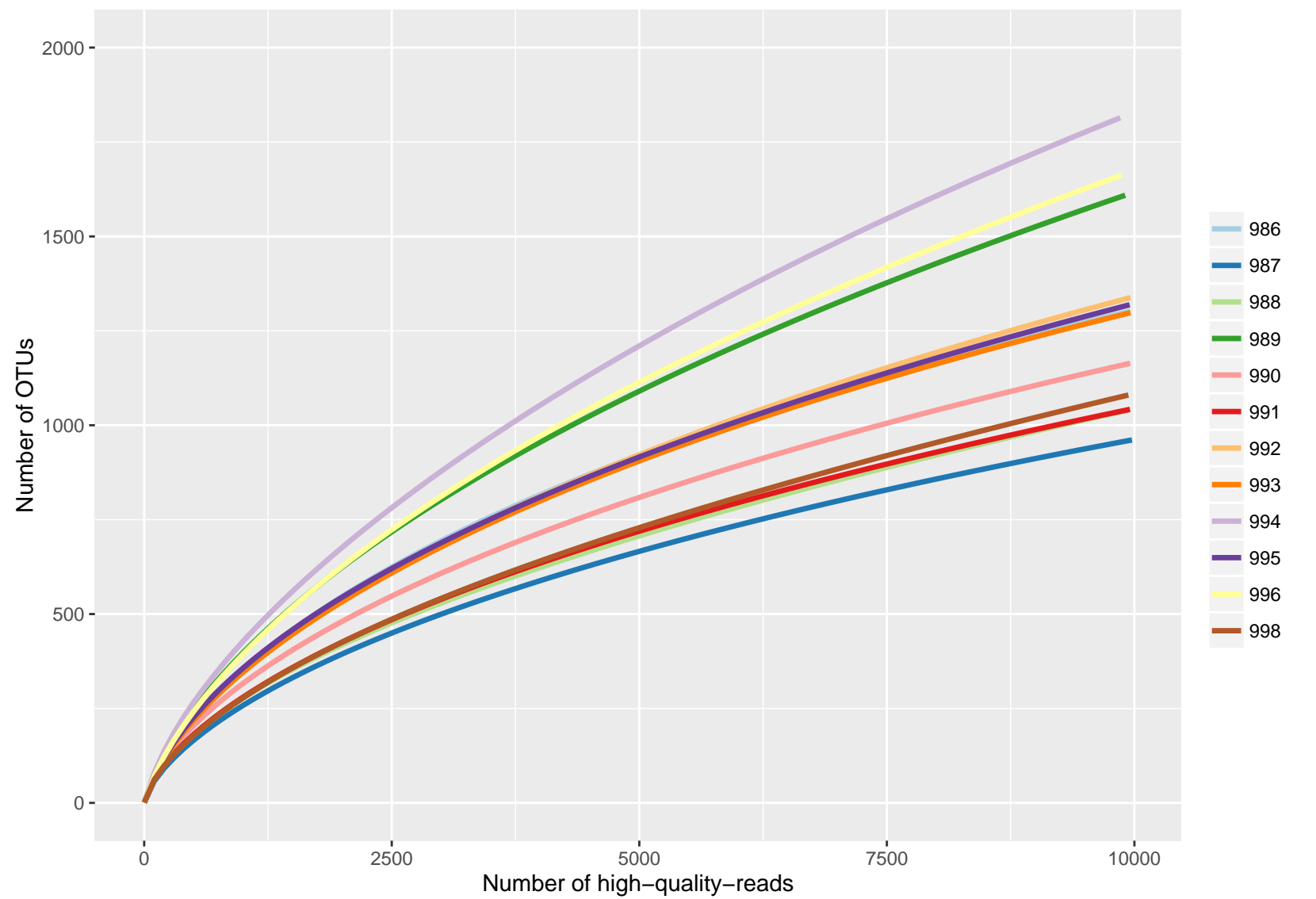

Supplement: S2 File — (ZIP) [file pone.0186766.s008.zip › Rarefact_curves_64.pdf]

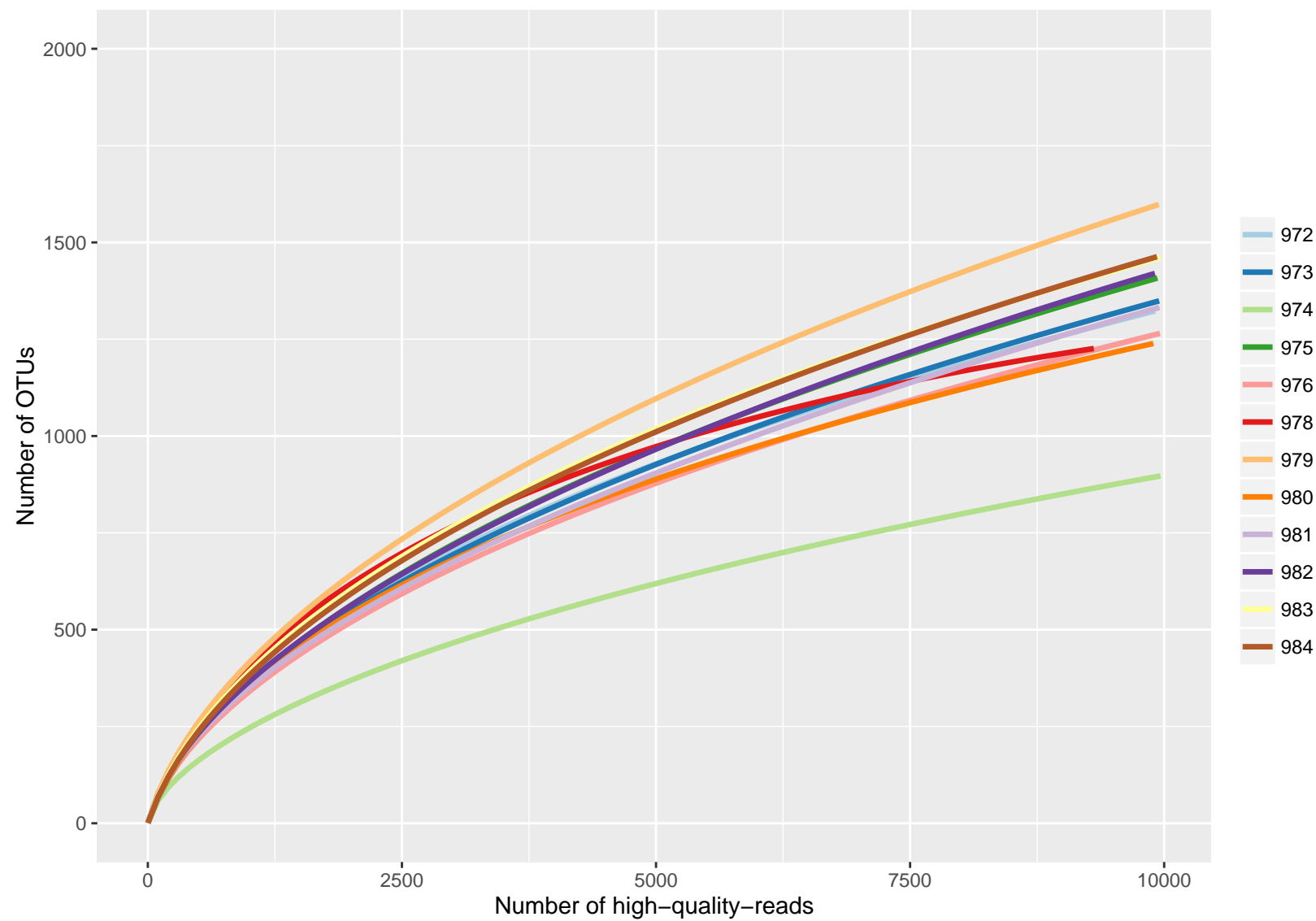

Supplement: S2 File — (ZIP) [file pone.0186766.s008.zip › Rarefact_curves_63.pdf]

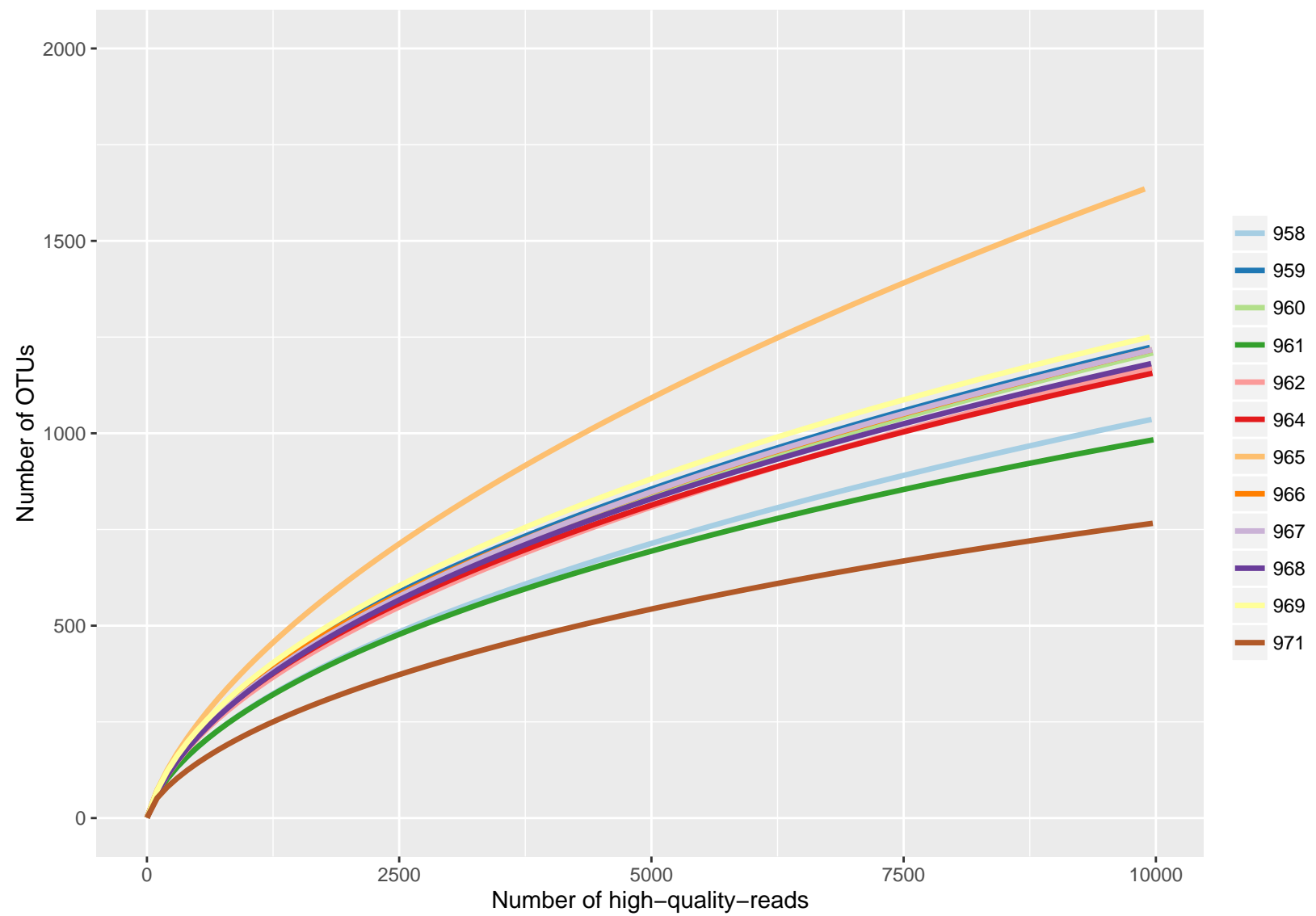

Supplement: S2 File — (ZIP) [file pone.0186766.s008.zip › Rarefact_curves_62.pdf]

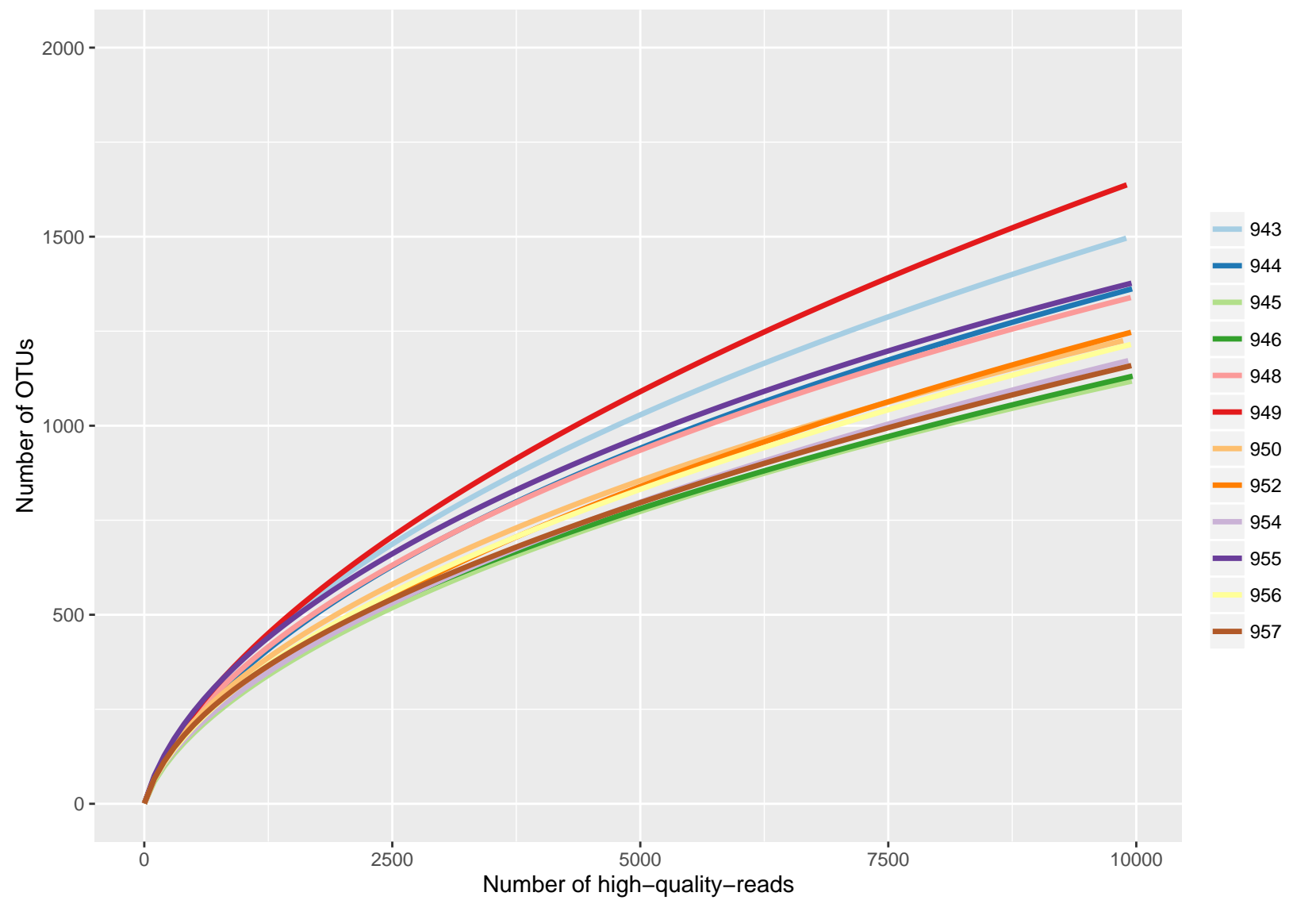

Supplement: S2 File — (ZIP) [file pone.0186766.s008.zip › Rarefact_curves_61.pdf]

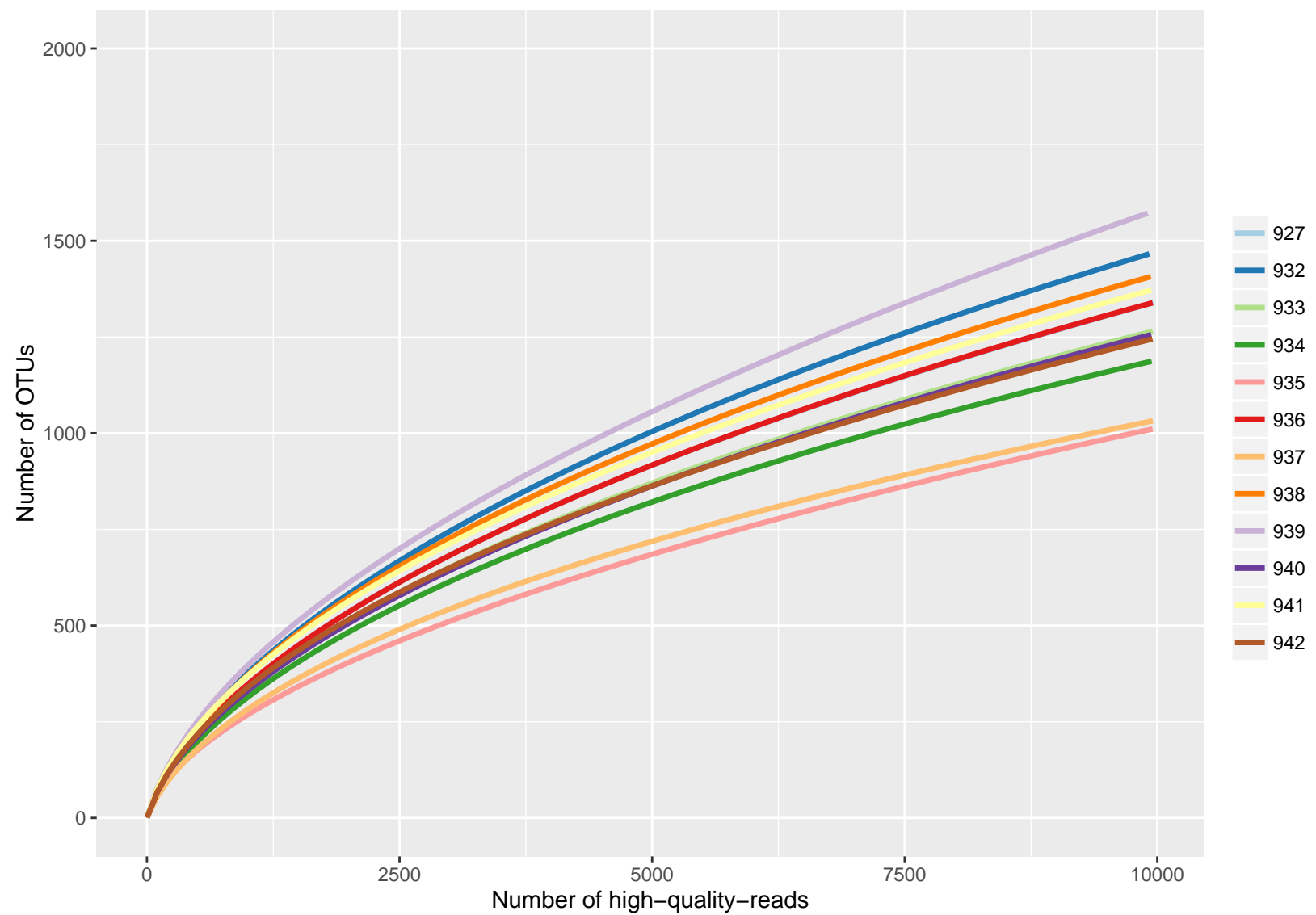

Supplement: S2 File — (ZIP) [file pone.0186766.s008.zip › Rarefact_curves_60.pdf]

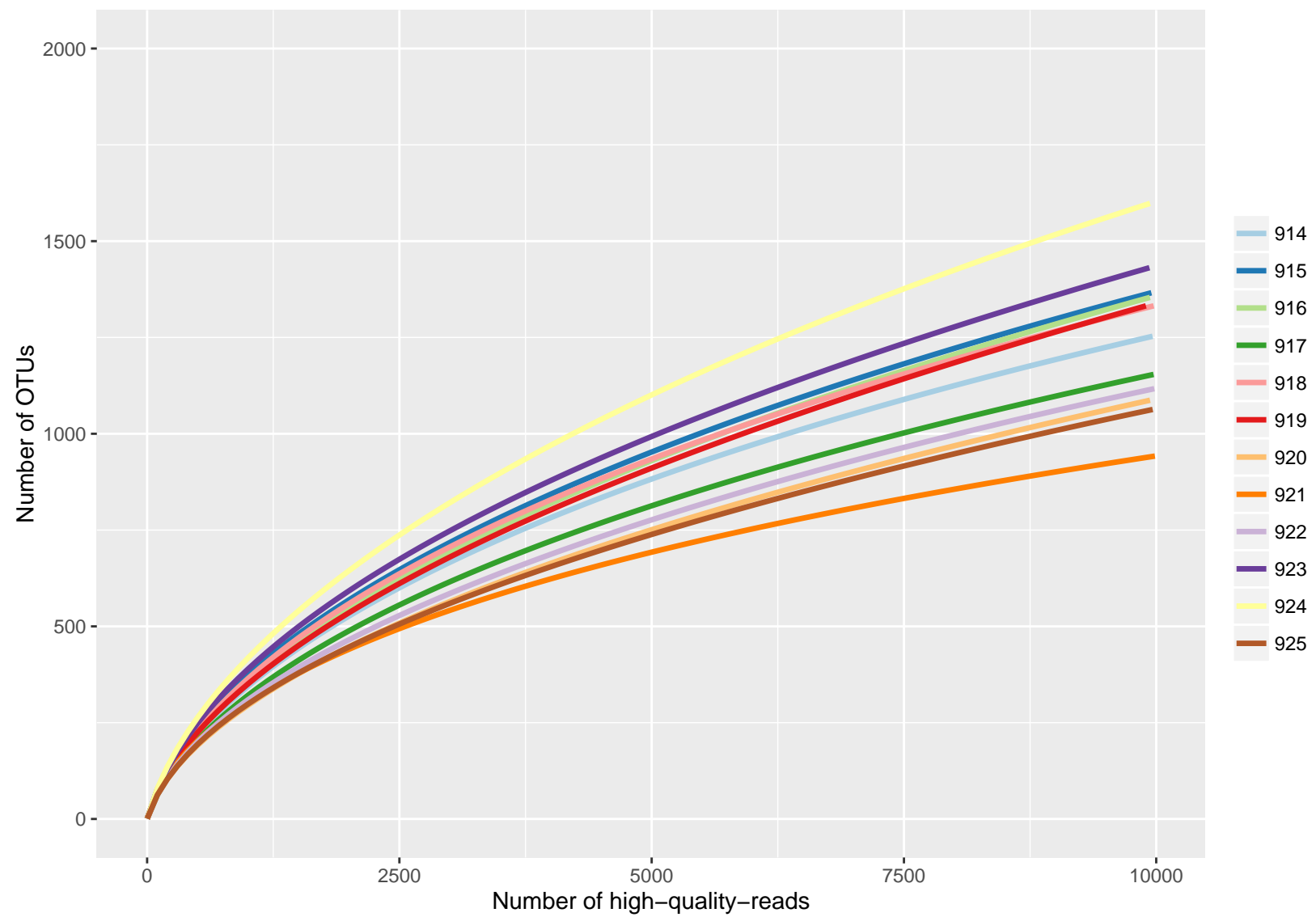

Supplement: S2 File — (ZIP) [file pone.0186766.s008.zip › Rarefact_curves_59.pdf]

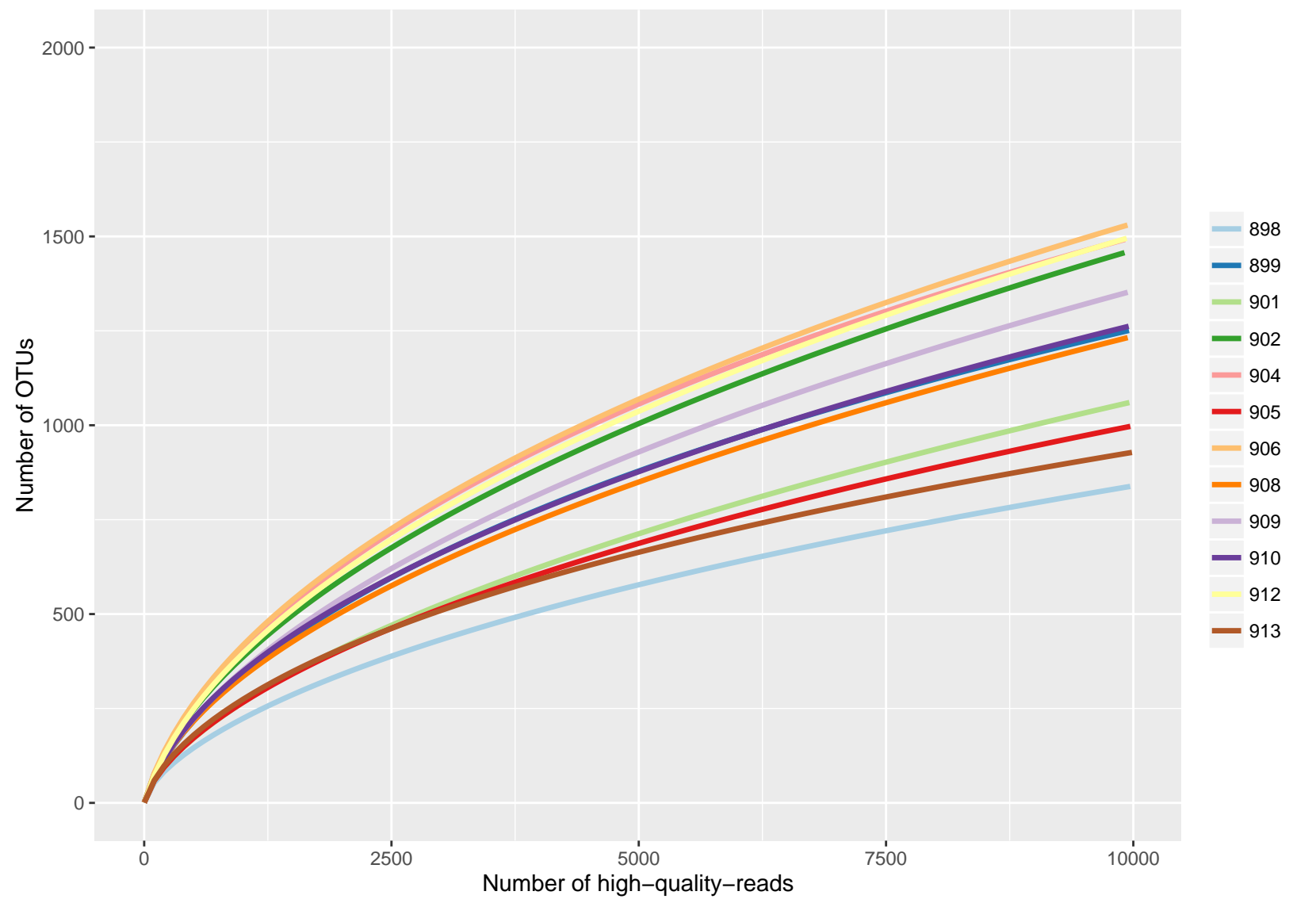

Supplement: S2 File — (ZIP) [file pone.0186766.s008.zip › Rarefact_curves_58.pdf]

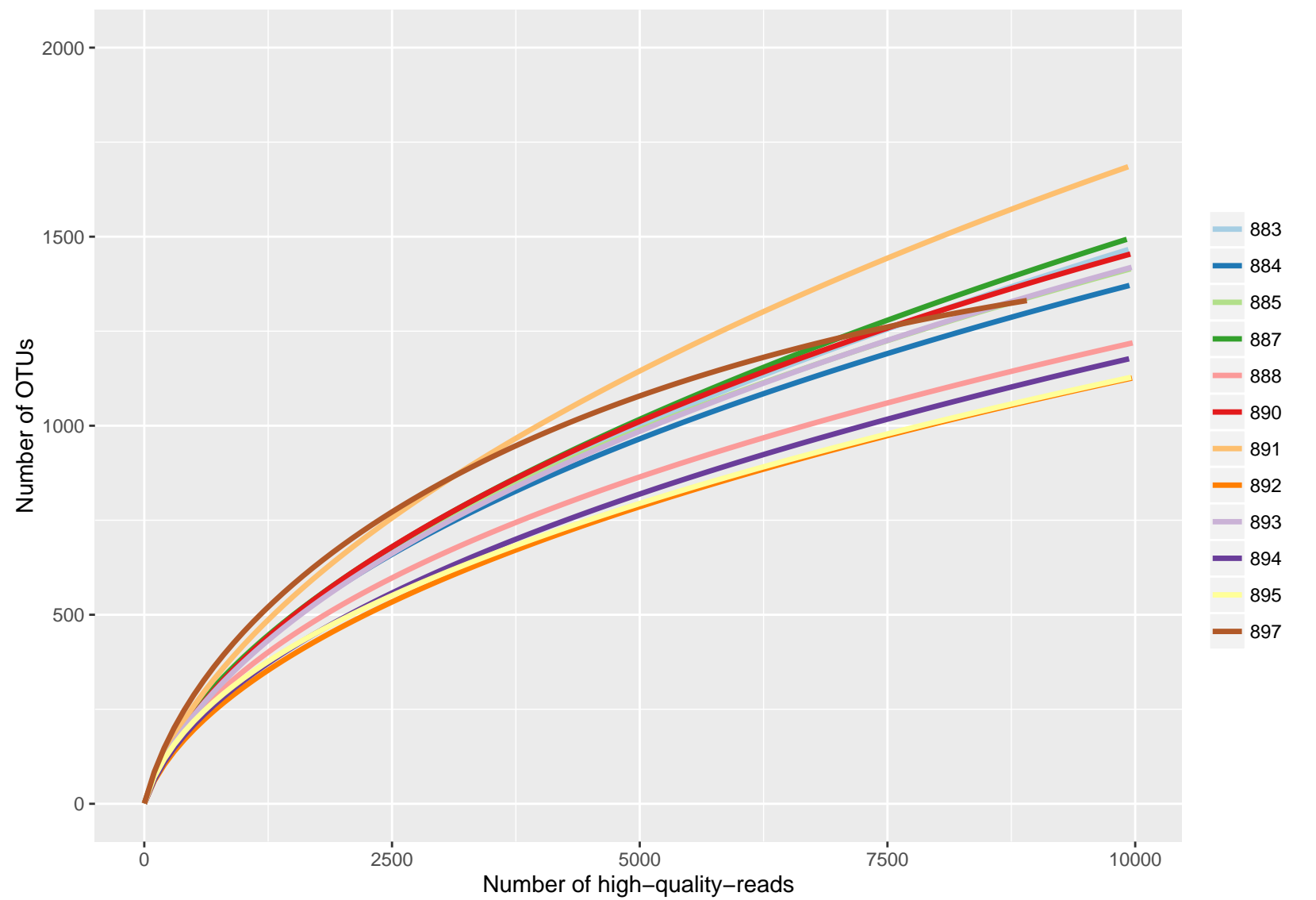

Supplement: S2 File — (ZIP) [file pone.0186766.s008.zip › Rarefact_curves_57.pdf]

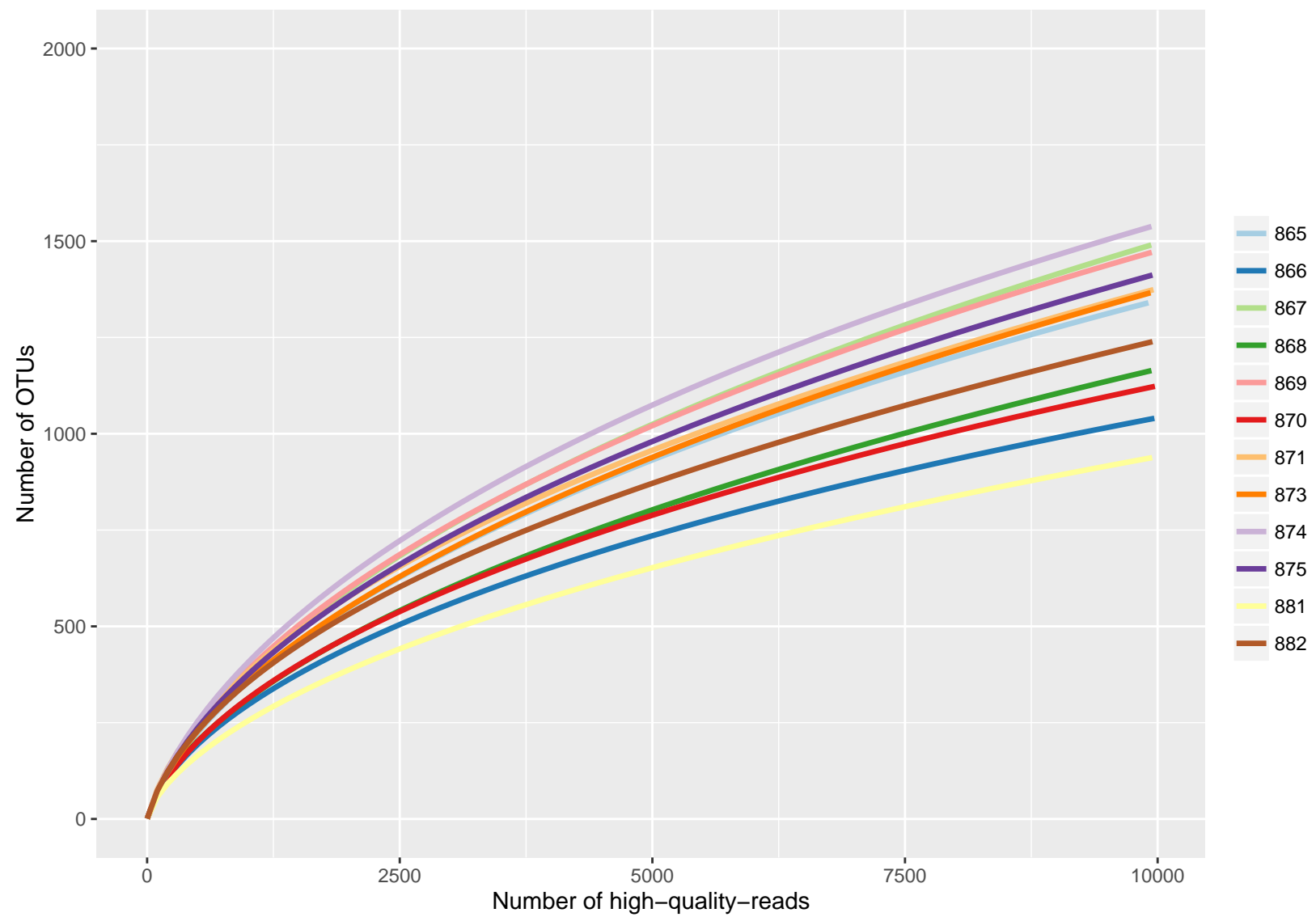

Supplement: S2 File — (ZIP) [file pone.0186766.s008.zip › Rarefact_curves_56.pdf]

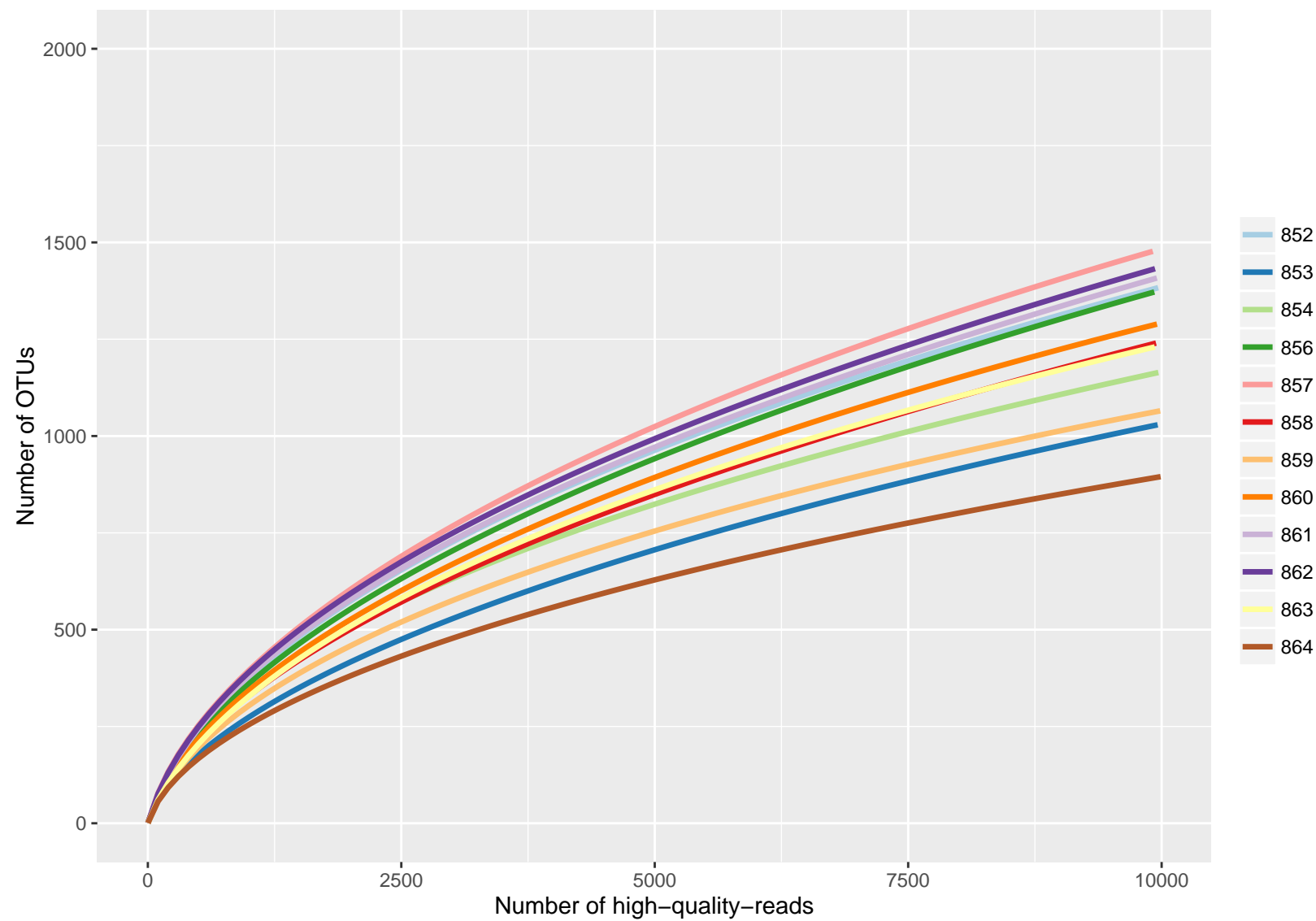

Supplement: S2 File — (ZIP) [file pone.0186766.s008.zip › Rarefact_curves_55.pdf]

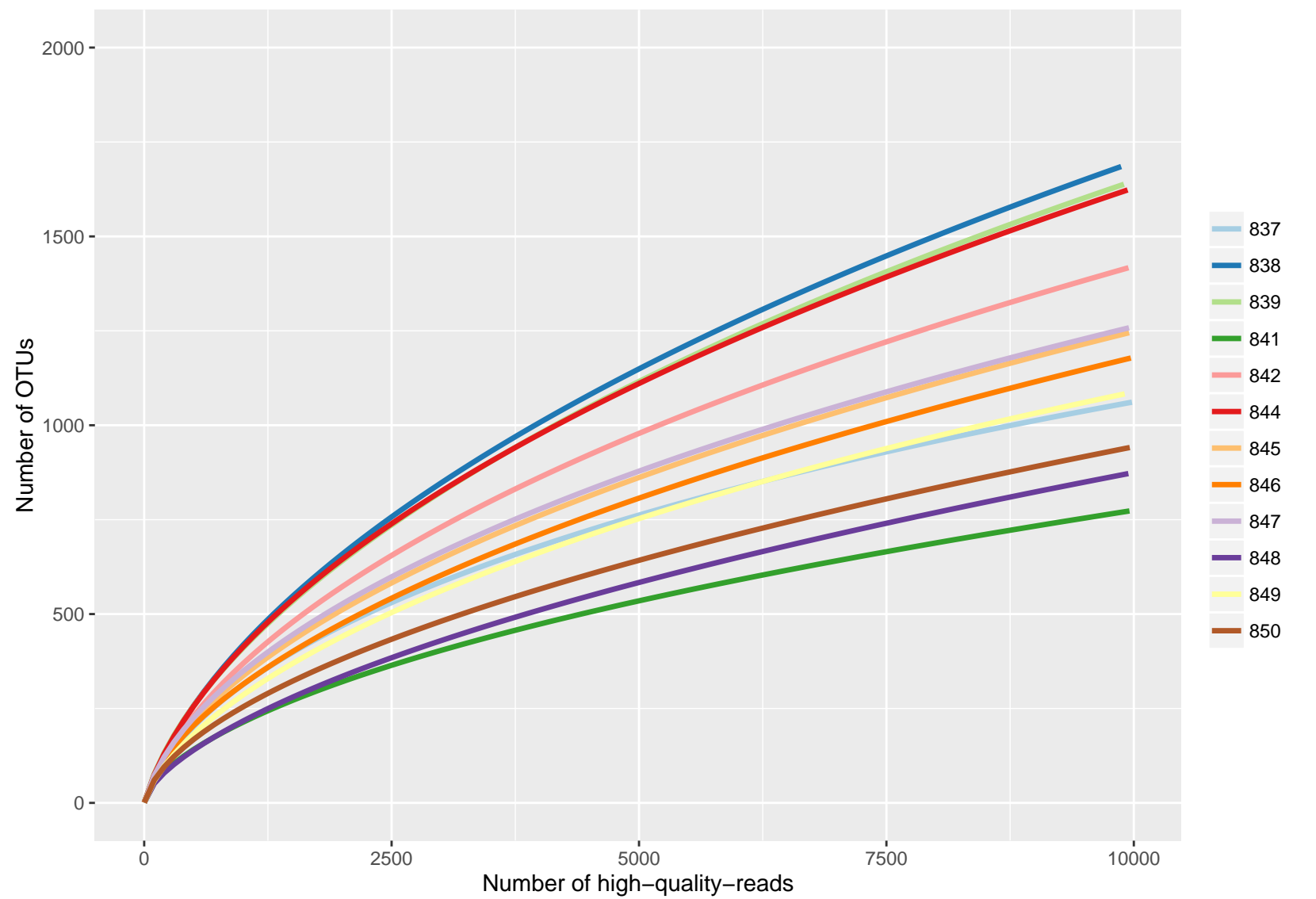

Supplement: S2 File — (ZIP) [file pone.0186766.s008.zip › Rarefact_curves_54.pdf]

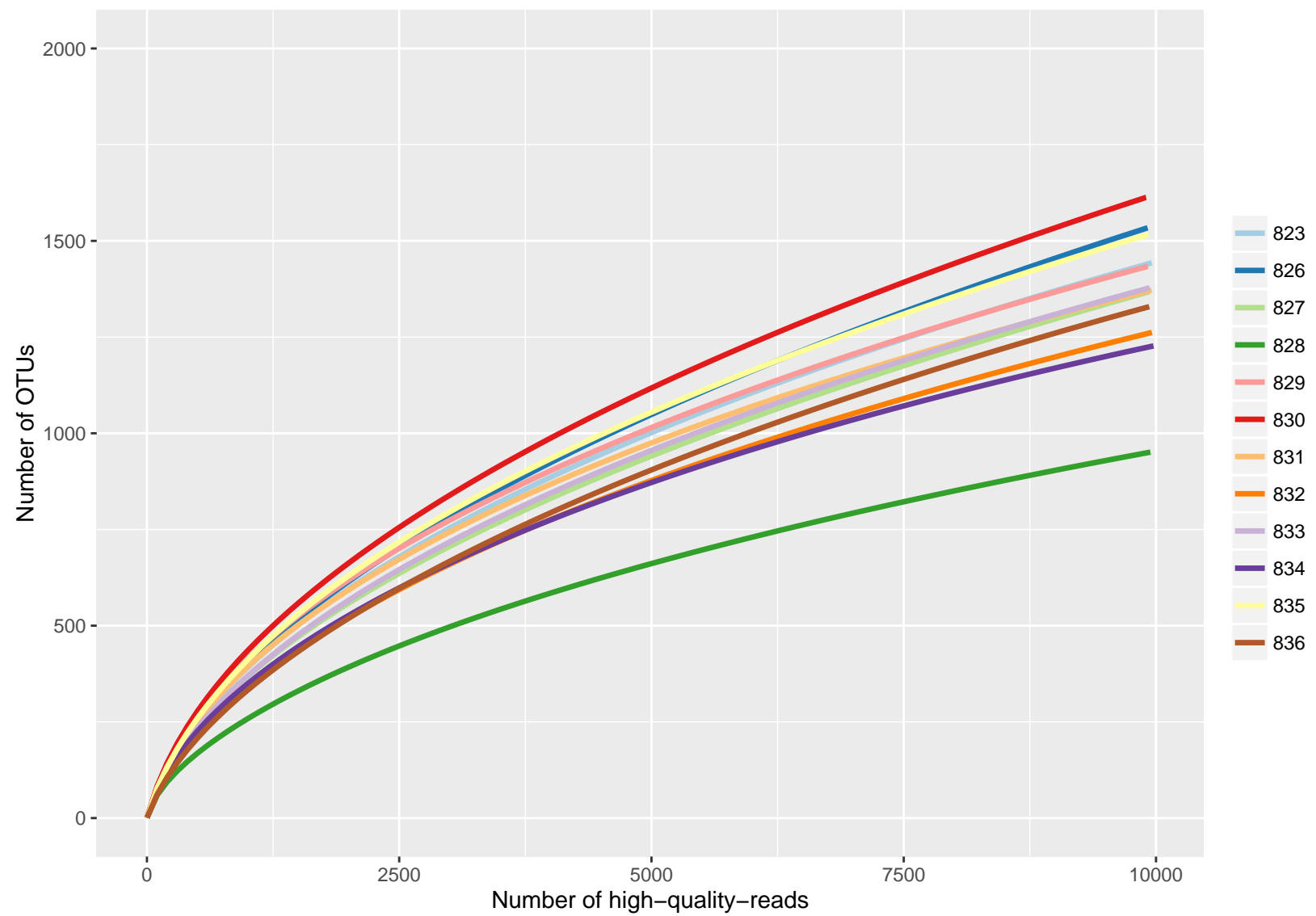

Supplement: S2 File — (ZIP) [file pone.0186766.s008.zip › Rarefact_curves_53.pdf]

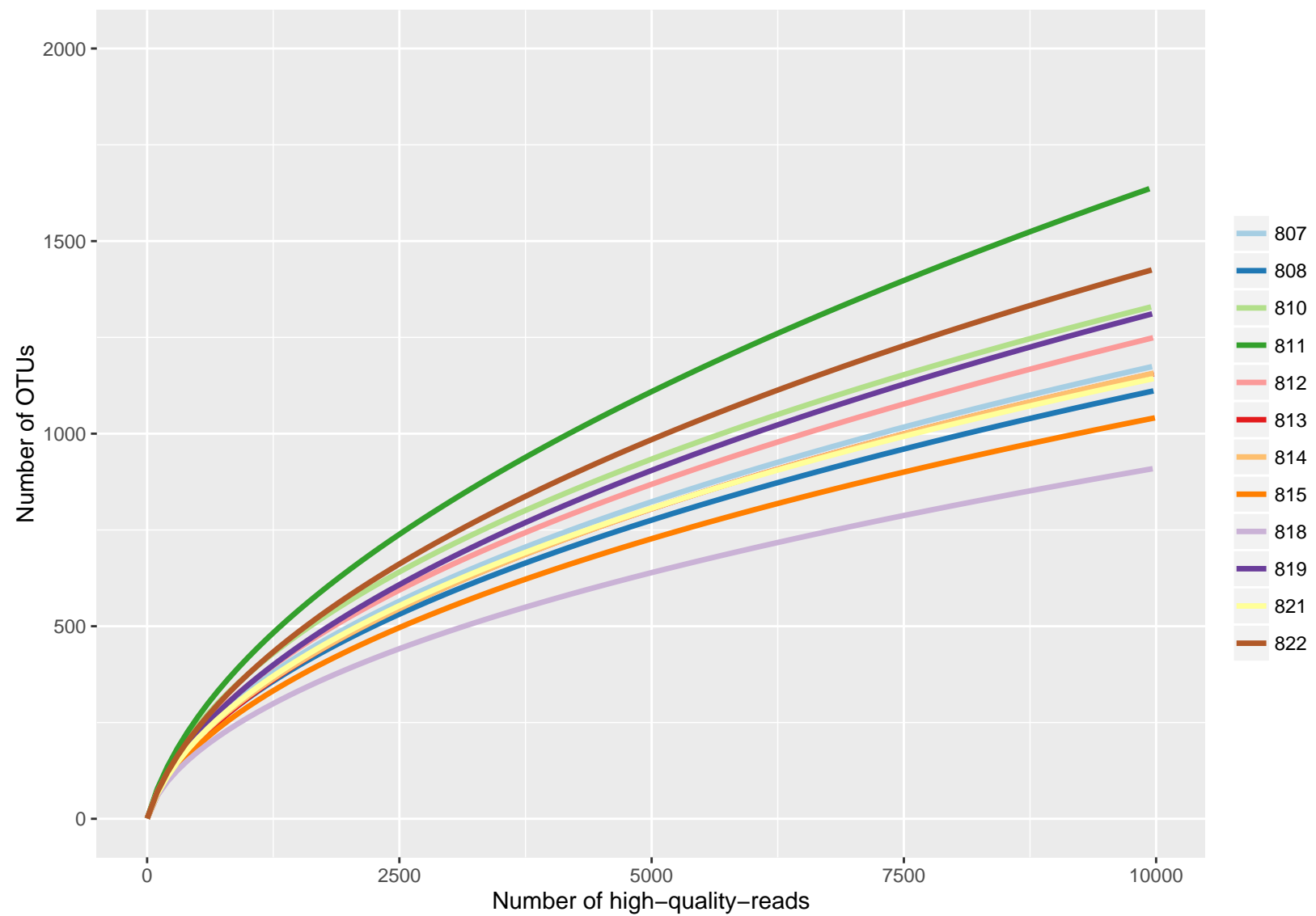

Supplement: S2 File — (ZIP) [file pone.0186766.s008.zip › Rarefact_curves_52.pdf]

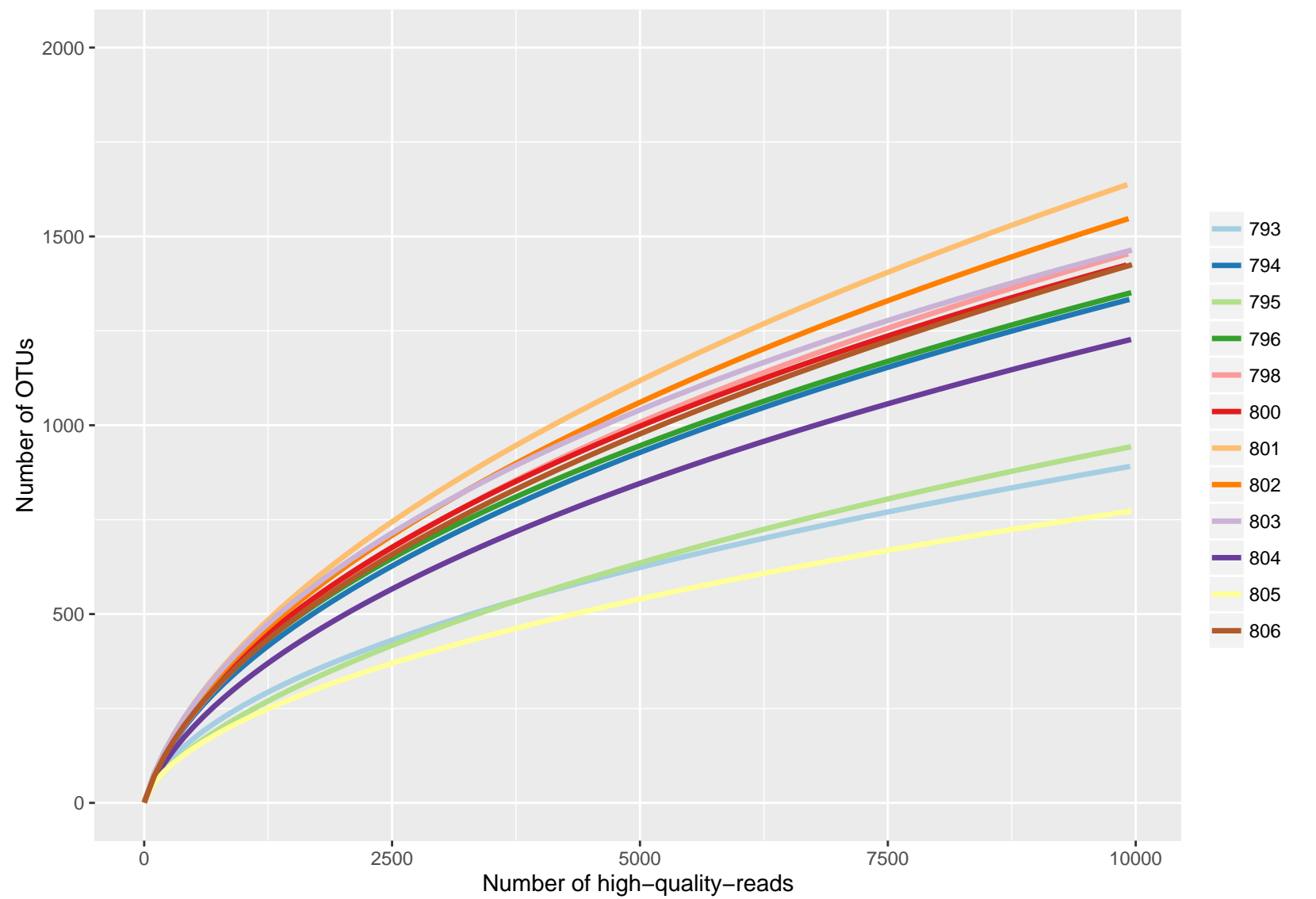

Supplement: S2 File — (ZIP) [file pone.0186766.s008.zip › Rarefact_curves_51.pdf]

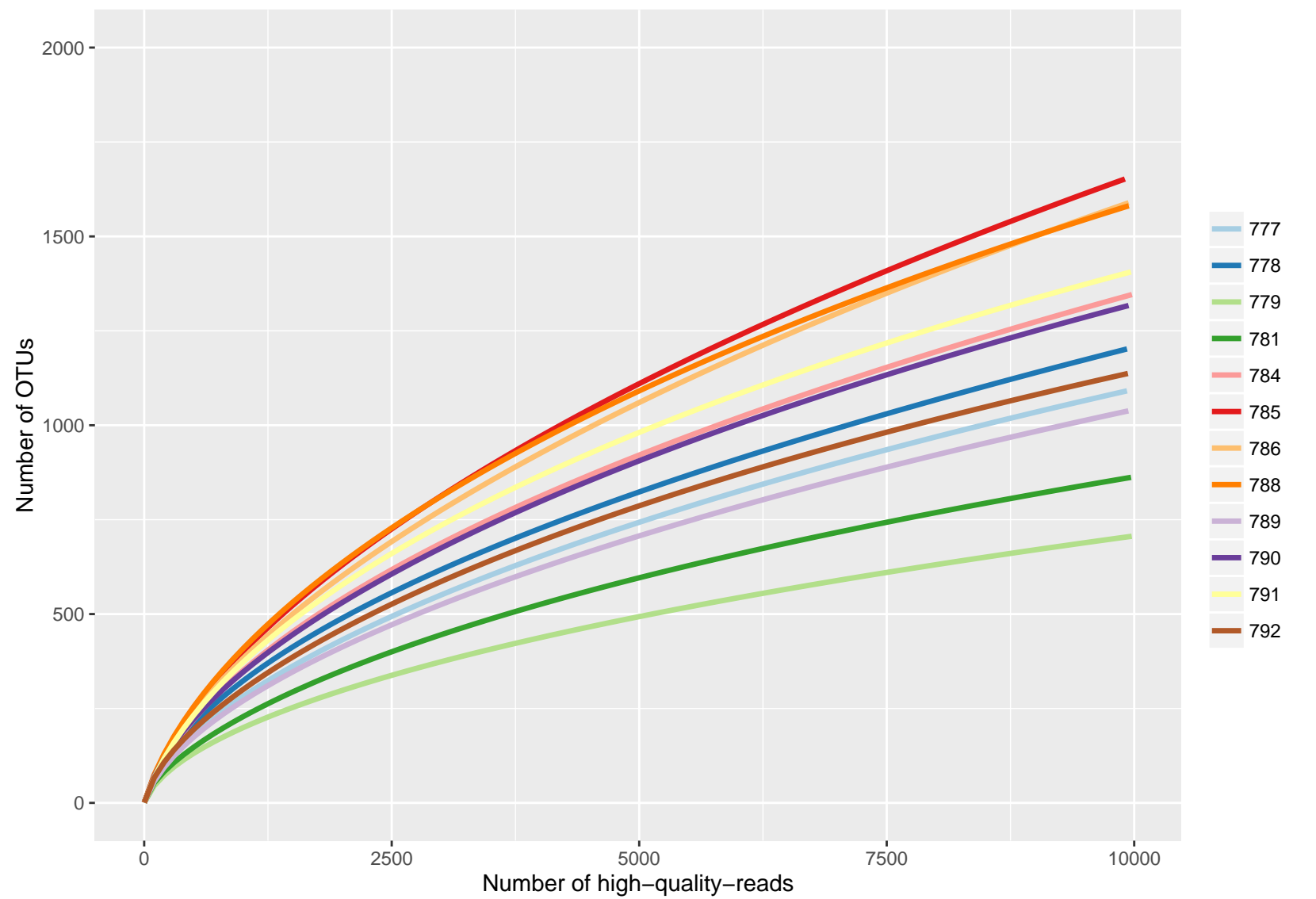

Supplement: S2 File — (ZIP) [file pone.0186766.s008.zip › Rarefact_curves_50.pdf]

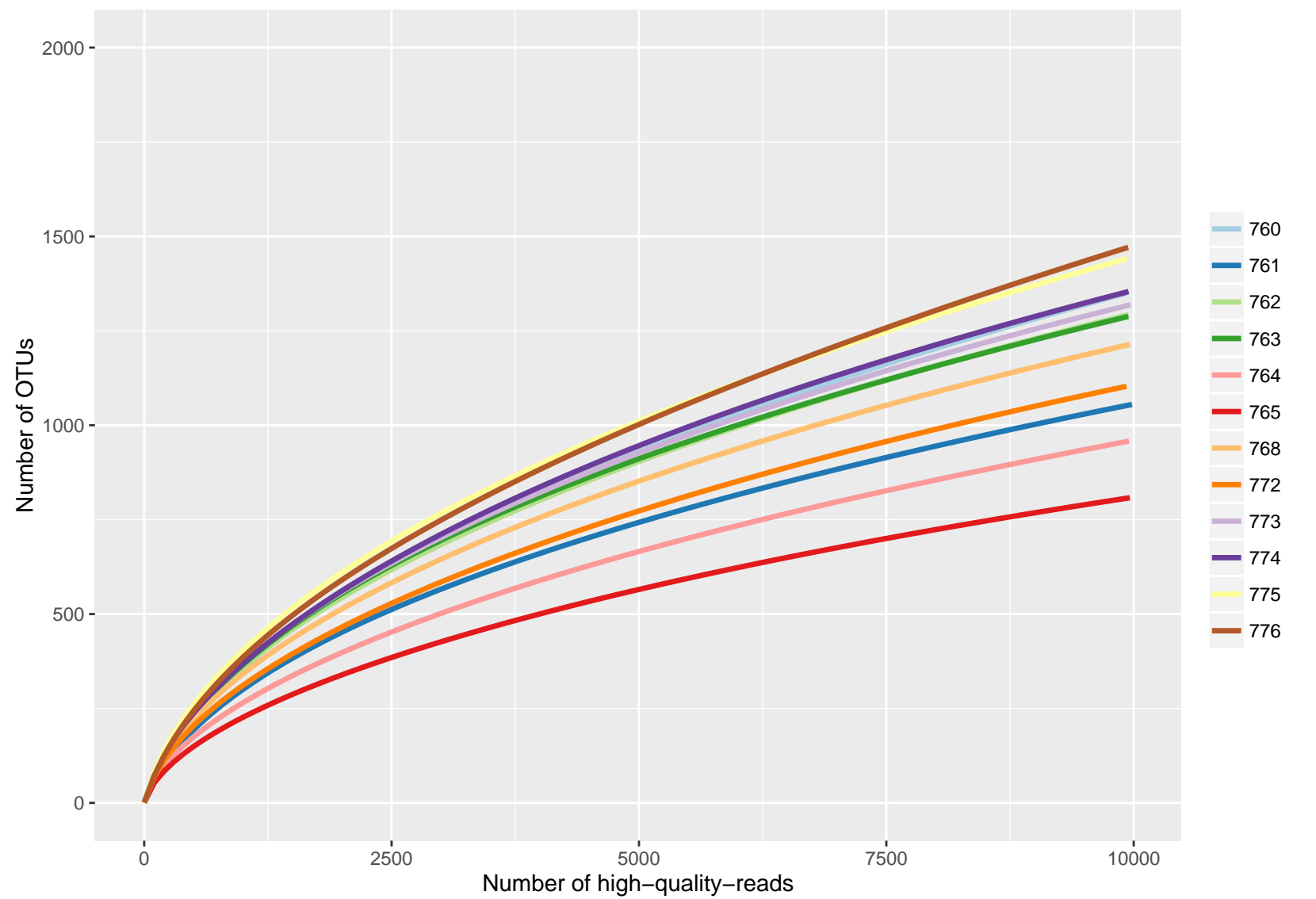

Supplement: S2 File — (ZIP) [file pone.0186766.s008.zip › Rarefact_curves_49.pdf]

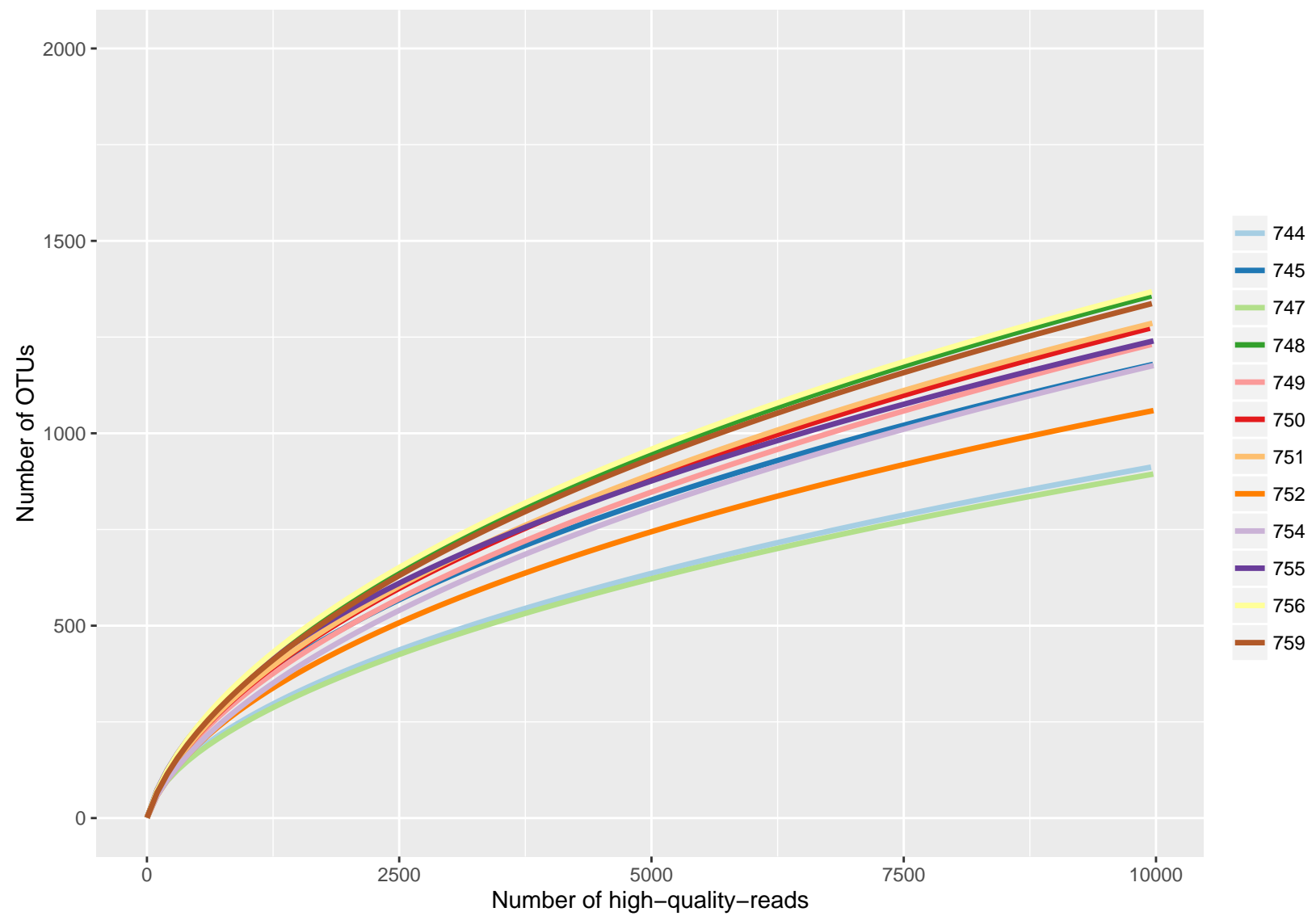

Supplement: S2 File — (ZIP) [file pone.0186766.s008.zip › Rarefact_curves_48.pdf]

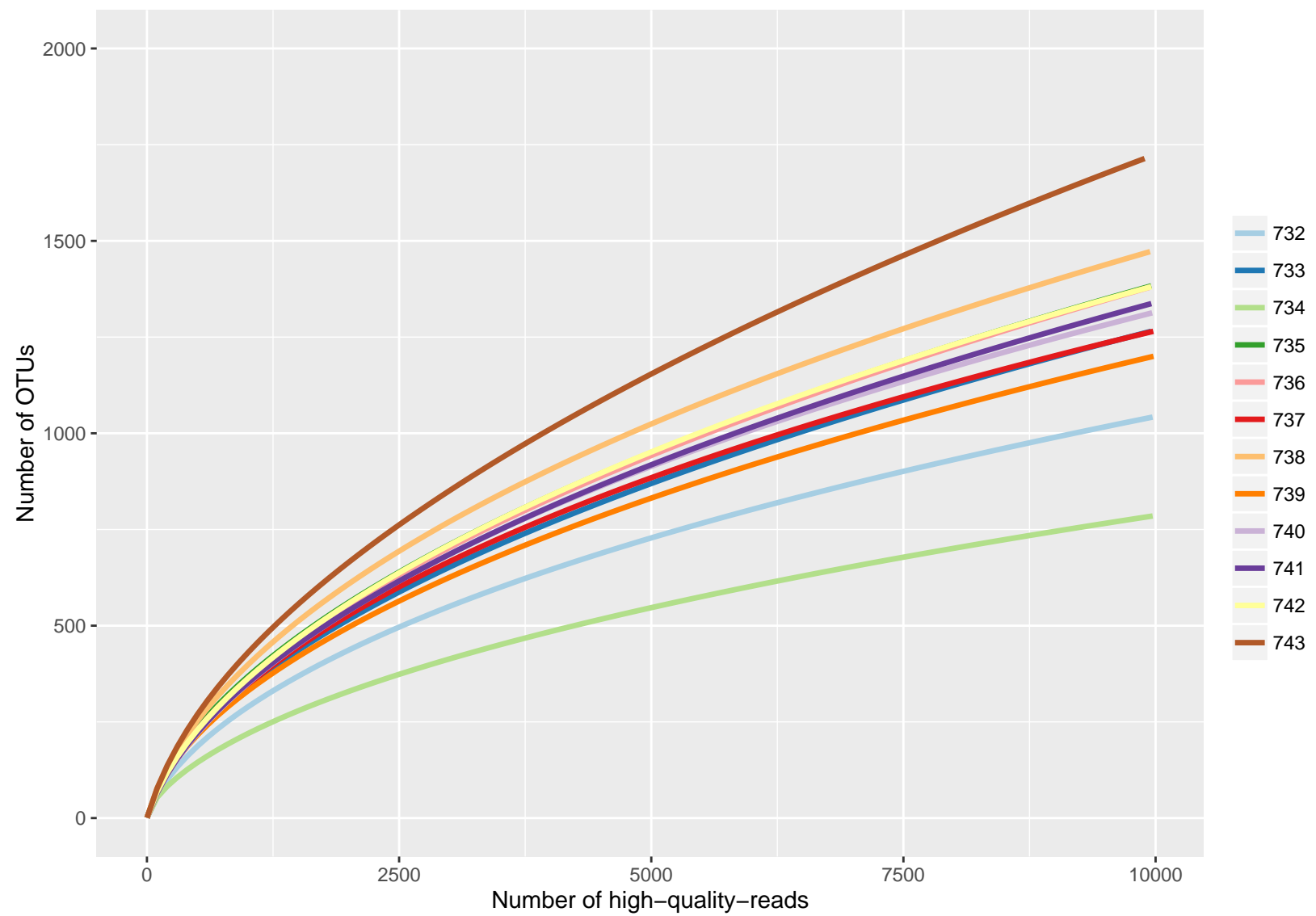

Supplement: S2 File — (ZIP) [file pone.0186766.s008.zip › Rarefact_curves_47.pdf]

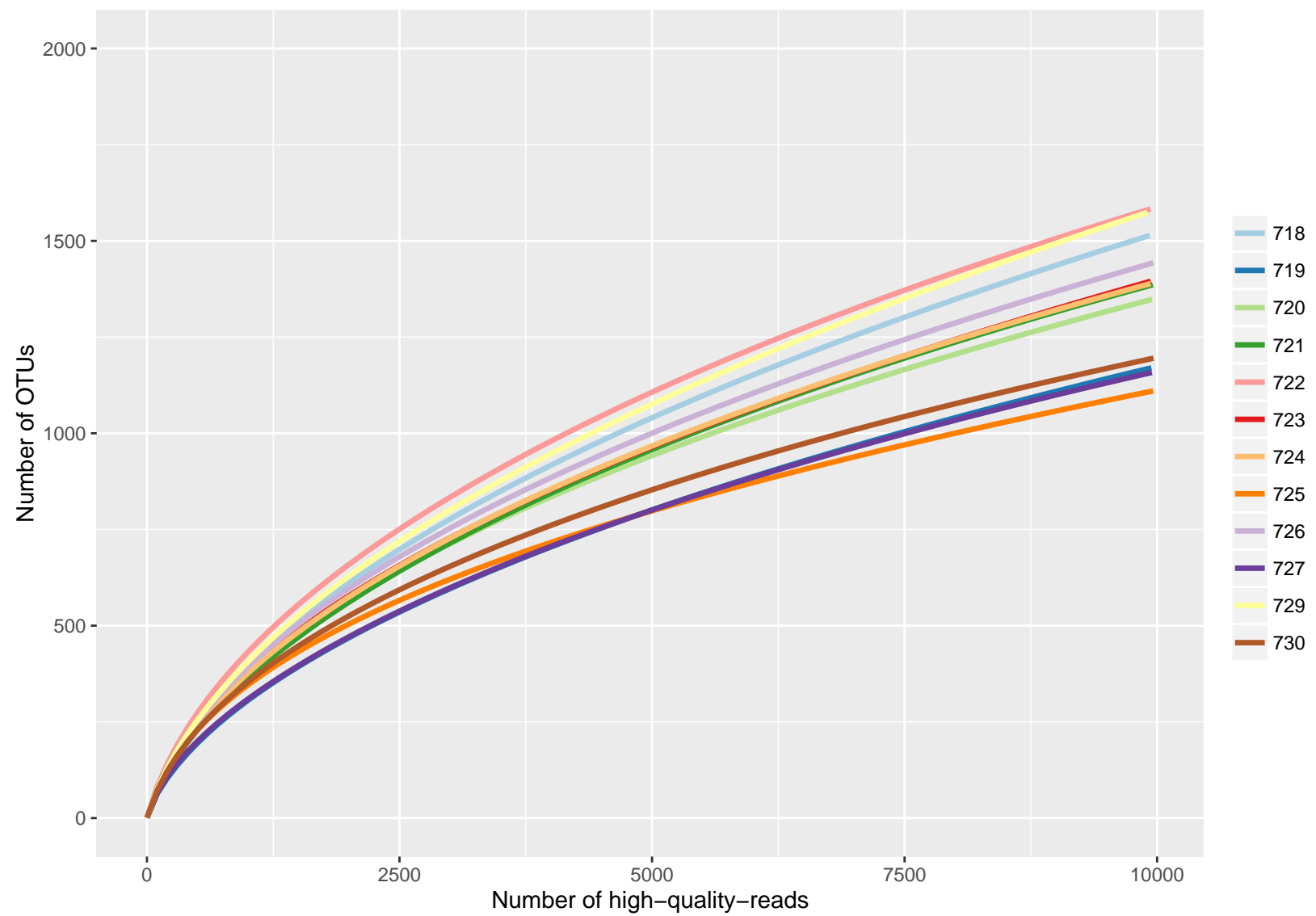

Supplement: S2 File — (ZIP) [file pone.0186766.s008.zip › Rarefact_curves_46.pdf]

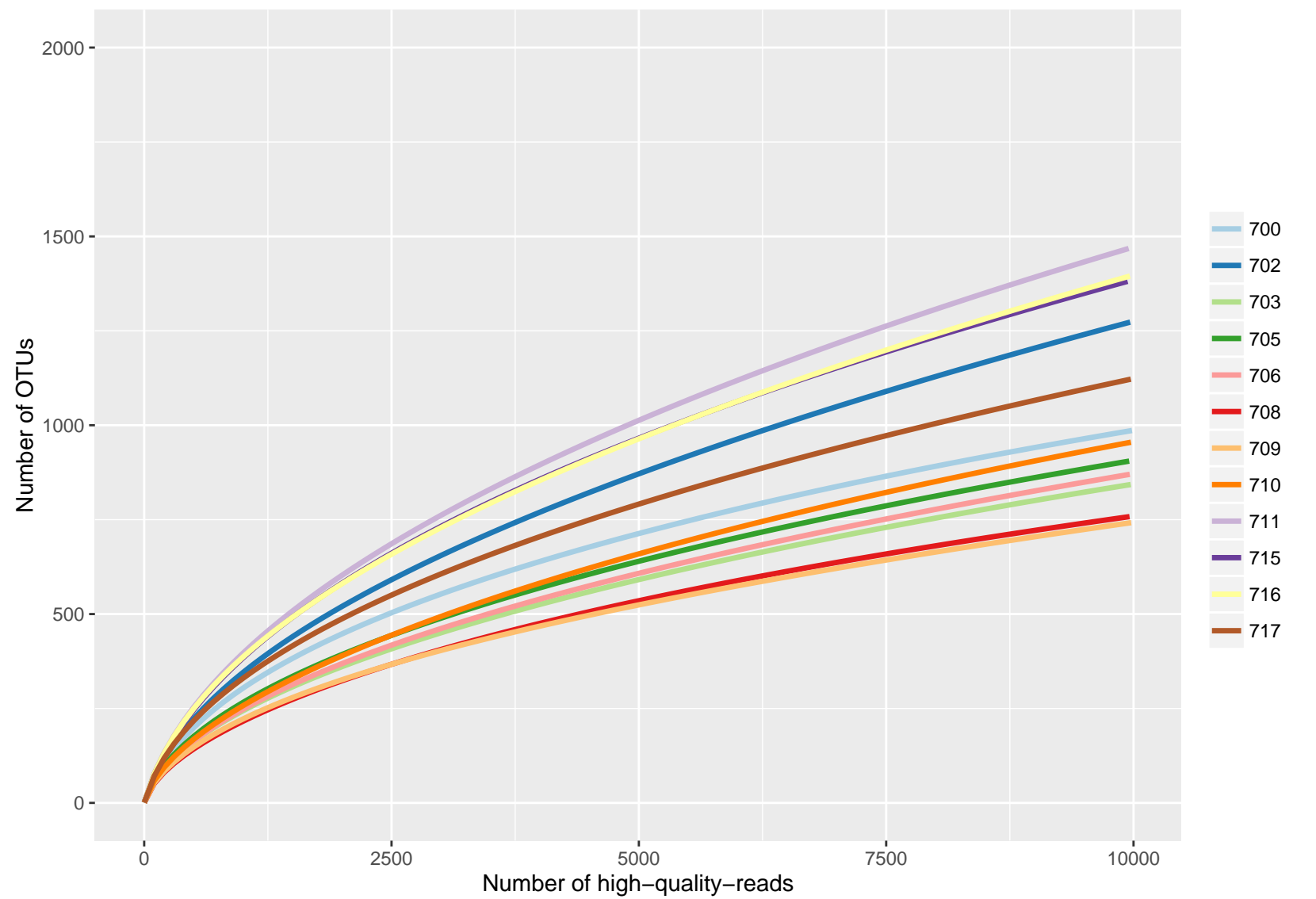

Supplement: S2 File — (ZIP) [file pone.0186766.s008.zip › Rarefact_curves_45.pdf]

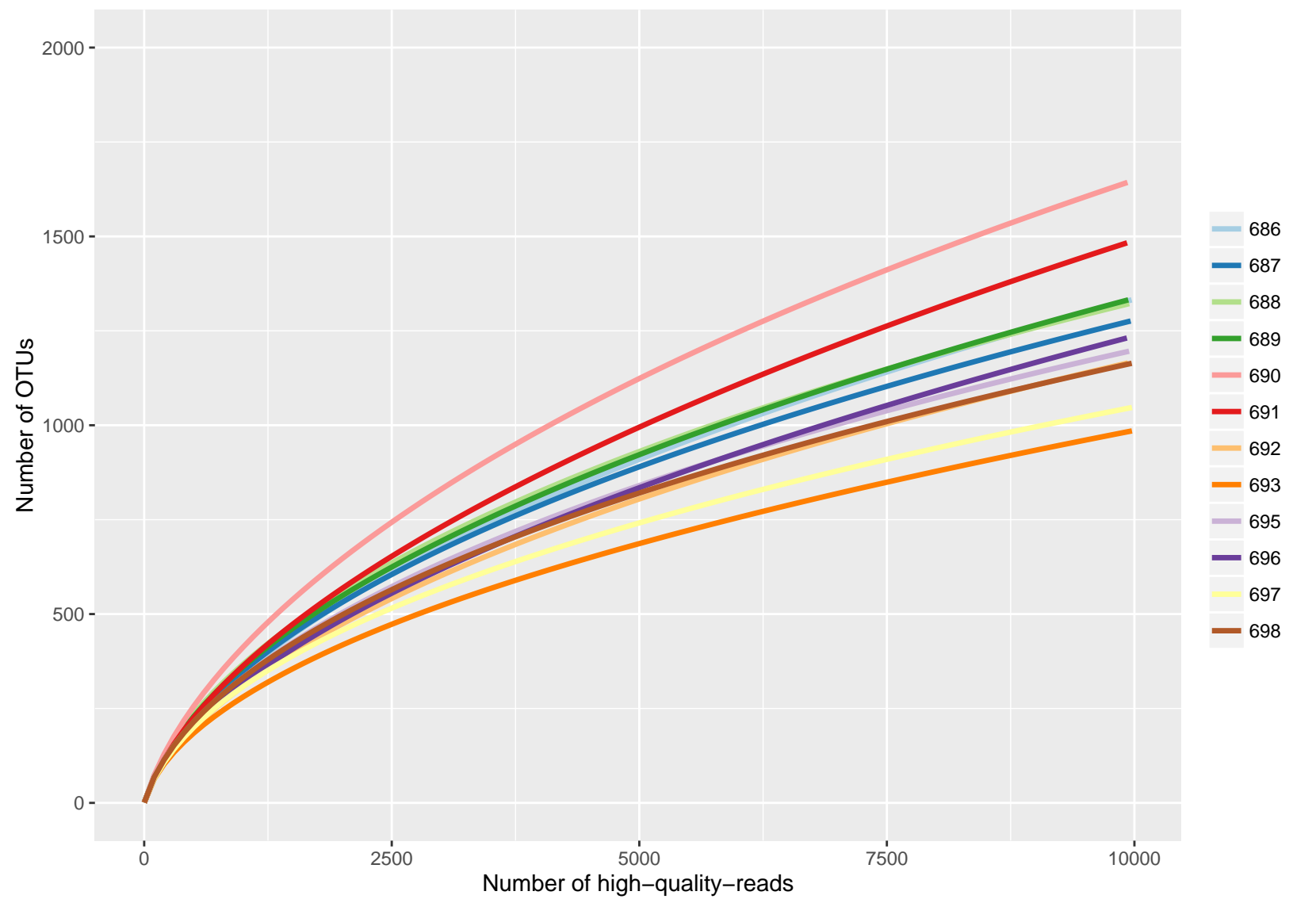

Supplement: S2 File — (ZIP) [file pone.0186766.s008.zip › Rarefact_curves_44.pdf]

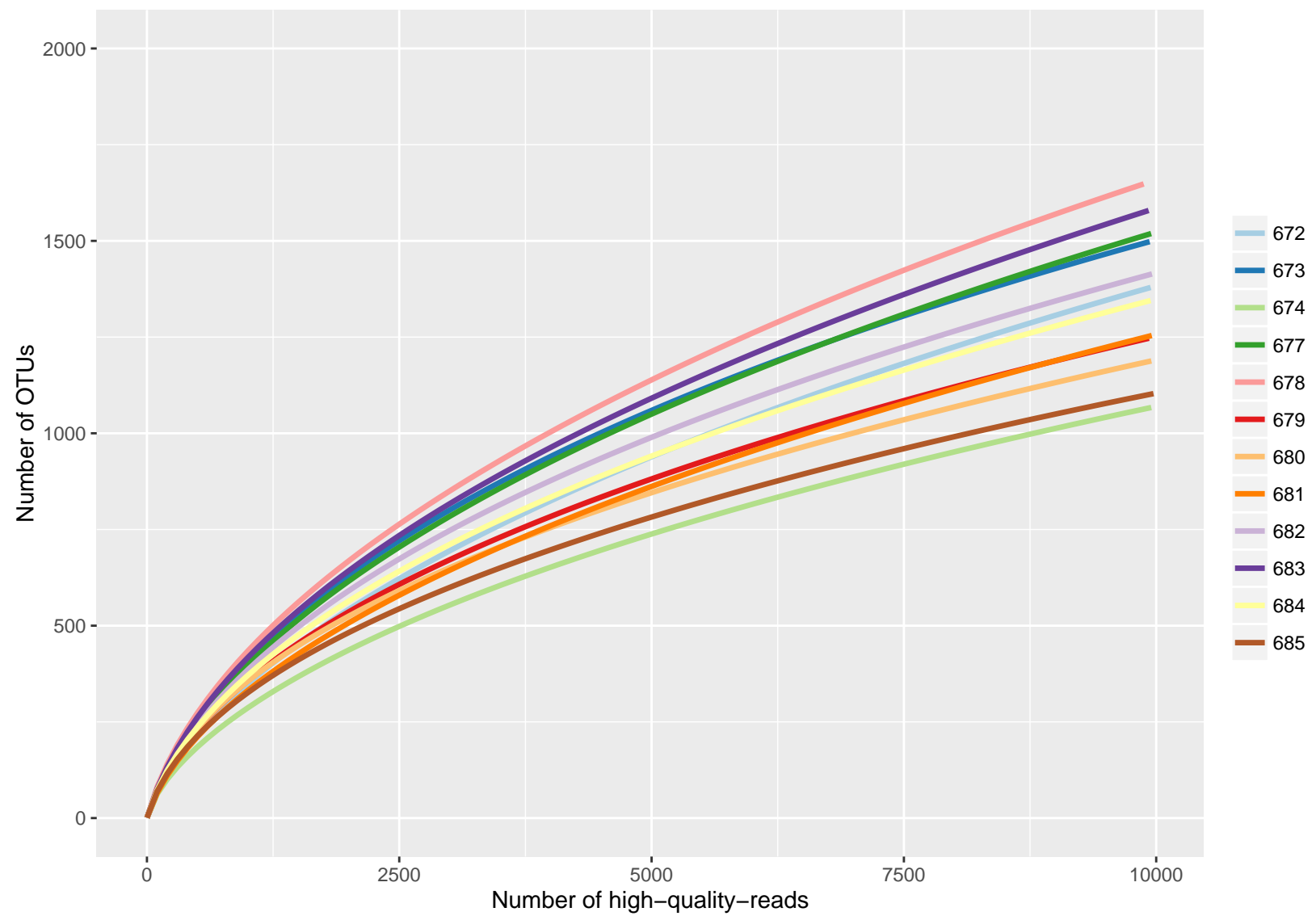

Supplement: S2 File — (ZIP) [file pone.0186766.s008.zip › Rarefact_curves_43.pdf]

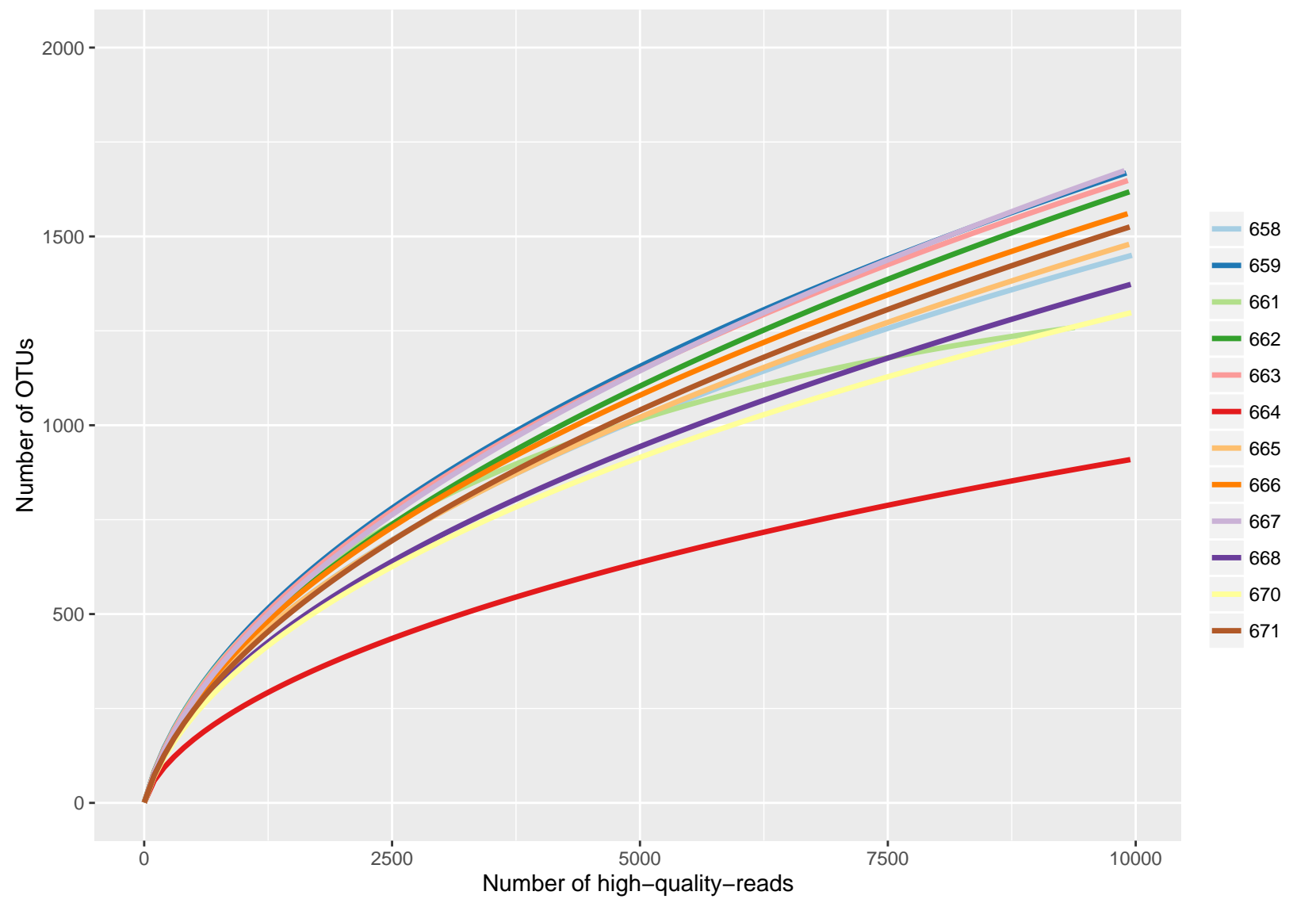

Supplement: S2 File — (ZIP) [file pone.0186766.s008.zip › Rarefact_curves_42.pdf]

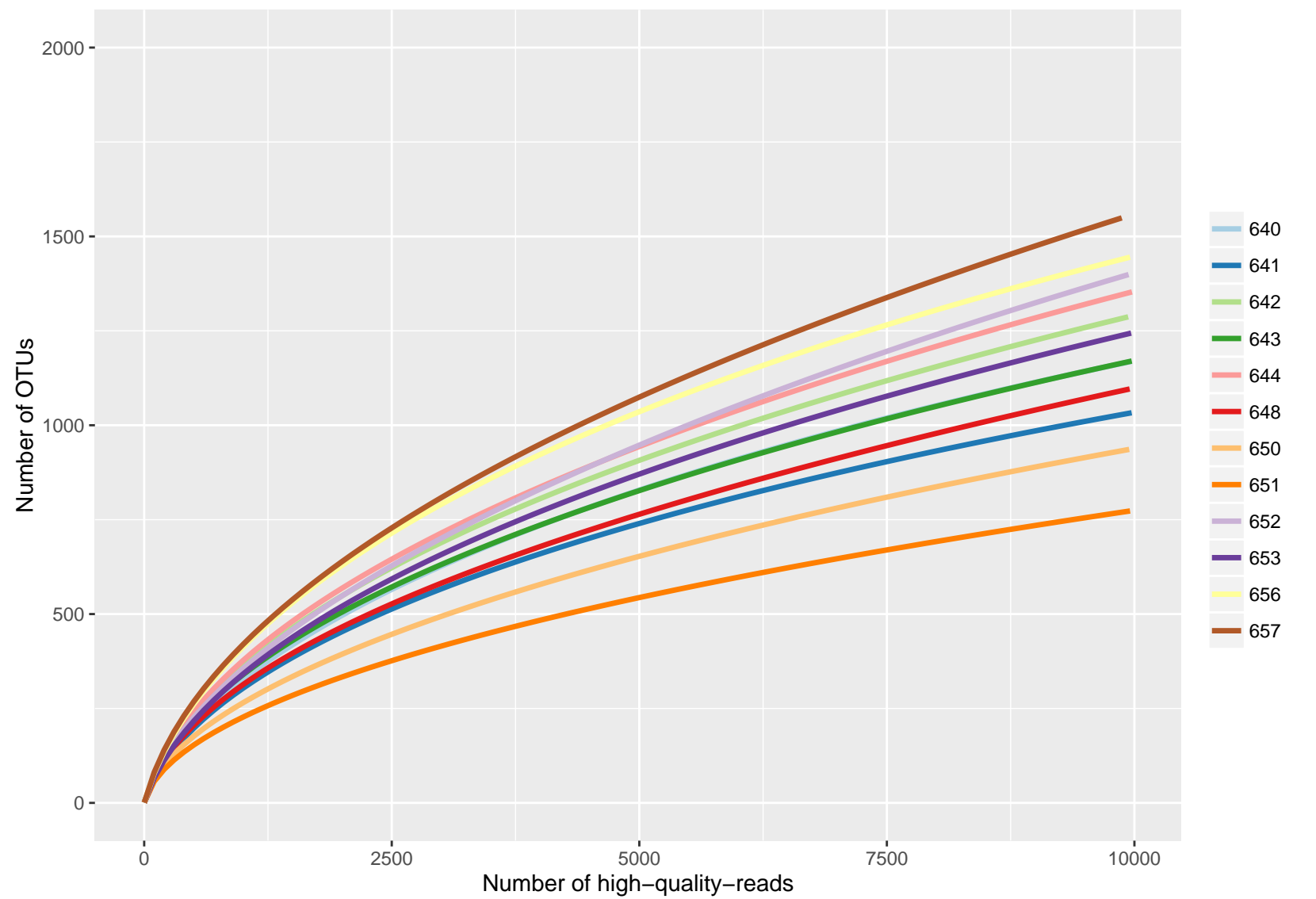

Supplement: S2 File — (ZIP) [file pone.0186766.s008.zip › Rarefact_curves_41.pdf]

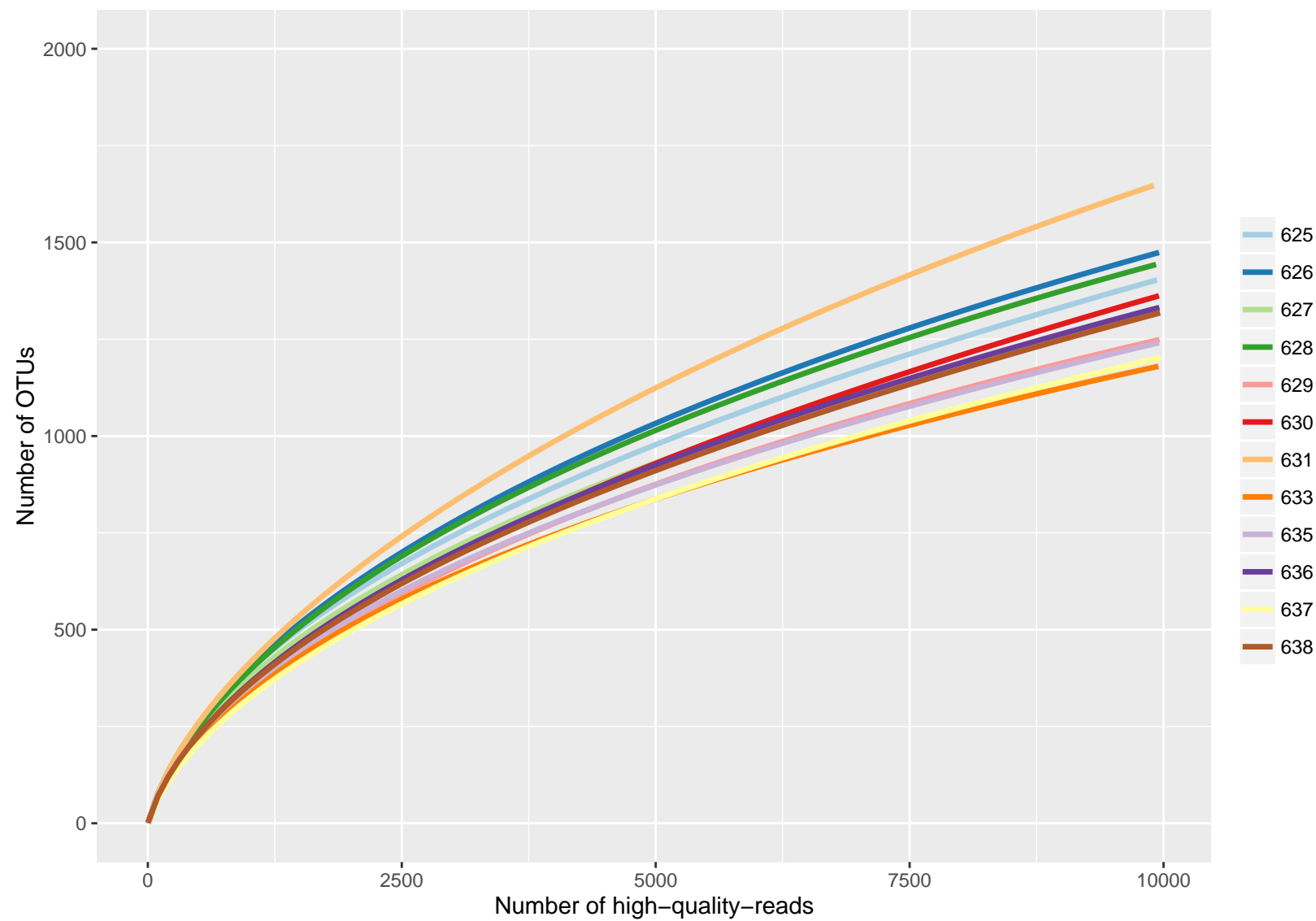

Supplement: S2 File — (ZIP) [file pone.0186766.s008.zip › Rarefact_curves_40.pdf]

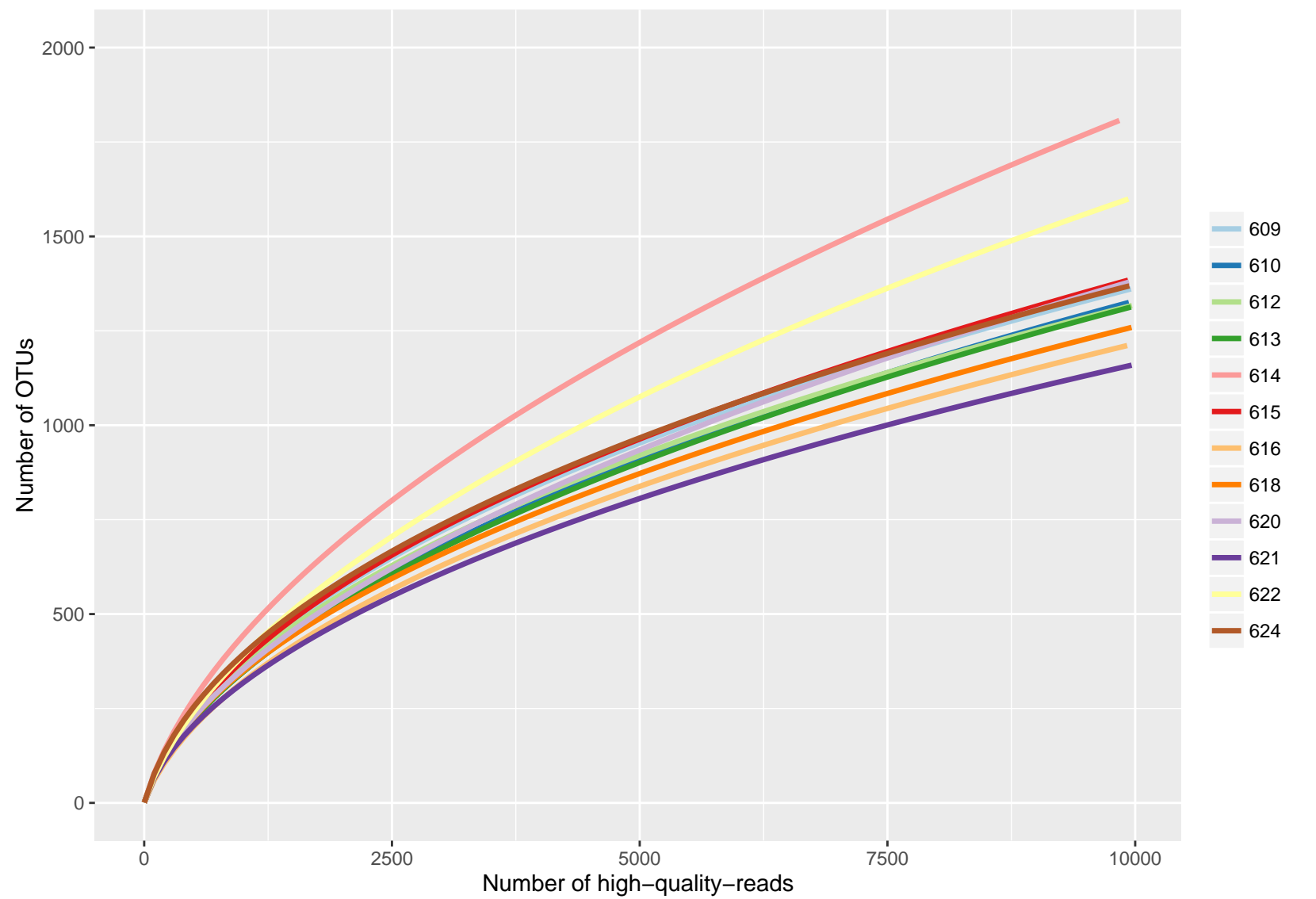

Supplement: S2 File — (ZIP) [file pone.0186766.s008.zip › Rarefact_curves_39.pdf]

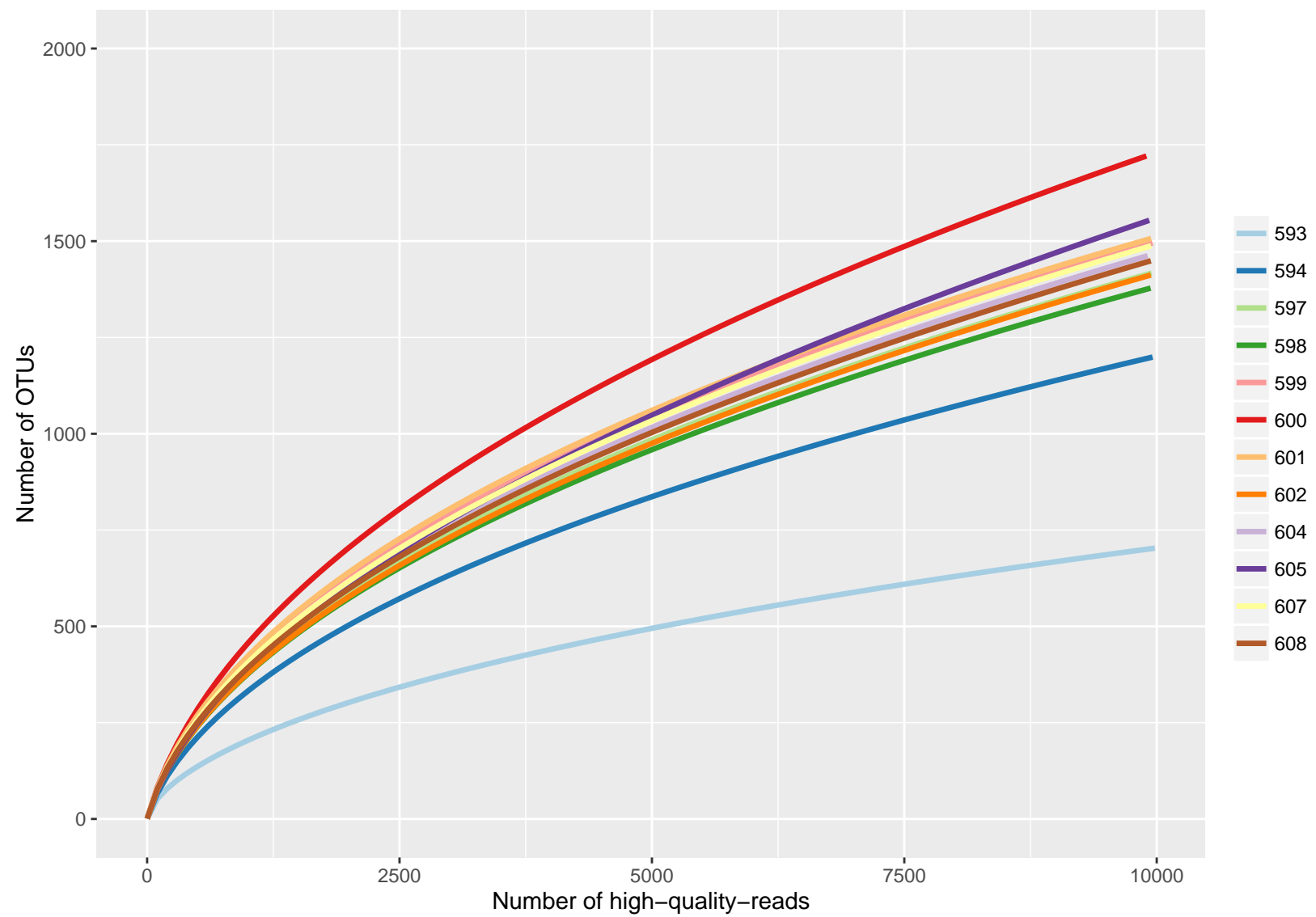

Supplement: S2 File — (ZIP) [file pone.0186766.s008.zip › Rarefact_curves_38.pdf]

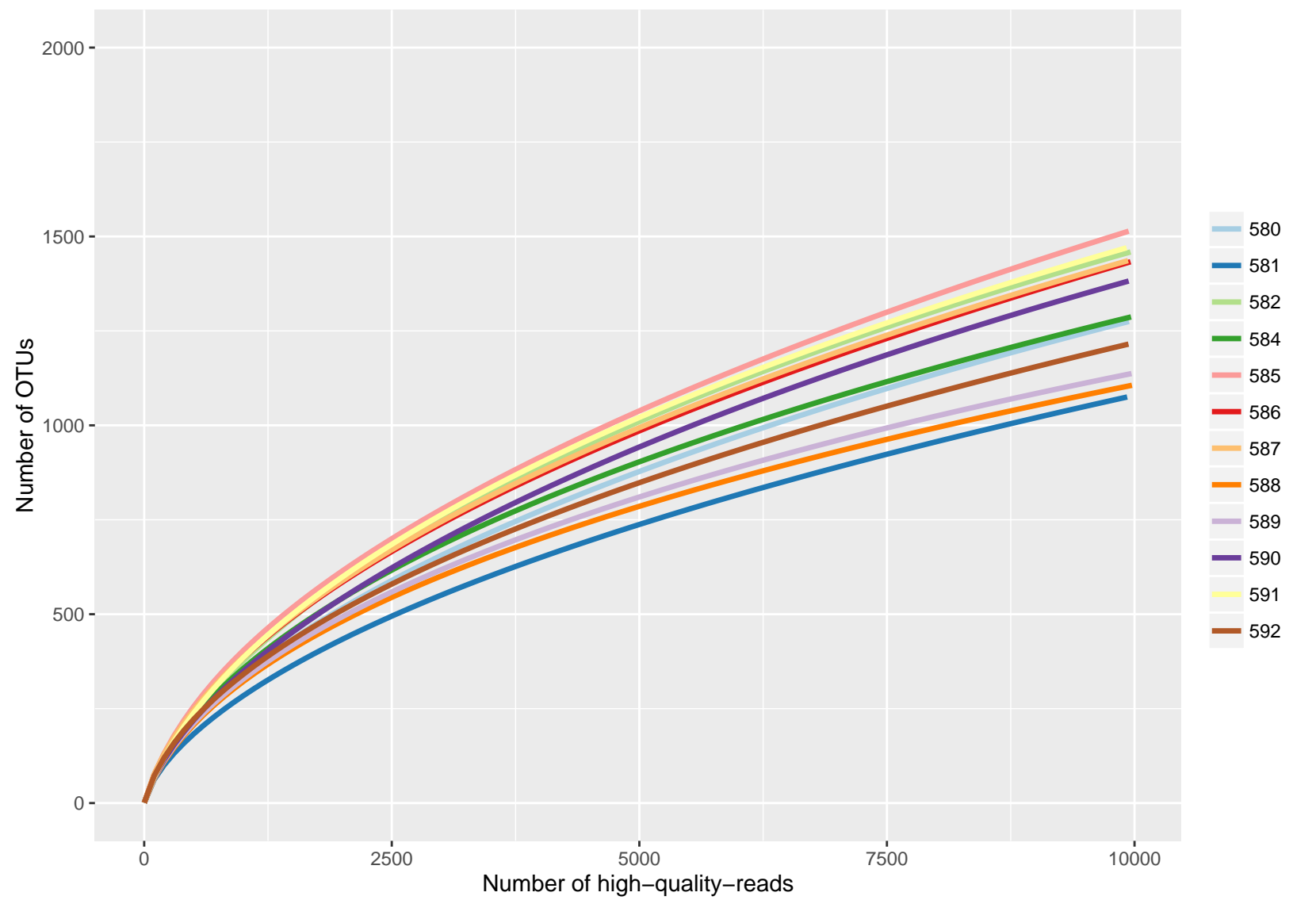

Supplement: S2 File — (ZIP) [file pone.0186766.s008.zip › Rarefact_curves_37.pdf]

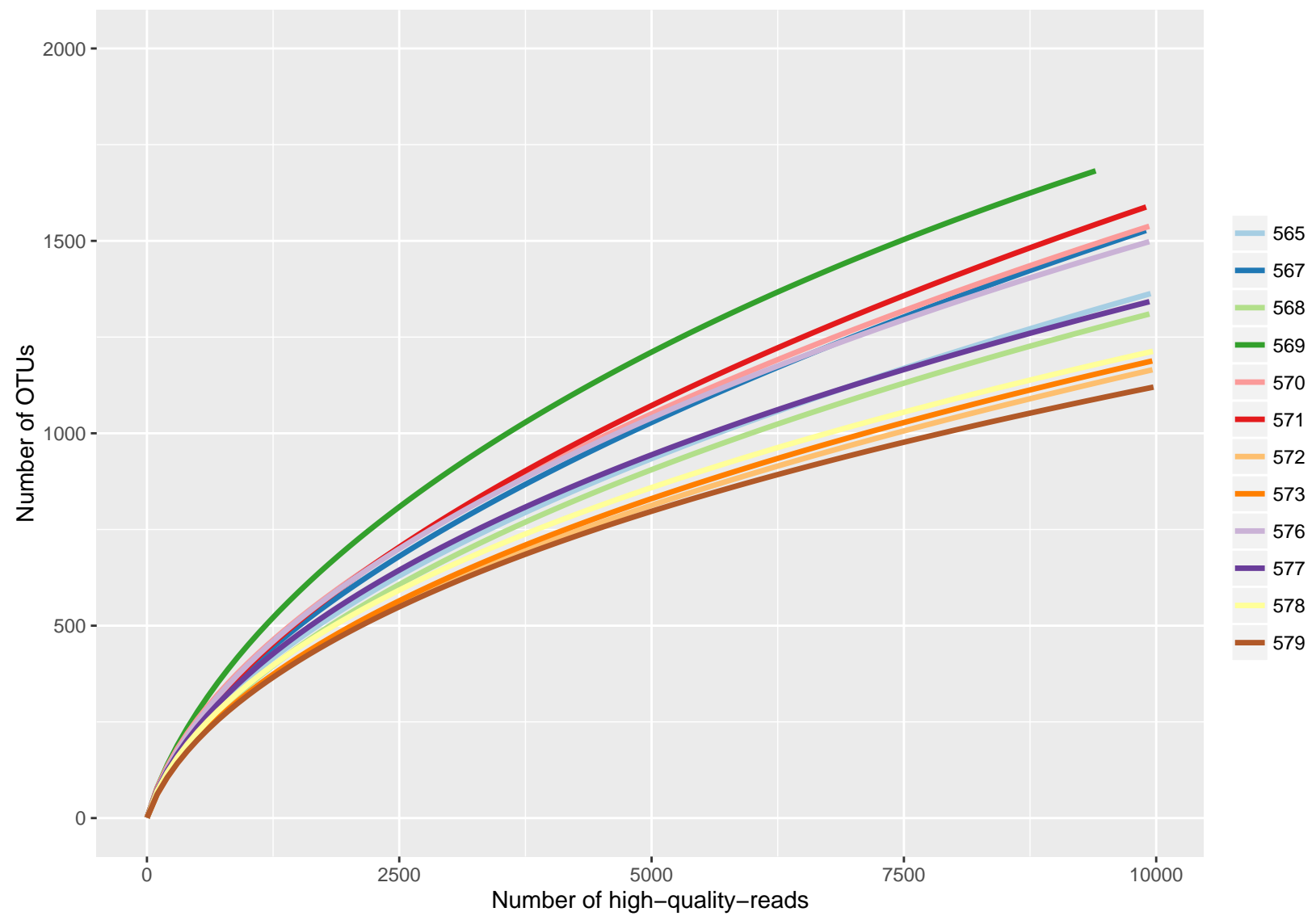

Supplement: S2 File — (ZIP) [file pone.0186766.s008.zip › Rarefact_curves_36.pdf]

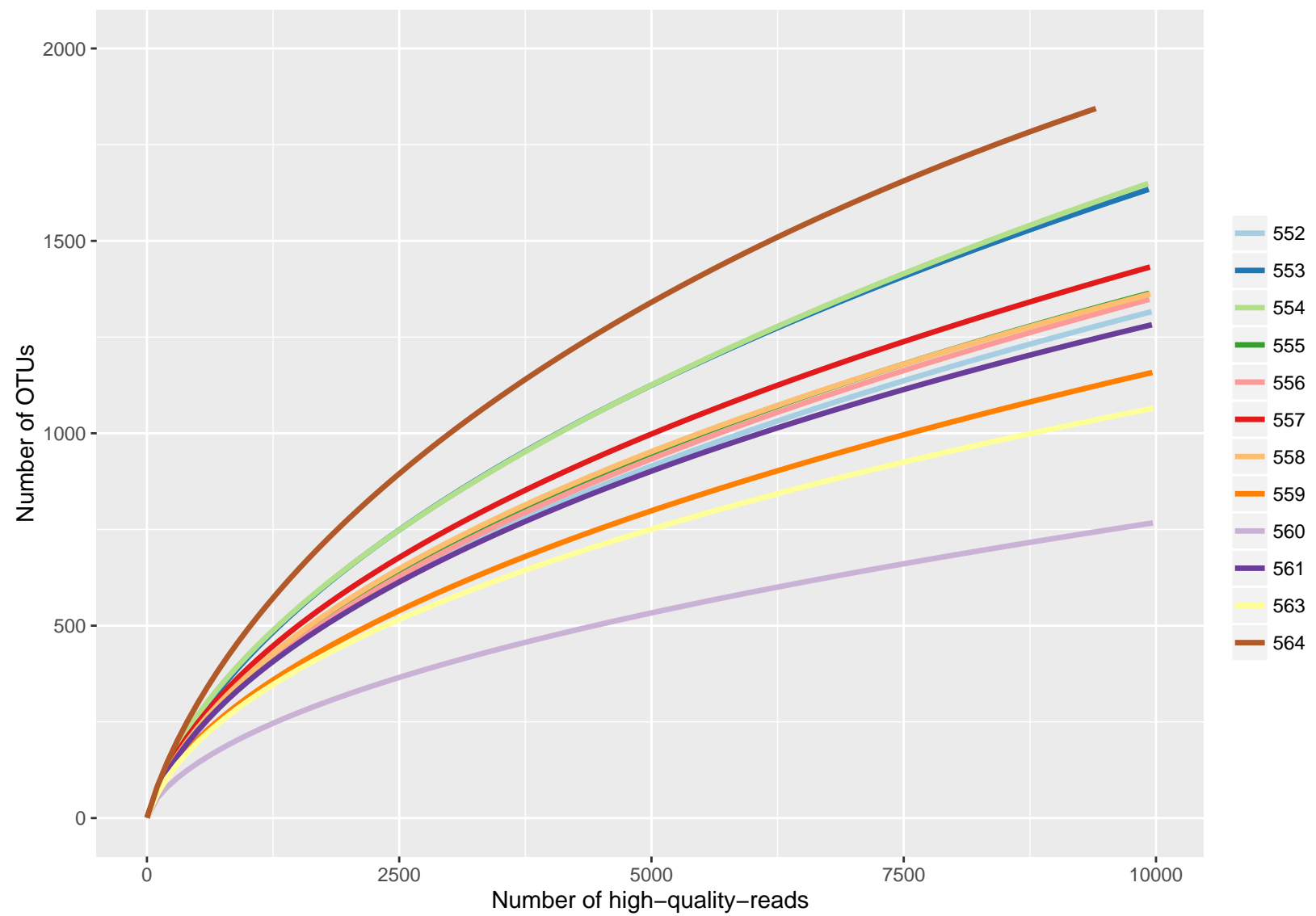

Supplement: S2 File — (ZIP) [file pone.0186766.s008.zip › Rarefact_curves_35.pdf]

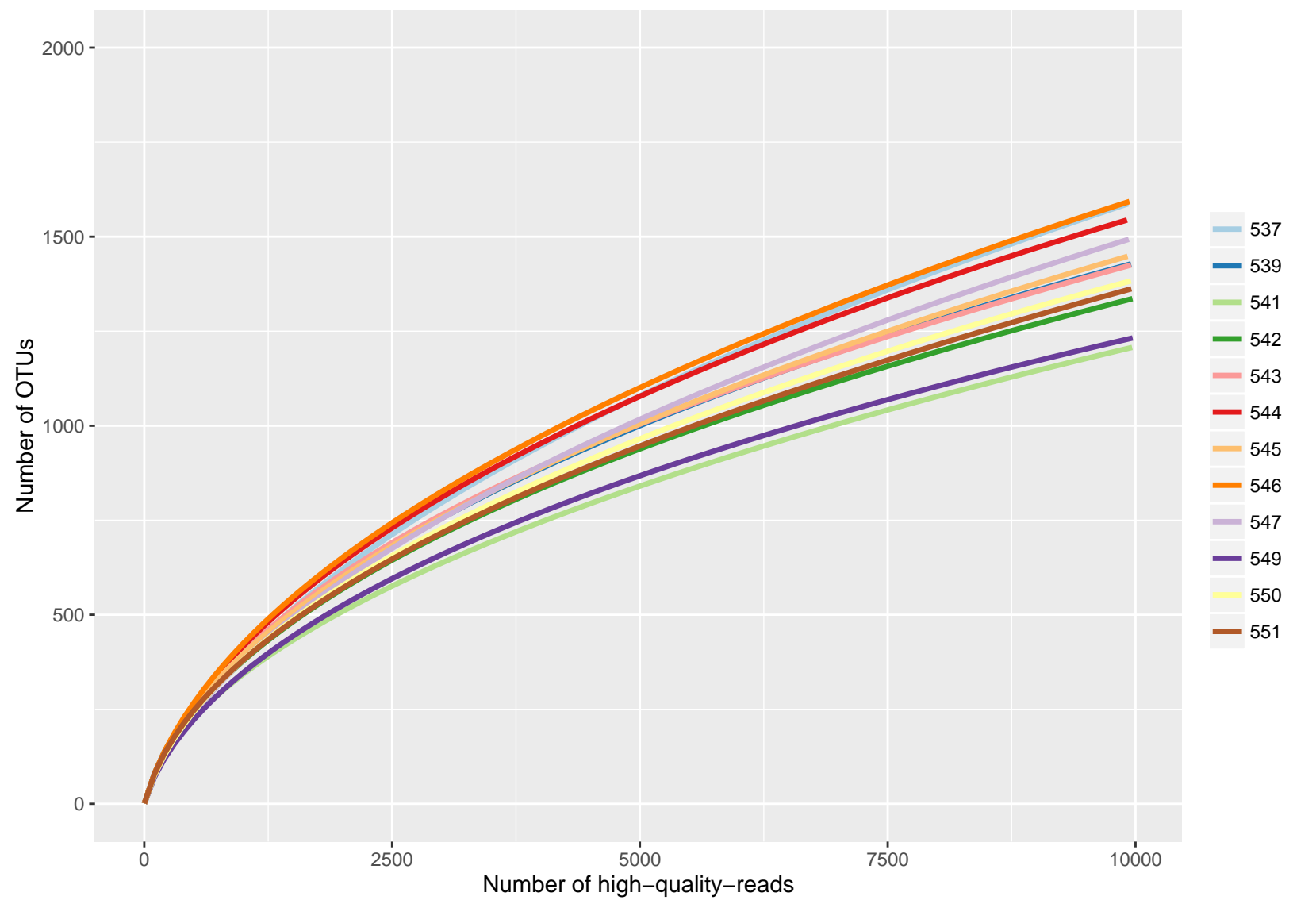

Supplement: S2 File — (ZIP) [file pone.0186766.s008.zip › Rarefact_curves_34.pdf]

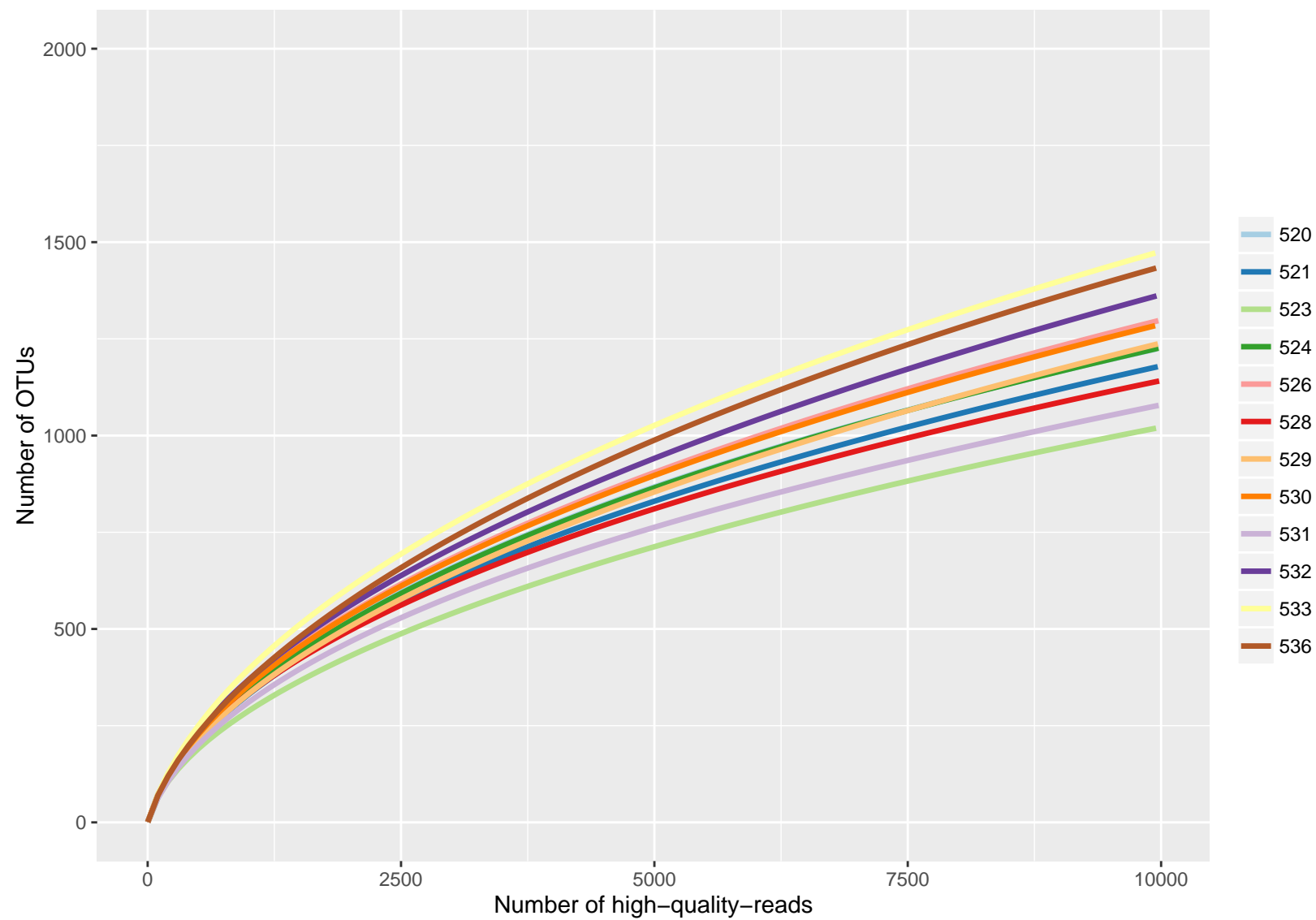

Supplement: S2 File — (ZIP) [file pone.0186766.s008.zip › Rarefact_curves_33.pdf]

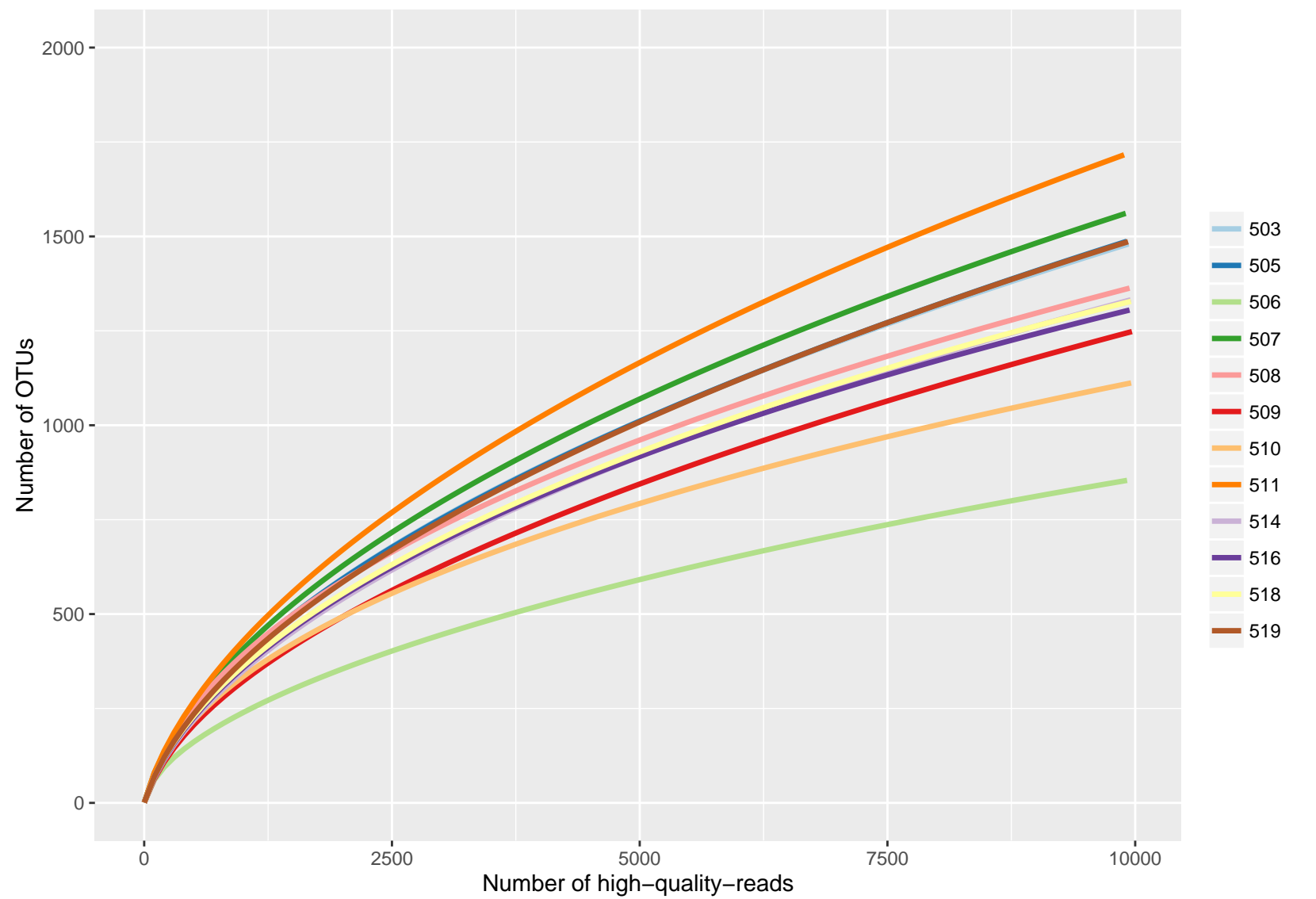

Supplement: S2 File — (ZIP) [file pone.0186766.s008.zip › Rarefact_curves_32.pdf]

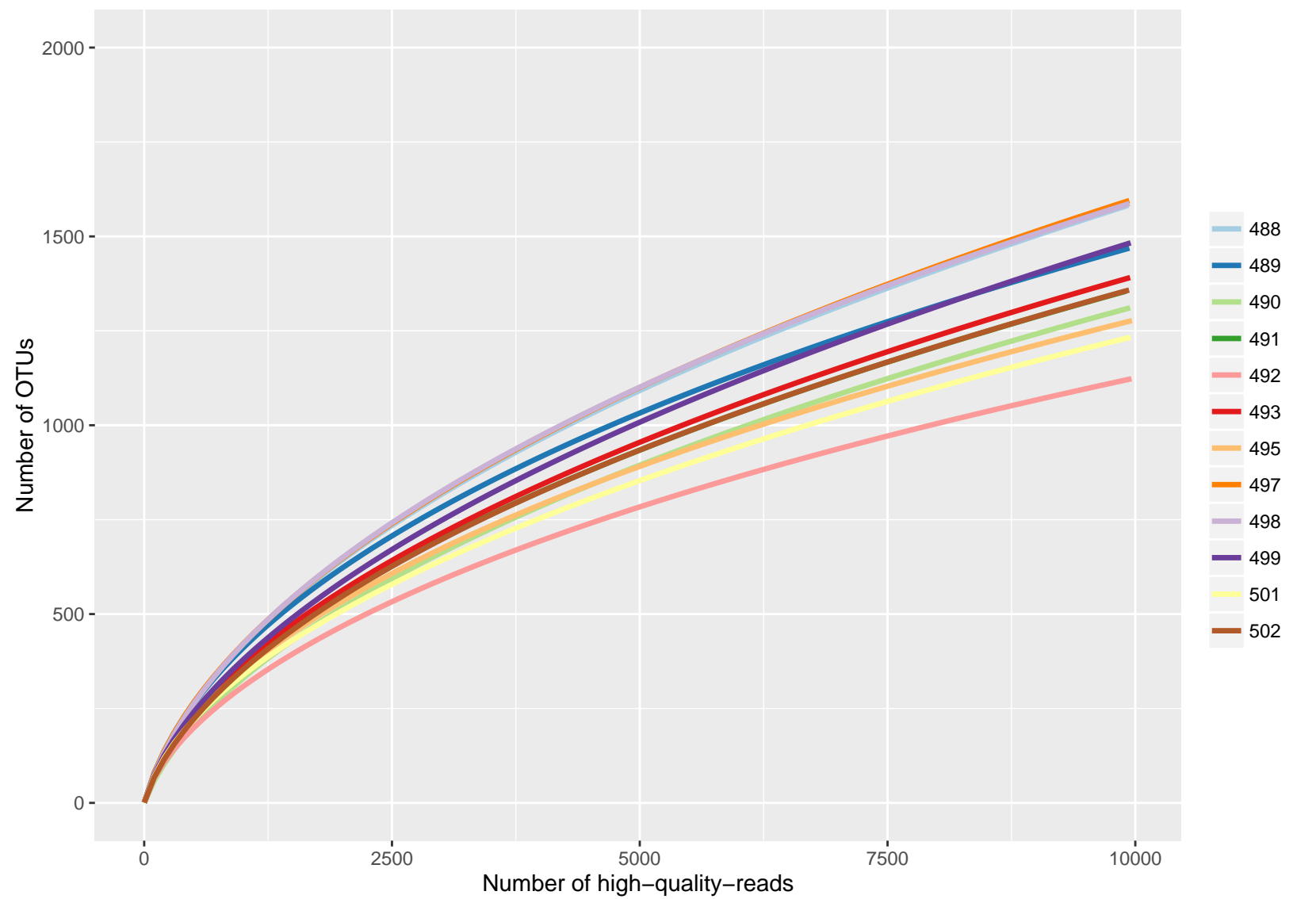

Supplement: S2 File — (ZIP) [file pone.0186766.s008.zip › Rarefact_curves_31.pdf]

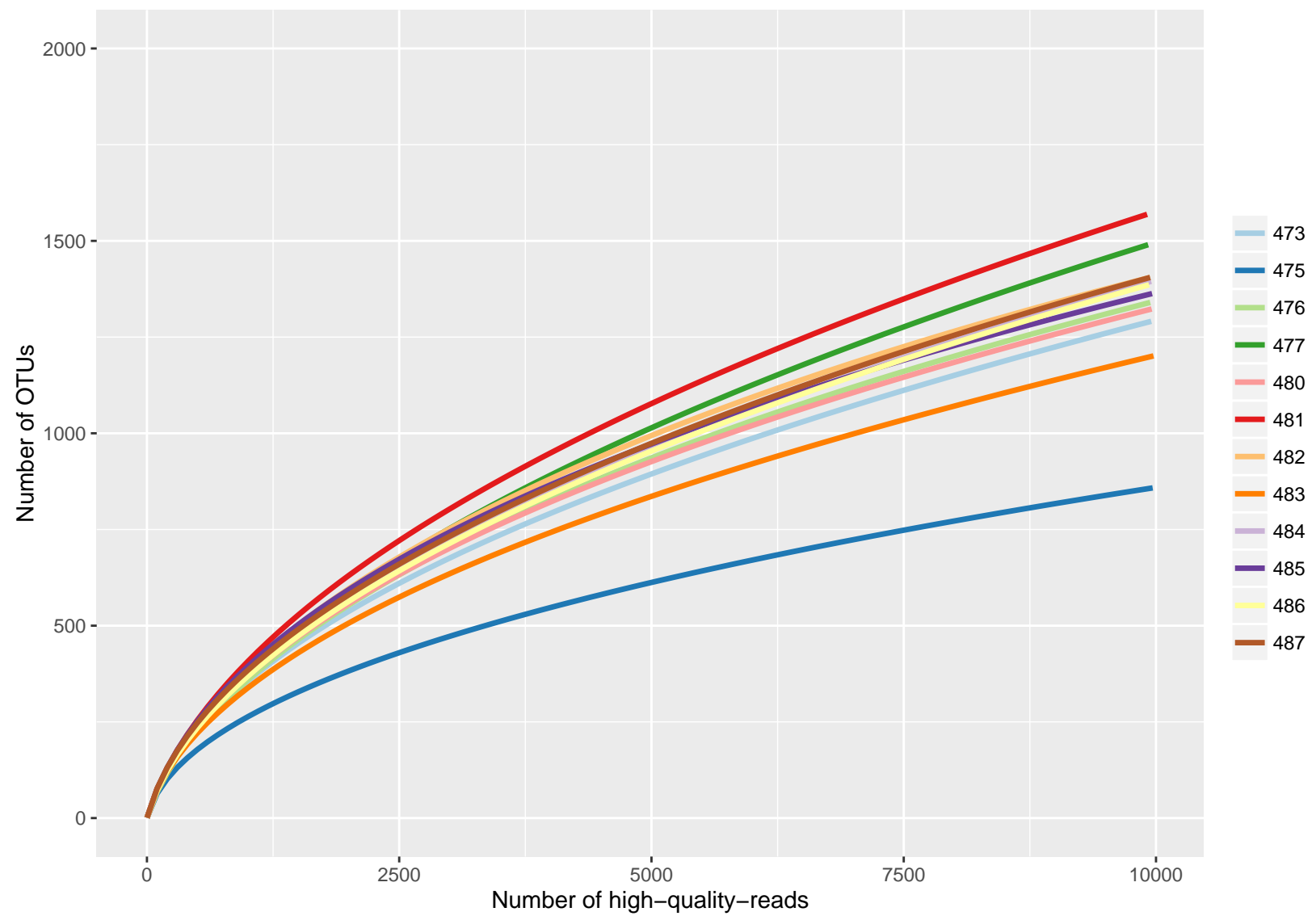

Supplement: S2 File — (ZIP) [file pone.0186766.s008.zip › Rarefact_curves_30.pdf]

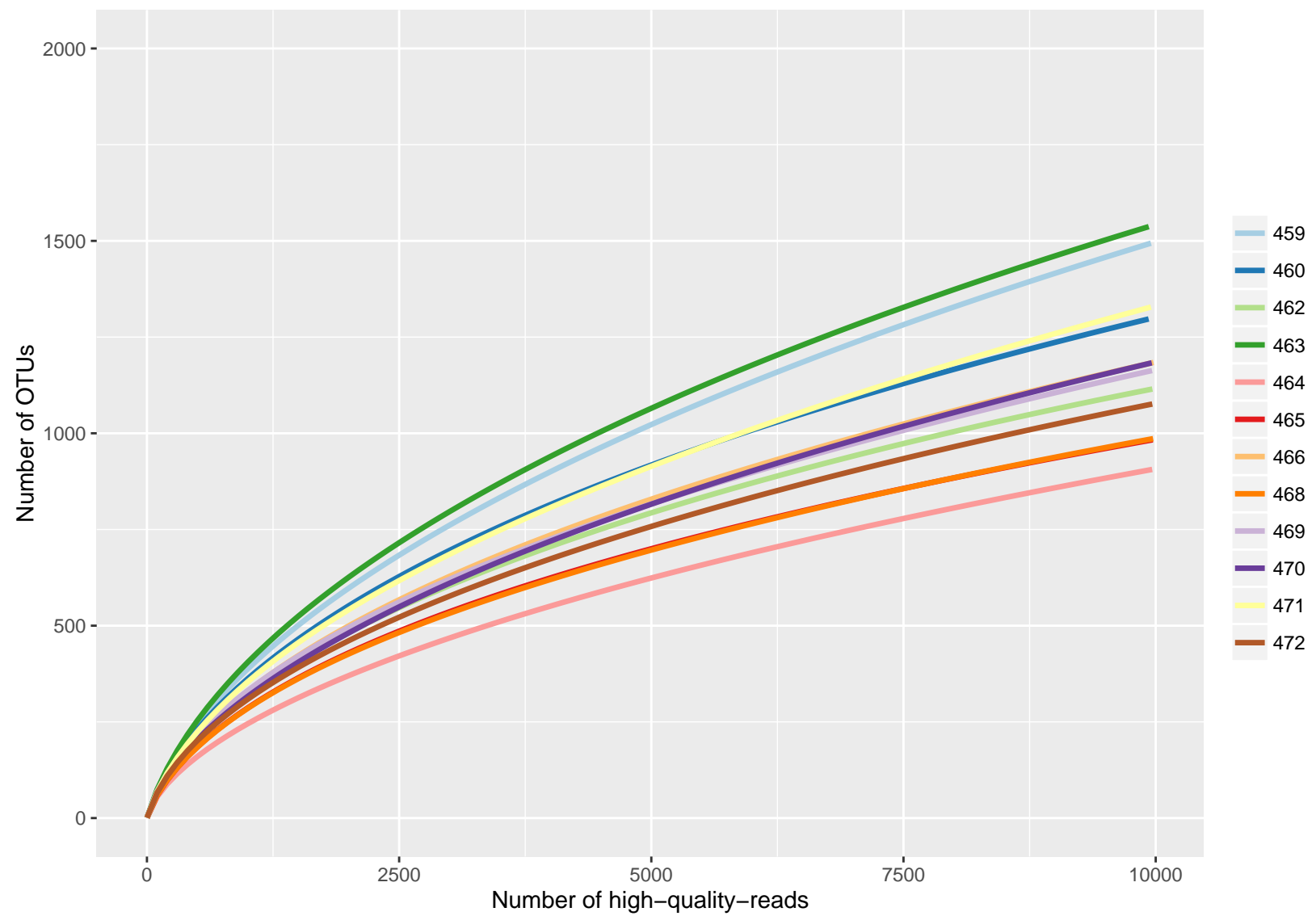

Supplement: S2 File — (ZIP) [file pone.0186766.s008.zip › Rarefact_curves_29.pdf]

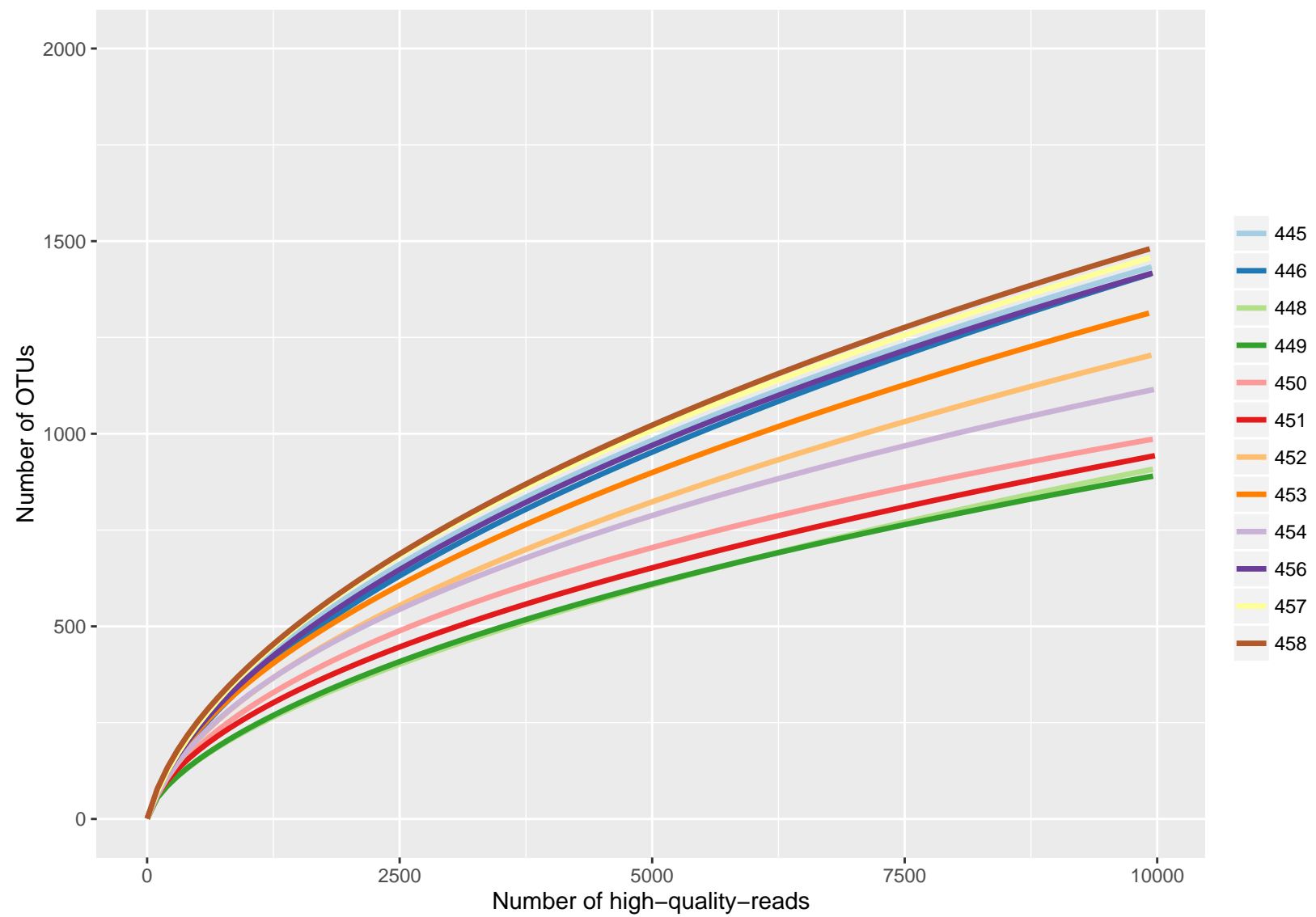

Supplement: S2 File — (ZIP) [file pone.0186766.s008.zip › Rarefact_curves_28.pdf]

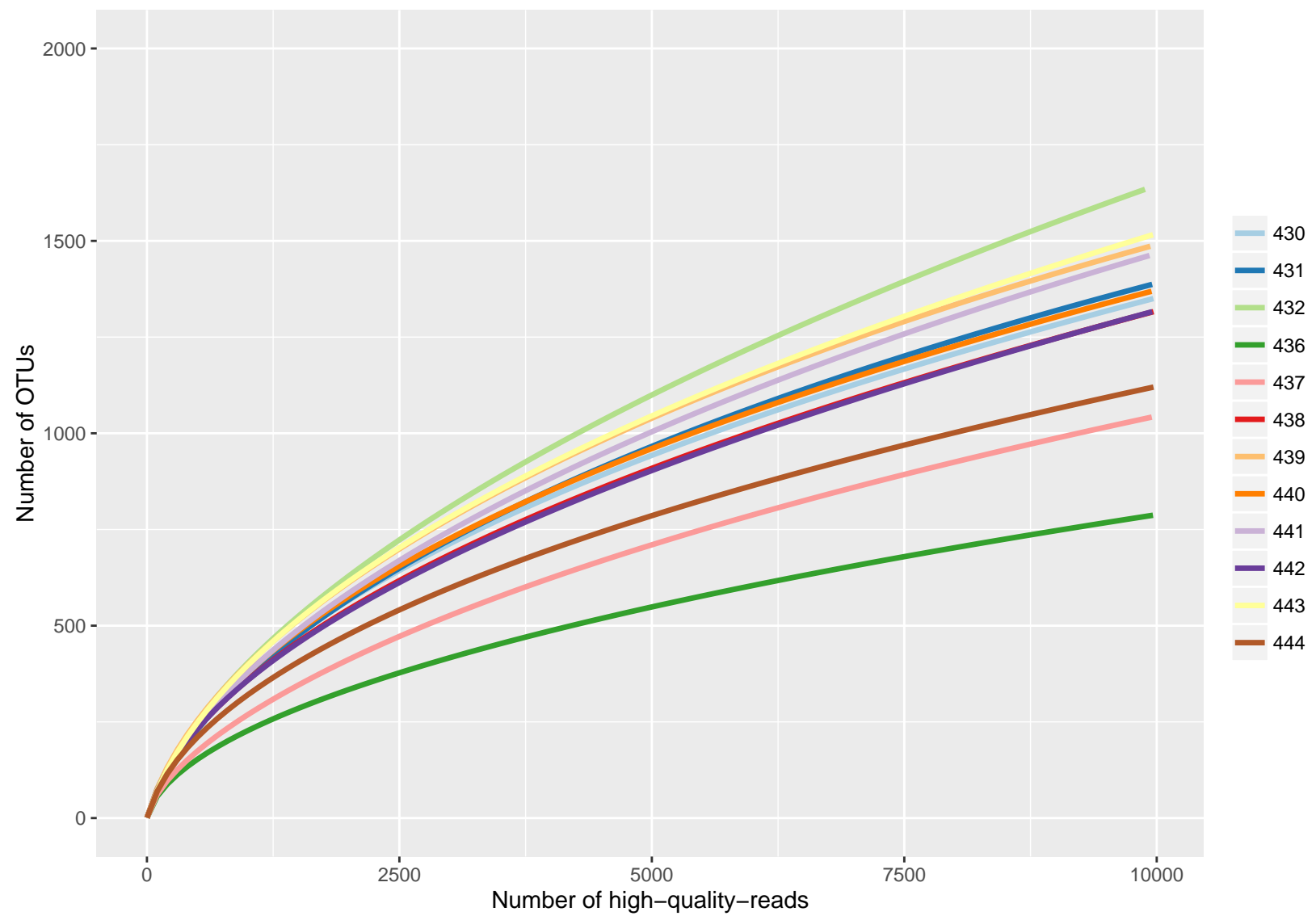

Supplement: S2 File — (ZIP) [file pone.0186766.s008.zip › Rarefact_curves_27.pdf]

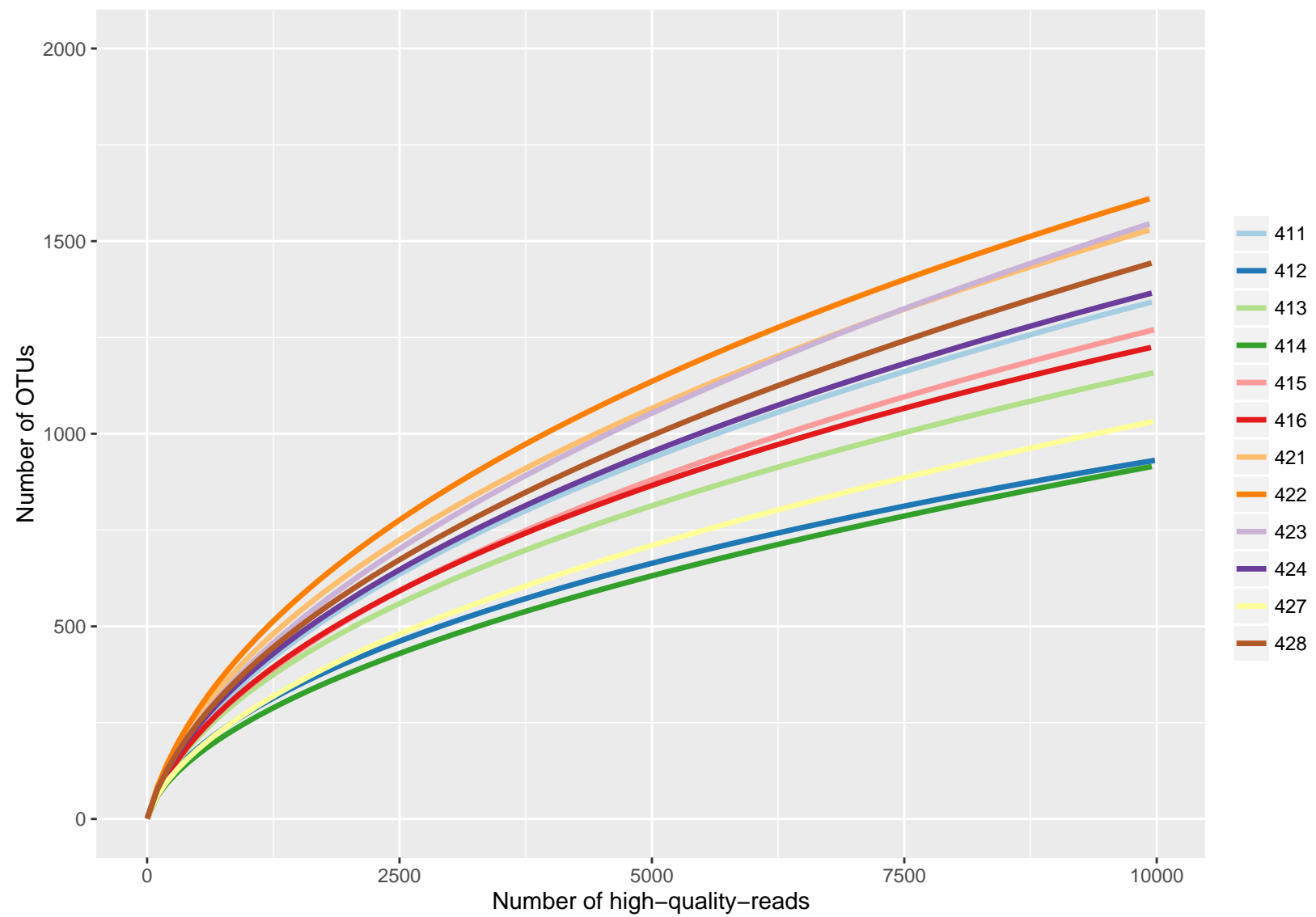

Supplement: S2 File — (ZIP) [file pone.0186766.s008.zip › Rarefact_curves_26.pdf]

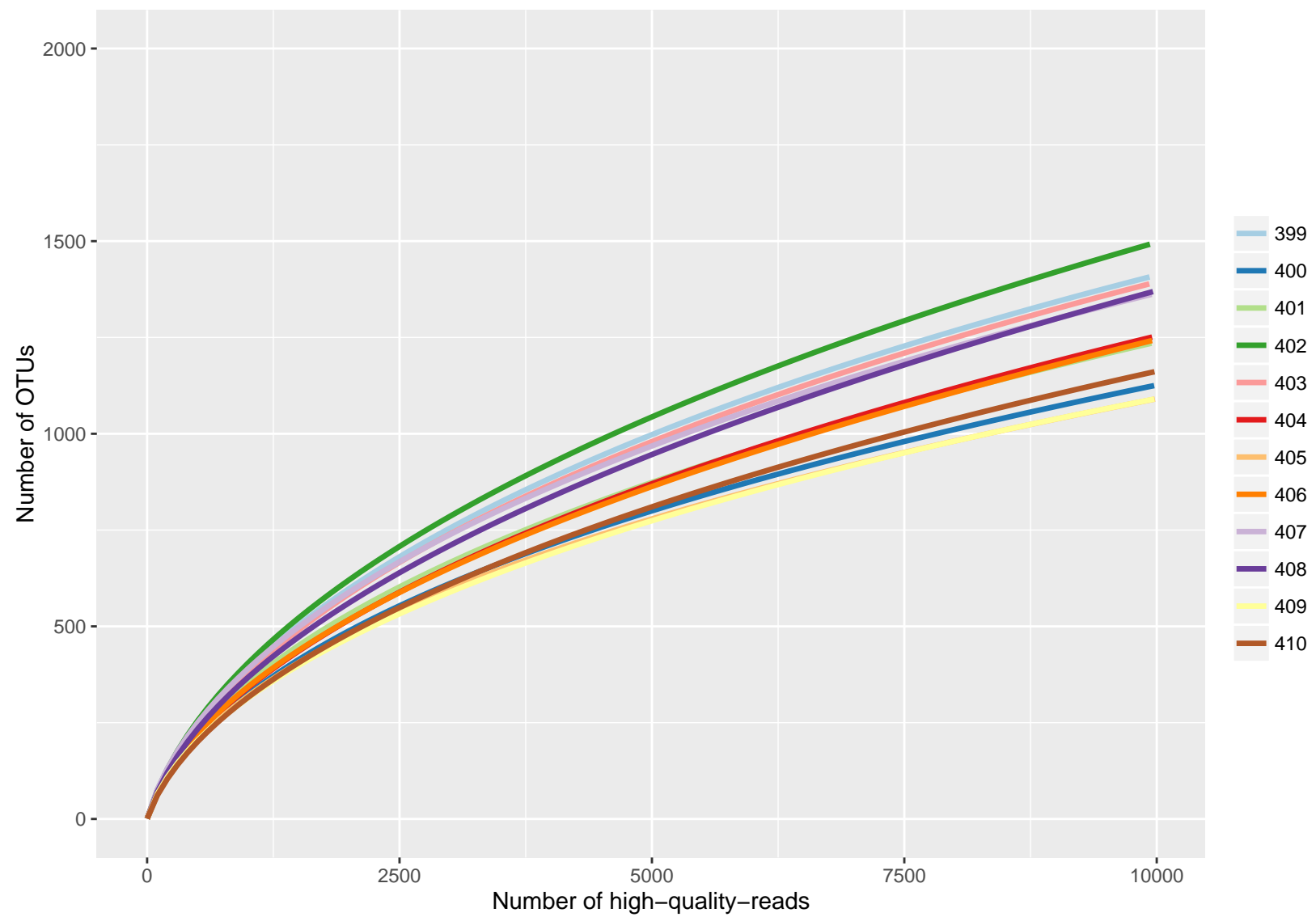

Supplement: S2 File — (ZIP) [file pone.0186766.s008.zip › Rarefact_curves_25.pdf]

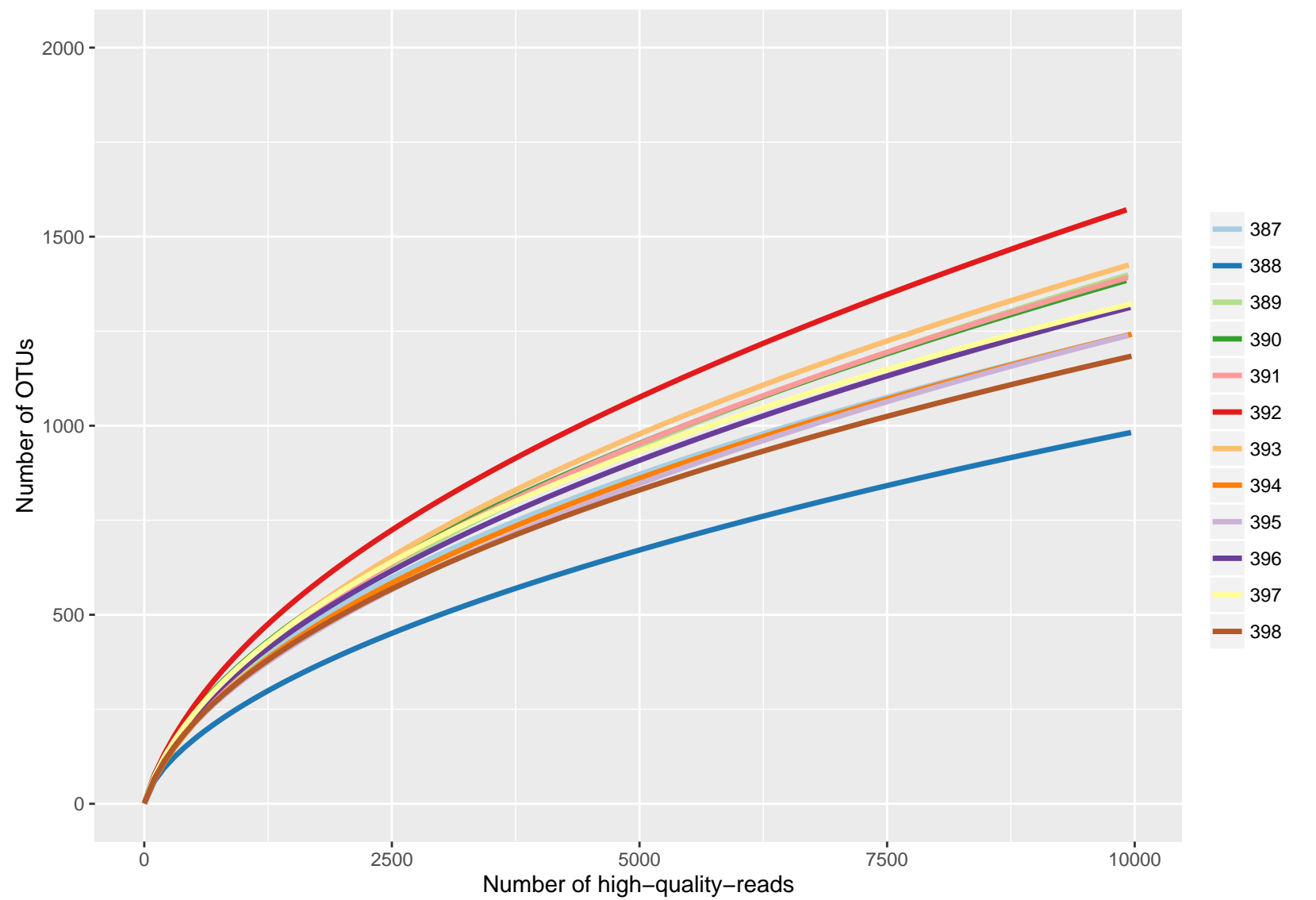

Supplement: S2 File — (ZIP) [file pone.0186766.s008.zip › Rarefact_curves_24.pdf]

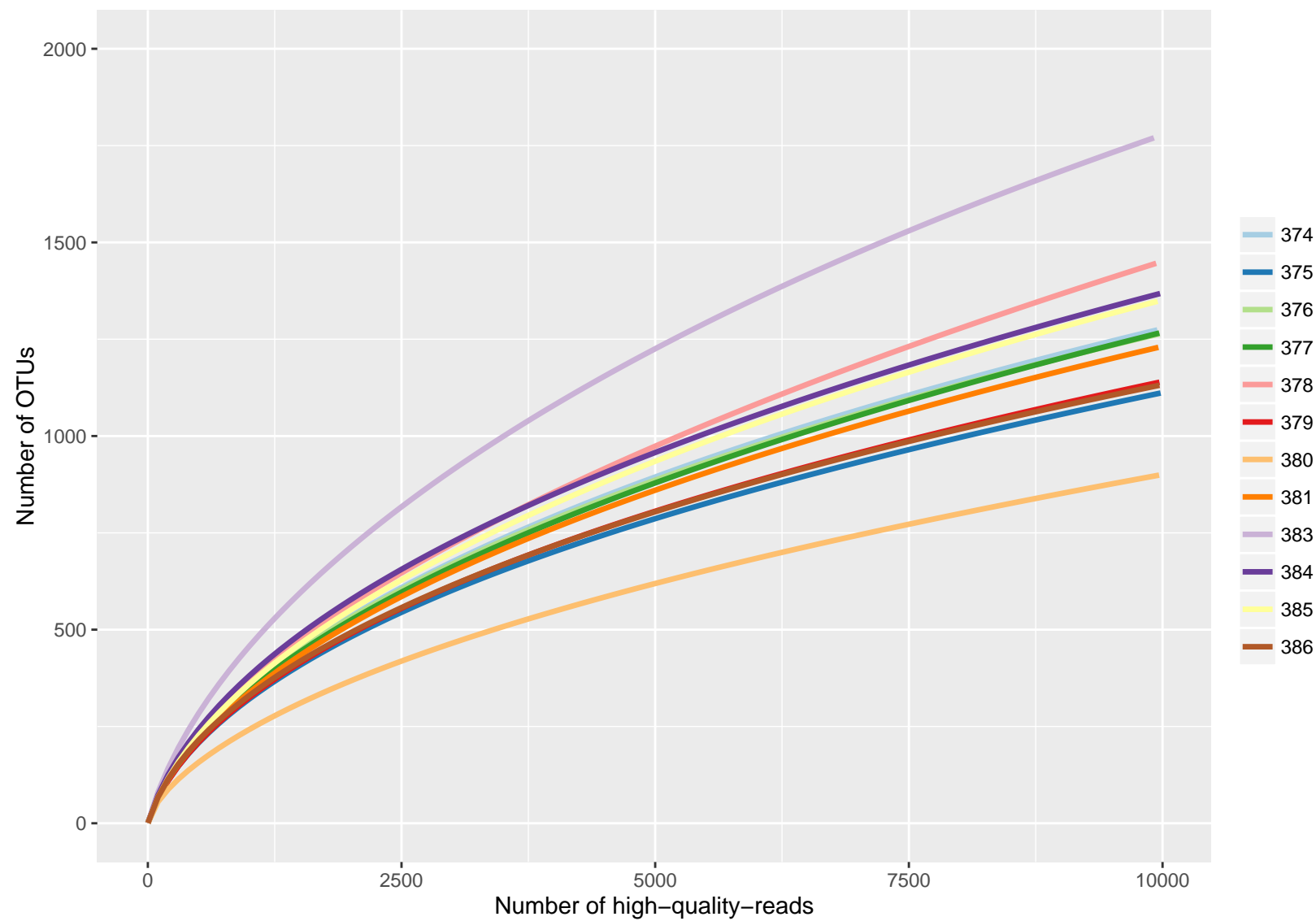

Supplement: S2 File — (ZIP) [file pone.0186766.s008.zip › Rarefact_curves_23.pdf]

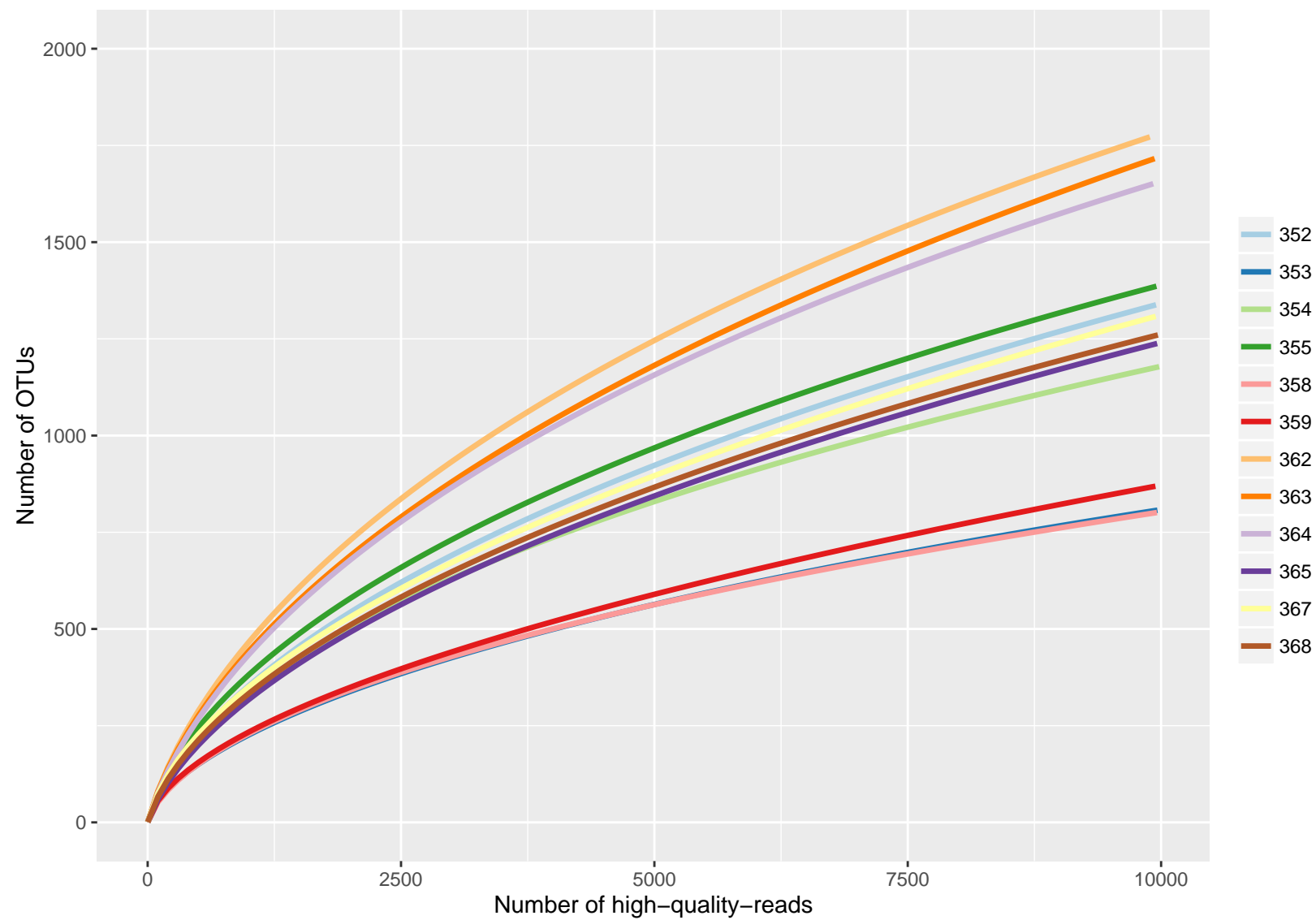

Supplement: S2 File — (ZIP) [file pone.0186766.s008.zip › Rarefact_curves_22.pdf]

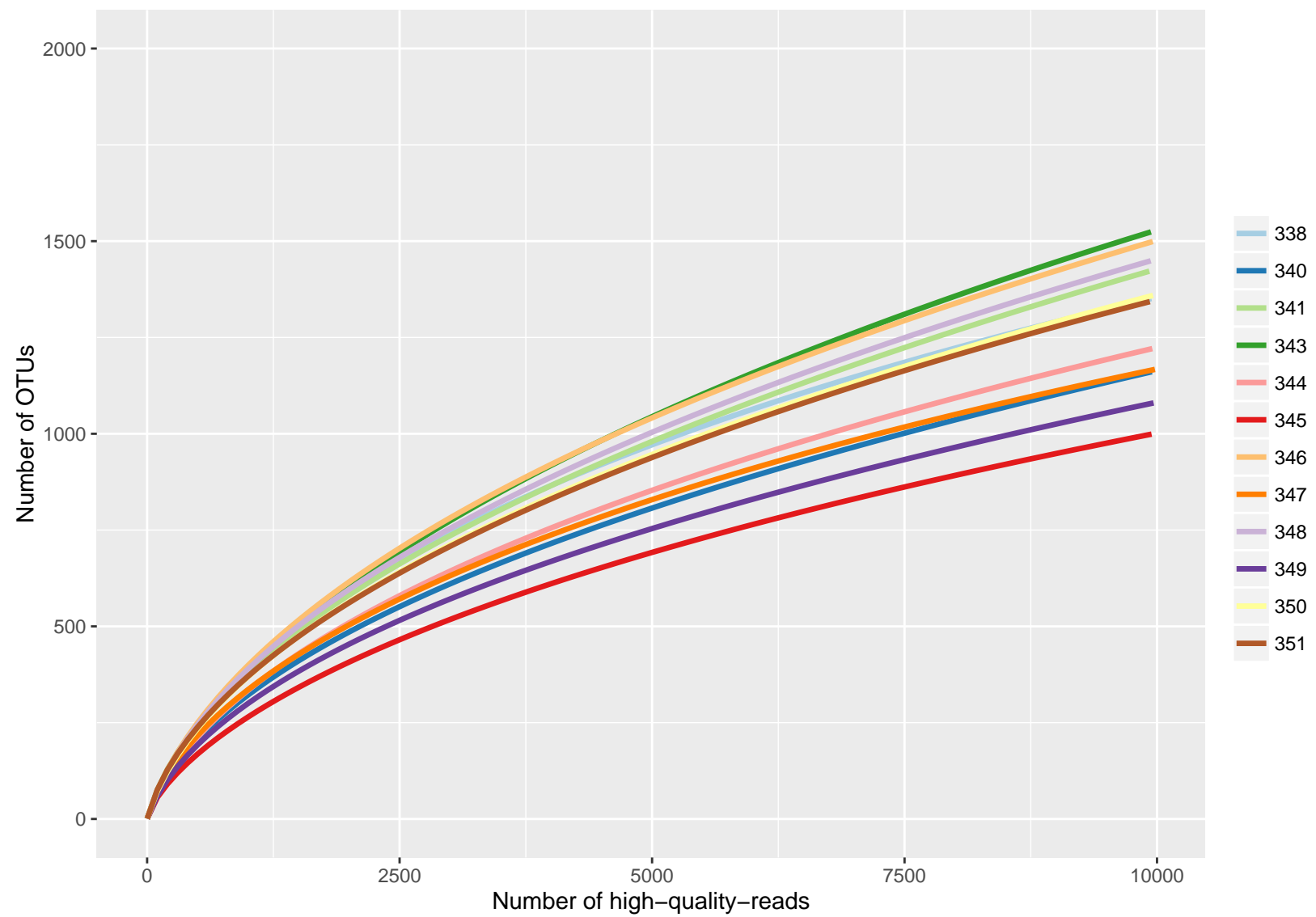

Supplement: S2 File — (ZIP) [file pone.0186766.s008.zip › Rarefact_curves_21.pdf]

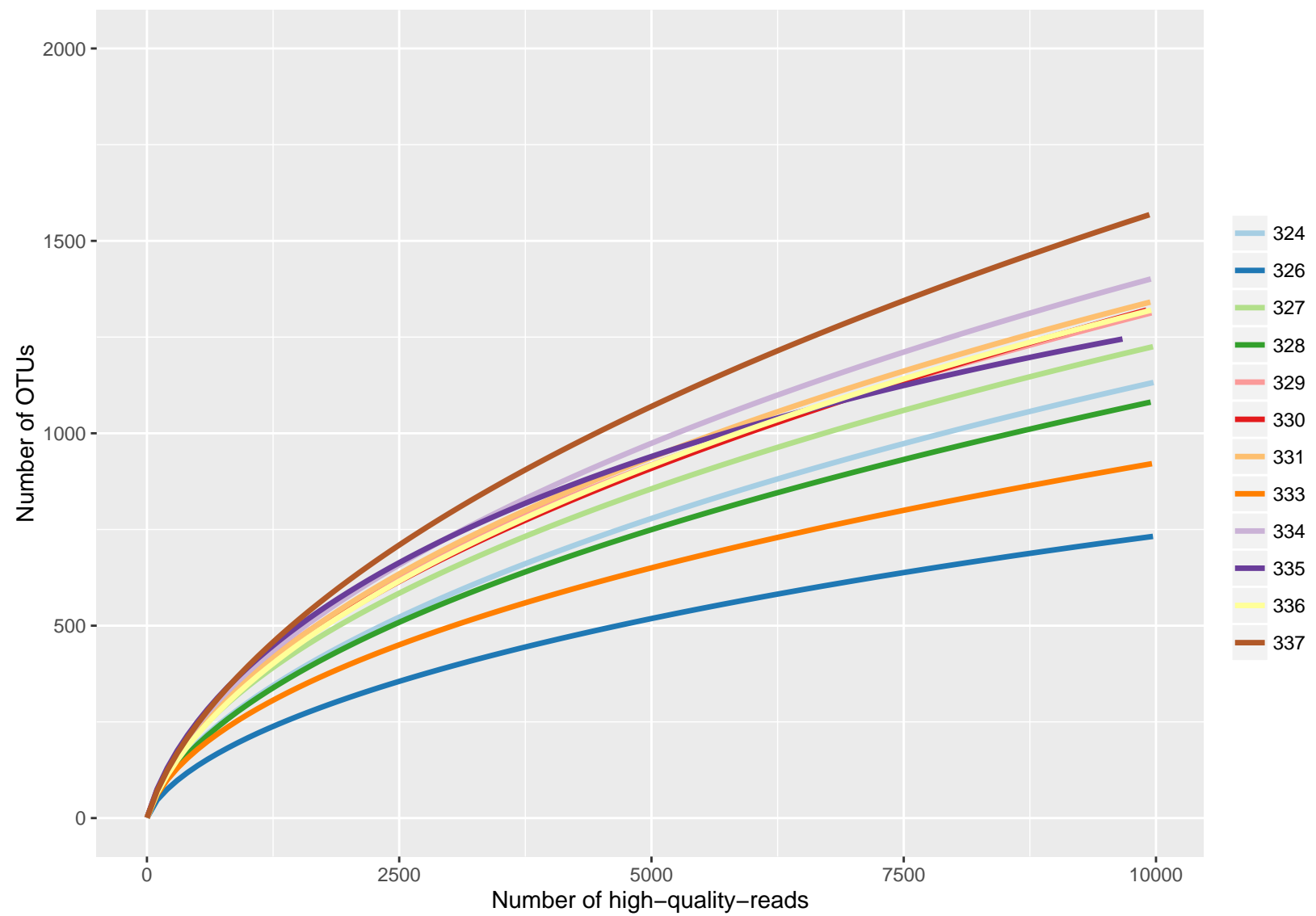

Supplement: S2 File — (ZIP) [file pone.0186766.s008.zip › Rarefact_curves_20.pdf]

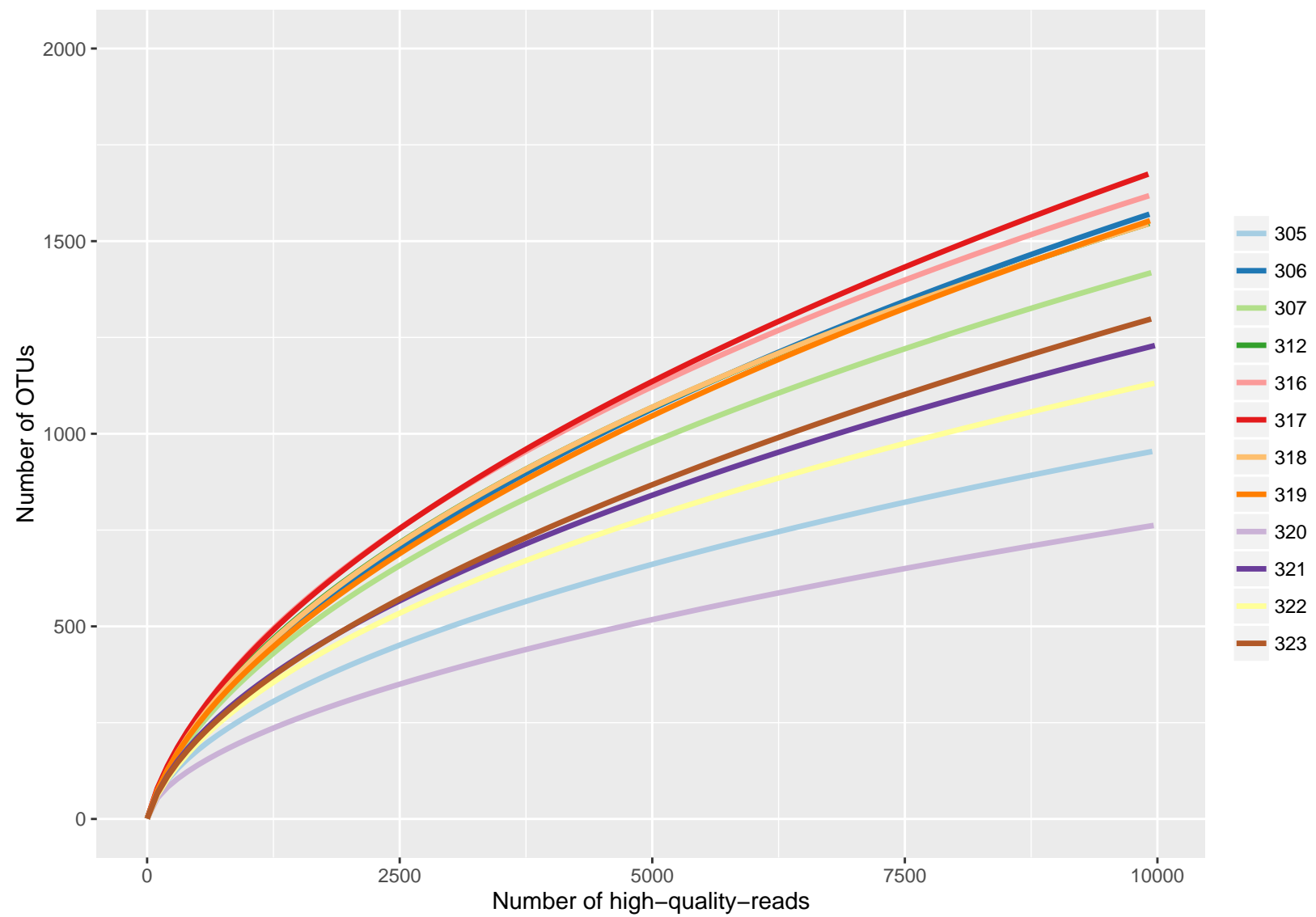

Supplement: S2 File — (ZIP) [file pone.0186766.s008.zip › Rarefact_curves_19.pdf]

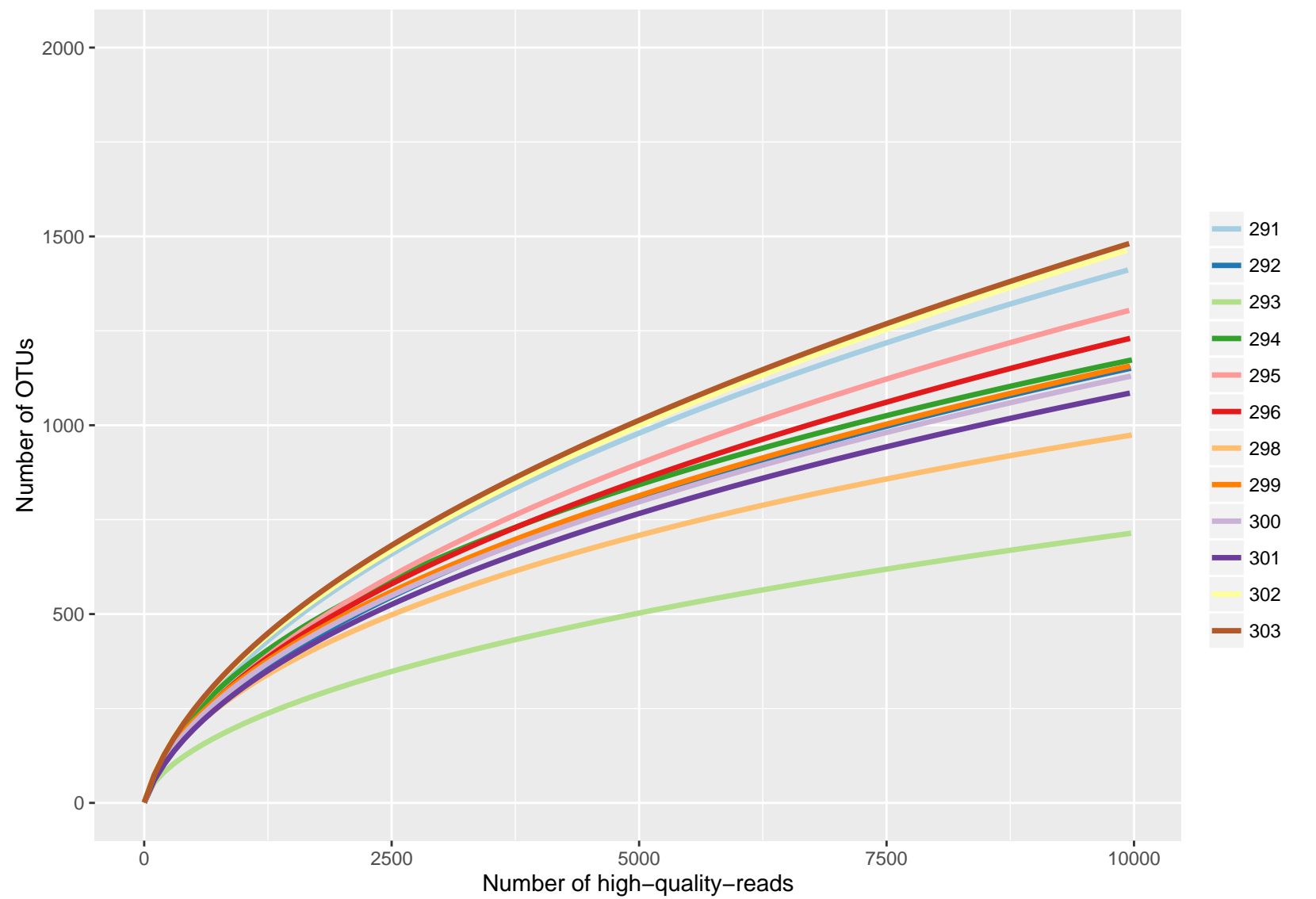

Supplement: S2 File — (ZIP) [file pone.0186766.s008.zip › Rarefact_curves_18.pdf]

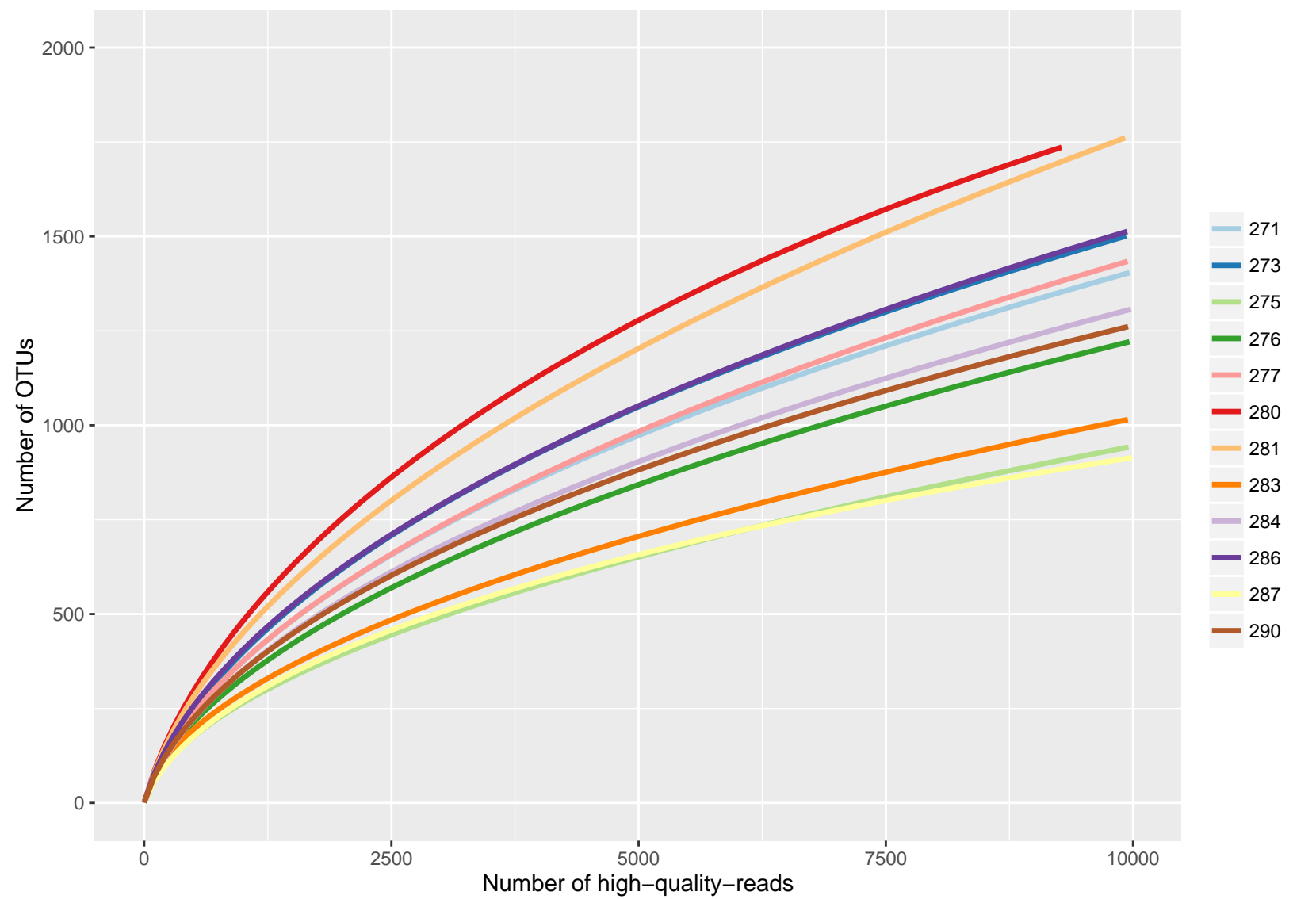

Supplement: S2 File — (ZIP) [file pone.0186766.s008.zip › Rarefact_curves_17.pdf]

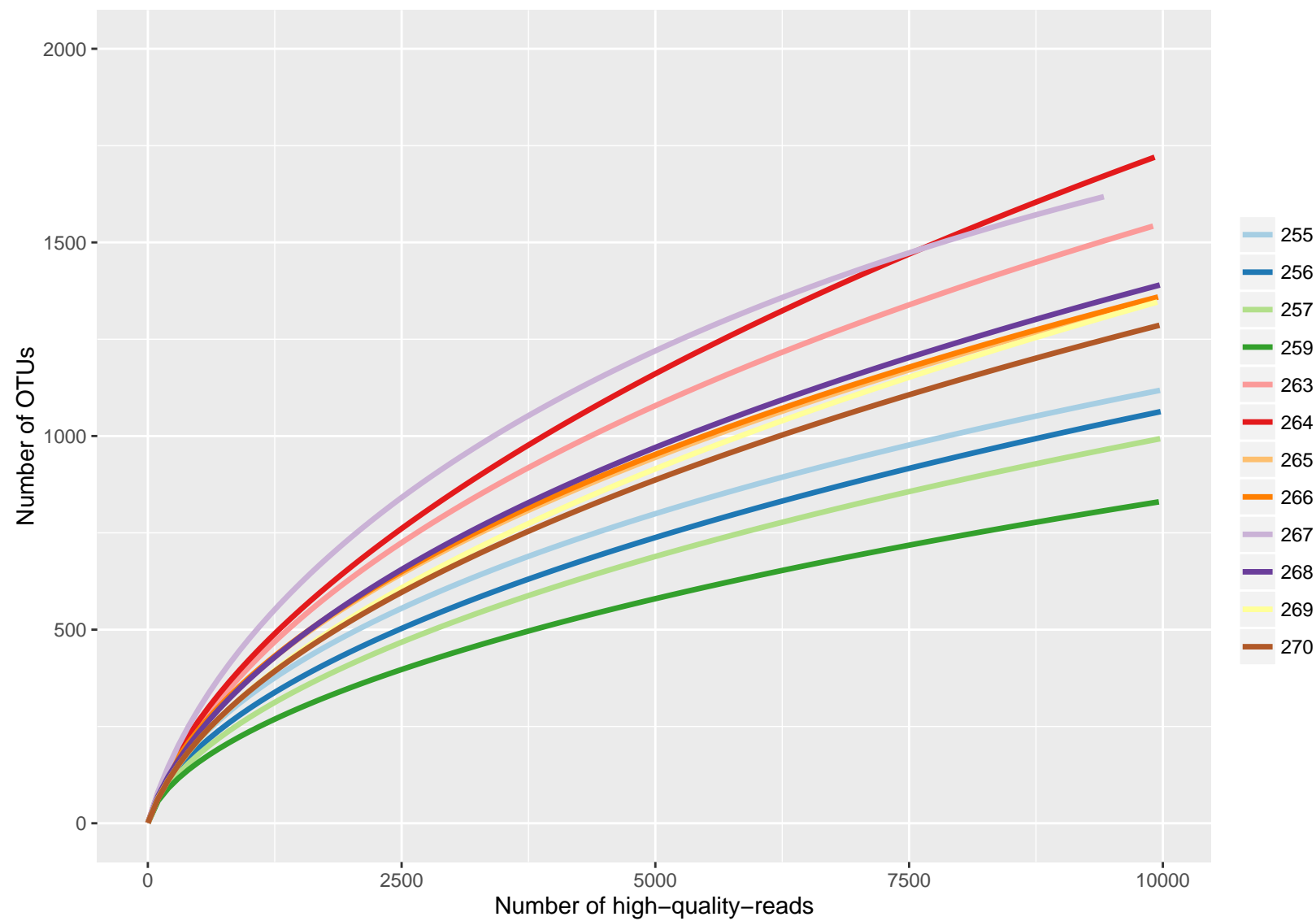

Supplement: S2 File — (ZIP) [file pone.0186766.s008.zip › Rarefact_curves_16.pdf]

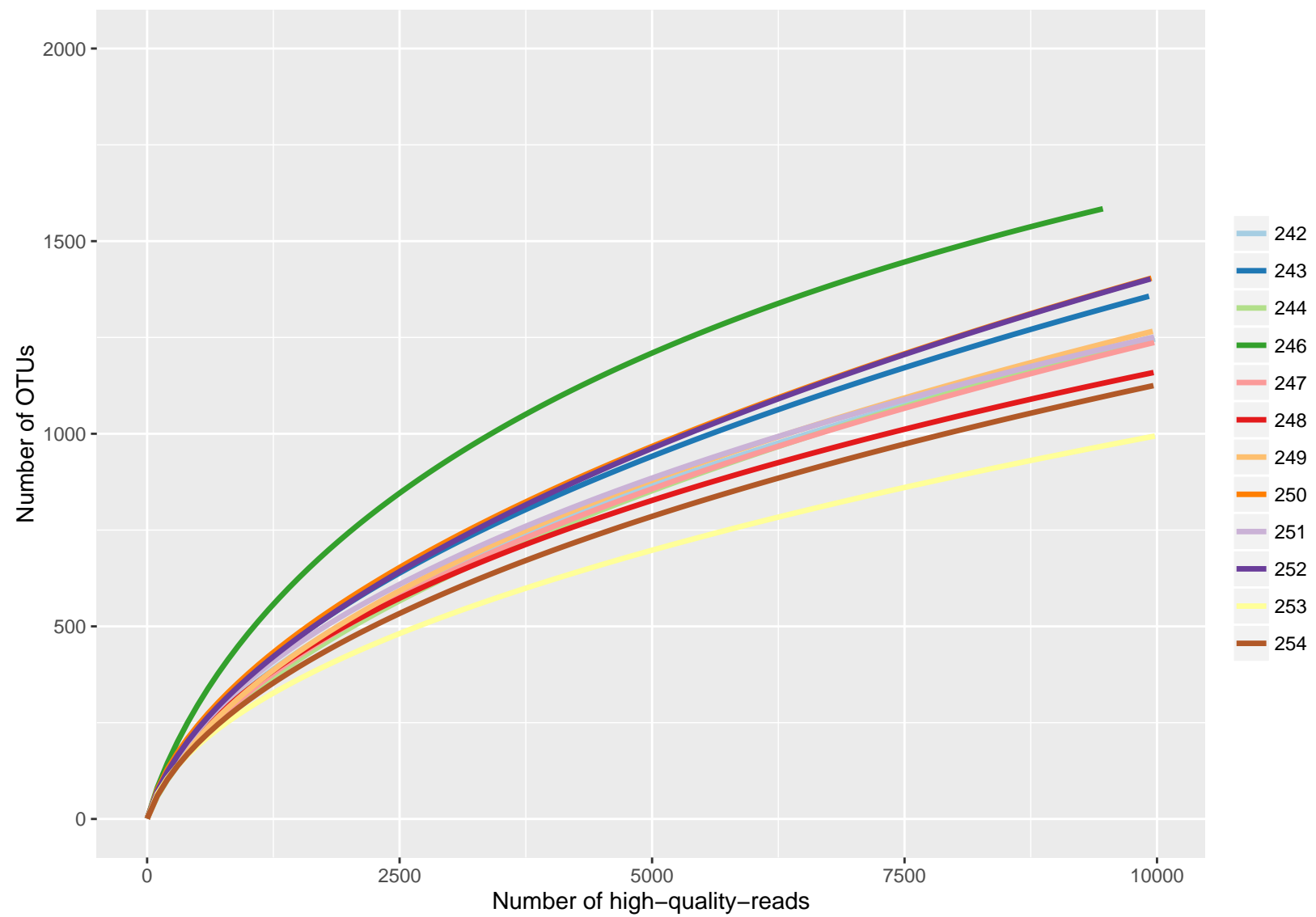

Supplement: S2 File — (ZIP) [file pone.0186766.s008.zip › Rarefact_curves_15.pdf]

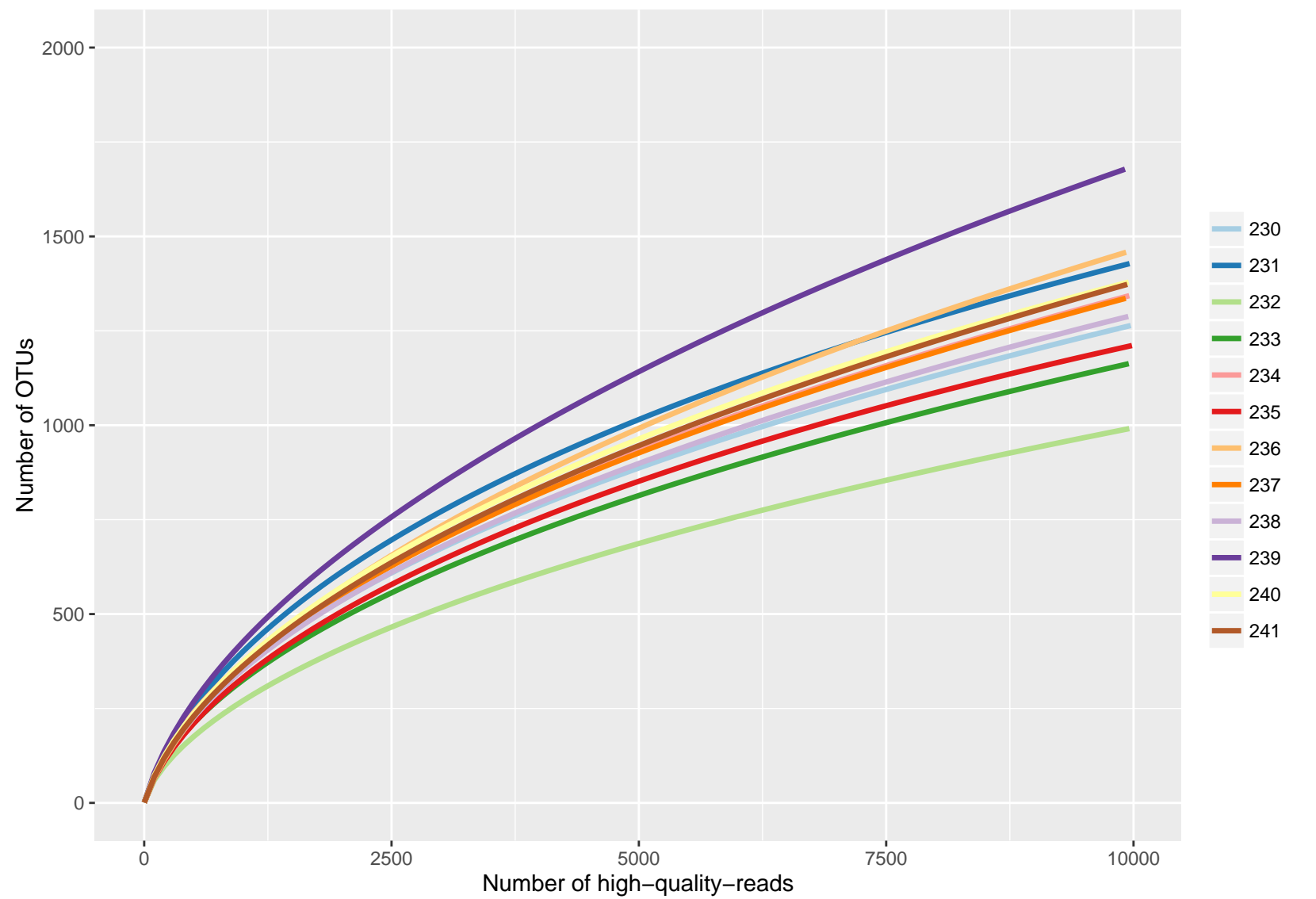

Supplement: S2 File — (ZIP) [file pone.0186766.s008.zip › Rarefact_curves_14.pdf]

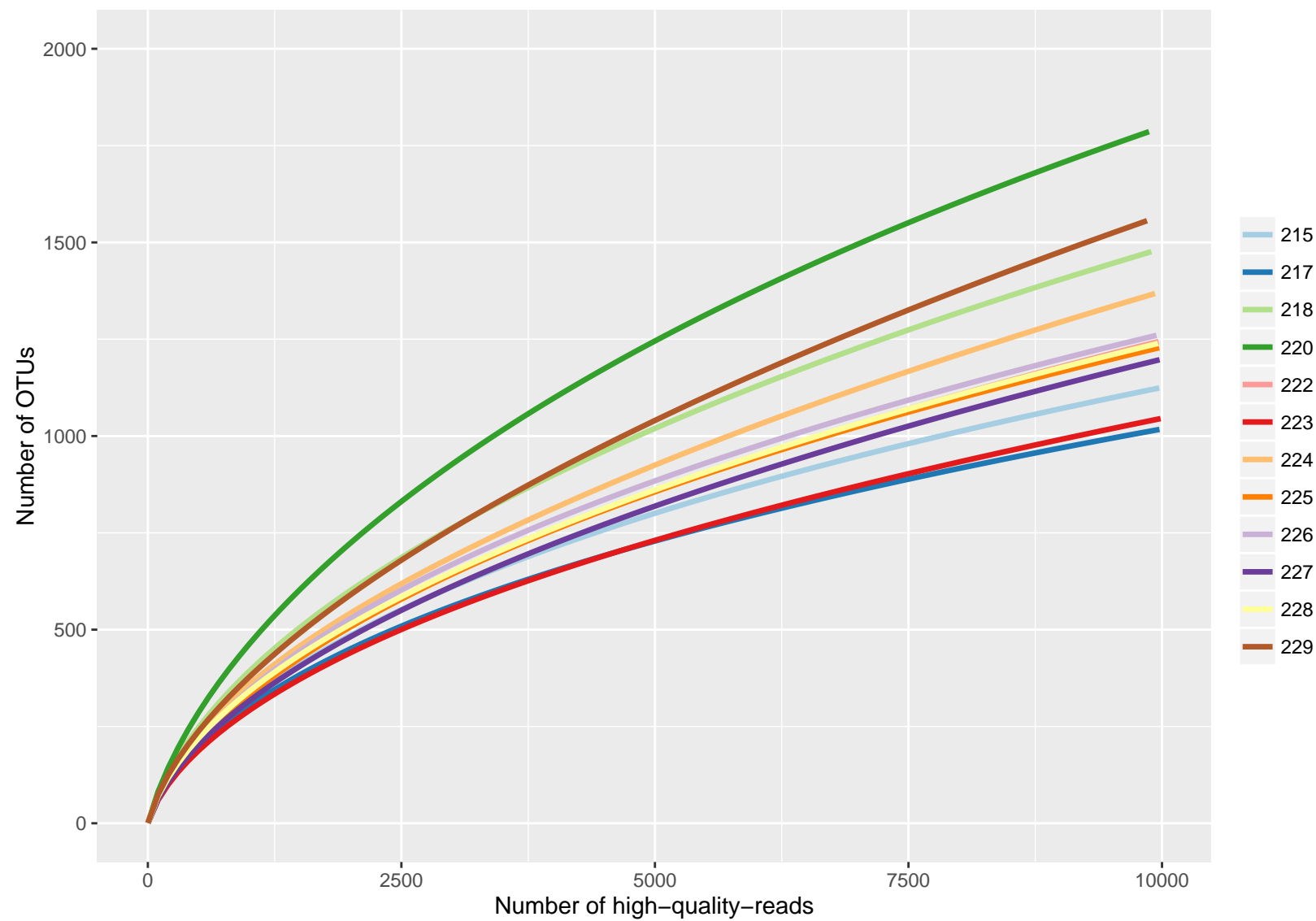

Supplement: S2 File — (ZIP) [file pone.0186766.s008.zip › Rarefact_curves_13.pdf]

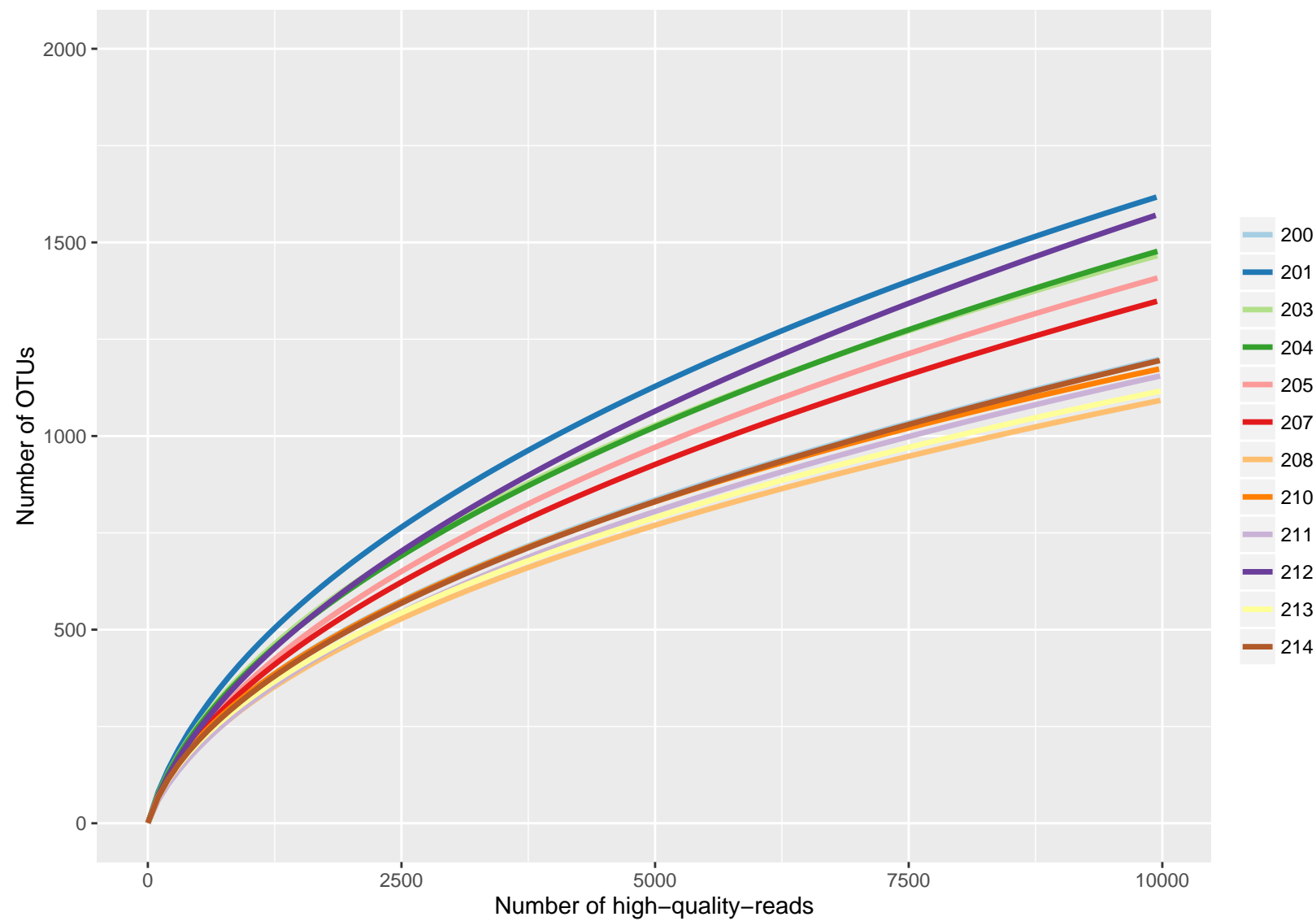

Supplement: S2 File — (ZIP) [file pone.0186766.s008.zip › Rarefact_curves_12.pdf]

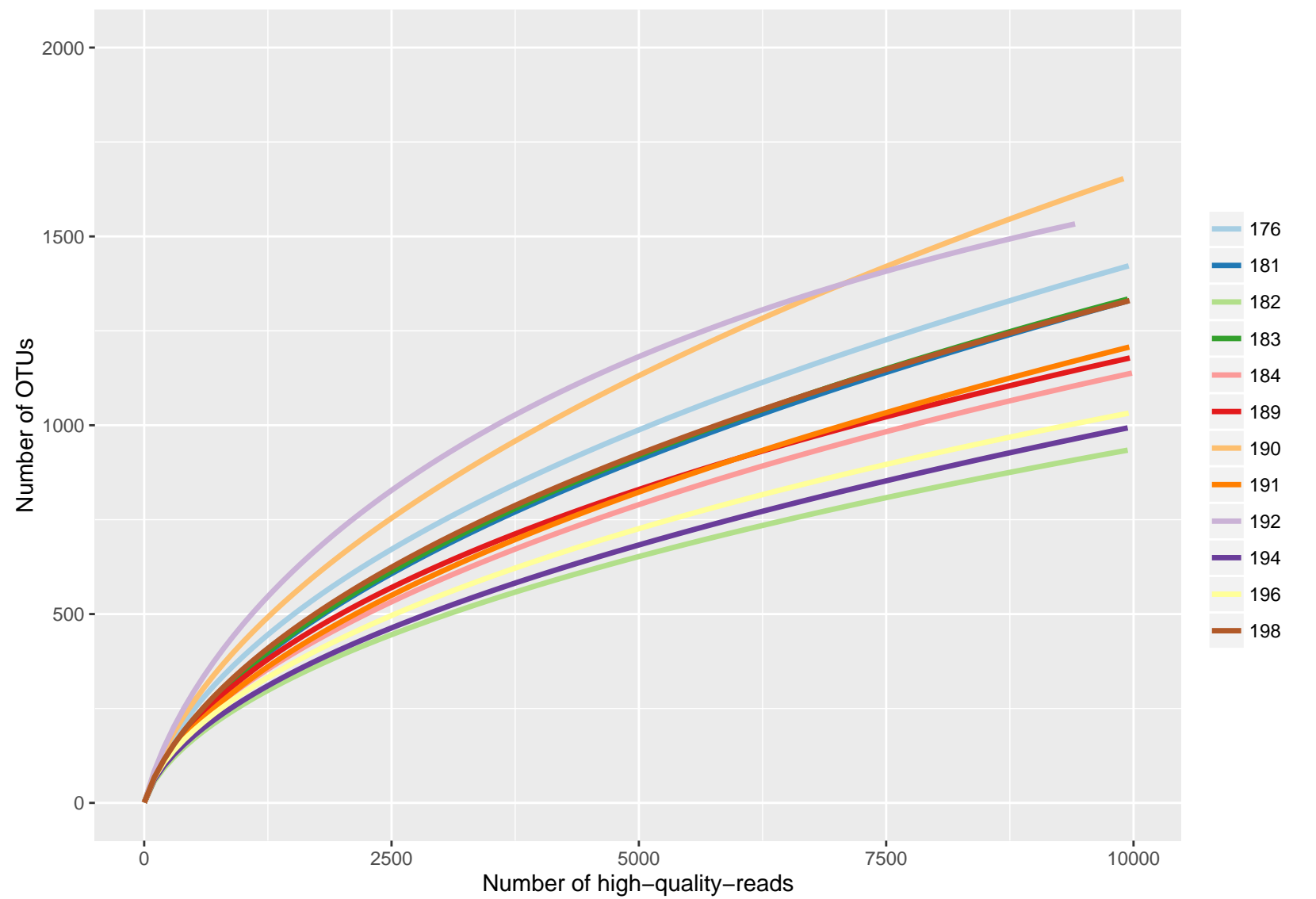

Supplement: S2 File — (ZIP) [file pone.0186766.s008.zip › Rarefact_curves_11.pdf]

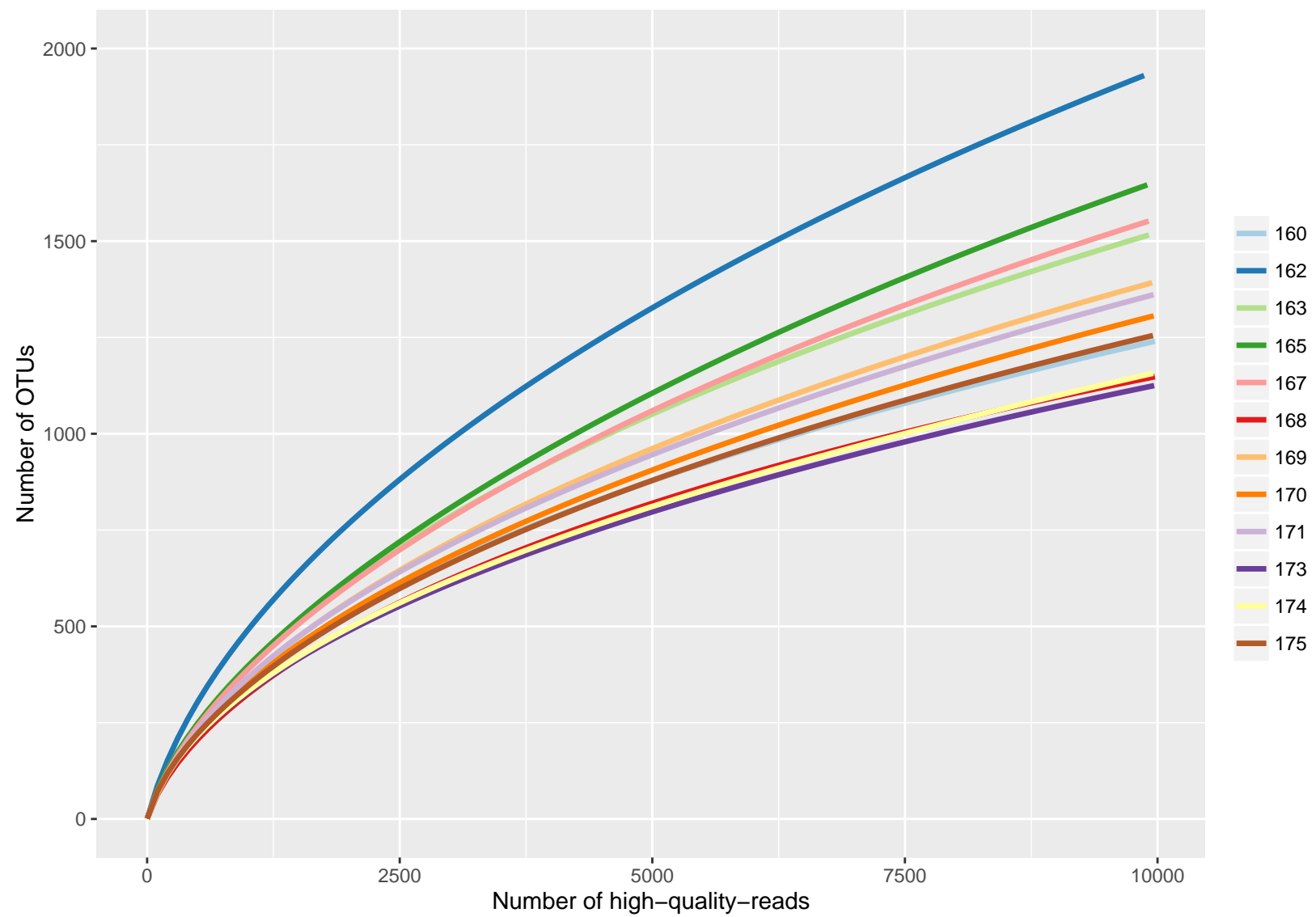

Supplement: S2 File — (ZIP) [file pone.0186766.s008.zip › Rarefact_curves_10.pdf]

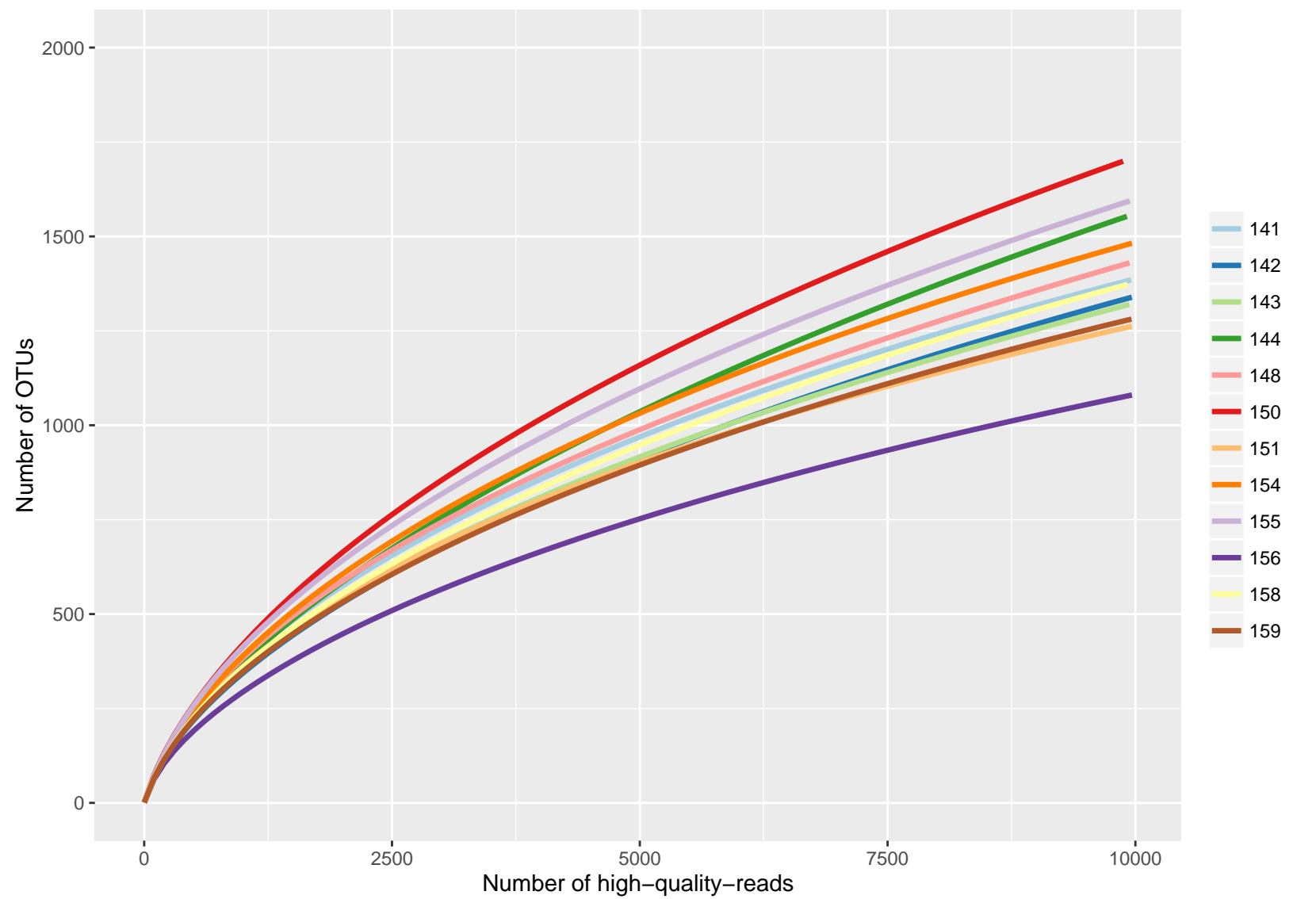

Supplement: S2 File — (ZIP) [file pone.0186766.s008.zip › Rarefact_curves_9.pdf]

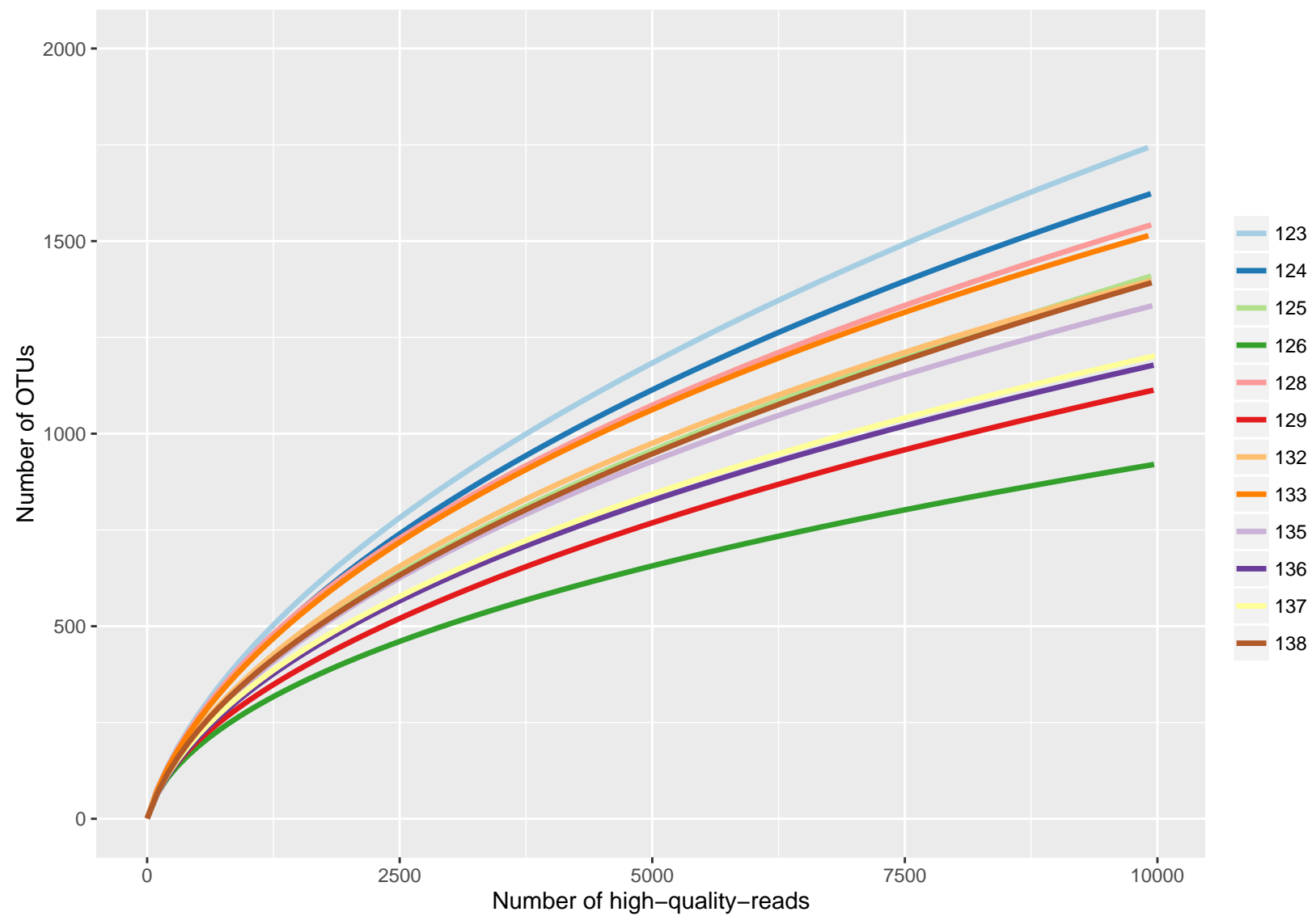

Supplement: S2 File — (ZIP) [file pone.0186766.s008.zip › Rarefact_curves_8.pdf]

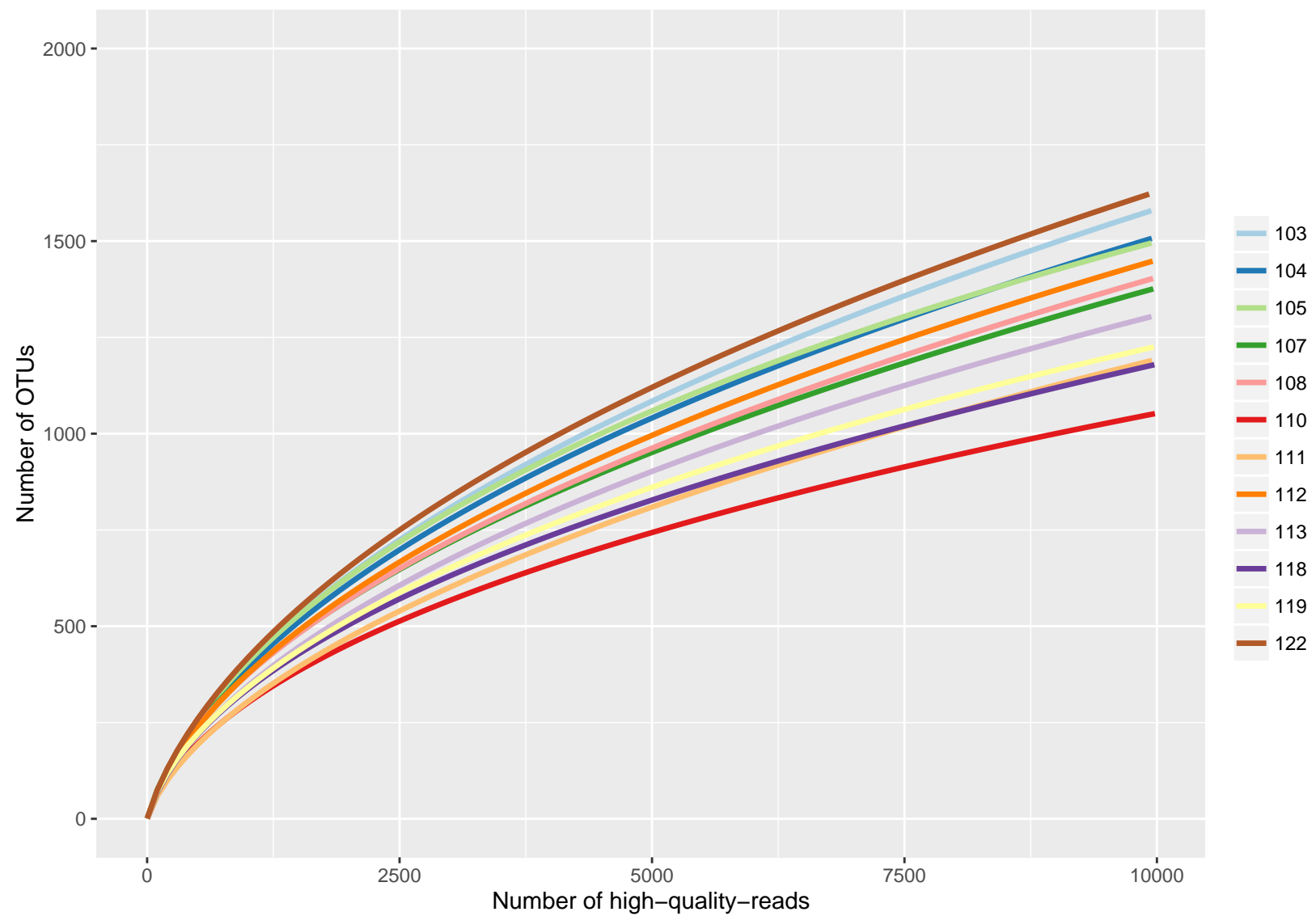

Supplement: S2 File — (ZIP) [file pone.0186766.s008.zip › Rarefact_curves_7.pdf]

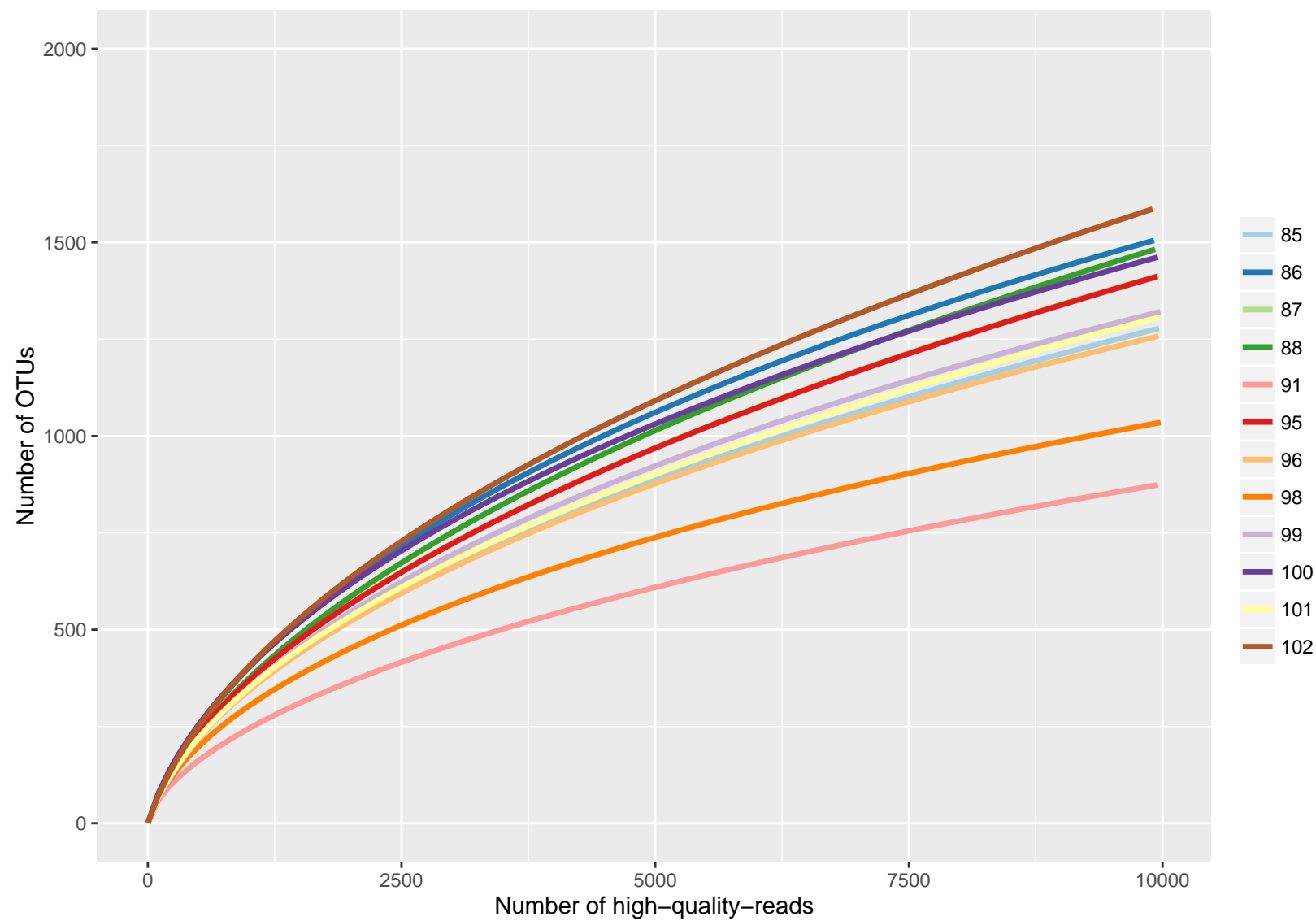

Supplement: S2 File — (ZIP) [file pone.0186766.s008.zip › Rarefact_curves_6.pdf]

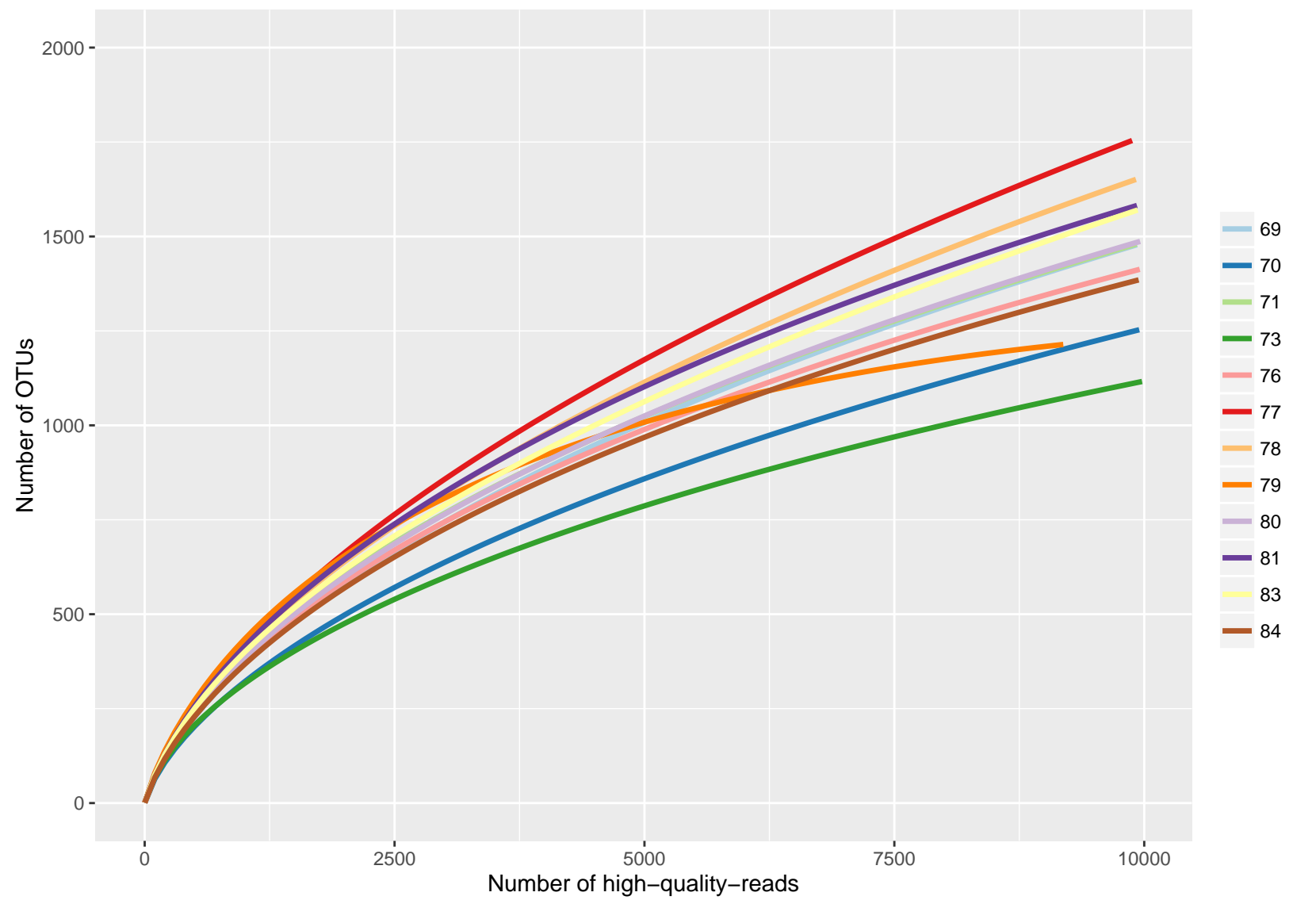

Supplement: S2 File — (ZIP) [file pone.0186766.s008.zip › Rarefact_curves_5.pdf]

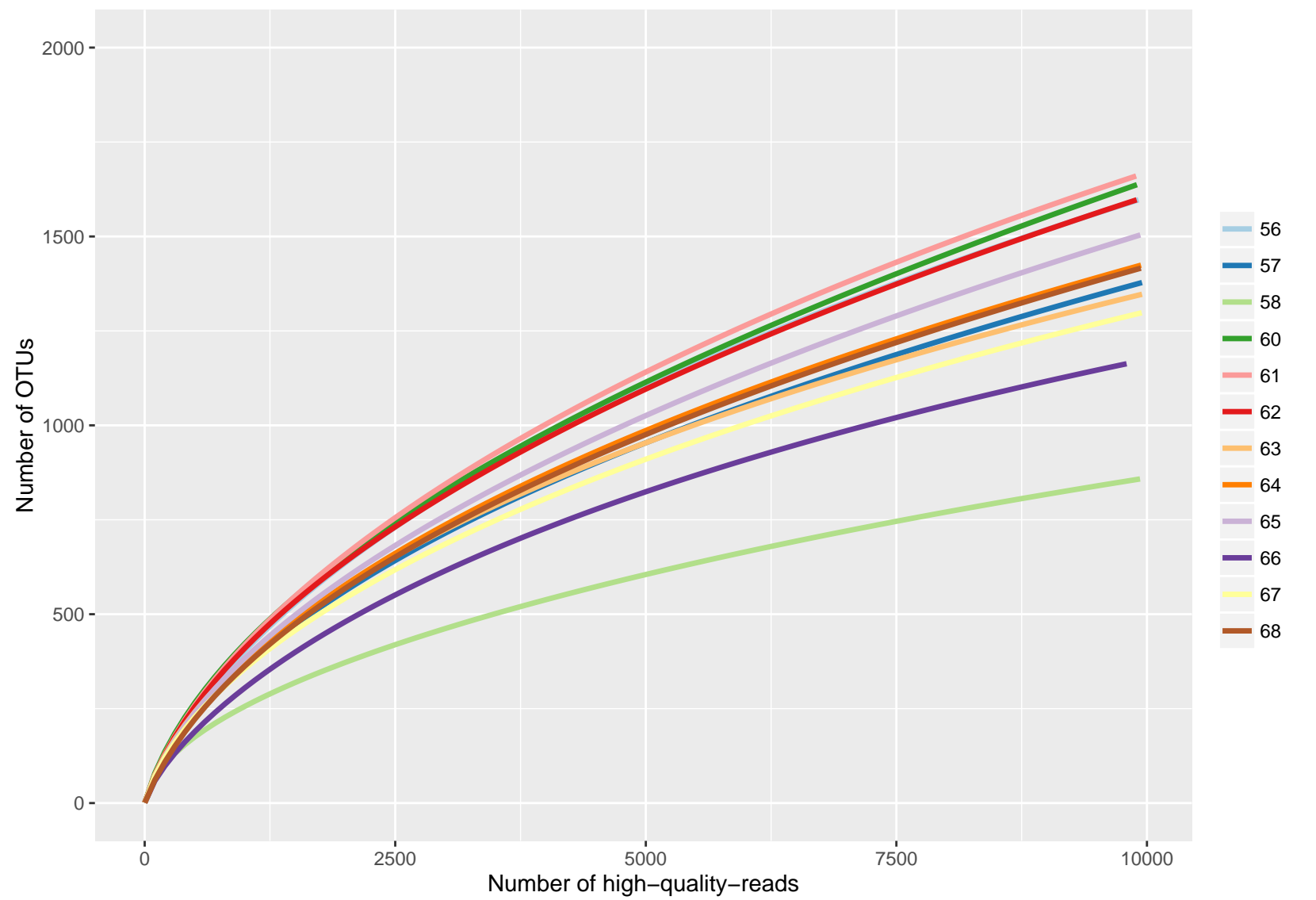

Supplement: S2 File — (ZIP) [file pone.0186766.s008.zip › Rarefact_curves_4.pdf]

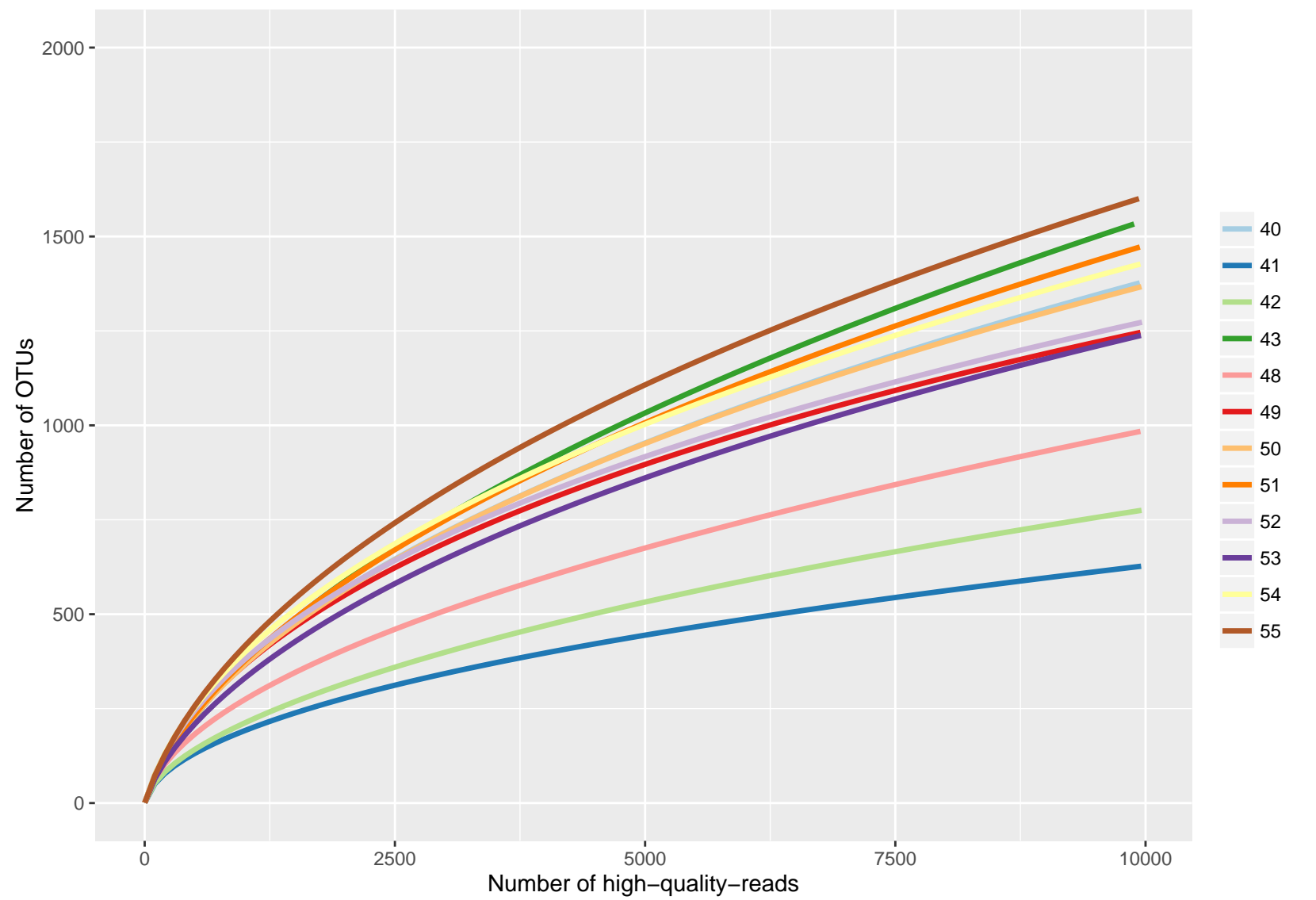

Supplement: S2 File — (ZIP) [file pone.0186766.s008.zip › Rarefact_curves_3.pdf]

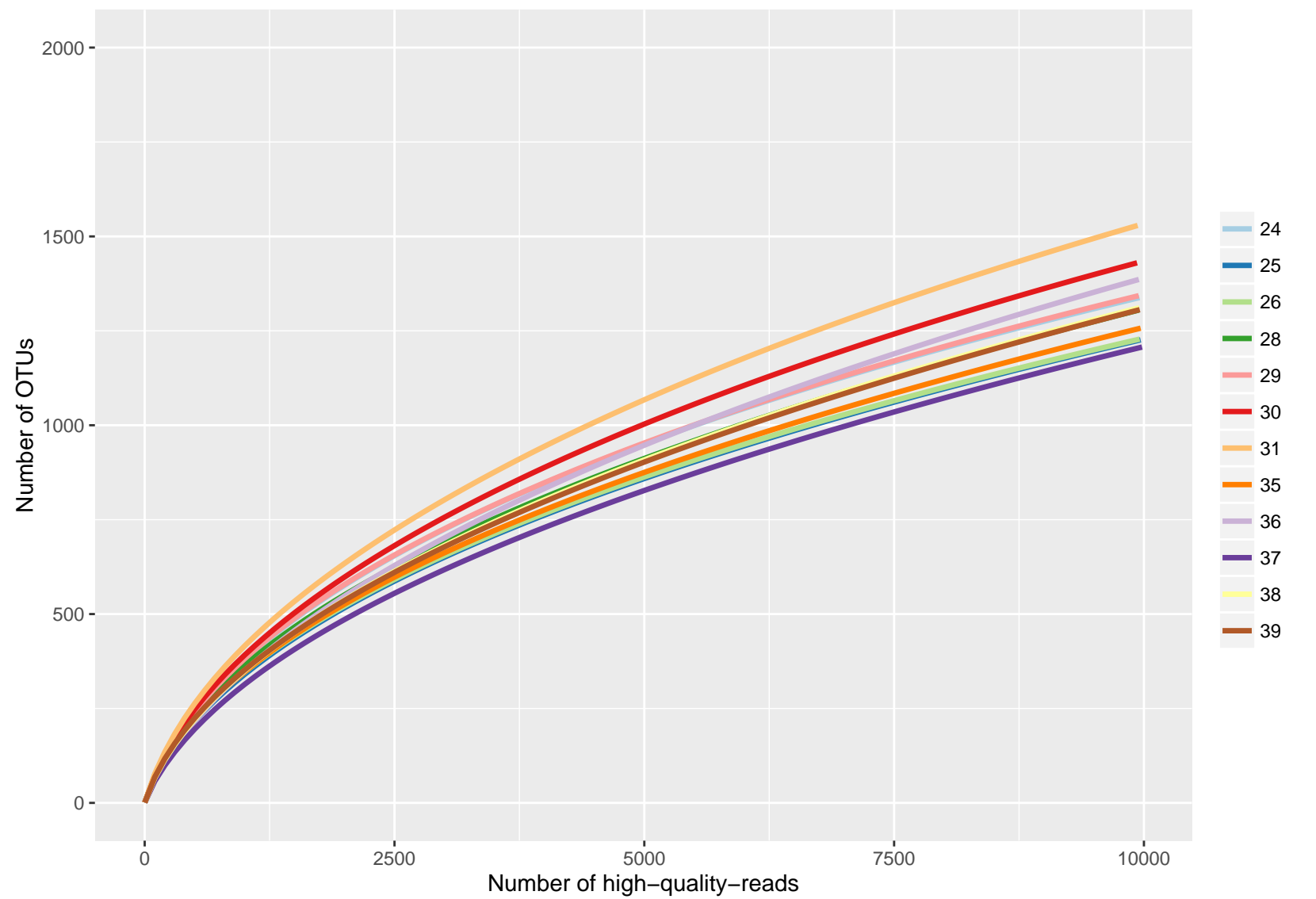

Supplement: S2 File — (ZIP) [file pone.0186766.s008.zip › Rarefact_curves_2.pdf]

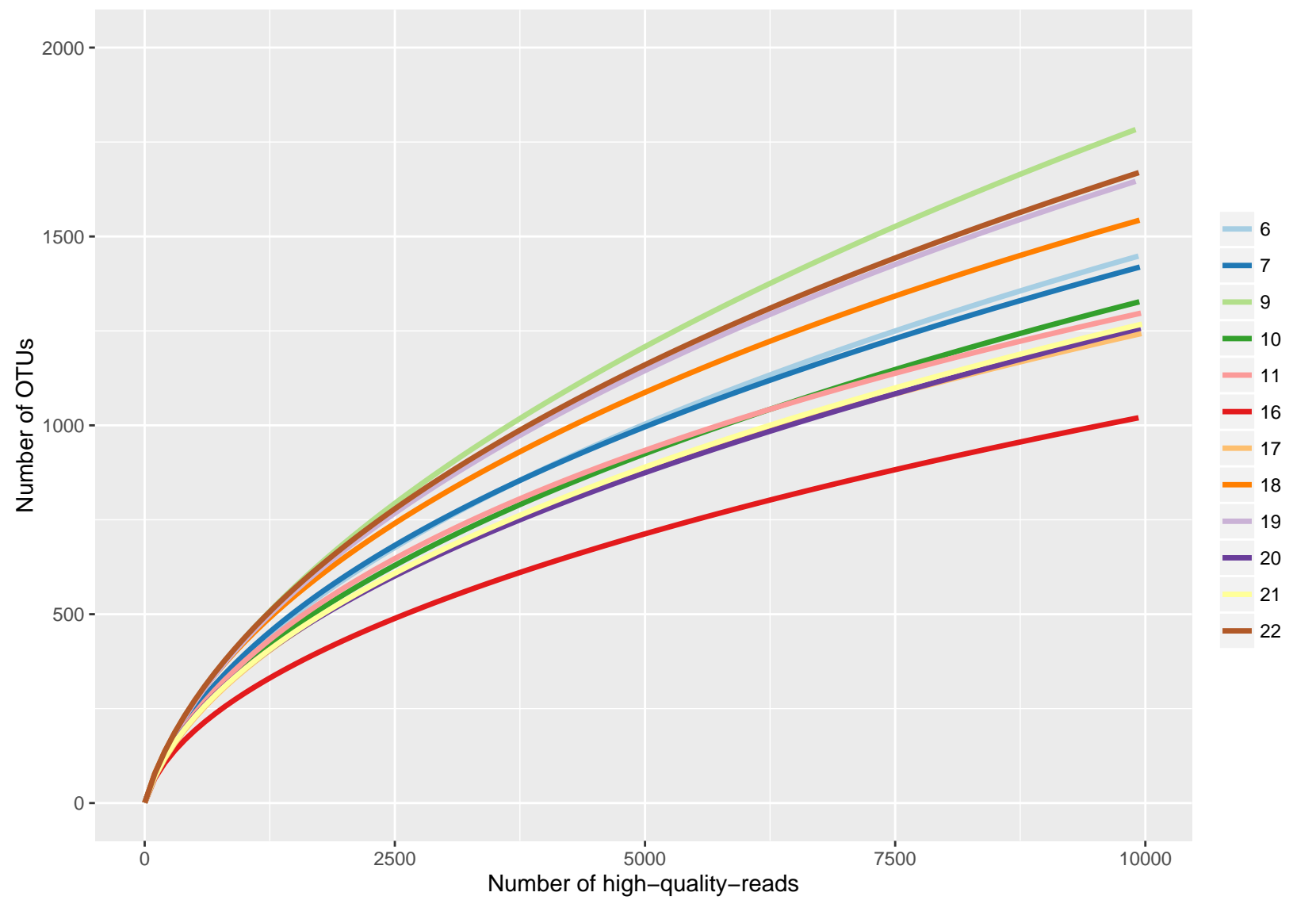

Supplement: S2 File — (ZIP) [file pone.0186766.s008.zip › Rarefact_curves_1.pdf]
